# Molecular and morphologic characterization of *Trichuris trichiura* infecting free-roaming African vervets (*Chlorocebus aethiops sabaeus*) on the Caribbean Island of St. Kitts

**DOI:** 10.1371/journal.pntd.0014539

**Published:** 2026-07-16

**Authors:** Travis Richins, Sarah G. H. Sapp, Jennifer K. Ketzis, Arve Lee Willingham, Samson Mukaratirwa, Yvonne Qvarnstrom, Joel L. N. Barratt

**Affiliations:** 1 Centers for Disease Control and Prevention, Division of Parasitic Diseases and Malaria, Laboratory Science and Diagnostic Branch, Atlanta, Georgia, United States of America; 2 Biomedical Sciences, One Health Center for Zoonoses & Tropical Veterinary Medicine, Ross University School of Veterinary Medicine, Basseterre, Saint Kitts; 3 Department of Veterinary Medicine, College of Agriculture & Veterinary Medicine, United Arab Emirates University, Al Ain, United Arab Emirates; 4 Emory University School of Medicine, Department of Pathology and Laboratory Medicine, Emory University, Atlanta, Georgia, United States of America; Washington University in St Louis School of Medicine, UNITED STATES OF AMERICA

## Abstract

Human trichuriasis is an important disease caused by the soil-transmitted helminth *Trichuris trichiura*, which typically affects human populations in low socioeconomic situations. Natural hosts of *T. trichiura* include humans and certain non-human primates (NHPs), giving rise to concerns that NHPs could serve as zoonotic reservoirs of *T. trichiura* in locations where humans and NHPs exist in proximity. Free-roaming vervets (*Chlorocebus aethiops sabaeus*), introduced from Africa to the Caribbean island of St. Kitts represent cause for concern given their propensity to roam within urban environments. To investigate the potential role of vervets as reservoirs of human trichuriasis, we sought to genotype *T. trichiura* collected from St. Kitts vervets and compare their genotypes to those observed previously from humans and other primates. Thirty-two adult *T. trichiura* and 17 fecal samples containing *T. trichiura* eggs collected from St. Kitts vervets were subjected to molecular analysis, involving deep sequencing of PCR amplicons targeting the 18S ribosomal DNA hypervariable regions I and IV (HVR-I & HVR-IV), and a fragment of the mitochondrial genome spanning part of the small subunit ribosomal RNA gene, the valine transfer RNA gene, and the large subunit rDNA. Morphometric analysis of adult worms demonstrated consistency with previous studies on *T. trichiura* from primate hosts. Phylogenetic analysis showed that *T. trichiura* from St. Kitts vervets belong to a clade that had recently been implicated in human infections and had also been detected in ancient human latrines in Europe. This observation supports that St. Kitts vervets might represent reservoirs for human infection.

## Author summary

This paper seeks to start to answer the question of whether vervet monkeys on the Caribbean island of St. Kitts can transmit *Trichuris trichiura* to the human population. This is especially of interest since we know that some humans on the island are affected by trichuriasis, and that the monkeys co-occur with the human population in time and space. This overlap may afford an opportunity for zoonotic transmission to occur. To investigate this, we utilized a series of PCR reactions targeting several *Trichuris* genes and amplified these genes from *Trichuris* worms and eggs isolated from this vervet population. We sequenced the amplicons using next generation sequencing technology and compared the genotypes identified to those seen previously in humans and other primates. We found that the types of *T. trichiura* in St Kitts vervets genetically matched some types observed previously in humans, supporting that the monkeys could serve as a reservoir for human infections. More evidence will need to be gathered to assess the human-infecting potential of *T. trichiura* from St Kitts vervets, including the collection of *T. trichiura* from humans on St. Kitts to ascertain if those genotypes are a match for those described from vervets.

## Introduction

Human trichuriasis is an important disease predominantly caused by the soil-transmitted helminth (STH) *Trichuris trichiura*. Trichuriasis affects approximately 464 million people worldwide, primarily in tropical regions with poor sanitation, and is associated with a range of symptoms that can be mild (or asymptomatic) in the context of light infections or severe for heavy infections [1]. Heavy infections may result in abdominal pain, fatigue, and protein energy malnutrition, and may progress to *Trichuris* dysentery syndrome where patients experience dysentery, rectal prolapse, anemia, poor growth, clubbing of the fingers, and delays in mental and physical developmental [1,2]. Severe disease is most common in chronically infected children who can have a 90% infection rate in highly endemic areas [1].

Studies previously conducted in Asia, Africa, Europe, and the Caribbean have documented *T. trichiura* infections in animals including the non-human primates (NHP) *Chlorocebus*, *Papio*, and *Macaca* spp. [3–5]. These reports have given rise to concern that NHPs may serve as zoonotic reservoirs of human infection in areas where populations of wild or free roaming NHP overlap with human activity. African vervets (*Chlorocebus aethiops sabaeus*) were introduced by Europeans to the Caribbean island of St. Kitts in the 17^th^ century [6] and continue to flourish there. This population of vervets roam the island freely, where they regularly come into close proximity with humans. Contact between humans and vervets on the island is a public health concern, given the parasites that this population of vervets reportedly harbors including *Trichuris* [3,7]. However, whether the *Trichuris* from St. Kitts vervets is of a strain or species that is capable of infecting humans currently represents an unexplored question.

Earlier investigators suggested that *Trichuris* spp*.* observed in NHPs are *T. trichiura*, with slight morphological differences noted in various hosts attributed in part to morphological plasticity within the species [5]. However, detailed morphological analyses supported by molecular evidence revealed certain morphological characters that distinguish *T. trichiura* from other primate-infecting species (e.g., *T. rhinopiptheroxella, T. colobae,* and *T. ursinus*), and demonstrated that *T. trichiura* is not the sole primate-infecting *Trichuris* [4,5,8]. Several genetic loci have been used in the past to differentiate *Trichuris* species, including the nuclear 18S ribosomal RNA gene, Internal Transcribed Spacers 1 and 2 (ITS1 and ITS2), and the mitochondrial genes cytochrome c oxidase subunit I (*cox1*), cytochrome B (*cyb*), and mitochondrial rDNA large subunit (*rrnL*) [3–5,9].

The present study sought to investigate whether free-roaming St. Kitts vervets are infected with a variety of *Trichuris* previously observed in humans. Using next generation sequencing (NGS) we deep sequenced amplicons of ITS1, ITS2, hypervariable regions (HVRs)- I and IV of the 18S rDNA, and a fragment of the mitochondrial genome spanning the small subunit rDNA gene (*mtSSU*), the tRNA-Val gene, and the large subunit rDNA gene (*rrn*L) from worms and eggs of *T. trichiura* from St. Kitts vervets. A phylogenetic analysis facilitated a direct comparison between the variety of *Trichuris* we describe from St. Kitts with published *Trichuris* genotypes from humans, NHPs, and swine living in Africa, Asia, Europe, and Central America. Additionally, we summarize and harmonize published genotyping data for primate-infecting *T. trichiura* available from the scientific literature and GenBank by developing a rudimentary haplotype naming system for the various 18S rDNA haplotypes observed at HVRs I and II.

## Materials and methods

### Ethics statement

#### Animal ethics.

The collection of vervet fecal specimens and the collection of adult *T. trichiura* from necropsied vervets was approved by the Ross University School of Veterinary Medicine Institutional Animal Care and Use Committee, Tissue/Specimen Use application #TSU10.23.19.

#### Vervet fecal specimens.

The fecal specimens analyzed here are the same as those examined in a previous study of *Strongyloides fuelleborni* in St. Kitts vervets [10]. 17 of the 34 fecal specimens examined in that previous study contained eggs consistent with *Trichuris*. The other 17 did not generate sequence data that were of a high enough quality to be analyzed, but were sequenced in case gel sensitivity was less than sequencing sensitivity. This illustrates the utility of analyzing specimens for multiple studies, increasing the return in investment for each collection, and informing on the incidence of coinfection. Briefly, these fecal specimens had been collected from transport cages of free ranging African vervet monkeys (*Chlorocebus aethiops sabaeus*) trapped on St. Kitts for purposes unrelated to this study. Specimens were placed in 90% ethanol (approximately 1 g of feces to 3 mL of ethanol) and stored at -20°C until shipment to the Division of Parasitic Diseases and Malaria (DPDM) at the US Centers for Disease Control and Prevention (CDC) for analysis. Fecal specimens were stored at 4 °C at CDC prior to subsequent molecular analyses.

#### Collection of adult Trichuris.

Molecular and morphologic analysis of adult *T. trichiura* collected from three vervet monkeys on St. Kitts (Animal IDs: 302480-10-5, 402796-3-4, and 202785-6-1) was also performed. Adult *T. trichiura* were observed incidentally in these animals during a necropsy performed as part of work unrelated to the present study. The worms were placed in 90% ethanol and stored at -20°C until shipment to DPDM for subsequent molecular and morphologic analysis. In all, 10 adult *Trichuris* were obtained from vervet 302480-10-5, 15 from vervet 402796-3-4, and 10 from vervet 202785-6-1.

#### Morphological analysis of adult worms.

*Trichuris* adults were subjected to morphometric analysis of various attributes previously investigated for *Trichuris* of non-human primates [4,5,11–13]. Adult worms in 90% ethanol were cleared via progressive glycerol concentration, examined first using a dissecting microscope (Olympus SX74) and in further detail under differential interference contrast (DIC) on a compound microscope (Olympus BX51). Relevant features were photographed and measured, specimen condition permitting, using Olympus cellSens Standard software (v. 2.3).

#### DNA extraction.

Following morphometric analysis, DNA was extracted from whole adult worms using a Qiagen Blood and Tissue DNA extraction kit (Qiagen, Germantown, Maryland, USA) following the ‘Animal Tissue Protocol’. Briefly, at step 1, each adult worm was added to 180 µL of buffer ATL and 20 µL of proteinase K. This solution was incubated at 56ºC until the entire worm was completely dissolved. The protocol continued in accordance with the ‘Animal Tissue Protocol’ ending with elution of DNA in 200 µL of elution buffer. DNA extracts were stored at -20ºC prior to molecular analysis. For fecal specimens, the same DNA extracts were used as in our previously published study and it was extracted using a DNeasy PowerSoil Kit (Qiagen) with some minor modifications as described by Richins, Sapp [10].

#### Polymerase Chain Reaction and Illumina sequencing.

Five *Trichuris* loci (Table 1) were PCR amplified from each DNA extract. Reactions were prepared to contain a final concentration of 0.5 µM of each forward and reverse primer, 25 µL of NEB Next Q5 Hot Start HiFi PCR Master Mix (New England Biolabs, Ipswich, MA, USA), and 2 µL of template DNA in a total volume of 50 μL. Thermal cycling conditions included an initial melt at 98°C for 2 minutes followed by 45 cycles of 98°C for 10 seconds, annealing between 59°C and 68°C (annealing varies by primer set; see Table 1) for 10 seconds, and extension 72°C for 10 seconds. The protocol ended with a final extension step of 72°C for 2 minutes, and holding at 4°C. All PCR runs were accompanied by a negative template control (PCR grade water instead of DNA). For PCR runs containing fecal DNA extracts, DNA extracted from adult *Trichuris* worms served as a positive control. Amplicons were visualized on a 2% agarose using E-gel Power Snap Electrophoresis system (Thermo Fisher Scientific Waltham, Massachusetts, USA) to confirm expected amplicon size prior to sequencing. Prior to sequencing, amplicons from the same sample were pooled. The pooled amplicons were purified with the SequalPrep Normalization Kit (Thermo Fisher Scientific Waltham, Massachusetts, USA), library prepared with the Nextera XT DNA Library Prep Kit (Illumina, San Diego, California, USA) and sequenced on the MiSeq Platform using the MiSeq Reagent Kits V2 (500 cycle) (Illumina) using the same methods described previously by Richins, Sapp [10].

**Table 1:** PCR primers and annealing temperatures.

Target locus	Primer pair	Amplicon Length	Primer sequence	Annealing temperature
Mt locus†	Trich_tRNA-Val_LSU_F Trich_tRNA-Val_LSU_R	~650 bp ‡	5’-GAGGTAAGTCGTAACAAAGTAGAT-3’ 5’-TTTAAAATTAATAAACAAATGATTATGCTACCTT-3’	59ºC
HVR-I	Trichuris_18S_HVR1_F Trichuris_18S_HVR1_R	~ 850 bp ‡	5’-TGGAAATGCTAGAGCTAATACATGCCT-3’ 5’-TCGTTCTTGATTAATGAAAACATTCTTGACAA-3’	65ºC
HVR-IV	Trichuris_18S_HVR4_F Trichuris_18S_HVR4_R	~ 450 bp ‡	5’-TCTTGATTCAGTGGGTAGTGGTGC-3’ 5’-GCTGATGACTCGCGCTTACTGG-3’	63ºC
ITS1	Trichuris_ITS1_F Trichuris_ITS1_R	~350 bp ‡	5’-CGTAGCTGCCGTGCGTTGT-3’ 5’-GCACGGTCGACTGCAACC-3’	67ºC
ITS2	Trichuris_5.8_ITS2_F Trichuris_5.8_ITS2_R	~650 bp ‡	5’-TCGTCGCCGGTTGGAA-3’ 5’-AACGTTTGCCGCCGTC-3’	68ºC

†Amplicon captures part of the mitochondrial SSU RNA gene, the complete tRNA-Val gene, and part of the *rrnL* gene.

‡ Length varies between haplotypes.

### Mitochondrial (Mt) DNA and 18S rDNA analysis

#### *Trichuris* 18S rDNA haplotypes.

A convention for naming HVR-I and HVR-IV haplotypes from primate-infecting *T. trichiura* (with some exceptions – see Tables 2 and 3) was established using sequences from GenBank, the National Center for Biotechnology Information (NCBI) Sequence Read Archive (SRA), and sequences generated in this study. *Trichuris muris* 18S rDNA haplotypes are also listed in Tables 2 and 3 (designated the name ‘TMUE’), to establish an outgroup for the subsequent phylogenetic analysis. Using the naming system outlined in Tables 2 and 3, an 18S rDNA genotype was assigned to all specimens for which 18S rDNA sequences were available. Genotype assignments were made via BLASTN comparison of *Trichuris* 18S rDNA haplotypes from Tables 2 and 3 to 18S rDNA sequences from the reference genotypes defined below, and those generated here from vervet *T. trichiura*. The sequence of each 18S haplotype is provided in S2 File.

**Table 2:** Summary of primate-infecting *Trichuris* species HVR-I haplotype naming scheme.

Haplotype	GenBank/SRA Accessions	Species designation	Host/s	Location	Reference
I	PX401234, ERR9805794, ERR9805795, ERR9805813, ERR9805816, ERR9805819, ERR9805821, ERR9805822, ERR9805783, ERR9805782, ERR9805781, ERR9805780	*Trichuris trichiura*	African vervet, *Papio hamadryas* (Denmark, captive), Human	St. Kitts, Denmark, Uganda, China	Present study, Doyle, Soe [14]
II	PX401235, LC596914.1, GQ352551.1, GQ352548.1, DQ118536.1, JF690953.1†, ERR9805831, ERR9805794, ERR9805795, ERR9805811, ERR9805812, ERR9805813, ERR9805815, ERR9805816, ERR9805817, ERR9805819, ERR9805820, ERR9805821, ERR9805822, ERR9805814, ERR9805783, ERR9805782, ERR9805781, ERR9805780, ERR9805779, ERR9805805, ERR9805803	*Trichuris trichiura*	African vervet, human, *Papio hamadryas*, Human	St. Kitts, Czech Republic, Japan, Thailand, Denmark (ancient latrine), Denmark (captive Papio hamadryas), Uganda, China, Honduras	Present study, Dolezalova, Obornik [15], Areekul, Putaporntip [16], Ishizaki, Kawashima [17], Doyle, Soe [14]
III	PX401236	*Trichuris trichiura*	African vervet	St. Kitts	Present study
IV	GQ352549.1	*Trichuris trichiura*	Human	Thailand	Areekul, Putaporntip [16]
V	PX401237	*Trichuris trichiura*	African vervet	St. Kitts	Present study
VI	GQ352547.1	*Trichuris trichiura*	Human	Thailand	Areekul, Putaporntip [16]
VII	PX401238	*Trichuris trichiura*	African vervet	St. Kitts	Present study
VIII	AB699092.1	*Trichuris trichiura*	Japanese macaque	Japan	Arizono, Yamada [18]
IX	GQ352555.1	*Trichuris trichiura*	Human	Thailand	Areekul, Putaporntip [16]
X	GQ352553.1	*Trichuris trichiura*	Human	Thailand	Areekul, Putaporntip [16]
XI	GQ352552.1	*Trichuris trichiura*	Dog ‡	Thailand	Areekul, Putaporntip [16]
XII	GQ352554.1	*Trichuris trichiura*	Human	Thailand	Areekul, Putaporntip [16]
XIII	GQ352550.1	*Trichuris trichiura*	Human	Thailand	Areekul, Putaporntip [16]
XIV	JX049338.1	*Trichuris* sp.	African vervet	Czech Republic (captive animal)	Dolezalova, Obornik [15]
XV	JF690957.1	*Trichuris* sp.	Lion-tailed macaque	Czech Republic (captive animal)	Dolezalova, Obornik [15]
XVI	JF690955.1	*Trichuris* sp.	Olive baboon	Czech Republic (captive animal)	Dolezalova, Obornik [15]
XVII	JX049339.1	*Trichuris* sp.	Rhesus macaque	Czech Republic (captive animal)	Dolezalova, Obornik [15]
XVIII	ERR9805786, PX401239	*Trichuris trichiura*	Francois’ leaf-monkey	China (captive animal)	Doyle, Soe [14]
XIX	ERR9805812, PX401240	*Trichuris trichiura*	Human	Uganda (Danish traveller)	Doyle, Soe [14]
XX	ERR9805814, PX401241	*Trichuris trichiura*	Human	Uganda (Ugandan child)	Doyle, Soe [14]
XXI	ERR9805811, PX401242	*Trichuris trichiura*	Human	Uganda (Danish traveller)	Doyle, Soe [14]
XXII	ERR9805831, PX401243	*Trichuris trichiura*	Ancient DNA	Denmark (Ancient latrine)	Doyle, Soe [14]
XXIII	ERR9805779, PX401244	*Trichuris trichiura*	Human	China	Doyle, Soe [14]
XXIV	HF586906.1, ERR9805796, ERR9805797	*Trichuris colobae*	*Colobus guereza kikuyensis*	Spain (captive animal)	Callejon, Nadler [9], Doyle, Soe [14]
TMUE	HF586907.1, HF586908.1, PRJEB126§	*Trichuris muris*, *Trichuris arvicolae*	Mouse, vole	Spain	Callejon, Nadler [9]

† The sequence under this accession is truncated relative to the region amplified by PCR here but it matches the assigned haplotype at the region captured.

‡ Dogs are an atypical host for *T. trichiura* though the sequence is derived from *T. trichiura* based on sequence homology.

§The haplotype assigned the name TMUE is from *Trichuris muris* which was used as an outgroup for the phylogenetic analysis. The present phylogenetic analysis also included sequences from *Trichuris suis*, though the *T. suis* strains included only had mitochondrial genome sequences available; *T. suis* 18S sequences are not listed here.

**Notes:** S1 File (Tab C) contains SRA accession numbers for each sample sequenced in this study (various combinations of Haplotype I, II, III, V, and VII). The sequence of each haplotype listed in this table is provided in S2 File. An 18S rDNA sequence with GenBank accession number AB699090.1 from Japanese human patients [18] is not listed in this table as it is a combination of HVR-I haplotypes I and II (the sequence contains IUPAC base codes that reflect this).

**Table 3:** Summary of primate-infecting *Trichuris* species HVR-IV haplotype naming scheme.

Hap.	GenBank/SRA Accessions	Species designation	Host/s	Location	Reference
A	PX401252, MF288617.1, MF288619.1 - MF288623.1, MF288632.1, MF288630.1, MF288629.1, MF288626.1, MF288625.1, KX961641.1, KX961640.1, KX961639.1, OQ152555.1 - OQ152569.1, OQ152579.1 - OQ152593.1, AB699090.1, GQ352555.1, GQ352554.1, GQ352551.1, GQ352550.1, GQ352548.1, GQ352547.1, ERR9805831, ERR9805794, ERR9805795, ERR9805811, ERR9805812, ERR9805813, ERR9805815, ERR9805816, ERR9805817, ERR9805819, ERR9805820, ERR9805821, ERR9805822, ERR9805814, ERR9805779, ERR9805780, ERR9805781, ERR9805782, ERR9805783, ERR9805803, ERR9805805, TTRE_003536^	*Trichuris trichiura*	Humans, African vervet (St. Kitts), *Papio hamadryas*	Myanmar, Thailand, Laos, Japan, Denmark (captive *Papio hamadryas*), Uganda, China, Honduras	Present study, Phosuk, Sanpool [19], Yao, Walkush [3], Arizono, Yamada [18], Areekul, Putaporntip [16], Ishizaki, Kawashima [17]
B	PX401253, MF288624.1, MF288618.1, LC596913.1, LC596914.1, GQ352553.1, ERR9805831, ERR9805794, ERR9805795, ERR9805811, ERR9805812, ERR9805815, ERR9805817, ERR9805819, ERR9805814, ERR9805781, ERR9805782	*Trichuris trichiura*	Human, African vervet (St. Kitts), *Papio hamadryas*	Myanmar, Laos, Thailand, Japan, Denmark (ancient latrine), Denmark (captive *Papio hamadryas*), Uganda, China	Present study, Phosuk, Sanpool [19], Ishizaki, Kawashima [17], Areekul, Putaporntip [16]
C	GQ352552.1	*Trichuris trichiura*	Dog†	Thailand, Myanmar, Malaysia	Areekul, Putaporntip [16]
D	GQ352549.1	*Trichuris trichiura*	Human	Thailand	Areekul, Putaporntip [16]
E	AB699092.1	*Trichuris trichiura*	Japanese macaque	Japan	Arizono, Yamada [18]
F	MF288631.1	*Trichuris trichiura*	Human	Myanmar	Phosuk, Sanpool [19]
G	MF288627.1	*Trichuris trichiura*	Human	Thailand	Phosuk, Sanpool [19]
H	DQ118536.1	*Trichuris trichiura*	Unknown	Unknown	Blaxter & Roche (2005) ^§^
I	KY971750.1	*Trichuris* sp.	*Rhinopithecus roxellana* (Golden snub-nosed monkey)	China	Shen et al. (2017) ^§^
J	KY971747.1	*Trichuris* sp.	*Pseudois nayaur* (Bharal)^&^	China	Shen et al. (2017) ^§^
K	ERR9805786, PX401254	*Trichuris trichiura*	Francois’ leaf-monkey	China (captive animal)	Doyle, Soe [14]
L	HF586906.1, ERR9805796, ERR9805797	*Trichuris colobae*	*Colobus guereza kikuyensis*	Spain (captive animal)	Callejon, Nadler [9], Doyle, Soe [14]
TMUE	HF586907.1, PRJEB126‡	*Trichuris muris*	Mouse	Spain	Callejon, Nadler [9]

† This sequence is derived from a non-primate host though it is > 99% identical to other *T. trichiura* sequences in GenBank.

‡ The haplotype assigned the name TMUE is from *Trichuris muris* which was used as an outgroup for the phylogenetic analysis. The present phylogenetic analysis also included sequences from *T. suis*, though the *T. suis* strains included only had mitochondrial genome sequences available so *T. suis* 18S sequences are not listed here.

§ No publication available, only GenBank accession numbers.

& The bharal is not a primate (it is a caprid), but this sequence is 99.6% identical to a sequence derived from a golden snub-nosed monkey isolate (GB: KY971750.1).

^A *Trichuris trichiura* genome available under BioProject PRJEB535.

**Note:** S1 File (Tab C) contains SRA accession numbers for each sample sequenced in this study (all were Haplotype A and/or B). The sequence of each haplotype listed in this table is provided in S2 File.

### References from GenBank

Notably, the genetic distance computation method used here includes routines that accommodate partial genotypes by imputing missing distances for absent markers, allowing genotypes comprising only mitochondrial (Mt) sequences to be included in the same phylogenetic analysis as those with complete (18S and Mt) genotypes (see methods below) [20–22]. Thus, 31 *Trichuris* species Mt genome sequences available in GenBank (GB) were included as reference sequences for phylogenetic analysis, using only the segments corresponding to our Mt locus amplicon. This included four Mt genomes belonging to *Trichuris* from non-human primates (GB: KC461179.1, MW448471.1, KT449824.1, KT449825.1, MW448470.1, MW448472.1), and eight sequences attributed to *Trichuris incognita* (GB: PQ571571.1 to PQ571578.1) [23,24]. The reference dataset also included 10 *T. trichiura* sequences generated from ancient European latrines and soil specimens (GB: KY368768, KY368768, KY368765, KY368772, KY368771, KY368773, KY368770, KY368767, KY368766, KY368774), 6 modern *T. trichiura* isolates derived from humans (GB: GU385218, KT449826, NC_017750.1, ON646012, ON682760, ON711246), and one isolate of unknown host origin (GB: AP017704.1). We also extracted the Mt locus and 18S rDNA gene from a publicly available *T. trichiura* reference genome (BioProject: PRJEB535) for inclusion in this analysis. The reference dataset also included Mt sequences from *Trichuris suis* [GB: GU070737, KT449822, KT449823, NC_017747), and a complete genotype (i.e., including the Mt locus,18S rDNA HVR-I and HVR-IV) extracted from a *Trichuris muris* genome (NCBI BioProject PRJEB126), which served as an outgroup for the phylogeny. The *T. muris* and *T. trichiura* genotypes extracted from the published genome assemblies included all three loci (the Mt locus, HVR-I, and HVR-IV) while the 4 *T. suis* reference sequences and the 31 additional reference sequences described above included only the Mt locus.

### References from the SRA database

Genotypes were extracted from the raw Illumina sequence data described by Doyle, Soe [14], available via the NCBI SRA database. The Mt locus could not be extracted from all genomes sequenced in that study due to a lack of coverage or the presence of highly mixed genotypes that prevented unphasing of the underlying haplotypes. In all, 21 *Trichuris* genotypes were extracted from the genomes described by Doyle, Soe [14]. Briefly, reads were quality trimmed using the bbduk tool from the BBMap toolkit (version 38.73) (Bushnell 2014) with the following parameters: minlen  =  50, qtrim  =  rl, trimq  =  30, ktrim  =  r, k  =  23, mink  =  11, hdist  =  1. Trimmed reads were mapped to GB reference sequences (18S rDNA: AB699092.1, Mt: KT449822.1) trimmed to the regions amplified using the primers in Table 1. Mapping was performed using BWA (default parameters). The fastq reads mapping to each reference sequence were extracted from the resulting SAM files using SAMtools (Li et al. 2009). These fastq files were imported into Geneious Prime (Biomatters LTD, Auckland, New Zealand: https://www.geneious.com) for haplotype assignment as described below.

### Haplotype assignment from Illumina data

The following methods were applied to published fastq reads obtained from the SRA database as described above, in addition to Illumina data generated here. Illumina reads generated from vervet specimens were subjected to the bbduk quality trimming process described above. Next, mapping of reads was performed (against reference sequences AB699092.1 and KT449822.1) using the Geneious Prime “Map to reference” function with the following parameters: maximum gap of 10% per read, maximum gap size of 15 bases, minimum overlap of 25 bases, minimum overlap similarity of 80%, maximum 20% mismatches per read, and a maximum of 4 ambiguities. Other values were set to default. Resultant alignment files were viewed manually in Geneious to assess the quality of mapping. After visual confirmation of mapping accuracy, a consensus sequence was generated for 18S HVR-I, HVR-IV, and the Mt locus for each sample/specimen.

### Establishing haplotype definitions for clustering and minimum data requirements

Barratt’s heuristic was used to compute pairwise genetic distances for phylogenetic tree construction [20,21,25]. This method was selected for inclusion of partial and complete genotypes collectively within the same phylogenetic analysis. Despite the absence of data for some genotypes, this can produce an accurate phylogeny provided that certain conditions are met. Firstly, most (ideally all) specimens should possess some intersecting markers or there will be no basis for comparison, and subsequent imputation of missing values [20]. Next, all specimens in the dataset must meet a rational minimum genotype completeness threshold, as set by the user prior to the analysis [20]. For this purpose, each locus was divided into a set of microhaplotypes as defined in Figs A-C and Tables A-C in S2 File. HVR-I was divided into 8 microhaplotypes, HVR-IV into 4 microhaplotypes, and the Mt locus into 37 microhaplotypes, noting that only 32 of these 37 were utilized for distance computation as described in Fig A in S2 File. As the Mt locus is more diverse, and therefore more informative than the 18S rDNA loci, distance computation was only performed on specimens with data available for at least 60% (i.e., 20) of the 32 Mt microhaplotype regions defined in Fig A in S2 File. The HVR-I and HVR-IV loci were utilized for distance computation if available for a given genotype, though these were not considered an absolute requirement for distance computation in this study.

### Haplotype data sheet generation and genetic distance computation

Prior to distance computation, a haplotype data sheet (HDS) was generated for all samples using the microhaplotypes identified for each sample as input. The HDS format is the required input format for genetic distance computation using Barratt’s heuristic. For a description of the HDS format see: https://github.com/Joel-Barratt/Eukaryotyping. Microhaplotype sequences (S2 File) were extracted from reference *Trichuris* sequences and the consensus sequences generated from the NGS data generated here. The microhaplotype composition of each *Trichuris* specimen is provided in the HDS within S1 File, Tab A. Genetic distance computation was performed using the scripts and instructions available here: https://github.com/Joel-Barratt/Eukaryotyping. The resultant pairwise genetic distance matrix was used to generate a neighbor-joining tree [26] via the ‘nj’ function available in the ‘ape’ R package. The ‘root’ function in the ‘ape’ R package was used to root the tree at the branch occupied by *T. muris*. The ‘ggtree’ R package was used to visualize and annotate the resultant trees. Images of relevant hosts were obtained from PhyloPic (http://phylopic.org) or prepared in-house for annotation of dendrograms. Maps were generated in R using ggplot. Images were rendered using the GNU Image manipulation program (https://www.gimp.org).

### Trichuris ITS1 and ITS2 analysis

#### Haplotype detection.

Paired Illumina reads were imported into Geneious Prime and subjected to quality trimming and filtering using bbduk (custom bbduk parameters: ktrimright = t k = 27 hdist = 1 edist = 0 ref = adapters.fa qtrim = rl trimq = 20 minlength = 100 ordered = t qin = 33). Using the Geneious mapper (default “Medium sensitivity/Fast” setting parameters), trimmed reads were aligned to *Trichuris* reference sequence MH390370.1. Reads mapping between the priming sites for the ITS1 and ITS2 primers (Table 1) were extracted manually in the Geneious interface, retaining only sequence falling between the priming sites for ITS1 and ITS2 (reads spanning the priming sites were trimmed to retain only the part of the read falling between the forward and reverse primers). Following extraction, reads less than 100 bases in length were discarded. Next, amplicon sequence variants (ASVs) were identified using an R implementation DADA2 [27]. Briefly, where possible, paired reads were merged using the “mergePairs” function. Chimeric amplicons were detected and removed using the “removeBimeraDenovo” function. Non-chimeric ASVs detected at a frequency of less than 20 were discarded. Remaining ASVs were exported to a fasta file. As the amplicons are relatively large (296 and 648 bases excluding the primer sequence for ITS1 and ITS2 respectively), the set of ASVs detected in each worm was subjected to a simple assembly using the Geneious de novo assembler, where ASVs from individual worms overlapping by at least 30 identical bases were merged into a single contig. The resulting contigs were aligned using the Geneious alignment tool (default parameters), and the aligned sequences were exported in fasta format for subsequent import into DNASP6 for conversion of the files to nexus format [28]. The nexus files were manually edited to incorporate trait data (i.e., geographic location). The edited nexus files were then imported into PopART for construction of Integer Neighbor Joining Networks [29].

## Results

### Morphological examination of adult Trichuris nematodes

**Fig 1:**
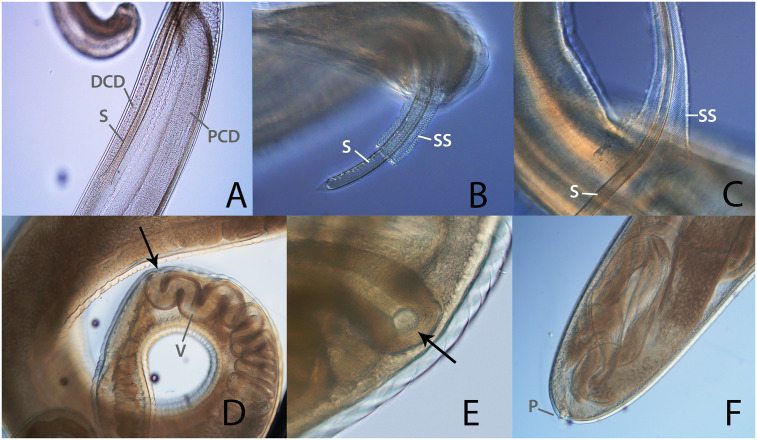
Representative images of features of male (top row) and female (bottom row) *Trichuris trichiura* in this study under differential interference contrast (DIC) microscopy. A) Proximal cloacal duct (PCD) and distal cloacal duct (DCD) containing spicule (S); B) Everted spicule (S) and spinous sheath (SS) with a straight shape; C) Everted spicule and spinous sheath with a flared shape; D) Midsection of female body showing long, convoluted vagina (V) and non-protruding vulva (arrow); E) Delicate spinous ornamentation apparent in the vulvar region of some individuals (arrow); F) Posterior end of female showing two terminal papillae (P; only one visible in focal plane). Size ranges are provided in S1 File.

All worms were found to be consistent with previous reports of *T. trichiura* from humans and non-human primates (Fig 1). Males possessed typical features of *T. trichiura* such as a spicular sheath covered in dense spines, broad proximal cloacal tube, a single spicule with a central clear zone, and one pair of peri-cloacal papillae. Observed minor variations in the shape of the end of the spicular sheath when everted (straight vs. flared) and course of the proximal cloacal tube (straight vs. slightly sigmoid) were seen in a few individuals. Males averaged 18.6 mm (range: 14.4 – 26.7 mm) in total length with an average anterior/posterior length ratio of 1.54 (range: 1.01 – 2.38). Similarly, all females possessed expected features of *T. trichiura* (convoluted vagina, non-protrusive vulva, subterminal anus with two terminal papillae). Minute spinous ornamentation of the vaginal opening was visible on a few female specimens in which the vagina was slightly everted, although this trait was difficult to confirm in others. Females averaged 18.9 mm (range: 13.9 – 28.5 mm) in total length with an average anterior/posterior length ratio of 2.26 (range: 1.41 – 2.99). Eggs in utero ranged from 49—58 x 22–28 µm. Additional morphometric data are given in S1 File.

### Trichuris 18S rDNA and Mt genotypes

A genotype was generated for 32 of 35 adult *Trichuris* from St. Kitts vervets, and from 17 of the 34 vervet fecal samples. Extraction of DNA from three worms failed and did not yield any sequence data, and 17 of the fecal samples did not generate PCR amplicons. Although microscopy indicated the presence of *Trichuris* eggs in all 34 of the extracted fecal samples, there are several reasons why they may not have been detected via PCR. For instance, few eggs were observed in some samples and fecal samples are notoriously complex, often containing PCR inhibitors that may have prevented amplification. For nine of the 17 fecal samples with successful sequencing two genotypes were identified resulting in 26 total genotypes derived from the fecal samples. For 24 of these 26 genotypes, a definitive set of HVR-I and HVR-IV haplotypes could not be determined; the sequence data supported a mixture of haplotypes that could not be accurately unphased. For these specimens, clustering was performed solely based on their Mt sequence which could be more easily unphased (see results below). Among the 116 genotypes analyzed, a complete 18S rDNA genotype was generated (or available) for 54. Of these the “I+II & A+B” genotype was the most common (Table 4). All genotypes are defined in S1 File, Tab C.

**Table 4:** Counts of 18S rDNA genotypes detected.

Genotype counts for *Trichuris trichiura* by clade (Clade 2b & Clade 2c)^†^			
Genotype (HVR-I & HVR-IV)	Clade 2b	Clade 2c	Total (both clades)
I + II & A + B	3	25	28
I + II & A	1	9	10
II+XXIII & A	1	0	1
II & A	2	1	3
II+XXII & A + B	0	1	1
II + VII & A + B	0	2	2
II & A + B	0	3	3
II & B	0	2	2
II + III & A	0	1	1
II + V & A + B	0	1	1
II + XIX & A + B	0	1	1
II + XX & A + B	0	1	1
Total	7	47	54

† Clades 2b and 2c were first defined by Rivero, Cutillas [5]. All genotypes sequenced in this study from St. Kitts vervets belong to clade 2c; worms assigned to clade 2b were identified solely from published reference data.

### Phylogenetic analysis of the 18S rDNA and Mt locus

**Fig 2:**
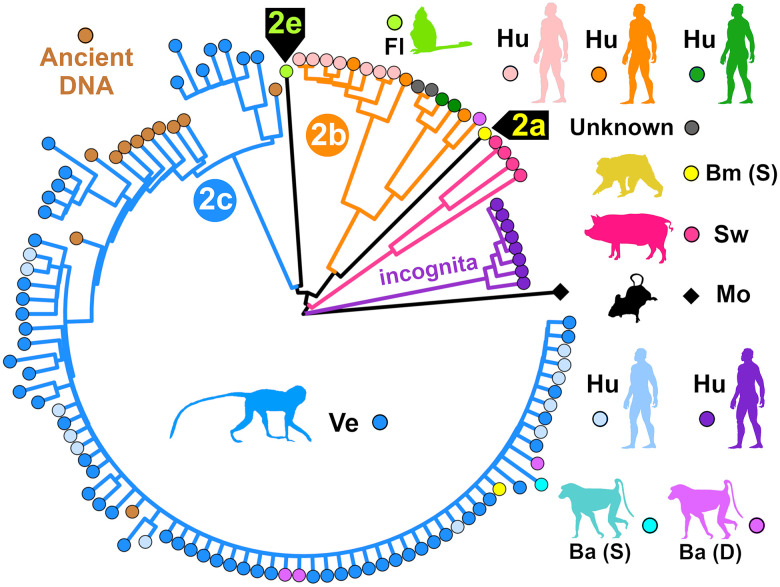
Neighbor-Joining tree of *Trichuris* genotypes.

A genetic distance matrix (S1 File, Tab B) was computed from the HDS (S1 File, Tab A), and this matrix was used to construct a neighbor joining tree (Fig 2). This tree supported that two dominant clades of *T. trichiura* (clades 2b and 2c, as previously described [5]) infect humans in addition to other non-human primates, with two reference specimens (one from a Francois’ leaf-monkey and another from a Barbary macaque) representing two additional populations (Fig 2, clades 2a and 2e) that were described previously [5]. *T. trichiura* clade 2d [5,30] was not represented in the present phylogeny, as the only evidence for the existence of these groups was derived from an amplicon of the Mt *rrnL* locus. The Mt locus sequenced in the present study does partially capture the *rrnL* gene, though reference sequences for clade 2d did not sufficiently overlap with the present Mt locus. Vervets from St. Kitts were infected with *T. trichiura* belonging to clade 2c, which was reported previously in humans from Uganda, in African primates in European zoos, and among *T. trichiura* types observed in ancient European latrines. The present phylogeny also suggests that *Trichuris incognita* occupies a phylogenetic position that is basal to the clades occupied by *T. suis* and *T. trichiura* (Fig 2).

### Haplotype analysis of ITS1 and ITS2

**Fig 3:**
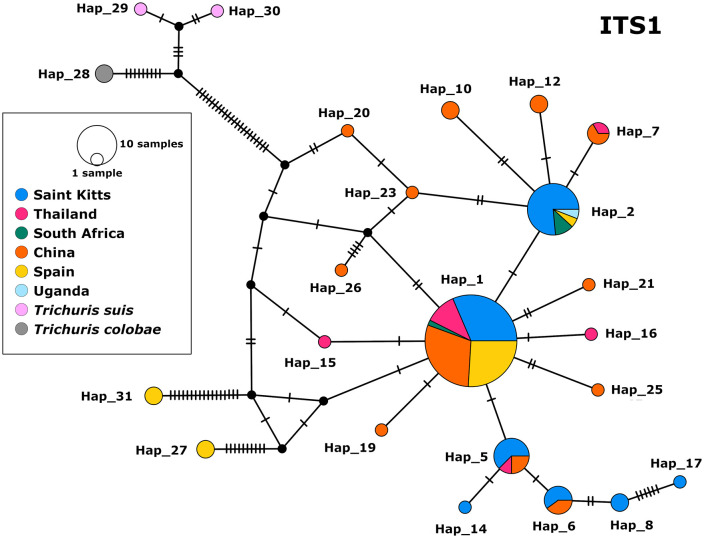
Integer Neighbor-Joining Network of *Trichuris* ITS1 haplotypes.

**Fig 4:**
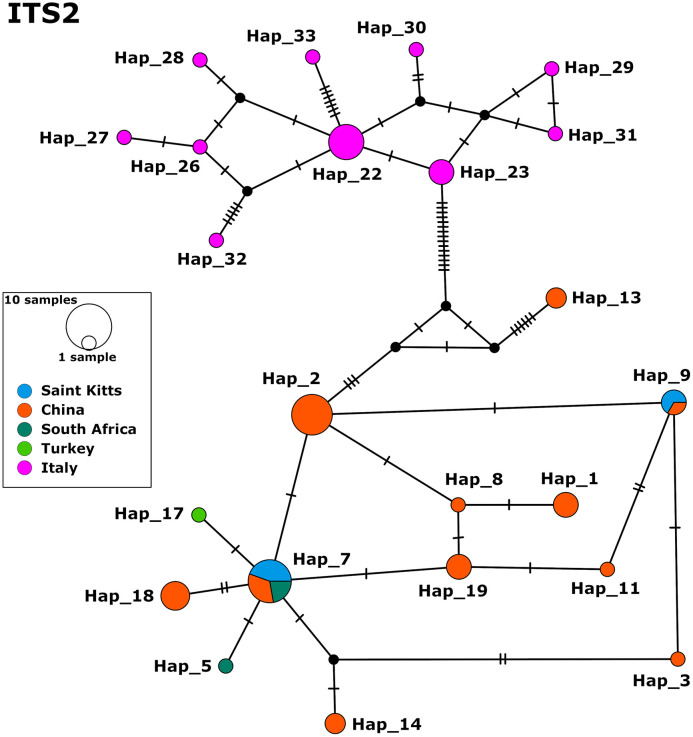
Interger Neighbor-Joining Network of *Trichuris* ITS2 haplotypes.

Seven ITS1 haplotypes (Fig 3) and two ITS2 haplotypes (Fig 4) were detected across all *T. trichiura* adult worms isolated from St Kitts vervets. ITS sequences from fecal samples could not be analyzed due to high intra-sample diversity. Individual *T. trichura* adults possessed up to three well-supported ITS1 haplotypes within their genomes. Three of the 7 ITS1 haplotypes from St Kitts vervets were presumed to be unique to this *T. trichiura* population as identical sequences were not found in GenBank. Four of the 7 ITS1 haplotypes detected in St Kitts vervets had been reported previously from humans and/or non-human primates in other locations. Regarding ITS2, due to the length of the ITS2 amplicon (Table 1) and the nature of the short-read sequence data, ITS2 sequences identified from several worms did not span the entire length of the ITS2 amplicon and were therefore excluded from the haplotype analysis (i.e., regions containing informative SNPs were missing from the ends of some sequences). For ITS1, many sequences generated were only slightly shorter than the expected amplicon, so all ITS1 haplotypes were trimmed to the same analogous region to facilitate inclusion of more sequences in the haplotype analysis. All ITS haplotype sequences (post trimming) are provided in S2 File.

This tree was constructed using the Neighbor-Joining method from a genetic distance matrix computed using Barratts heuristic [26]. The tree includes 116 *Trichuris* genotypes, including 58 from *T. trichiura* infecting St. Kitts vervets. Branches are colored according to their population membership (*T. trichiura* subclade 2b in orange, *T. trichiura* subclade 2c in blue, *Trichuris suis* in pink, *Trichuris incognita* in purple and other *Trichuris* types including *T. muris* in black). Colored circles on branch tips indicate the host type for a given *Trichuris* specimen, in addition to the geographic origin of the specimen. Hu = Human from either Uganda (light blue tip circle), China (light pink tip circle), Korea (orange tip circle), or Honduras (dark green tip circle). Ba (S) = Baboon from a Spanish zoo, Ba (D) = Baboon from a Danish zoo, BM (S) = Barbary macaque from a Spanish zoo, Fl = Francois’ leaf-monkey from China, Sw = *T. suis* from Chinese and Ugandan swine, Ancient DNA = Ancient DNA from soil/ancient latrines in Denmark and The Netherlands, Unknown = *Trichuris* DNA sequences of unknown source, Ve = vervets from St. Kitts. A black diamond indicates a genotype of *T. muris* obtained from a mouse (Mo) which serves as the outgroup for this tree. A version of this tree with isolate names shown on the branch tips is provided in S3 File. Four of the five major *T. trichiura* subclades described by Rivero, Cutillas [5] are shown on this tree; subclade 2d is not represented as only sequence data for the *rrnL* genes are available for this clade, with insufficient overlap with our mitochondrial amplicon for inclusion in this analysis.

An Integer Neighbor-Joining Network was constructed from *Trichuris* ITS1 sequences using a reticulation tolerance of 1. Hatch marks on edges each represent a single nucleotide polymorphism. Black nodes represent inferred haplotypes that were not detected in this study or were not observed in the NCBI nucleotide database. Nodes of all other colors represent haplotypes that were detected in the St Kitts *Trichuris* population (darker blue) or those that were publicly available in the NCBI nucleotide database (all colors excluding black and the darker blue). The size of the nodes is scaled proportionally to the frequency that a given haplotype was observed in this study and/or in the NCBI nucleotide database. The key shows node sizes for a haplotype observed at a frequency of 1 and 10 for comparison. Nodes were represented by a pie chart when a given haplotype was detected in *Trichuris* isolated from multiple locations. In these cases, the size of the pie segments represents the frequency at which a haplotype was observed at a given location as reflected in the key. Indels are ignored for the purpose of this analysis. This image was rendered using the GNU Image Manipulation Suite (GIMP).

An Integer Neighbor-Joining Network was constructed from *Trichuris* ITS2 sequences using a reticulation tolerance of 1. Hatch marks on edges each represent a single nucleotide polymorphism. Black nodes represent inferred haplotypes that were not detected in this study or were not observed in the NCBI nucleotide database. Nodes of all other colors represent haplotypes that were detected in the St Kitts *Trichuris* population (blue) or those that were publicly available in the NCBI nucleotide database (all colors excluding black and blue). The size of the nodes is scaled proportionally to the frequency that a given haplotype was observed in this study and/or in the NCBI nucleotide database. The key shows node sizes for a haplotype observed at a frequency of 1 and 10 for comparison. Nodes were represented by a pie chart when a given haplotype was detected in *Trichuris* isolated from multiple locations. In these cases, the size of the pie segments represents the frequency at which a haplotype was observed at a given location as reflected in the key. Indels are ignored for the purpose of this analysis. This image was rendered using the GNU Image Manipulation Suite (GIMP).

## Discussion

This study confirms that the *Trichuris* species infecting the St. Kitts vervets is *T. trichiura*, as was reported previously [3,7]. Genetic characterization found that the St. Kitts vervet *T. trichiura* belonged to a variety (clade 2c) that has been reported from humans in Uganda [31], and from ancient European soil samples and latrines [14,32]. This corroborates previous reports that *T. trichiura* from NHPs can infect humans [15], and supports that varieties observed in St. Kitts vervets may be sources of human infection on the island. Genotyping of *T. trichiura* from humans inhabiting St. Kitts is necessary for further exploration of this hypothesis. Based on the present results and those previously described [5], separation of clades 2a-2e was driven by differences within the Mt locus supporting distinct maternal lineages, as specific 18S rDNA genotypes did not cluster consistently to either of the 2b or 2c clades (Table 4). Both of these *T. trichiura* clades are widespread geographically, with 2c being seemingly more common in Europe and Africa while 2b is more common in Asia, though also includes two specimens from Honduras derived from previously published data [14].

Based on our analysis, the main *T. trichiura* subclades (2a-2e) are driven by the existence of 5 major maternal (mitochondrial) lineages, which agrees with prior studies [4,5]. This is supported by the fact that the 18S rDNA haplotypes are inherited independently of the population structure as the various 18S rDNA haplotypes do not track with one clade or the other, and heterozygosity at these loci was common within individual worms. These results support that gene flow has recently occurred (or continues to occur) between clades 2b and 2c or may hint at incomplete lineage sorting of the nuclear 18S rDNA genes. This situation differs compared to what has been observed for other nematodes, such as *Strongyloides*, for which the 18S rDNA HVRs – particularly HVR-IV – are useful for delineating major lineages [22,33].

We remain uncertain of the significance of the various clades. Often, the emergence of distinct genetic clades within a species is driven by environmental pressure (e.g., adaptation to new hosts), and/or vicariance (geographic separation over long periods). Our analysis supports no clear relationship between host and membership to a specific *T. trichiura* clade. Similarly, the clades are quite cosmopolitan as is the distribution of the various ITS1 and ITS2 haplotypes, so it is difficult to comment on whether vicariance was once at play but is no longer observable due to dispersal of the parasite via human activities.

This study also sought to sequence ITS1 and ITS2 since these loci have been used extensively for conventional Sanger sequencing based genotyping studies of *Trichuris* [4,5,34–36]. As reported previously, we observed multiple paralogous copies of ITS1 within individual worms [37]. The DNA extracted from vervet stool samples was particularly problematic as it originated from a population of *Trichuris* eggs and even other nematode species (versus material from a single worm) so stool samples were excluded from the ITS haplotype analysis. Ultimately, analysis of ITS1 and ITS2 data from the adult worms largely supports the 18S rDNA and Mt DNA results (Fig 2), suggesting that *T. trichiura* from St Kitts vervets belong to a globally dispersed population of *T. trichiura* infecting humans and various non-human primates.

Our morphometric measurements of worms assigned to clade 2c in this study were proportionally analogous to other reports of *T. trichiura* from humans and non-human primates, albeit the overall lengths were generally slightly smaller than measurements reported in recent comparative studies of a similar design [4,5,12]. Other male- and female-specific measurements (S1 File, Tabs E and F) fell within the typical ranges reported for *T. trichiura.* The apparent variations in qualitative characteristics observed in some individuals, such as the shape of the end of the spicule sheath, straight vs. slightly sigmoid proximal cloacal duct, and presence of small ornamentation on the vulva, similarly did not appear to correspond to clade membership. Variability among many different morphologic characteristics has been reported in the *T. trichiura* literature and is usually attributed to normal intraspecific heterogeneity, host-induced adaptation, or impacts of specimen fixation. Overall, these results are unsurprising; historical and contemporary authors alike have consistently acknowledged the difficulty of morphologic differentiation among closely related *Trichuris* populations due to both intrinsic and extrinsic factors [4,5,8,11,12,38,39].

An inherent challenge of studies involving integrated molecular and morphologic characterization of individual worms is balancing the need for high quality preservation of both parasite morphology and DNA. Fixation in formalin-based solutions is generally regarded as the best choice for morphologic studies; however, this has a substantial negative impact on DNA quality. The common alternative of ethanol overcomes this problem but often induces shrinkage and damage of finer morphologic features of small nematodes. Morphometric assessment in this study was constrained by physical damage to some specimens preventing evaluation of certain features as well as distortion from 90% ethanol fixation. Future studies focusing on *Trichuris* morphometrics in this population may consider either prioritizing fixation methods ideal for morphology (including preparation for scanning electron microscopy), or employing solutions described as dual-purpose such as DESS [40].

Failure to amplify the target *Trichuris* markers from some microscopy-positive vervet fecal samples is a noteworthy limitation of the present study. For the most part, we attribute this issue to the fact that some samples contained very few *Trichuris* eggs (i.e., below the minimum limit required for successful amplification and sequencing), and perhaps due to the presence of PCR inhibitors in the final DNA extracts. However, we cannot exclude the possibility that some vervets were infected with *Trichuris* strains (or species) for which the primers were not a sufficient match at their intended annealing sites. Consequently, the design of additional primer pairs that capture a broader diversity of *Trichuris* might address this problem should similar studies be performed on this population of vervets in the future. Additionally, an internal control assay may be of use in future studies, which would give definitive indications of the presence or absence of PCR inhibitors.

In summary, this study confirms that vervet monkeys on the island of St. Kitts are infected with *T. trichiura* belonging to clade 2c. Worms belonging to this clade have been observed in humans from Africa, from multiple species of non-human primates kept in European zoos, and in ancient European latrines. This suggests that clade 2c comprises worms widely distributed geographically and are primate generalists, possessing zoonotic potential. Genotyping of *T. trichiura* from humans on the island of St. Kitts in the future could help confirm the role of St. Kitts vervets as reservoirs of human infection, as suggested by the present work.

### Highlights

- *Trichuris trichiura* infects free-roaming vervets on the Caribbean Island of Saint Kitts.
- Eggs and adults of T. trichiura infecting St. Kitts vervets were genotyped to assess the zoonotic potential St. Kitts vervets are infected with *T. trichiura* genotypes found in humans.
- St. Kitts vervets may represent reservoirs of human *T. trichiura* infection.

## Supporting information

S1 File Excel spreadsheet containing specimen metadata and other information. This Excel spreadsheet includes the GenBank nucleotide and SRA database accession numbers of the data analyzed here, the genotype of each specimen, and the pairwise distance matrices generated using our ML procedure.
(XLSX)

S2 File Trichuris haplotypes and supplemental methods. A word document containing all haplotype sequences and other relevant information.
(DOCX)

S3 File Phylogenetic tree shown in Fig 1 with tip labels. A phylogenetic tree identical to the tree shown in Fig 1, though with specimen labels provided on branch tips.
(PDF)

## References

[1] Else KJ, Keiser J, Holland CV, Grencis RK, Sattelle DB, Fujiwara RT, et al. Whipworm and roundworm infections. Nat Rev Dis Primers. 2020;6(1):44. doi: 10.1038/s41572-020-0171-3 32467581

[2] Stephenson LS, Holland CV, Cooper ES. The public health significance of Trichuris trichiura. Parasitology. 2000;121 Suppl:S73–95. doi: 10.1017/s0031182000006867 11386693

[3] Yao C, Walkush J, Shim D, Cruz K, Ketzis J. Molecular species identification of Trichuris trichiura in African green monkey on St. Kitts, West Indies. Vet Parasitol Reg Stud Reports. 2018;11:22–6. doi: 10.1016/j.vprsr.2017.11.004 31014613

[4] Rivero J, García-Sánchez ÁM, Zurita A, Cutillas C, Callejón R. Trichuris trichiura isolated from Macaca sylvanus: morphological, biometrical, and molecular study. BMC Vet Res. 2020;16(1):445. doi: 10.1186/s12917-020-02661-4 33203430 PMC7672873

[5] Rivero J, Cutillas C, Callejón R. Trichuris trichiura (Linnaeus, 1771) From Human and Non-human Primates: Morphology, Biometry, Host Specificity, Molecular Characterization, and Phylogeny. Front Vet Sci. 2021;7:626120. doi: 10.3389/fvets.2020.626120 33681315 PMC7934208

[6] McGuire MT. The history of the St. Kitts vervet. Caribbean Quarterly. 2017;20(2):37–52.

[7] Gallagher C, Beierschmitt A, Cruz K, Choo J, Ketzis J. Should monkeys wash their hands and feet: A pilot-study on sources of zoonotic parasite exposure. One Health. 2019;7:100088. doi: 10.1016/j.onehlt.2019.100088 30963089 PMC6434333

[8] Ooi HK, Tenora F, Itoh K, Kamiya M. Comparative study of Trichuris trichiura from non-human primates and from man, and their difference with T. suis. J Vet Med Sci. 1993;55(3):363–6. doi: 10.1292/jvms.55.363 8357906

[9] Callejón R, Nadler S, De Rojas M, Zurita A, Petrášová J, Cutillas C. Molecular characterization and phylogeny of whipworm nematodes inferred from DNA sequences of cox1 mtDNA and 18S rDNA. Parasitol Res. 2013;112(11):3933–49. doi: 10.1007/s00436-013-3584-z 24018707

[10] Richins T, Sapp SGH, Ketzis JK, Willingham AL, Mukaratirwa S, Qvarnstrom Y, et al. Genetic characterization of Strongyloides fuelleborni infecting free-roaming African vervets (Chlorocebus aethiops sabaeus) on the Caribbean island of St. Kitts. Int J Parasitol Parasites Wildl. 2023;20:153–61. doi: 10.1016/j.ijppaw.2023.02.003 36860205 PMC9969202

[11] Cutillas C, Callejón R, de Rojas M, Tewes B, Ubeda JM, Ariza C, et al. Trichuris suis and Trichuris trichiura are different nematode species. Acta Trop. 2009;111(3):299–307. doi: 10.1016/j.actatropica.2009.05.011 19467214

[12] García-Sánchez AM, Rivero J, Callejón R, Zurita A, Reguera-Gomez M, Valero MA, et al. Differentiation of Trichuris species using a morphometric approach. Int J Parasitol Parasites Wildl. 2019;9:218–23. doi: 10.1016/j.ijppaw.2019.05.012 31194117 PMC6551462

[13] Cutillas C, de Rojas M, Zurita A, Oliveros R, Callejón R. Trichuris colobae n. sp. (Nematoda: Trichuridae), a new species of Trichuris from Colobus guereza kikuyensis. Parasitol Res. 2014;113(7):2725–32. doi: 10.1007/s00436-014-3933-6 24853537

[14] Doyle SR, Søe MJ, Nejsum P, Betson M, Cooper PJ, Peng L, et al. Population genomics of ancient and modern Trichuris trichiura. Nat Commun. 2022;13(1):3888. doi: 10.1038/s41467-022-31487-x 35794092 PMC9259628

[15] Dolezalova J, Obornik M, Hajduskova E, Jirku M, Petrzelkova KJ, Bolechova P, et al. How many species of whipworms do we share? Whipworms from man and other primates form two phylogenetic lineages. Folia Parasitol. 2015;62:2015.063. doi: 10.14411/fp.2015.063 26668135

[16] Areekul P, Putaporntip C, Pattanawong U, Sitthicharoenchai P, Jongwutiwes S. Trichuris vulpis and T. trichiura infections among schoolchildren of a rural community in northwestern Thailand: the possible role of dogs in disease transmission. Asian Biomedicine. 2010;4(1):49–60. doi: 10.2478/abm-2010-0006

[17] Ishizaki Y, Kawashima K, Gunji N, Onizawa M, Hikichi T, Hasegawa M, et al. Trichuris trichiura Incidentally Detected by Colonoscopy and Identified by a Genetic Analysis. Intern Med. 2022;61(6):821–5. doi: 10.2169/internalmedicine.8012-21 34471029 PMC8987263

[18] Arizono N, Yamada M, Tegoshi T, Onishi K. Molecular identification of Oesophagostomum and Trichuris eggs isolated from wild Japanese macaques. Korean J Parasitol. 2012;50(3):253–7. doi: 10.3347/kjp.2012.50.3.253 22949756 PMC3428574

[19] Phosuk I, Sanpool O, Thanchomnang T, Sadaow L, Rodpai R, Anamnart W, et al. Molecular Identification of Trichuris suis and Trichuris trichiura Eggs in Human Populations from Thailand, Lao PDR, and Myanmar. Am J Trop Med Hyg. 2018;98(1):39–44. doi: 10.4269/ajtmh.17-0651 29165218 PMC5928743

[20] Jacobson D, Zheng Y, Plucinski MM, Qvarnstrom Y, Barratt JLN. Evaluation of various distance computation methods for construction of haplotype-based phylogenies from large MLST datasets. Mol Phylogenet Evol. 2022;177:107608. doi: 10.1016/j.ympev.2022.107608 35963590 PMC10127246

[21] Nascimento FS, Barratt J, Houghton K, Plucinski M, Kelley J, Casillas S, et al. Evaluation of an ensemble-based distance statistic for clustering MLST datasets using epidemiologically defined clusters of cyclosporiasis. Epidemiol Infect. 2020;148:e172. doi: 10.1017/S0950268820001697 32741426 PMC7439293

[22] Barratt JLN, Sapp SGH. Machine learning-based analyses support the existence of species complexes for Strongyloides fuelleborni and Strongyloides stercoralis. Parasitology. 2020;147(11):1184–95. doi: 10.1017/S0031182020000979 32539880 PMC7443747

[23] Venkatesan A, Chen R, Bär M, Schneeberger PHH, Reimer B, Hürlimann E, et al. Trichuriasis in Human Patients from Côte d’Ivoire Caused by Novel Trichuris incognita Species with Low Sensitivity to Albendazole/Ivermectin Combination Treatment. Emerg Infect Dis. 2025;31(1):104–14. doi: 10.3201/eid3101.240995 39714288 PMC11682790

[24] Bär MA, Kouamé NA, Touré S, Coulibaly JT, Schneeberger PHH, Keiser J. Characterisation of Trichuris incognita n sp in Côte d’Ivoire: a morphological, genomic, and genome-wide association with drug sensitivity study. Lancet Microbe. 2026;7(2):101264. doi: 10.1016/j.lanmic.2025.101264 41655578

[25] Barratt JLN, Park S, Nascimento FS, Hofstetter J, Plucinski M, Casillas S, et al. Genotyping genetically heterogeneous Cyclospora cayetanensis infections to complement epidemiological case linkage. Parasitology. 2019;146(10):1275–83. doi: 10.1017/S0031182019000581 31148531 PMC6699905

[26] Saitou N, Nei M. The neighbor-joining method: a new method for reconstructing phylogenetic trees. Mol Biol Evol. 1987;4(4):406–25. doi: 10.1093/oxfordjournals.molbev.a040454 3447015

[27] Callahan BJ, McMurdie PJ, Rosen MJ, Han AW, Johnson AJA, Holmes SP. DADA2: High-resolution sample inference from Illumina amplicon data. Nat Methods. 2016;13(7):581–3. doi: 10.1038/nmeth.3869 27214047 PMC4927377

[28] Rozas J, Ferrer-Mata A, Sánchez-DelBarrio JC, Guirao-Rico S, Librado P, Ramos-Onsins SE, et al. DnaSP 6: DNA Sequence Polymorphism Analysis of Large Data Sets. Mol Biol Evol. 2017;34(12):3299–302. doi: 10.1093/molbev/msx248 29029172

[29] Leigh JW, Bryant D. popart: full‐feature software for haplotype network construction. Methods Ecol Evol. 2015;6(9):1110–6. doi: 10.1111/2041-210x.12410

[30] Cavallero S, De Liberato C, Friedrich KG, Di Cave D, Masella V, D’Amelio S, et al. Genetic heterogeneity and phylogeny of Trichuris spp. from captive non-human primates based on ribosomal DNA sequence data. Infect Genet Evol. 2015;34:450–6. doi: 10.1016/j.meegid.2015.06.009 26066463

[31] Nissen S, Al-Jubury A, Hansen TVA, Olsen A, Christensen H, Thamsborg SM, et al. Genetic analysis of Trichuris suis and Trichuris trichiura recovered from humans and pigs in a sympatric setting in Uganda. Vet Parasitol. 2012;188(1–2):68–77. doi: 10.1016/j.vetpar.2012.03.004 22494938

[32] Søe MJ, Nejsum P, Seersholm FV, Fredensborg BL, Habraken R, Haase K, et al. Ancient DNA from latrines in Northern Europe and the Middle East (500 BC-1700 AD) reveals past parasites and diet. PLoS One. 2018;13(4):e0195481. doi: 10.1371/journal.pone.0195481 29694397 PMC5918799

[33] Hasegawa H, Hayashida S, Ikeda Y, Sato H. Hyper-variable regions in 18S rDNA of Strongyloides spp. as markers for species-specific diagnosis. Parasitol Res. 2009;104(4):869–74. doi: 10.1007/s00436-008-1269-9 19050926

[34] Hong JH, Seo M, Oh CS, Shin DH. Genetic Analysis of Small-Subunit Ribosomal RNA, Internal Transcribed Spacer 2, and ATP Synthase Subunit 8 of Trichuris trichiura Ancient DNA Retrieved from the 15th to 18th Century Joseon Dynasty Mummies’ Coprolites from Korea. J Parasitol. 2019;105(4):539–45. doi: 10.1645/19-31 31310584

[35] Liu GH, Zhou W, Nisbet AJ, Xu MJ, Zhou DH, Zhao GH, et al. Characterization of Trichuris trichiura from humans and T. suis from pigs in China using internal transcribed spacers of nuclear ribosomal DNA. J Helminthol. 2014;88(1):64–8. doi: 10.1017/S0022149X12000740 23113971

[36] Liu G-H, Gasser RB, Nejsum P, Wang Y, Chen Q, Song H-Q, et al. Mitochondrial and nuclear ribosomal DNA evidence supports the existence of a new Trichuris species in the endangered françois’ leaf-monkey. PLoS One. 2013;8(6):e66249. doi: 10.1371/journal.pone.0066249 23840431 PMC3688784

[37] Mejías-Alpízar MJ, Porras-Silesky C, Rodríguez EJ, Quesada J, Alfaro-Segura MP, Robleto-Quesada J, et al. Mitochondrial and ribosomal markers in the identification of nematodes of clinical and veterinary importance. Parasit Vectors. 2024;17(1):77. doi: 10.1186/s13071-023-06113-4 38378676 PMC10880205

[38] Špakulová M. Discriminant analysis as a method for the numerical evaluation of taxonomic characters in male trichurid nematodes. Syst Parasitol. 1994;29(2):113–9. doi: 10.1007/bf00009807

[39] Chandler AC. Specific characters in the genus Trichuris, with a description of a new species, Trichuris tenuis, from a camel. J Parasitol. 1930;16(4).

[40] Yoder M, De Ley IT, Wm King I, Mundo-Ocampo M, Mann J, Blaxter M, et al. DESS: a versatile solution for preserving morphology and extractable DNA of nematodes. Nematol. 2006;8(3):367–76. doi: 10.1163/156854106778493448

