# Supplementary material for: Molecular and morphologic characterization of Trichuris trichiura infecting free-roaming African vervets (Chlorocebus aethiops sabaeus) on the Caribbean Island of St. Kitts
Source: PLoS Negl Trop Dis. 2026 Jul 16;20(7):e0014539. doi: 10.1371/journal.pntd.0014539 (PMC13405091; doi:10.1371/journal.pntd.0014539)
Supplement: S2 File — A word document containing all haplotype sequences and other relevant information. (DOCX) [file pntd.0014539.s002.docx]

# Supplementary File S2 to accompany the study:

Molecular and morphologic characterization of *Trichuris trichiura* infecting free-roaming African vervets (*Chlorocebus aethiops sabaeus*) on the Caribbean Island of St. Kitts

Travis Richins^1^*, Sarah G. H. Sapp^1^, Jennifer K. Ketzis^2^, Arve Lee Willingham^3^, Samson Mukaratirwa^2^, Yvonne Qvarnstrom^1^, Joel L. N. Barratt^1,4^*

^1^ Centers for Disease Control and Prevention, Division of Parasitic Diseases and Malaria, Laboratory Science and Diagnostic Branch

^2^Biomedical Sciences, One Health Center for Zoonoses & Tropical Veterinary Medicine, Ross University School of Veterinary Medicine, Saint Kitts

^3^Department of Veterinary Medicine, College of Agriculture & Veterinary Medicine, United Arab Emirates University, United Arab Emirates

^4^Emory School of Medicine, Department of Pathology and Laboratory Medicine, Emory University, Atlanta, Georgia, USA

*Corresponding authors: T Richins and JLN Barratt

**ORCID ID of T Richins:** 0000-0002-0957-8058

**Email (T. Richins):** ofo9@cdc.gov

**ORCID ID of JLN Barratt:** 0000-0001-8711-2408

**Email (J. L. N. Barratt):** joel.barratt@emory.edu

## Defining microhaplotypes for the tRNA val amplicon

Relative to reference sequence ON646012.1 the forward and the reverse primers produce an amplicon extending from bases 10543 to 11163; a total of 621 bases. Microhaplotypes of approximately 15 bases were defined along this amplicon as shown in the schematic below (Figure A). Table A below provides the sequence of all unique tRNA val microhaplotypes identified across all samples clustered in the present study. Note that haplotypes at regions G1, H3, I1, I2 and J2 are excluded from Table A. The haplotype data sheet in File S1, Tab A indicates the specific haplotype found at each region (the regions defined in Figure A) for every sample.

### Figure A. Schematic defining the microhaplotypes of the tRNA val amplicon

The gray bar represents the amplicon from bases 1 through 621, or bases 10543 to 11163 relative to the reference sequence (GenBank accession: ON646012.1) which is a complete mitochondrial genome of *Trichuris trichiura*. The priming sites (green) and the various microhaplotype regions (yellow or pink) are indicated as different colored bars. Microhaplotypes shown in pink are those that were excluded from our analysis due to the presence of insertions and deletions at these locations among the *Trichuris* strains and species being compared, resulting in a poor alignment at these sites. The yellow bars indicate microhaplotype locations utilized for clustering, and these are represented in the haplotype data sheet (Supplementary File S1, Tab A) while those shown in pink are not.


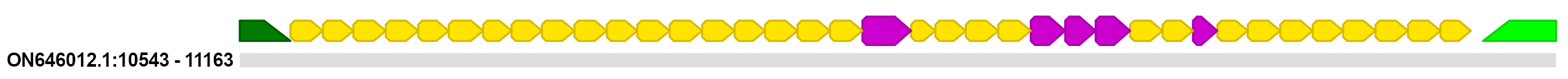


|  | **Base locations** | |  |  |  | **Base locations** | |  |
| --- | --- | --- | --- | --- | --- | --- | --- | --- |
|  | **Minimum** | **Maximum** | **Length** |  |  | **Minimum** | **Maximum** | **Length** |
| **FWD. PRIMER** | 1 | 24 | 24 |  | **G2** | 318 | 328 | 11 |
| **A1** | 25 | 39 | 15 |  | **G3** | 329 | 343 | 15 |
| **A2** | 40 | 54 | 15 |  | **H1** | 344 | 358 | 15 |
| **A3** | 55 | 69 | 15 |  | **H2** | 359 | 373 | 15 |
| **B1** | 70 | 84 | 15 |  | **H3** | 374 | 389 | 16 |
| **B2** | 85 | 99 | 15 |  | **I1** | 390 | 404 | 15 |
| **B3** | 100 | 115 | 16 |  | **I2** | 405 | 420 | 16 |
| **C1** | 116 | 128 | 13 |  | **I3** | 421 | 435 | 15 |
| **C2** | 129 | 143 | 15 |  | **J1** | 436 | 450 | 15 |
| **C3** | 144 | 158 | 15 |  | **J2** | 451 | 461 | 11 |
| **D1** | 159 | 173 | 15 |  | **J3** | 462 | 476 | 15 |
| **D2** | 174 | 188 | 15 |  | **K1** | 477 | 491 | 15 |
| **D3** | 189 | 203 | 15 |  | **K2** | 492 | 506 | 15 |
| **E1** | 204 | 218 | 15 |  | **K3** | 507 | 521 | 15 |
| **E2** | 219 | 233 | 15 |  | **L1** | 522 | 536 | 15 |
| **E3** | 234 | 248 | 15 |  | **L2** | 537 | 551 | 15 |
| **F1** | 249 | 263 | 15 |  | **L3** | 552 | 566 | 15 |
| **F2** | 264 | 278 | 15 |  | **M1** | 567 | 581 | 15 |
| **F3** | 279 | 294 | 16 |  | **REV. PRIMER** | 588 | 621 | 34 |
| **G1** | 295 | 317 | 23 |  |  |  |  |  |

### Table A. tRNA val haplotypes identified for all samples included in the present analysis

| >tRNA_val_PART_A1_Hap_1_  CCTTCGGAAGAAGGA  >tRNA_val_PART_A1_Hap_2_  TCTTCGGAAGAAGAA  >tRNA_val_PART_A1_Hap_3_  TCTTTGGAAAAAGAA  >tRNA_val_PART_A1_Hap_4_  CCTTCGGAAGAAAGA  >tRNA_val_PART_A1_Hap_5_  TTTTTGGAAAAAGAA  >tRNA_val_PART_A1_Hap_6_  TTTTTGGAAAAAAAA  >tRNA_val_PART_A1_Hap_7_  TCTCCGGAAGAAGAA  >tRNA_val_PART_A1_Hap_8_  CTTTCGGAAGAAGGA  >tRNA_val_PART_A2_Hap_1_  TCTAGTCAATAGGAA  >tRNA_val_PART_A2_Hap_2_  TCTAGCCAATAGGAA  >tRNA_val_PART_A2_Hap_3_  TCTAGATTAGGAC  >tRNA_val_PART_A2_Hap_4_  TCTAGATTAGAAC  >tRNA_val_PART_A2_Hap_5_  TCTAGATAAATAGGAA  >tRNA_val_PART_A2_Hap_6_  TCTAGTCAGGGA  >tRNA_val_PART_A2_Hap_7_ TCTAGTTTAGAAT  >tRNA_val_PART_A2_Hap_8_ TCTAGTCTAGAAT  >tRNA_val_PART_A3_Hap_1_  ATAATATAAATTAGA  >tRNA_val_PART_A3_Hap_2_  ATAATATAAATCAGA  >tRNA_val_PART_A3_Hap_3_  ATAATATAAGTCAGA  >tRNA_val_PART_A3_Hap_4_  ATAATATAAATTGGA  >tRNA_val_PART_A3_Hap_5_  ATAGTATAAAAGA  >tRNA_val_PART_A3_Hap_6_  ATAATATAAACTGGA  >tRNA_val_PART_B1_Hap_1_  GTATACTTTACTTAC  >tRNA_val_PART_B1_Hap_2_  GTATATTTTATTTAC  >tRNA_val_PART_B1_Hap_3_  GTATATTTTACTTAC  >tRNA_val_PART_B2_Hap_1_  AATAAAAATGTAACG  >tRNA_val_PART_B2_Hap_2_  GATAAAGATGTATCA  >tRNA_val_PART_B2_Hap_3_  GATAAAGATGTATCG  >tRNA_val_PART_B2_Hap_4_  GATAAAGATGTATCT  >tRNA_val_PART_B2_Hap_5_  GATAAAGAAGTACTA  >tRNA_val_PART_B2_Hap_6_  AATAAAGAAGTAGTA  >tRNA_val_PART_B2_Hap_7_  AATAAAAAGGTTTAAAC  >tRNA_val_PART_B2_Hap_8_  AATAAAGATGTAGAA  >tRNA_val_PART_B3_Hap_1_  TCCTAATAACAAG  >tRNA_val_PART_B3_Hap_2_  TCCTAAAATAAA  >tRNA_val_PART_B3_Hap_3_  TCCTAACTAAA  >tRNA_val_PART_B3_Hap_4_  TCCTAATAGCAAG  >tRNA_val_PART_B3_Hap_5_  TTCTAACTAGCTATAT  >tRNA_val_PART_B3_Hap_6_  TTCTAACTAATTACATTA  >tRNA_val_PART_B3_Hap_7_  TTCTAACTAATTACATTC  >tRNA_val_PART_B3_Hap_8_  TCCTAATAATAAG  >tRNA_val_PART_B3_Hap_9_  TCCTAATAACAAA  >tRNA_val_PART_B3_Hap_10_  TCATAAAATAAA  >tRNA_val_PART_B3_Hap_11_  TCTGATTATTGTAAA  >tRNA_val_PART_B3_Hap_12_ TTCTAATCAGCCACAGCTCAATTTT  >tRNA_val_PART_B3_Hap_13_ TTCTAATCAGCCACAGGTCAATTTT  >tRNA_val_PART_C1_Hap_1_  CTATAAATTAACTTTA  >tRNA_val_PART_C1_Hap_2_  CTATAAATGTCAATTTTA  >tRNA_val_PART_C1_Hap_3_  CATAGCAAATATAAAACTAAAA  >tRNA_val_PART_C1_Hap_4_  TTATAAATGTCAATTTTA  >tRNA_val_PART_C1_Hap_5_  TAATTATTACATGTTTCATAATAT  >tRNA_val_PART_C1_Hap_6_  CTATAAACTAATTTTA  >tRNA_val_PART_C1_Hap_7_  CTTTTGCATGTTTCATTATAC  >tRNA_val_PART_C1_Hap_8_  TTTTGCATGTTTCATTATAC  >tRNA_val_PART_C1_Hap_9_  TTATAAATTAACTTTA  >tRNA_val_PART_C1_Hap_10_  TTATAAATTAATTTTA  >tRNA_val_PART_C1_Hap_11_  TAAATTATTTAAT  >tRNA_val_PART_C1_Hap_12_ CAGTAAACTTAT | >tRNA_val_PART_C1_Hap_13_ CTAGTAAACTTAT  >tRNA_val_PART_C2_Hap_1_  TTCCTTTTGCATAAG  >tRNA_val_PART_C2_Hap_2_  TTCCTTTTGCATCAG  >tRNA_val_PART_C2_Hap_3_  TTCCTTTTGTATAAG  >tRNA_val_PART_C3_Hap_1_  GATTAATTAACAATA  >tRNA_val_PART_C3_Hap_2_  GACTAATTAACAATA  >tRNA_val_PART_C3_Hap_3_  GATTAGTCAAAAATC  >tRNA_val_PART_C3_Hap_4_  GATTAATCAAAAATA  >tRNA_val_PART_C3_Hap_5_  GATTAGTTAAAAATA  >tRNA_val_PART_C3_Hap_6_  GATTAATTAAAAATA  >tRNA_val_PART_C3_Hap_7_  GATTAGTTAAGAATA  >tRNA_val_PART_C3_Hap_8_  GATTAATTGAAAATA  >tRNA_val_PART_C3_Hap_9_  GATTACTTTAAAATA  >tRNA_val_PART_D1_Hap_1_  AGAAAGTTTTCTACT  >tRNA_val_PART_D1_Hap_2_  AGAAAATTTTCTATC  >tRNA_val_PART_D1_Hap_3_  AGAAAACTTTCTATC  >tRNA_val_PART_D1_Hap_4_  AGAAAATTTTCTACC  >tRNA_val_PART_D1_Hap_5_  AGAAAAATTTCTACA  >tRNA_val_PART_D1_Hap_6_  AGAAGGTTTTCTACT  >tRNA_val_PART_D1_Hap_7_  AGAAAGTTTTCTATT  >tRNA_val_PART_D1_Hap_8_  AGAAAGTTTTCTGCT  >tRNA_val_PART_D1_Hap_9_  TGAAACTCTTCTATT  >tRNA_val_PART_D1_Hap_10_  GGAAAATTTTCCACC  >tRNA_val_PART_D1_Hap_11_  AGAAAAATTTCTATA  >tRNA_val_PART_D1_Hap_12_  AGAAATATTTCTAAT  >tRNA_val_PART_D1_Hap_13_  AGAAAATTTTCTAAA  >tRNA_val_PART_D2_Hap_1_  AAAGAAAAACACCCA  >tRNA_val_PART_D2_Hap_2_  AAAGAAAGCTCCCCA  >tRNA_val_PART_D2_Hap_3_  AAAGAAAATCCCCCA  >tRNA_val_PART_D2_Hap_4_  AAAGAAAAATATCGA  >tRNA_val_PART_D2_Hap_5_  AAAGAAGAACACCCA  >tRNA_val_PART_D2_Hap_6_  AAAGAAAATTAGTGA  >tRNA_val_PART_D2_Hap_7_  AAAGAAAAATACTGA  >tRNA_val_PART_D3_Hap_1_  CCTAAAAAAACATTA  >tRNA_val_PART_D3_Hap_2_  ACTAAAATAACAAAA  >tRNA_val_PART_D3_Hap_3_  ATTAAAATAACAAAA  >tRNA_val_PART_D3_Hap_4_  GCTAAAACAATAAAA  >tRNA_val_PART_D3_Hap_5_  ATTAAAGCAATAAAA  >tRNA_val_PART_D3_Hap_6_  GTTAAAACAATAAAA  >tRNA_val_PART_D3_Hap_7_  ATTAGAACAATAGCA  >tRNA_val_PART_D3_Hap_8_  TTTATTTTCATAAAA  >tRNA_val_PART_D3_Hap_9_  CTTAAAAAAACACTT  >tRNA_val_PART_E1_Hap_1_  CCTATATATTAAATA  >tRNA_val_PART_E1_Hap_2_  TCTATATGTTAAATG  >tRNA_val_PART_E1_Hap_3_  TCTGTATGTTAAATA  >tRNA_val_PART_E1_Hap_4_  TAAATATATTACATA  >tRNA_val_PART_E1_Hap_5_  CCTATATGTTAAATA  >tRNA_val_PART_E1_Hap_6_  TCTATATGTTAAATA  >tRNA_val_PART_E1_Hap_7_  AATATACATAACATG  >tRNA_val_PART_E1_Hap_8_  TTAATATATTACATA  >tRNA_val_PART_E2_Hap_1_  GATTTAAAATATAGT  >tRNA_val_PART_E2_Hap_2_  GATCAATAATGTTTT  >tRNA_val_PART_E2_Hap_3_  GATTAATAATGTTTT  >tRNA_val_PART_E2_Hap_4_  GATCTCAAATATAAT  >tRNA_val_PART_E2_Hap_5_  GATCCCAAATATAAT  >tRNA_val_PART_E2_Hap_6_  TATTTAAAATATAGT  >tRNA_val_PART_E2_Hap_7_  GATTATAAATATAGC  >tRNA_val_PART_E2_Hap_8_  GGCTTCAAATATGTC | >tRNA_val_PART_E2_Hap_9_  GATTAAAAATGTTTT  >tRNA_val_PART_E2_Hap_10_  TATGAAAAATATGAA  >tRNA_val_PART_E2_Hap_11_  TGTATAAAATGTTCT  >tRNA_val_PART_E3_Hap_1_  TTTAAGTAATATGCA  >tRNA_val_PART_E3_Hap_2_  TTTAGGTAATATGCA  >tRNA_val_PART_E3_Hap_3_  TTTAAGTCATATGCA  >tRNA_val_PART_E3_Hap_4_  TCTAAGCGAAATACA  >tRNA_val_PART_E3_Hap_5_  TTTACTTGTTATATGCA  >tRNA_val_PART_E3_Hap_6_  TTTATTCGTTACATGCA  >tRNA_val_PART_E3_Hap_7_  TTTATTCGTTATATGCA  >tRNA_val_PART_E3_Hap_8_  ATTAAAATTTTGTGACATGCA  >tRNA_val_PART_E3_Hap_9_  TTTAAAAGCCAAATAC  >tRNA_val_PART_F1_Hap_1_  ATTACGGTAGCTGAT  >tRNA_val_PART_F1_Hap_2_  ATTACGGTGATTGAT  >tRNA_val_PART_F1_Hap_3_  TTATGCTATTAGAT  >tRNA_val_PART_F1_Hap_4_  ATTACGGTGGCTGAT  >tRNA_val_PART_F1_Hap_5_  ATTACGGTGGTTGAT  >tRNA_val_PART_F1_Hap_6_  TTATGTTATTAGAT  >tRNA_val_PART_F1_Hap_7_  TTACACTAATGAAT  >tRNA_val_PART_F1_Hap_8_  ATTACATTGTTAGAT  >tRNA_val_PART_F2_Hap_1_  AACTTTTTAATTTTAG  >tRNA_val_PART_F2_Hap_2_  AACTTTTCAATTTAT  >tRNA_val_PART_F2_Hap_3_  ACCTTTTTATTCTT  >tRNA_val_PART_F2_Hap_4_  ATCTTTTTATTCTT  >tRNA_val_PART_F2_Hap_5_  AACTTTTCAATTTTAT  >tRNA_val_PART_F2_Hap_6_  AACTTTTTAATCTGCC  >tRNA_val_PART_F2_Hap_7_  AACTTTTTAATTTTAT  >tRNA_val_PART_F2_Hap_8_  AACTTTTTAAGTAATAA  >tRNA_val_PART_F2_Hap_9_  ACCTTTTTATTCAT  >tRNA_val_PART_F2_Hap_10_  ATCTTTTTATTTTAA  >tRNA_val_PART_F2_Hap_11_  ATCTTTTCGATTA  >tRNA_val_PART_F3_Hap_1_  TGTTAAGGACAATC  >tRNA_val_PART_F3_Hap_2_  ACCTTAAGGATTATC  >tRNA_val_PART_F3_Hap_3_  TAATTTAAGGATAATC  >tRNA_val_PART_F3_Hap_4_  ACCTTAAGGATAATC  >tRNA_val_PART_F3_Hap_5_  CGTTAAGGACAATC  >tRNA_val_PART_F3_Hap_6_  ACATTACGGACAACC  >tRNA_val_PART_F3_Hap_7_  CCCTTTATGGATAATC  >tRNA_val_PART_F3_Hap_8_  CTCTTTACGGATAAAC  >tRNA_val_PART_F3_Hap_9_  CTCTTTACGGATAATC  >tRNA_val_PART_F3_Hap_10_  TCTTTTAGGTTAATC  >tRNA_val_PART_F3_Hap_11_  ATATTTAAGGATAATC  >tRNA_val_PART_G2_Hap_1_  TATTATAAAAACCAA  >tRNA_val_PART_G2_Hap_2_  AATAATTAATAATTGA  >tRNA_val_PART_G2_Hap_3_  AAGAATACACATTA  >tRNA_val_PART_G2_Hap_4_  ATAAATGAAAAACCAA  >tRNA_val_PART_G2_Hap_5_  AATAATTAATAACTGA  >tRNA_val_PART_G2_Hap_6_  TATATTTAACTTAAACTA  >tRNA_val_PART_G2_Hap_7_  TATATTTTAACTTAGACTA  >tRNA_val_PART_G2_Hap_8_  AATAACTAATAACTGA  >tRNA_val_PART_G2_Hap_9_  AATAATTAATAGTTGA  >tRNA_val_PART_G2_Hap_10_  AGTAATTAATAACTGA  >tRNA_val_PART_G2_Hap_11_  TATTACAAAACCCAA  >tRNA_val_PART_G2_Hap_12_  GATTATTAATGATGAATTAA  >tRNA_val_PART_G2_Hap_13_  TATTACAAAAACCAA  >tRNA_val_PART_G2_Hap_14_  CATAAATGAAAAACCAA  >tRNA_val_PART_G2_Hap_15_  ACTAGTAGACATTCA  >tRNA_val_PART_G2_Hap_16_  ATCCATAATAA | >tRNA_val_PART_G3_Hap_1_  GTTTTAGGATTAGAA  >tRNA_val_PART_G3_Hap_2_  ATTTTAGGATTAGAA  >tRNA_val_PART_G3_Hap_3_  GTTTTAGGACTAGAA  >tRNA_val_PART_G3_Hap_4_  GTTTTAGGTTTAGAA  >tRNA_val_PART_G3_Hap_5_  ATCCTAGGACTAGAA  >tRNA_val_PART_G3_Hap_6_  ATAGTAGGATTAGAA  >tRNA_val_PART_G3_Hap_7_  ACAGTAGGATTAGAA  >tRNA_val_PART_G3_Hap_8_  ACAGTGGGATTAGAA  >tRNA_val_PART_G3_Hap_9_  ATTTTAGGACCAGAA  >tRNA_val_PART_G3_Hap_10_  ATTAAAGGATTAGAA  >tRNA_val_PART_G3_Hap_11_  GTATCTCAAAAGAAGGTTTAGAA  >tRNA_val_PART_H1_Hap_1_  GTAACCAAACTATTA  >tRNA_val_PART_H1_Hap_2_  GTAACCAAACCACTA  >tRNA_val_PART_H1_Hap_3_  ATAACCCAGCTATCG  >tRNA_val_PART_H1_Hap_4_  GTAACCAAACCATCG  >tRNA_val_PART_H1_Hap_5_  GTAACCAAGCTAACT  >tRNA_val_PART_H1_Hap_6_  GTAACCAAACCACCA  >tRNA_val_PART_H1_Hap_7_  GTAACCAAACCATTA  >tRNA_val_PART_H1_Hap_8_  GTAGCCAGACCATTA  >tRNA_val_PART_H1_Hap_9_  GTAACCAAACTAACT  >tRNA_val_PART_H1_Hap_10_  GTATCCTATTTAAAT  >tRNA_val_PART_H1_Hap_11_ GTAACCAGTCTAATA  >tRNA_val_PART_H1_Hap_12_ GTAACCAGTCTAATG  >tRNA_val_PART_H2_Hap_1_  ATTTGTAACAAAACA  >tRNA_val_PART_H2_Hap_2_  ATTTGTAGTAAAAT  >tRNA_val_PART_H2_Hap_3_  ATTTGTAACAAAAT  >tRNA_val_PART_H2_Hap_4_  ATTTGTAGCAAAATA  >tRNA_val_PART_H2_Hap_5_  ATTTGTAACAAAATA  >tRNA_val_PART_H2_Hap_6_  ACTTGTAACAAAACA  >tRNA_val_PART_H2_Hap_7_  ATTTGTAGCAAAACA  >tRNA_val_PART_H2_Hap_8_  ATTTGTAGTAAAATA  >tRNA_val_PART_H2_Hap_9_  TTTTGTAGTAAAACA  >tRNA_val_PART_H2_Hap_10_  TTTTGTAATAAAACA  >tRNA_val_PART_H2_Hap_11_  ATTTATAATAAAACA  >tRNA_val_PART_H2_Hap_12_  ATTTGTAGCAAAA  >tRNA_val_PART_H2_Hap_13_  TTTTATAATAAAAA  >tRNA_val_PART_H2_Hap_14_  TCTTATAGTAAAATA  >tRNA_val_PART_I3_Hap_1_  AAACATAAACTCAA  >tRNA_val_PART_I3_Hap_2_  AAACATAAATTCAA  >tRNA_val_PART_I3_Hap_3_  AAACGTTTATTCAGA  >tRNA_val_PART_I3_Hap_4_  AAACATAAGCTCAA  >tRNA_val_PART_I3_Hap_5_  AAACGTAAACTCAA  >tRNA_val_PART_I3_Hap_6_  AAACATTTATTTAA  >tRNA_val_PART_I3_Hap_7_  AAACATTTGTTCAA  >tRNA_val_PART_I3_Hap_8_  AAACACAAACTCAA  >tRNA_val_PART_I3_Hap_9_  AAACACAAGCTCAA  >tRNA_val_PART_I3_Hap_10_  AAACATGAACTCAA  >tRNA_val_PART_I3_Hap_11_  AAACAGCCATCATT  >tRNA_val_PART_I3_Hap_12_  AAATTGGTTTAA  >tRNA_val_PART_I3_Hap_13_  AAATTGATTTAA  >tRNA_val_PART_I3_Hap_14_  AAAAATCAAATCTAA  >tRNA_val_PART_I3_Hap_15_  AAATTAACTTAAA  >tRNA_val_PART_J1_Hap_1_  AAAAAAATAATTACG  >tRNA_val_PART_J1_Hap_2_  AAGAAAAATAATTATG  >tRNA_val_PART_J1_Hap_3_  AAAAAATATGATTTAAG  >tRNA_val_PART_J1_Hap_4_  AAAAAGAATAATTACG  >tRNA_val_PART_J1_Hap_5_  AATTATAATGACTAAG  >tRNA_val_PART_J1_Hap_6_  AAAAAAAATAATTATG | >tRNA_val_PART_J1_Hap_7_  AATTACAATGACTAAG  >tRNA_val_PART_J1_Hap_8_  AATAATAATGACTAAG  >tRNA_val_PART_J1_Hap_9_  TTTATAATAATTAAG  >tRNA_val_PART_J3_Hap_1_  AAATAAAATTCTAAT  >tRNA_val_PART_J3_Hap_2_  AAAAAAAATTCTAAT  >tRNA_val_PART_J3_Hap_3_  AAGTAAAATTTAAAT  >tRNA_val_PART_J3_Hap_4_  TCTAAAATCCTAAT  >tRNA_val_PART_J3_Hap_5_  AGGTAAAATTTAAAT  >tRNA_val_PART_J3_Hap_6_  TTAAAATCTTTAAT  >tRNA_val_PART_J3_Hap_7_  ATCTAAAAATCTTTAAT  >tRNA_val_PART_J3_Hap_8_  AAATAAAATTTAAAT  >tRNA_val_PART_J3_Hap_9_  ACATAAAATTTAAAT  >tRNA_val_PART_J3_Hap_10_  CAATAGAATTTATTAAT  >tRNA_val_PART_J3_Hap_11_  AACTTAATTCTATAAT  >tRNA_val_PART_K1_Hap_1_  TAAAATTCGGCACAT  >tRNA_val_PART_K1_Hap_2_  TAAAACTCAGCCCAA  >tRNA_val_PART_K1_Hap_3_  TAAAATTCAGCACAT  >tRNA_val_PART_K1_Hap_4_  TAAAATTCGGCATAT  >tRNA_val_PART_K1_Hap_5_  TAAAACTCGGCAAAT  >tRNA_val_PART_K1_Hap_6_  TAAAATTCAGCCCGG  >tRNA_val_PART_K1_Hap_7_  TAAAATTCAGCCCAA  >tRNA_val_PART_K1_Hap_8_  TAAAACTCTACAATT  >tRNA_val_PART_K1_Hap_9_  TAAAATTCAGCCTAT  >tRNA_val_PART_K2_Hap_1_  AATATACTACTGTTT  >tRNA_val_PART_K2_Hap_2_  AGTATACTACTGTTT  >tRNA_val_PART_K2_Hap_3_  CGCATACTAATGTTT  >tRNA_val_PART_K2_Hap_4_  AATATTCTACTGTTT  >tRNA_val_PART_K2_Hap_5_  ATCATGCTACTGTTT  >tRNA_val_PART_K3_Hap_1_  AATAAAAACATTGTA  >tRNA_val_PART_K3_Hap_2_  AATAAAAACATTGCA  >tRNA_val_PART_K3_Hap_3_  ATTAAAAACATTTTA  >tRNA_val_PART_K3_Hap_4_  AATAAAAACATTGTG  >tRNA_val_PART_L1_Hap_1_  ATATCCTTATATTGT  >tRNA_val_PART_L1_Hap_2_  ATATTGATATATTGT  >tRNA_val_PART_L1_Hap_3_  ATACCCCTGTGTTGT  >tRNA_val_PART_L1_Hap_4_  ATATTCTTATACTGT  >tRNA_val_PART_L1_Hap_5_  ATATTCTTATATTGT  >tRNA_val_PART_L1_Hap_6_  ATATATATCTATATTAT  >tRNA_val_PART_L1_Hap_7_  ATATATTTATATTAT  >tRNA_val_PART_L1_Hap_8_  ATATATCTATATTAT  >tRNA_val_PART_L1_Hap_9_  ATATCTAATATATTGT  >tRNA_val_PART_L1_Hap_10_  ATATTACTATATTAT  >tRNA_val_PART_L1_Hap_11_  GTATCTACATTTGTACCAT  >tRNA_val_PART_L2_Hap_1_  AAATTTCCTGCCCAA  >tRNA_val_PART_L2_Hap_2_  AAATCTCCTGCCCAA  >tRNA_val_PART_L2_Hap_3_  AATTCTCCTGCCCAA  >tRNA_val_PART_L2_Hap_4_  AAGTCTCCTGCCCAA  >tRNA_val_PART_L2_Hap_5_  ATATTCCTGCCCAC  >tRNA_val_PART_L2_Hap_6_  ATCTCTCCTGCCCAA  >tRNA_val_PART_L3_Hap_1_  TGATAAATTAAATGG  >tRNA_val_PART_L3_Hap_2_  TGATAAGTTAAATGG  >tRNA_val_PART_L3_Hap_3_  TGATAGATTAAATGG  >tRNA_val_PART_L3_Hap_4_  TGATTTATATTAAATGG  >tRNA_val_PART_L3_Hap_5_  TGATACATTTAAATGG  >tRNA_val_PART_L3_Hap_6_  TGATTATTTATTTGAATGG  >tRNA_val_PART_M1_Hap_1_  CCGCAGTAATCTGAC  >tRNA_val_PART_M1_Hap_2_  CCGCAGTAACCTGAC |
| --- | --- | --- | --- | --- |

## Defining microhaplotypes for the HVR-I amplicon

Relative to reference sequence AB699090.1 the forward and the reverse primers produce an amplicon extending from bases 45 to 905; a total of 861 bases. Eight microhaplotypes from 100 (*n* = 7) to 80 (*n* = 1) bases were defined along this amplicon as shown in the schematic below (Figure B). Table B below provides the sequence of all unique HVR-I microhaplotypes identified across all samples clustered in the present study. The haplotype data sheet in File S1, Tab A indicates the specific haplotype found at each region (defined in Figure S2) for every sample clustered in this study.

### Figure B. Schematic defining the microhaplotypes of the HVR-I amplicon

The gray bar represents the amplicon from bases 1 through 861, or bases 45 to 905 relative to the reference sequence (GenBank accession: AB699090.1). The priming sites (green) and the various microhaplotype regions (yellow) are indicated as orange colored bars.


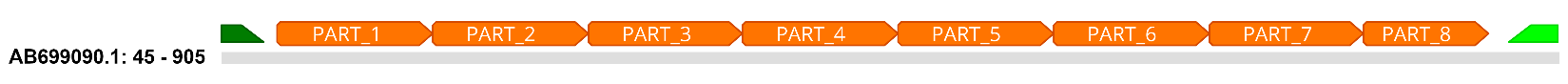


|  | **Base locations** | |  |
| --- | --- | --- | --- |
| **Name** | **Minimum** | **Maximum** | **Length** |
| **Forward Primer** | 1 | 27 | 27 |
| **PART_1** | 37 | 136 | 100 |
| **PART_2** | 137 | 236 | 100 |
| **PART_3** | 237 | 336 | 100 |
| **PART_4** | 337 | 436 | 100 |
| **PART_5** | 437 | 536 | 100 |
| **PART_6** | 537 | 636 | 100 |
| **PART_7** | 637 | 736 | 100 |
| **PART_8** | 737 | 816 | 80 |
| **Reverse Primer** | 830 | 861 | 32 |

### Table B. HVR-I haplotypes identified for all samples included in the present analysis†

| >HVR-I_PART_1_Hap_1_  GTCGCGCGCTGCGCGACGGAGCGCGTTTATTTGAACAAAACCAATCGGACGCAGGCTAGCTATTGGTCTGAGTCCGCGAAATGTTGGTGAATCGGAATAA  >HVR-I_PART_1_Hap_2_  GTCGCGCACTGCGCGTCGGAGCGCGTTTATTTGAACAAAACCAATCGGGCGCAGGCTAGCTATTGGCCTGAGTCCGCAAAATATTGGTGAATCGGAATAA  >HVR-I_PART_1_Hap_3_  GTCGCGCGCTGCGCGTCGGAGCGCGTTTATTTGAACAAAACCAATCGGACGCAGGCTAGCTATTGGTCTGAGTCCGCGAAATGTTGGTGAATCGGAATAA  >HVR-I_PART_1_Hap_4_  GTCGCGCGCTGCGCGTCGGAGCGCGTTTATTTGAACAAAACCAATCGGACGCAGGCTAGCTATTGGTCTGAGTCCGCGAAATGTCGGTGAATCGGAATAA  >HVR-I_PART_1_Hap_5_  GTCGCGCGCTGCGCGACGGAGCGCGTTTATTTGAACAAAACCAATCGGACGCAGGCTAGCTATTGGTCTGAGTCCGCGAAATTTTGGTGAATCGGAATAA  >HVR-I_PART_1_Hap_6_  GTCGCGCGCTGCGCGTCGGAGCGCGTTTATTTGAACAAAACCAATCGGACGCAGACTAGCTATTGGTCTGAGTCCGCGAAATGTTGGTGAATCGGAATAA  >HVR-I_PART_1_Hap_7_  GTCGCGCGCTGCGCGTCGGAGCGCGTTTATTTGAACGAAACCAATCGGACGCAGGCTAGCTATTGGTCTGAGTCCGCGAAATGTTGGTGAATCGGAATAA  >HVR-I_PART_1_Hap_8_  GTCGCGCGCTGCGCGTCGGAGCGCGTTTATTTGAACAAAACCAATCGGACGCAGGCTAGCTGTTGGTCTGAGTCCGCGAAATGTTGGTGAATCGGAATAA  >HVR-I_PART_1_Hap_9_  GTCGCGCTCTGCGCGTCGGAGCGCGTTTATTTGAACAAAACCAATCGGACGCAGGCTAGCTATTGGTCTGAGTCCGCGAAATGTTGGTGAATCGGAATAA  >HVR-I_PART_1_Hap_10_  GTCGCGCGCTGCGCGTACGGAGCGCAGTTTATTATGAACAAAACCAATCGGACGCAGGCTAGCTCTATTGGTCTGAGTCCGCGAAATGTTGGTGAATCGGAATAA  >HVR-I_PART_1_Hap_11_  TCGGCGCGCAATGCGGCGGAGCGCATTTATTGGTACAAAACCAATCGGACTTTCGCTCTTTGCGTCCGTCCGTCAACGGTTGGTGAATCGGAATAA  >HVR-I_PART_2_Hap_1_  CTATGCTGATCGCACGGTCCAGTACCGGCGACGATGCTTTGAAACGACTTGCTCATCAACTTTCGATGGTACGCTACGTGCTTACCATGGTGACAACGGT  >HVR-I_PART_2_Hap_2_  CTATGCTGATCGCACGGTCCAGTACCGGCGACGATACTTTGAAACGACTTGCTCATCAACTTTCGATGGTACGCTACGTGCTTACCATGGTGACAACGGT  >HVR-I_PART_2_Hap_3_  CTATGCTGATCGCACGGTCCGGTACCGGCGACGATGCTTTGAAACGACTTGCTCATCAACTTTCGATGGTACGCTACGTGCTTACCATGGTGACAACGGT  >HVR-I_PART_2_Hap_4_  CTATGCTGATCGCACGGTCCAGTACCGGCGACGGTGCTTTGAAACGACTTGCTCATCAACTTTCGATGGTACGCTACGTGCTTACCATGGTGACAACGGT  >HVR-I_PART_2_Hap_5_  CTATGCTGATCGCACGGTCCAGCACCGGCGACGATGCTTTGAAACGACTTGCTCATCAACTTTCGATGGTACGCTACGTGCTTACCATGGTGACAACGGT  >HVR-I_PART_2_Hap_6_  CTATGCTGATCGCACGGTCCAGCACCGGCGACGAATCTTTGAAATGACTTGCTCATCAACTTTCGATGGTACGCTACGTGCTTACCATGGTGACAACGGT  >HVR-I_PART_3_Hap_1_  TAACGGAGAATCAGGGTTCGGCTCCGGAGAGGGAGCCTGAGAAACGGCTACCACATCCAAGGAAGGCAGCAGGCACGCAAATTACCCACTCCCAGATCGG  >HVR-I_PART_3_Hap_2_  TAACGGAGAATCAGGGTTCGACTCCGGAGAGGGAGCCTGAGAAACGGCTACCACATCCAAGGAAGGCAGCAGGCACGCAAATTACCCACTCCCAGATCGG  >HVR-I_PART_3_Hap_3_  TAACGGAGAATCAGGGTTCGGCTCCGGAGAGGGAGCCTGAGAGACGGCTACCACATCCAAGGAAGGCAGCAGGCACGCAAATTACCCACTCCCAGATCGG  >HVR-I_PART_3_Hap_4_  TAACGGAGAATCAAGGTTCGGCTCCGGAGAGGGAGCCTGAGAAACGGCTACCACATCCAAGGAAGGCAGCAGGCACGCAAATTACCCACTCCCAGATCGG  >HVR-I_PART_3_Hap_5_  TAACGGAGAATCAGGGTTCGGCTCCGGAGAGGGAGCCTGAGAAACGGCTACCACATCCAAGGAAGGCAGCAGGCATGCAAATTACCCACTCCCAGATCGG  >HVR-I_PART_4_Hap_1_  GGAGGTAGTGACGAAAAATAACGGAACGTTTCTCCATGAGACGCGTTACCGGAACGATCGAGCCGTACATAAGTTCGGCTAAATCTATTGGAGGGCAAGT  >HVR-I_PART_4_Hap_2_  GGAGGTAGTGACGAAAAATAACGGAACGTTTCTCCATGAGACGCGTTACCGGAACGATCGAGCCGCACATAAGTTCGGCTAAATCTATTGGAGGGCAAGT  >HVR-I_PART_4_Hap_3_  GGAGGTAGTGACGAAAAATAACGGAACGTTTCTCCATGAGACGCGTTACCGGAACGATCGAGCCGTACATAAGTTCGGCTAAGTCTATTGGAGGGCAAGT  >HVR-I_PART_4_Hap_4_  GGAGGTAGTGACGAAAAATAACGGAACGTTTCTCCATGAGACGCGTTACCGGAACGATCGAGCCGTACATAAGTTCAGCTAAATCTATTGGAGGGAAAGT  >HVR-I_PART_4_Hap_5_  GGAGGTAGTGACGAAAAATAACGGAACGTATCTCCATGAGACGCGTTACCGGAACGACCGAGCCGTACATAAGCTCGGCTAATTCTATTGGAGGGCAAGT  >HVR-I_PART_5_Hap_1_  CTGGTGCCAGCAGCCGCGGTAATTCCAGCTCCAATAGCGTATATTAAAGTTGCTGCGGTTAAACCGCTCGTAGTTGGATTGCGGATGTCGACGACTGTCG  >HVR-I_PART_5_Hap_2_  CTGGTGCCAGCAGCCGCGGTAATTCCAGCTCCAATAGCGTATATTAAAGTTGCTGCGGTTAAACCGCTCGTAGTTGGATTGCGGATGTCGACGACGGTCG  >HVR-I_PART_5_Hap_3_  CTGGTGCCAGCAGTCGCGGTAATTCCAGCTCCAATAGCGTATATTAAAGTTGCTGCGGTTAAACCGCTCGTAGTTGGATTGCGGATGTCGACGACGGTCG  >HVR-I_PART_5_Hap_4_  CTGGTGCCAGCAGCCGCGGTAATTCCAGCTCCAATAGCGTATATCAAAGTTGCTGCGGTTAAACCGCTCGTAGTTGGATTGCGGATGTCGACGACGGTCG  >HVR-I_PART_5_Hap_5_  CTGGTGCCACCAGCCGCGGTAATTCCGGCTCCAATAGCGTATATTAAAGTTGCTGCGGTTAAACCGCTCGTAGTTGGATTGCGGATGTAGACCACGGACT  >HVR-I_PART_5_Hap_6_  CTGGTGCCAGCAGCCGCGGTAATTCCAGCTCCAATAGCGTATATTAAAGTTGCTGCGGTTAAACCGCTCGTAGTTGGATTTTGGTGCACGACGACGGTCG  >HVR-I_PART_6_Hap_1_  TCCTAAGCAGGAGTCGTTCCGTCGCTCGTCACCTGTTCGATCAAGATTGCCCTGGATGCTCTTCAGTGAGTGTCCTTGGCGACTTGAAAGTTTACTTTGA  >HVR-I_PART_6_Hap_2_  TCCTGAGCAGGAGTCGTTCCGTCGCTCGTCACCTGTTCGATCAAGATTGCCCTGGATGCTCTTCAGTGAGTGTCCTTGGCGACTTGAAAGTTTACTTTGA  >HVR-I_PART_6_Hap_3_  TCCTAAGCAGGAGTCGTTCTGTCGCTCGTCACCTGTTCGCTCAAGCTTGTCCTTGATGCTCTTGAGTGAGTGTCCTGGGCGACTTGAAAGTTTACTTTGA  >HVR-I_PART_7_Hap_1_  GAAAATGAGAGCGCTCAAGGCAAGCCGTAGTGCTTGAACCGTGGTGCATGGAATAATGAAAGATGGCCTCAGTGCTATTTTGTTGGTTTACGGCTATGAG  >HVR-I_PART_7_Hap_2_  GAAAATGAGAGCGCTCAAGGCAAGCCGTAGTGCTTGAACCGTGGTGCATGGAATAATGAAAGATGGCCTCAGTGCTATTTTGTTGGTTTACGGCTACGAG  >HVR-I_PART_7_Hap_3_  GAAAATGAGAGCGCTCAAGGCAAGCCGTAGTGCTTGAACCGTGGTGCATGGAATAATGAAAGATGGCCTCAGTGCTATTTCGTTGGTTTACGGCTATGAG  >HVR-I_PART_7_Hap_4_  GAAAATGAGAGCGCTCAAGGCAAGCCGTAATGCTTGAACCGTGGTGCATGGAATAATGAAAGATGGCCTCAGTGCTATTTTGTTGGTTTACGGCTATGAG  >HVR-I_PART_7_Hap_5_  GAAAATGAGAGCGCTCAAGGCAAGCCGTAGTGCTTGAACCGTGGTGCATGGAATAATGAAAGATGGCCTCAGTGCTATTTTGTTGGGTTTACGGTTTACGGCTATGAG  >HVR-I_PART_7_Hap_6_  GAAAATGGAAGCGCTCAAGGCAGGCCGTAGTGCTTGAACAGTGGTGCATGGAATAATGAAAGATGGCCTCGGTGCTATTTTGTTGGTTTACGGTGACGAG  >HVR-I_PART_8_Hap_1_  GCAATGATTAAAAGAGACTGACGGGGACATTCGTATTGCTGCGTTAGAGGTGAAATTCTTGGATCGCAGCAAGACGACCA  >HVR-I_PART_8_Hap_2_  GCAATGATTAAAAGAGACTGACGGGGACATTCGTATTGCTGCGTTGGAGGTGAAATTCTTGGATCGCAGCAAGACGACCA  >HVR-I_PART_8_Hap_3_  GCAATGATTAAAAGAGACTGACGGGGACATTCGTATTGCTGCGTTAGAGGTGAAATTCTTGGATCGCGGCAAGACGACCA  >HVR-I_PART_8_Hap_4_  GCAATGATTAAAAGAGACAGACGGGGACATTCGTATTGCTGCGTTAGAGGTGAAATTCTTGGATCGCAGCAAGACGAACA |
| --- |

†Excludes haplotypes from *Trichuris colobae.*

## Defining microhaplotypes for the HVR-IV amplicon

Relative to reference sequence MF288632.1 the forward and the reverse primers produce an amplicon extending from bases 235 to 672; a total of 438 bases. Four microhaplotypes between 100 and 80 bases were defined along this amplicon as shown in the schematic below (Figure C). Table C below provides the sequence of all unique HVR-I microhaplotypes identified across all samples clustered in the present study. The haplotype data sheet in S3 File, Tab A indicates the specific haplotype found at each region (defined in Figure C) for every sample clustered in this study.

### Figure C. Schematic defining the microhaplotypes of the HVR-I amplicon

The gray bar represents the amplicon from bases 1 through 438, or bases 235 to 672 relative to the reference sequence (GenBank accession: MF288632.1). The priming sites (green) and the various microhaplotype regions (yellow) are indicated as orange-colored bars.


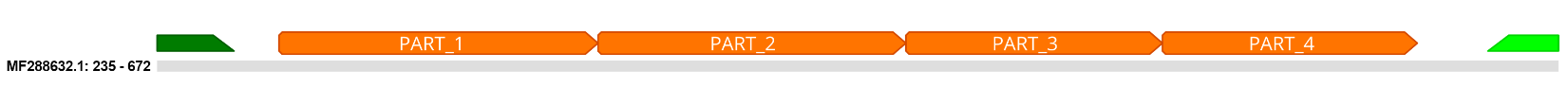


| **Name** | **Minimum** | **Maximum** | **Length** |
| --- | --- | --- | --- |
| **Forward primer** | 1 | 24 | 24 |
| **PART_1** | 39 | 138 | 100 |
| **PART_2** | 139 | 234 | 96 |
| **PART_3** | 235 | 314 | 80 |
| **PART_4** | 315 | 394 | 80 |
| **Reverse primer** | 417 | 438 | 22 |

### Table C. HVR-IV haplotypes identified for all samples included in the present analysis†

| >HVR-IV_PART_1_Hap_1_  TTGGTGGAGCGATTTGTCTGGCTAATTCCGATAACGAACGAGACTCTGGCCTACTAACTAGCGGCGGTATTCACGTCTCCCGCGCGACGCCGCGCTGCGC  >HVR-IV_PART_1_Hap_2_  TTGGTGGAGCGATTTGTCTGGCGAATTCCGATAACGAACGAGACTCTGGCCTACTAACTAGCGGCGGTATTCACGTCTCCCGCGCGACGCCGCGCTGCGC  >HVR-IV_PART_1_Hap_3_  TTGGTGGAGCGATTTGTCTGGCTAATTCCGATAACGAACGAGACTCTGGCCTACTAACTAGGGCCGGTATTCACGTCTCCCGCGCGACGCCGCGCTGCGC  >HVR-IV_PART_1_Hap_4_  TTGGTGGAACGATTTGTCTGGCTAATTCCGATATCGAACGAGACTCTGGCCTACTAACTAGCGGCGGTATTACGTCTCCCGCGCGACGCCGCGCTGCGC  >HVR-IV_PART_1_Hap_5_  TTGGTGGAGCGATTTGTCTGGCTAATTCCGATAACGAACGAGACTCTGGCCTACTAACTAGCGGCGGTATTCACGTCTCCCGCGCGACGCCGCGCTGCGT  >HVR-IV_PART_1_Hap_6_  TTGGTGGAGCGATTTGTCTGGCTAATTCCGATAACGAACGAGACTCTGGCCTACTAACTAGCGGCGGTATTCACGTCTCCTGCGCGACGCCGCGCTGCGT  >HVR-IV_PART_1_Hap_7_  TTGGTGGAGCGATTTGTCTGGCTAATTCCGATAACGAACGAGACTCTGGCCTACTAACTAGCGGCGGTATTCACGTCTGCCGCGCGACGCCGCGCTGCGT  >HVR-IV_PART_1_Hap_8_  TTGGTGGAGCGATTTGTCTGGCTAATTCCGATAACGAACGAGACTCTGGCCTACTAACTAGCGGCGGTGTTCATGTCTCCTGTATGGCCGCA  >HVR-IV_PART_2_Hap_1_  TGCGGCTGAGCACAGTCGTCGCCGTTTGCGCGAGCGCTCCGCGGAGCACCTGCAGTGCCGGCAGCCGCTTCTTAGAGGGACCAGCGACACTTTCGC  >HVR-IV_PART_2_Hap_2_  TGCGGCTGAGCACAGTCGTCGCCGTTTGCGCGAGCGCTCCGCGGAGCACCTGTAGTGCCGGCAGCCGCTTCTTAGAGGGACCAGCGACACTTTCGC  >HVR-IV_PART_2_Hap_3_  TTCGGCTGAGCACAGTCGTCGCCGTTTGCGCGAGCGCTCCGCGGAGCACCTGTAGTGCCGGCAGCCGCTTCTTAGAGGGACCAGCGACACTTTCGC  >HVR-IV_PART_2_Hap_4_  CACGGCGCGGCTGAACACAGTCGTCGTCGTCGTCGCCGTTTGCGCGAGCGCTCCGCGGAGCACTTGCAGTGCCGGTAGCCGCTTCTTAGAGGGACCAGCGACACTTTCGC  >HVR-IV_PART_2_Hap_5_  CACGGCGCGGCTGAGCACAGTCGTCGTCGTCGTCGCCGTTTGCGCGAGCGCTCCGCGGAGCACTTGCAGTGCCGGCAGCCGCTTCTTAGAGGGACCAGCGACACTTTCGC  >HVR-IV_PART_2_Hap_6_  GACGGTTGCGCGCTCGTCGTTACAACCGCTGCGCGCTCTATGGAGCAGCAGCGCCGGCAGCCGCTTCTTAGAGGGACCAGCGACACTTTCGC  >HVR-IV_PART_3_Hap_1_  AAGCCGCACGAGAAAGAGCAATAACAGGTCTGTGATGCCCTTAGATGTACGGGGCTGCACGCGTGCTACACTGACGGCGT  >HVR-IV_PART_3_Hap_2_  AAGCCGCACGAGGAAGAGCAATAACAGGTCTGTGATGCCCTTAGATGTACGGGGCTGCACGCGTGCTACACTGACGGCGT  >HVR-IV_PART_3_Hap_3_  AAGCCGCACGAGAAAGAGCAATAACAGGTCTGTGATGCCCTTAGATGTACGGGGCTGCACGCGTGCTACACTGACGGCGA  >HVR-IV_PART_4_Hap_1_  CAGCGTGCGTTCAAGCCCGGCCTGGCAAGGCCAGGAAATCGGTTGAAACGTTCTCGTGACTGGGACAGGGAATTGCAATT  >HVR-IV_PART_4_Hap_2_  CAGCGTGCGTTCAAGCCCGGCCTGGCAAGGCCAGGAAATCGGTTGAAACGTTCTCGTGACTGGGACAGGTAATTGCAATT  >HVR-IV_PART_4_Hap_3_  CAGCGTGCGTTCAAGCCCGGCCTGCCAAGGTCGGGAAATCGGTTGAAACGTTCTCGTGACTGGGACAGGGAATTGAAATT |
| --- |

†Excludes haplotypes from *Trichuris colobae.*

## Full length 18S rDNA haplotypes

#### 18S rDNA HVR-I full-length haplotypes

>HVR-I_Hap_I

CAGTC-GCGCGCTGCGCG-ACGGAGCGC-GTTTATT-TGAACAAAACCAATCGGACGCAGGCTAG--CTATTGGTCTGAGTCCGCGAAATGTTGGTGAATCGGAATAACTATGCTGATCGCACGGTCCAGTACCGGCGACGATGCTTTGAAACGACTTGCTCATCAACTTTCGATGGTACGCTACGTGCTTACCATGGTGACAACGGTTAACGGAGAATCAGGGTTCGGCTCCGGAGAGGGAGCCTGAGAAACGGCTACCACATCCAAGGAAGGCAGCAGGCACGCAAATTACCCACTCCCAGATCGGGGAGGTAGTGACGAAAAATAACGGAACGTTTCTCCATGAGACGCGTTACCGGAACGATCGAGCCGTACATAAGTTCGGCTAAATCTATTGGAGGGCAAGTCTGGTGCCAGCAGCCGCGGTAATTCCAGCTCCAATAGCGTATATTAAAGTTGCTGCGGTTAAACCGCTCGTAGTTGGATTGCGGATGTCGACGACGGTCGTCCTAAGCAGGAGTCGTTCCGTCGCTCGTCACCTGTTCGATCAAGATTGCCCTGGATGCTCTTCAGTGAGTGTCCTTGGCGACTTGAAAGTTTACTTTGAGAAAATGAGAGCGCTCAAGGCAAGCCGTAGTGCTTGAACCGTGGTGCATGGAATAATGAAAGATGGCCTCAGTGCTATTTTGTT--------GGTTTACGGCTATGAGGCAATGATTAAAAGAGACTGACGGGGACATTCGTATTGCTGCGTTAGAGGTGAAATTCTTGGATCGCAGCAAGACGACCAAT

>HVR-I_Hap_II

CAGTC-GCGCGCTGCGCGT-CGGAGCGC-GTTTATT-TGAACAAAACCAATCGGACGCAGGCTAG--CTATTGGTCTGAGTCCGCGAAATGTTGGTGAATCGGAATAACTATGCTGATCGCACGGTCCAGTACCGGCGACGATGCTTTGAAACGACTTGCTCATCAACTTTCGATGGTACGCTACGTGCTTACCATGGTGACAACGGTTAACGGAGAATCAGGGTTCGGCTCCGGAGAGGGAGCCTGAGAAACGGCTACCACATCCAAGGAAGGCAGCAGGCACGCAAATTACCCACTCCCAGATCGGGGAGGTAGTGACGAAAAATAACGGAACGTTTCTCCATGAGACGCGTTACCGGAACGATCGAGCCGTACATAAGTTCGGCTAAATCTATTGGAGGGCAAGTCTGGTGCCAGCAGCCGCGGTAATTCCAGCTCCAATAGCGTATATTAAAGTTGCTGCGGTTAAACCGCTCGTAGTTGGATTGCGGATGTCGACGACGGTCGTCCTAAGCAGGAGTCGTTCCGTCGCTCGTCACCTGTTCGATCAAGATTGCCCTGGATGCTCTTCAGTGAGTGTCCTTGGCGACTTGAAAGTTTACTTTGAGAAAATGAGAGCGCTCAAGGCAAGCCGTAGTGCTTGAACCGTGGTGCATGGAATAATGAAAGATGGCCTCAGTGCTATTTTGTT--------GGTTTACGGCTATGAGGCAATGATTAAAAGAGACTGACGGGGACATTCGTATTGCTGCGTTAGAGGTGAAATTCTTGGATCGCAGCAAGACGACCAAT

>HVR-I_Hap_III

CAGTC-GCGCGCTGCGCGT-CGGAGCGC-GTTTATT-TGAACAAAACCAATCGGACGCAGACTAG--CTATTGGTCTGAGTCCGCGAAATGTTGGTGAATCGGAATAACTATGCTGATCGCACGGTCCAGTACCGGCGACGATGCTTTGAAACGACTTGCTCATCAACTTTCGATGGTACGCTACGTGCTTACCATGGTGACAACGGTTAACGGAGAATCAGGGTTCGGCTCCGGAGAGGGAGCCTGAGAAACGGCTACCACATCCAAGGAAGGCAGCAGGCACGCAAATTACCCACTCCCAGATCGGGGAGGTAGTGACGAAAAATAACGGAACGTTTCTCCATGAGACGCGTTACCGGAACGATCGAGCCGTACATAAGTTCGGCTAAATCTATTGGAGGGCAAGTCTGGTGCCAGCAGCCGCGGTAATTCCAGCTCCAATAGCGTATATTAAAGTTGCTGCGGTTAAACCGCTCGTAGTTGGATTGCGGATGTCGACGACGGTCGTCCTAAGCAGGAGTCGTTCCGTCGCTCGTCACCTGTTCGATCAAGATTGCCCTGGATGCTCTTCAGTGAGTGTCCTTGGCGACTTGAAAGTTTACTTTGAGAAAATGAGAGCGCTCAAGGCAAGCCGTAGTGCTTGAACCGTGGTGCATGGAATAATGAAAGATGGCCTCAGTGCTATTTTGTT--------GGTTTACGGCTATGAGGCAATGATTAAAAGAGACTGACGGGGACATTCGTATTGCTGCGTTAGAGGTGAAATTCTTGGATCGCAGCAAGACGACCAAT

>HVR-I_Hap_IV

CAGTC-GCGCGCTGCGCGT-CGGAGCGC-GTTTATT-TGAACGAAACCAATCGGACGCAGGCTAG--CTATTGGTCTGAGTCCGCGAAATGTTGGTGAATCGGAATAACTATGCTGATCGCACGGTCCAGTACCGGCGACGATGCTTTGAAACGACTTGCTCATCAACTTTCGATGGTACGCTACGTGCTTACCATGGTGACAACGGTTAACGGAGAATCAGGGTTCGGCTCCGGAGAGGGAGCCTGAGAAACGGCTACCACATCCAAGGAAGGCAGCAGGCACGCAAATTACCCACTCCCAGATCGGGGAGGTAGTGACGAAAAATAACGGAACGTTTCTCCATGAGACGCGTTACCGGAACGATCGAGCCGTACATAAGTTCGGCTAAATCTATTGGAGGGCAAGTCTGGTGCCAGCAGCCGCGGTAATTCCAGCTCCAATAGCGTATATTAAAGTTGCTGCGGTTAAACCGCTCGTAGTTGGATTGCGGATGTCGACGACGGTCGTCCTAAGCAGGAGTCGTTCCGTCGCTCGTCACCTGTTCGATCAAGATTGCCCTGGATGCTCTTCAGTGAGTGTCCTTGGCGACTTGAAAGTTTACTTTGAGAAAATGAGAGCGCTCAAGGCAAGCCGTAGTGCTTGAACCGTGGTGCATGGAATAATGAAAGATGGCCTCAGTGCTATTTTGTT--------GGTTTACGGCTATGAGGCAATGATTAAAAGAGACTGACGGGGACATTCGTATTGCTGCGTTAGAGGTGAAATTCTTGGATCGCAGCAAGACGACCAAT

>HVR-I_Hap_V

CAGTC-GCGCGCTGCGCGT-CGGAGCGC-GTTTATT-TGAACAAAACCAATCGGACGCAGGCTAG--CTATTGGTCTGAGTCCGCGAAATGTTGGTGAATCGGAATAACTATGCTGATCGCACGGTCCAGTACCGGCGACGATGCTTTGAAACGACTTGCTCATCAACTTTCGATGGTACGCTACGTGCTTACCATGGTGACAACGGTTAACGGAGAATCAGGGTTCGACTCCGGAGAGGGAGCCTGAGAAACGGCTACCACATCCAAGGAAGGCAGCAGGCACGCAAATTACCCACTCCCAGATCGGGGAGGTAGTGACGAAAAATAACGGAACGTTTCTCCATGAGACGCGTTACCGGAACGATCGAGCCGTACATAAGTTCGGCTAAATCTATTGGAGGGCAAGTCTGGTGCCAGCAGCCGCGGTAATTCCAGCTCCAATAGCGTATATTAAAGTTGCTGCGGTTAAACCGCTCGTAGTTGGATTGCGGATGTCGACGACGGTCGTCCTAAGCAGGAGTCGTTCCGTCGCTCGTCACCTGTTCGATCAAGATTGCCCTGGATGCTCTTCAGTGAGTGTCCTTGGCGACTTGAAAGTTTACTTTGAGAAAATGAGAGCGCTCAAGGCAAGCCGTAGTGCTTGAACCGTGGTGCATGGAATAATGAAAGATGGCCTCAGTGCTATTTTGTT--------GGTTTACGGCTATGAGGCAATGATTAAAAGAGACTGACGGGGACATTCGTATTGCTGCGTTAGAGGTGAAATTCTTGGATCGCAGCAAGACGACCAAT

>HVR-I_Hap_VI

CAGTC-GCGCGCTGCGCGT-CGGAGCGC-GTTTATT-TGAACAAAACCAATCGGACGCAGGCTAG--CTATTGGTCTGAGTCCGCGAAATGTTGGTGAATCGGAATAACTATGCTGATCGCACGGTCCAGTACCGGCGACGATGCTTTGAAACGACTTGCTCATCAACTTTCGATGGTACGCTACGTGCTTACCATGGTGACAACGGTTAACGGAGAATCAGGGTTCGGCTCCGGAGAGGGAGCCTGAGAAACGGCTACCACATCCAAGGAAGGCAGCAGGCACGCAAATTACCCACTCCCAGATCGGGGAGGTAGTGACGAAAAATAACGGAACGTTTCTCCATGAGACGCGTTACCGGAACGATCGAGCCGTACATAAGTTCGGCTAAATCTATTGGAGGGCAAGTCTGGTGCCAGCAGCCGCGGTAATTCCAGCTCCAATAGCGTATATTAAAGTTGCTGCGGTTAAACCGCTCGTAGTTGGATTGCGGATGTCGACGACGGTCGTCCTGAGCAGGAGTCGTTCCGTCGCTCGTCACCTGTTCGATCAAGATTGCCCTGGATGCTCTTCAGTGAGTGTCCTTGGCGACTTGAAAGTTTACTTTGAGAAAATGAGAGCGCTCAAGGCAAGCCGTAGTGCTTGAACCGTGGTGCATGGAATAATGAAAGATGGCCTCAGTGCTATTTTGTT--------GGTTTACGGCTATGAGGCAATGATTAAAAGAGACTGACGGGGACATTCGTATTGCTGCGTTAGAGGTGAAATTCTTGGATCGCAGCAAGACGACCAAT

>HVR-I_Hap_VII

CAGTC-GCGCGCTGCGCGT-CGGAGCGC-GTTTATT-TGAACAAAACCAATCGGACGCAGGCTAG--CTGTTGGTCTGAGTCCGCGAAATGTTGGTGAATCGGAATAACTATGCTGATCGCACGGTCCAGTACCGGCGACGATGCTTTGAAACGACTTGCTCATCAACTTTCGATGGTACGCTACGTGCTTACCATGGTGACAACGGTTAACGGAGAATCAGGGTTCGGCTCCGGAGAGGGAGCCTGAGAAACGGCTACCACATCCAAGGAAGGCAGCAGGCACGCAAATTACCCACTCCCAGATCGGGGAGGTAGTGACGAAAAATAACGGAACGTTTCTCCATGAGACGCGTTACCGGAACGATCGAGCCGTACATAAGTTCGGCTAAATCTATTGGAGGGCAAGTCTGGTGCCAGCAGCCGCGGTAATTCCAGCTCCAATAGCGTATATTAAAGTTGCTGCGGTTAAACCGCTCGTAGTTGGATTGCGGATGTCGACGACGGTCGTCCTAAGCAGGAGTCGTTCCGTCGCTCGTCACCTGTTCGATCAAGATTGCCCTGGATGCTCTTCAGTGAGTGTCCTTGGCGACTTGAAAGTTTACTTTGAGAAAATGAGAGCGCTCAAGGCAAGCCGTAGTGCTTGAACCGTGGTGCATGGAATAATGAAAGATGGCCTCAGTGCTATTTTGTT--------GGTTTACGGCTATGAGGCAATGATTAAAAGAGACTGACGGGGACATTCGTATTGCTGCGTTAGAGGTGAAATTCTTGGATCGCAGCAAGACGACCAAT

>HVR-I_Hap_VIII

CAGTC-GCGCTCTGCGCGT-CGGAGCGC-GTTTATT-TGAACAAAACCAATCGGACGCAGGCTAG--CTATTGGTCTGAGTCCGCGAAATGTTGGTGAATCGGAATAACTATGCTGATCGCACGGTCCAGTACCGGCGACGATGCTTTGAAACGACTTGCTCATCAACTTTCGATGGTACGCTACGTGCTTACCATGGTGACAACGGTTAACGGAGAATCAGGGTTCGGCTCCGGAGAGGGAGCCTGAGAAACGGCTACCACATCCAAGGAAGGCAGCAGGCACGCAAATTACCCACTCCCAGATCGGGGAGGTAGTGACGAAAAATAACGGAACGTTTCTCCATGAGACGCGTTACCGGAACGATCGAGCCGTACATAAGTTCGGCTAAATCTATTGGAGGGCAAGTCTGGTGCCAGCAGCCGCGGTAATTCCAGCTCCAATAGCGTATATTAAAGTTGCTGCGGTTAAACCGCTCGTAGTTGGATTGCGGATGTCGACGACGGTCGTCCTAAGCAGGAGTCGTTCCGTCGCTCGTCACCTGTTCGATCAAGATTGCCCTGGATGCTCTTCAGTGAGTGTCCTTGGCGACTTGAAAGTTTACTTTGAGAAAATGAGAGCGCTCAAGGCAAGCCGTAGTGCTTGAACCGTGGTGCATGGAATAATGAAAGATGGCCTCAGTGCTATTTTGTT--------GGTTTACGGCTACGAGGCAATGATTAAAAGAGACTGACGGGGACATTCGTATTGCTGCGTTAGAGGTGAAATTCTTGGATCGCAGCAAGACGACCAAT

>HVR-I_Hap_IX

CAGTC-GCGCGCTGCGCGT-CGGAGCGC-GTTTATT-TGAACAAAACCAATCGGACGCAGGCTAG--CTATTGGTCTGAGTCCGCGAAATGTTGGTGAATCGGAATAACTATGCTGATCGCACGGTCCGGTACCGGCGACGATGCTTTGAAACGACTTGCTCATCAACTTTCGATGGTACGCTACGTGCTTACCATGGTGACAACGGTTAACGGAGAATCAGGGTTCGGCTCCGGAGAGGGAGCCTGAGAAACGGCTACCACATCCAAGGAAGGCAGCAGGCACGCAAATTACCCACTCCCAGATCGGGGAGGTAGTGACGAAAAATAACGGAACGTTTCTCCATGAGACGCGTTACCGGAACGATCGAGCCGTACATAAGTTCGGCTAAATCTATTGGAGGGCAAGTCTGGTGCCAGCAGCCGCGGTAATTCCAGCTCCAATAGCGTATATTAAAGTTGCTGCGGTTAAACCGCTCGTAGTTGGATTGCGGATGTCGACGACGGTCGTCCTAAGCAGGAGTCGTTCCGTCGCTCGTCACCTGTTCGATCAAGATTGCCCTGGATGCTCTTCAGTGAGTGTCCTTGGCGACTTGAAAGTTTACTTTGAGAAAATGAGAGCGCTCAAGGCAAGCCGTAGTGCTTGAACCGTGGTGCATGGAATAATGAAAGATGGCCTCAGTGCTATTTCGTT--------GGTTTACGGCTATGAGGCAATGATTAAAAGAGACTGACGGGGACATTCGTATTGCTGCGTTAGAGGTGAAATTCTTGGATCGCAGCAAGACGACCAAT

>HVR-I_Hap_X

CAGTC-GCGCGCTGCGCGT-CGGAGCGC-GTTTATT-TGAACAAAACCAATCGGACGCAGGCTAG--CTATTGGTCTGAGTCCGCGAAATGTTGGTGAATCGGAATAACTATGCTGATCGCACGGTCCAGTACCGGCGACGATGCTTTGAAACGACTTGCTCATCAACTTTCGATGGTACGCTACGTGCTTACCATGGTGACAACGGTTAACGGAGAATCAGGGTTCGGCTCCGGAGAGGGAGCCTGAGAAACGGCTACCACATCCAAGGAAGGCAGCAGGCACGCAAATTACCCACTCCCAGATCGGGGAGGTAGTGACGAAAAATAACGGAACGTTTCTCCATGAGACGCGTTACCGGAACGATCGAGCCGTACATAAGTTCGGCTAAATCTATTGGAGGGCAAGTCTGGTGCCAGCAGCCGCGGTAATTCCAGCTCCAATAGCGTATATTAAAGTTGCTGCGGTTAAACCGCTCGTAGTTGGATTGCGGATGTCGACGACGGTCGTCCTAAGCAGGAGTCGTTCCGTCGCTCGTCACCTGTTCGATCAAGATTGCCCTGGATGCTCTTCAGTGAGTGTCCTTGGCGACTTGAAAGTTTACTTTGAGAAAATGAGAGCGCTCAAGGCAAGCCGTAATGCTTGAACCGTGGTGCATGGAATAATGAAAGATGGCCTCAGTGCTATTTTGTT--------GGTTTACGGCTATGAGGCAATGATTAAAAGAGACTGACGGGGACATTCGTATTGCTGCGTTGGAGGTGAAATTCTTGGATCGCAGCAAGACGACCAAT

>HVR-I_Hap_XI

CAGTC-GCGCGCTGCGCGT-CGGAGCGC-GTTTATT-TGAACAAAACCAATCGGACGCAGGCTAG--CTATTGGTCTGAGTCCGCGAAATGTTGGTGAATCGGAATAACTATGCTGATCGCACGGTCCAGTACCGGCGACGATGCTTTGAAACGACTTGCTCATCAACTTTCGATGGTACGCTACGTGCTTACCATGGTGACAACGGTTAACGGAGAATCAGGGTTCGGCTCCGGAGAGGGAGCCTGAGAGACGGCTACCACATCCAAGGAAGGCAGCAGGCACGCAAATTACCCACTCCCAGATCGGGGAGGTAGTGACGAAAAATAACGGAACGTTTCTCCATGAGACGCGTTACCGGAACGATCGAGCCGTACATAAGTTCGGCTAAATCTATTGGAGGGCAAGTCTGGTGCCAGCAGTCGCGGTAATTCCAGCTCCAATAGCGTATATTAAAGTTGCTGCGGTTAAACCGCTCGTAGTTGGATTGCGGATGTCGACGACGGTCGTCCTAAGCAGGAGTCGTTCCGTCGCTCGTCACCTGTTCGATCAAGATTGCCCTGGATGCTCTTCAGTGAGTGTCCTTGGCGACTTGAAAGTTTACTTTGAGAAAATGAGAGCGCTCAAGGCAAGCCGTAGTGCTTGAACCGTGGTGCATGGAATAATGAAAGATGGCCTCAGTGCTATTTTGTT--------GGTTTACGGCTATGAGGCAATGATTAAAAGAGACTGACGGGGACATTCGTATTGCTGCGTTAGAGGTGAAATTCTTGGATCGCAGCAAGACGACCAAT

>HVR-I_Hap_XII

CAGTC-GCGCGCTGCGCG-ACGGAGCGC-GTTTATT-TGAACAAAACCAATCGGACGCAGGCTAG--CTATTGGTCTGAGTCCGCGAAATGTTGGTGAATCGGAATAACTATGCTGATCGCACGGTCCAGTACCGGCGACGGTGCTTTGAAACGACTTGCTCATCAACTTTCGATGGTACGCTACGTGCTTACCATGGTGACAACGGTTAACGGAGAATCAAGGTTCGGCTCCGGAGAGGGAGCCTGAGAAACGGCTACCACATCCAAGGAAGGCAGCAGGCACGCAAATTACCCACTCCCAGATCGGGGAGGTAGTGACGAAAAATAACGGAACGTTTCTCCATGAGACGCGTTACCGGAACGATCGAGCCGTACATAAGTTCGGCTAAGTCTATTGGAGGGCAAGTCTGGTGCCAGCAGCCGCGGTAATTCCAGCTCCAATAGCGTATATCAAAGTTGCTGCGGTTAAACCGCTCGTAGTTGGATTGCGGATGTCGACGACGGTCGTCCTAAGCAGGAGTCGTTCCGTCGCTCGTCACCTGTTCGATCAAGATTGCCCTGGATGCTCTTCAGTGAGTGTCCTTGGCGACTTGAAAGTTTACTTTGAGAAAATGAGAGCGCTCAAGGCAAGCCGTAGTGCTTGAACCGTGGTGCATGGAATAATGAAAGATGGCCTCAGTGCTATTTTGTT--------GGTTTACGGCTATGAGGCAATGATTAAAAGAGACTGACGGGGACATTCGTATTGCTGCGTTAGAGGTGAAATTCTTGGATCGCAGCAAGACGACCAAT

>HVR-I_Hap_XIII

CAGTC-GCGCGCTGCGCGT-CGGAGCGC-GTTTATT-TGAACAAAACCAATCGGACGCAGGCTAG--CTATTGGTCTGAGTCCGCGAAATGTTGGTGAATCGGAATAACTATGCTGATCGCACGGTCCAGTACCGGCGACGATGCTTTGAAACGACTTGCTCATCAACTTTCGATGGTACGCTACGTGCTTACCATGGTGACAACGGTTAACGGAGAATCAGGGTTCGGCTCCGGAGAGGGAGCCTGAGAAACGGCTACCACATCCAAGGAAGGCAGCAGGCATGCAAATTACCCACTCCCAGATCGGGGAGGTAGTGACGAAAAATAACGGAACGTTTCTCCATGAGACGCGTTACCGGAACGATCGAGCCGTACATAAGTTCGGCTAAATCTATTGGAGGGCAAGTCTGGTGCCAGCAGCCGCGGTAATTCCAGCTCCAATAGCGTATATTAAAGTTGCTGCGGTTAAACCGCTCGTAGTTGGATTGCGGATGTCGACGACGGTCGTCCTAAGCAGGAGTCGTTCCGTCGCTCGTCACCTGTTCGATCAAGATTGCCCTGGATGCTCTTCAGTGAGTGTCCTTGGCGACTTGAAAGTTTACTTTGAGAAAATGAGAGCGCTCAAGGCAAGCCGTAGTGCTTGAACCGTGGTGCATGGAATAATGAAAGATGGCCTCAGTGCTATTTTGTTGGGTTTACGGTTTACGGCTATGAGGCAATGATTAAAAGAGACTGACGGGGACATTCGTATTGCTGCGTTAGAGGTGAAATTCTTGGATCGCAGCAAGACGACCAAT

>HVR-I_Hap_XIV

CAGTC-GCGCGCTGCGCGTACGGAGCGCAGTTTATTATGAACAAAACCAATCGGACGCAGGCTAGCTCTATTGGTCTGAGTCCGCGAAATGTTGGTGAATCGGAATAACTATGCTGATCGCACGGTCCAGTACCGGCGACGATGCTTTGAAACGACTTGCTCATCAACTTTCGATGGTACGCTACGTGCTTACCATGGTGACAACGGTTAACGGAGAATCAGGGTTCGGCTCCGGAGAGGGAGCCTGAGAAACGGCTACCACATCCAAGGAAGGCAGCAGGCACGCAAATTACCCACTCCCAGATCGGGGAGGTAGTGACGAAAAATAACGGAACGTTTCTCCATGAGACGCGTTACCGGAACGATCGAGCCGTACATAAGTTCGGCTAAATCTATTGGAGGGCAAGTCTGGTGCCAGCAGCCGCGGTAATTCCAGCTCCAATAGCGTATATTAAAGTTGCTGCGGTTAAACCGCTCGTAGTTGGATTGCGGATGTCGACGACGGTCGTCCTAAGCAGGAGTC-----------------------------------------------------------------------------------------------------------------------------------------------------------------------------------------------------------------------------------------------------------------------------------

>HVR-I_Hap_XV

CAGTC-GCGCGCTGCGCGTACGGAGCGCAGTTTATTATGAACAAAACCAATCGGACGCAGGCTAGCTCTATTGGTCTGAGTCCGCGAAATGTTGGTGAATCGGAATAACTATGCTGATCGCACGGTCCAGCACCGGCGACGATGCTTTGAAACGACTTGCTCATCAACTTTCGATGGTACGCTACGTGCTTACCATGGTGACAACGGTTAACGGAGAATCAGGGTTCGGCTCCGGAGAGGGAGCCTGAGAAACGGCTACCACATCCAAGGAAGGCAGCAGGCACGCAAATTACCCACTCCCAGATCGGGGAGGTAGTGACGAAAAATAACGGAACGTTTCTCCATGAGACGCGTTACCGGAACGATCGAGCCGTACATAAGTTCGGCTAAATCTATTGGAGGGCAAGTCTGGTGCCAGCAGCCGCGGTAATTCCAGCTCCAATAGCGTATATTAAAGTTGCTGCGGTTAAACCGCTCGTAGTTGGATTGCGGATGTCGACGACGGTCGTCCTAAGC------------------------------------------------------------------------------------------------------------------------------------------------------------------------------------------------------------------------------------------------------------------------------------------

>HVR-I_Hap_XVI

CAGTC-GCGCGCTGCGCG-ACGGAGCGC-GTTTATT-TGAACAAAACCAATCGGACGCAGGCTAG--CTATTGGTCTGAGTCCGCGAAATGTTGGTGAATCGGAATAACTATGCTGATCGCACGGTCCAGTACCGGCGACGATGCTTTGAAACGACTTGCTCATCAACTTTCGATGGTACGCTACGTGCTTACCATGGTGACAACGGTTAACGGAGAATCAGGGTTCGGCTCCGGAGAGGGAGCCTGAGAAACGGCTACCACATCCAAGGAAGGCAGCAGGCACGCAAATTACCCACTCCCAGATCGGGGAGGTAGTGACGAAAAATAACGGAACGTTTCTCCATGAGACGCGTTACCGGAACGATCGAGCCGTACATAAGTTCAGCTAAATCTATTGGAGGGAAAGTCTGGTGCCACCAGCCGCGGTAATTCCGGCTCCAATAGCGTATATTAAAGTTGCTGCGGTTAAACCGCTCGTAGTTGGATTGCGGATGTAGACCACGGACTTCCTA---------------------------------------------------------------------------------------------------------------------------------------------------------------------------------------------------------------------------------------------------------------------------------------------

>HVR-I_Hap_XVII

CAGTC-GCGCGCTGCGCG-ACGGAGCGC-GTTTATT-TGAACAAAACCAATCGGACGCAGGCTAG--CTATTGGTCTGAGTCCGCGAAATGTTGGTGAATCGGAATAACTATGCTGATCGCACGGTCCAGTACCGGCGACGATGCTTTGAAACGACTTGCTCATCAACTTTCGATGGTACGCTACGTGCTTACCATGGTGACAACGGTTAACGGAGAATCAGGGTTCGGCTCCGGAGAGGGAGCCTGAGAAACGGCTACCACATCCAAGGAAGGCAGCAGGCACGCAAATTACCCACTCCCAGATCGGGGAGGTAGTGACGAAAAATAACGGAACGTTTCTCCATGAGACGCGTTACCGGAACGATCGAGCCGTACATAAGTTCGGCTAAATCTATTGGAGGGCAAGTCTGGTGCCAGCAGCCGCGGTAATTCCAGCTCCAATAGCGTATATTAAAGTTGCTGCGGTTAAACCGCTCGTAGTTGGATTGCGGATGTCGACGACTGTCGTCCTAAGCAAGAGTC-----------------------------------------------------------------------------------------------------------------------------------------------------------------------------------------------------------------------------------------------------------------------------------

>HVR-I_Hap_XVIII

CAGTC-GCGCACTGCGCGT-CGGAGCGC-GTTTATT-TGAACAAAACCAATCGGGCGCAGGCTAG--CTATTGGCCTGAGTCCGCAAAATATTGGTGAATCGGAATAACTATGCTGATCGCACGGTCCAGTACCGGCGACGATACTTTGAAACGACTTGCTCATCAACTTTCGATGGTACGCTACGTGCTTACCATGGTGACAACGGTTAACGGAGAATCAGGGTTCGGCTCCGGAGAGGGAGCCTGAGAAACGGCTACCACATCCAAGGAAGGCAGCAGGCACGCAAATTACCCACTCCCAGATCGGGGAGGTAGTGACGAAAAATAACGGAACGTTTCTCCATGAGACGCGTTACCGGAACGATCGAGCCGTACATAAGTTCGGCTAAATCTATTGGAGGGCAAGTCTGGTGCCAGCAGCCGCGGTAATTCCAGCTCCAATAGCGTATATTAAAGTTGCTGCGGTTAAACCGCTCGTAGTTGGATTGCGGATGTCGACGACGGTCGTCCTAAGCAGGAGTCGTTCCGTCGCTCGTCACCTGTTCGATCAAGATTGCCCTGGATGCTCTTCAGTGAGTGTCCTTGGCGACTTGAAAGTTTACTTTGAGAAAATGAGAGCGCTCAAGGCAAGCCGTAGTGCTTGAACCGTGGTGCATGGAATAATGAAAGATGGCCTCAGTGCTATTTTGTT--------GGTTTACGGCTATGAGGCAATGATTAAAAGAGACTGACGGGGACATTCGTATTGCTGCGTTAGAGGTGAAATTCTTGGATCGCAGCAAGACGACCAAT

>HVR-I_Hap_XIX

CAGTC-GCGCGCTGCGCGT-CGGAGCGC-GTTTATT-TGAACAAAACCAATCGGACGCAGGCTAG--CTATTGGTCTGAGTCCGCGAAATGTTGGTGAATCGGAATAACTATGCTGATCGCACGGTCCAGTACCGGCGACGATGCTTTGAAACGACTTGCTCATCAACTTTCGATGGTACGCTACGTGCTTACCATGGTGACAACGGTTAACGGAGAATCAGGGTTCGGCTCCGGAGAGGGAGCCTGAGAAACGGCTACCACATCCAAGGAAGGCAGCAGGCACGCAAATTACCCACTCCCAGATCGGGGAGGTAGTGACGAAAAATAACGGAACGTTTCTCCATGAGACGCGTTACCGGAACGATCGAGCCGCACATAAGTTCGGCTAAATCTATTGGAGGGCAAGTCTGGTGCCAGCAGCCGCGGTAATTCCAGCTCCAATAGCGTATATTAAAGTTGCTGCGGTTAAACCGCTCGTAGTTGGATTGCGGATGTCGACGACGGTCGTCCTAAGCAGGAGTCGTTCCGTCGCTCGTCACCTGTTCGATCAAGATTGCCCTGGATGCTCTTCAGTGAGTGTCCTTGGCGACTTGAAAGTTTACTTTGAGAAAATGAGAGCGCTCAAGGCAAGCCGTAGTGCTTGAACCGTGGTGCATGGAATAATGAAAGATGGCCTCAGTGCTATTTTGTT--------GGTTTACGGCTATGAGGCAATGATTAAAAGAGACTGACGGGGACATTCGTATTGCTGCGTTAGAGGTGAAATTCTTGGATCGCAGCAAGACGACCAAT

>HVR-I_Hap_XX

CAGTC-GCGCGCTGCGCGT-CGGAGCGC-GTTTATT-TGAACAAAACCAATCGGACGCAGGCTAG--CTATTGGTCTGAGTCCGCGAAATGTTGGTGAATCGGAATAACTATGCTGATCGCACGGTCCAGTACCGGCGACGATGCTTTGAAACGACTTGCTCATCAACTTTCGATGGTACGCTACGTGCTTACCATGGTGACAACGGTTAACGGAGAATCAGGGTTCGGCTCCGGAGAGGGAGCCTGAGAAACGGCTACCACATCCAAGGAAGGCAGCAGGCACGCAAATTACCCACTCCCAGATCGGGGAGGTAGTGACGAAAAATAACGGAACGTTTCTCCATGAGACGCGTTACCGGAACGATCGAGCCGTACATAAGTTCGGCTAAATCTATTGGAGGGCAAGTCTGGTGCCAGCAGCCGCGGTAATTCCAGCTCCAATAGCGTATATTAAAGTTGCTGCGGTTAAACCGCTCGTAGTTGGATTGCGGATGTCGACGACGGTCGTCCTAAGCAGGAGTCGTTCCGTCGCTCGTCACCTGTTCGATCAAGATTGCCCTGGATGCTCTTCAGTGAGTGTCCTTGGCGACTTGAAAGTTTACTTTGAGAAAATGAGAGCGCTCAAGGCAAGCCGTAGTGCTTGAACCGTGGTGCATGGAATAATGAAAGATGGCCTCAGTGCTATTTTGTT--------GGTTTACGGCTATGAGGCAATGATTAAAAGAGACTGACGGGGACATTCGTATTGCTGCGTTAGAGGTGAAATTCTTGGATCGCGGCAAGACGACCAAT

>HVR-I_Hap_XXI

CAGTC-GCGCGCTGCGCGT-CGGAGCGC-GTTTATT-TGAACAAAACCAATCGGACGCAGGCTAG--CTATTGGTCTGAGTCCGCGAAATGTCGGTGAATCGGAATAACTATGCTGATCGCACGGTCCAGTACCGGCGACGATGCTTTGAAACGACTTGCTCATCAACTTTCGATGGTACGCTACGTGCTTACCATGGTGACAACGGTTAACGGAGAATCAGGGTTCGGCTCCGGAGAGGGAGCCTGAGAAACGGCTACCACATCCAAGGAAGGCAGCAGGCACGCAAATTACCCACTCCCAGATCGGGGAGGTAGTGACGAAAAATAACGGAACGTTTCTCCATGAGACGCGTTACCGGAACGATCGAGCCGCACATAAGTTCGGCTAAATCTATTGGAGGGCAAGTCTGGTGCCAGCAGCCGCGGTAATTCCAGCTCCAATAGCGTATATTAAAGTTGCTGCGGTTAAACCGCTCGTAGTTGGATTGCGGATGTCGACGACGGTCGTCCTAAGCAGGAGTCGTTCCGTCGCTCGTCACCTGTTCGATCAAGATTGCCCTGGATGCTCTTCAGTGAGTGTCCTTGGCGACTTGAAAGTTTACTTTGAGAAAATGAGAGCGCTCAAGGCAAGCCGTAGTGCTTGAACCGTGGTGCATGGAATAATGAAAGATGGCCTCAGTGCTATTTTGTT--------GGTTTACGGCTATGAGGCAATGATTAAAAGAGACTGACGGGGACATTCGTATTGCTGCGTTAGAGGTGAAATTCTTGGATCGCAGCAAGACGACCAAT

>HVR-I_Hap_XXII

CAGTC-GCGCGCTGCGCG-ACGGAGCGC-GTTTATT-TGAACAAAACCAATCGGACGCAGGCTAG--CTATTGGTCTGAGTCCGCGAAATGTTGGTGAATCGGAATAACTATGCTGATCGCACGGTCCAGTACCGGCGACGATGCTTTGAAACGACTTGCTCATCAACTTTCGATGGTACGCTACGTGCTTACCATGGTGACAACGGTTAACGGAGAATCAGGGTTCGGCTCCGGAGAGGGAGCCTGAGAAACGGCTACCACATCCAAGGAAGGCAGCAGGCACGCAAATTACCCACTCCCAGATCGGGGAGGTAGTGACGAAAAATAACGGAACGTTTCTCCATGAGACGCGTTACCGGAACGATCGAGCCGTACATAAGTTCGGCTAAATCTATTGGAGGGCAAGTCTGGTGCCAGCAGCCGCGGTAATTCCAGCTCCAATAGCGTATATTAAAGTTGCTGCGGTTAAACCGCTCGTAGTTGGATTGCGGATGTCGACGACGGTCGTCCTAAGCAGGAGTCGTTCCGTCGCTCGTCACCTGTTCGATCAAGATTGCCCTGGATGCTCTTCAGTGAGTGTCCTTGGCGACTTGAAAGTTTACTTTGAGAAAATGAGAGCGCTCAAGGCAAGCCGTAGTGCTTGAACCGTGGTGCATGGAATAATGAAAGATGGCCTCAGTGCTATTTTGTT--------GGTTTACGGCTACGAGGCAATGATTAAAAGAGACTGACGGGGACATTCGTATTGCTGCGTTAGAGGTGAAATTCTTGGATCGCAGCAAGACGACCAAT

>HVR-I_Hap_XXIII

CAGTC-GCGCGCTGCGCG-ACGGAGCGC-GTTTATT-TGAACAAAACCAATCGGACGCAGGCTAG--CTATTGGTCTGAGTCCGCGAAATTTTGGTGAATCGGAATAACTATGCTGATCGCACGGTCCAGTACCGGCGACGATGCTTTGAAACGACTTGCTCATCAACTTTCGATGGTACGCTACGTGCTTACCATGGTGACAACGGTTAACGGAGAATCAGGGTTCGGCTCCGGAGAGGGAGCCTGAGAAACGGCTACCACATCCAAGGAAGGCAGCAGGCACGCAAATTACCCACTCCCAGATCGGGGAGGTAGTGACGAAAAATAACGGAACGTTTCTCCATGAGACGCGTTACCGGAACGATCGAGCCGTACATAAGTTCGGCTAAATCTATTGGAGGGCAAGTCTGGTGCCAGCAGCCGCGGTAATTCCAGCTCCAATAGCGTATATTAAAGTTGCTGCGGTTAAACCGCTCGTAGTTGGATTGCGGATGTCGACGACGGTCGTCCTAAGCAGGAGTCGTTCCGTCGCTCGTCACCTGTTCGATCAAGATTGCCCTGGATGCTCTTCAGTGAGTGTCCTTGGCGACTTGAAAGTTTACTTTGAGAAAATGAGAGCGCTCAAGGCAAGCCGTAGTGCTTGAACCGTGGTGCATGGAATAATGAAAGATGGCCTCAGTGCTATTTTGTT--------GGTTTACGGCTATGAGGCAATGATTAAAAGAGACTGACGGGGACATTCGTATTGCTGCGTTAGAGGTGAAATTCTTGGATCGCAGCAAGACGACCAAT

>HVR-I_Hap_XXIV

TCGGTCGCGCGCAATGCGT-CGGAGCGCA-TTTATTA-GAACAAAACCAATCGGGCGCAGGCTAA--TTATTGGTCTGAGTCCGCGAAAGGTTGGCGAATCGGAATAACTATGCTGATCGCACGGTCCAGCACCGGCGACGAATCTTTGAAATGACTTGCTCATCAACTTTCGATGGTACGCTACGTGCTTACCATGGTGACAACGGTTAACGGAGAATCAGGGTTCGACTCCGGAGAGGGAGCCTGAGAAACGGCTACCACATCCAAGGAAGGCAGCAGGCACGCAAATTACCCACTCCCAGATCGGGGAGGTAGTGACGAAAAATAACGGAACGTATCTCCATGAGACGCGTTACCGGAACGACTGAGCCGTACACAAGCTCGGCTAAATCTATTGGAGGGCAAGTCTGGTGCCAGCAGCCGCGGTAATTCCAGCTCCAATAGCGTATATTAAAGTTGCTGCGGTTAAACCGCTCGTCGTTGGATTGCGGATGTCGACGACGGTCGTCCTAAGCAGGAGTCGTTCCGTCGCTCGTCACCTGTTCGATCAAGATTGCCTTGGATGCTCTTCAGTGAGTGTCCTTGGCGACTTGAAAGTTTACTTTGAGAAAATGGAAGCGCTCAAAGCAAGCCGTAGTGCTTGAACCGTGGTGCATGGAATAATGAAAGATGGCCTCGATGCTATTTTGTT--------GGTTTGCGGCTATGAGGCAATGATTAAAAGAGACAGACGGGGACATTCGTATTGCTGCGTTAGAGGTGAAATTCTTGGATCGCAGCAAGACGACCAAT

>HVR-I_Hap_TMUE

---TCGGCGCGCAATGCGG-CGGAGCGC-ATTTATT-GGTACAAAACCAATCGGACTTTCGCT----CTTTGCGTCCG--TCCGTCAACGGTTGGTGAATCGGAATAACTATGCTGATCGCACGGTCCAGCACCGGCGACGAATCTTTGAAATGACTTGCTCATCAACTTTCGATGGTACGCTACGTGCTTACCATGGTGACAACGGTTAACGGAGAATCAGGGTTCGACTCCGGAGAGGGAGCCTGAGAAACGGCTACCACATCCAAGGAAGGCAGCAGGCACGCAAATTACCCACTCCCAGATCGGGGAGGTAGTGACGAAAAATAACGGAACGTATCTCCATGAGACGCGTTACCGGAACGACCGAGCCGTACATAAGCTCGGCTAATTCTATTGGAGGGCAAGTCTGGTGCCAGCAGCCGCGGTAATTCCAGCTCCAATAGCGTATATTAAAGTTGCTGCGGTTAAACCGCTCGTAGTTGGATTTTGGTGCACGACGACGGTCGTCCTAAGCAGGAGTCGTTCTGTCGCTCGTCACCTGTTCGCTCAAGCTTGTCCTTGATGCTCTTGAGTGAGTGTCCTGGGCGACTTGAAAGTTTACTTTGAGAAAATGGAAGCGCTCAAGGCAGGCCGTAGTGCTTGAACAGTGGTGCATGGAATAATGAAAGATGGCCTCGGTGCTATTTTGTT--------GGTTTACGGTGACGAGGCAATGATTAAAAGAGACAGACGGGGACATTCGTATTGCTGCGTTAGAGGTGAAATTCTTGGATCGCAGCAAGACGAACAAT

#### 18S rDNA HVR-IV full-length haplotypes

>HVR-IV_Hap_A

TTAGTTGGTGGAGCGATTTGTCTGGCTAATTCCGATAACGAACGAGACTCTGGCCTACTAACTAGCGGCGGTATTCACGTCTCCCGCGCGACGCCGCGC-TGCGC-----TGCGGCTGAGCACA---------GTCGTCGCCGTTTGCGC-GAGCGCTC--CGCGGAGCACCTGCAGTGCCGGCAGCCGCTTCTTAGAGGGACCAGCGACACTTTCGCAAGCCGCACGAGAAAGAGCAATAACAGGTCTGTGATGCCCTTAGATGTACGGGGCTGCACGCGTGCTACACTGACGGCGTCAGCGTGCGTTCAAGCCCGGCCTGGCAAGGCCAGGAAATCGGTTGAAACGTTCTCGTGACTGGGACAGGGAATTGCAATTATTTCCCTCGAA

>HVR-IV_Hap_B

TTAGTTGGTGGAGCGATTTGTCTGGCTAATTCCGATAACGAACGAGACTCTGGCCTACTAACTAGCGGCGGTATTCACGTCTCCCGCGCGACGCCGCGC-TGCGC-----TGCGGCTGAGCACA---------GTCGTCGCCGTTTGCGC-GAGCGCTC--CGCGGAGCACCTGTAGTGCCGGCAGCCGCTTCTTAGAGGGACCAGCGACACTTTCGCAAGCCGCACGAGAAAGAGCAATAACAGGTCTGTGATGCCCTTAGATGTACGGGGCTGCACGCGTGCTACACTGACGGCGTCAGCGTGCGTTCAAGCCCGGCCTGGCAAGGCCAGGAAATCGGTTGAAACGTTCTCGTGACTGGGACAGGGAATTGCAATTATTTCCCTCGAA

>HVR-IV_Hap_C

TTAGTTGGTGGAGCGATTTGTCTGGCGAATTCCGATAACGAACGAGACTCTGGCCTACTAACTAGCGGCGGTATTCACGTCTCCCGCGCGACGCCGCGC-TGCGC-----TGCGGCTGAGCACA---------GTCGTCGCCGTTTGCGC-GAGCGCTC--CGCGGAGCACCTGCAGTGCCGGCAGCCGCTTCTTAGAGGGACCAGCGACACTTTCGCAAGCCGCACGAGAAAGAGCAATAACAGGTCTGTGATGCCCTTAGATGTACGGGGCTGCACGCGTGCTACACTGACGGCGTCAGCGTGCGTTCAAGCCCGGCCTGGCAAGGCCAGGAAATCGGTTGAAACGTTCTCGTGACTGGGACAGGGAATTGCAATTATTTCCCTCGAA

>HVR-IV_Hap_D

TTAGTTGGTGGAGCGATTTGTCTGGCTAATTCCGATAACGAACGAGACTCTGGCCTACTAACTAGCGGCGGTATTCACGTCTCCCGCGCGACGCCGCGC-TGCGC-----TGCGGCTGAGCACA---------GTCGTCGCCGTTTGCGC-GAGCGCTC--CGCGGAGCACCTGCAGTGCCGGCAGCCGCTTCTTAGAGGGACCAGCGACACTTTCGCAAGCCGCACGAGGAAGAGCAATAACAGGTCTGTGATGCCCTTAGATGTACGGGGCTGCACGCGTGCTACACTGACGGCGTCAGCGTGCGTTCAAGCCCGGCCTGGCAAGGCCAGGAAATCGGTTGAAACGTTCTCGTGACTGGGACAGGGAATTGCAATTATTTCCCTCGAA

>HVR-IV_Hap_E

TTAGTTGGTGGAGCGATTTGTCTGGCTAATTCCGATAACGAACGAGACTCTGGCCTACTAACTAGCGGCGGTATTCACGTCTCCCGCGCGACGCCGCGC-TGCGC-----TTCGGCTGAGCACA---------GTCGTCGCCGTTTGCGC-GAGCGCTC--CGCGGAGCACCTGTAGTGCCGGCAGCCGCTTCTTAGAGGGACCAGCGACACTTTCGCAAGCCGCACGAGAAAGAGCAATAACAGGTCTGTGATGCCCTTAGATGTACGGGGCTGCACGCGTGCTACACTGACGGCGTCAGCGTGCGTTCAAGCCCGGCCTGGCAAGGCCAGGAAATCGGTTGAAACGTTCTCGTGACTGGGACAGGGAATTGCAATTATTTCCCTCGAA

>HVR-IV_Hap_F

TTAGTTGGTGGAGCGATTTGTCTGGCTAATTCCGATAACGAACGAGACTCTGGCCTACTAACTAGGGCCGGTATTCACGTCTCCCGCGCGACGCCGCGC-TGCGC-----TGCGGCTGAGCACA---------GTCGTCGCCGTTTGCGC-GAGCGCTC--CGCGGAGCACCTGCAGTGCCGGCAGCCGCTTCTTAGAGGGACCAGCGACACTTTCGCAAGCCGCACGAGAAAGAGCAATAACAGGTCTGTGATGCCCTTAGATGTACGGGGCTGCACGCGTGCTACACTGACGGCGTCAGCGTGCGTTCAAGCCCGGCCTGGCAAGGCCAGGAAATCGGTTGAAACGTTCTCGTGACTGGGACAGGGAATTGCAATTATTTCCCTCGAA

>HVR-IV_Hap_G

TTAGTTGGTGGAGCGATTTGTCTGGCTAATTCCGATAACGAACGAGACTCTGGCCTACTAACTAGCGGCGGTATTCACGTCTCCCGCGCGACGCCGCGC-TGCGC-----TGCGGCTGAGCACA---------GTCGTCGCCGTTTGCGC-GAGCGCTC--CGCGGAGCACCTGCAGTGCCGGCAGCCGCTTCTTAGAGGGACCAGCGACACTTTCGCAAGCCGCACGAGAAAGAGCAATAACAGGTCTGTGATGCCCTTAGATGTACGGGGCTGCACGCGTGCTACACTGACGGCGACAGCGTGCGTTCAAGCCCGGCCTGGCAAGGCCAGGAAATCGGTTGAAACGTTCTCGTGACTGGGACAGGTAATTGCAATTATTTCCCTCGAA

>HVR-IV_Hap_H

TTAGTTGGTGGAACGATTTGTCTGGCTAATTCCGATATCGAACGAGACTCTGGCCTACTAACTAGCGGCGGTATT-ACGTCTCCCGCGCGACGCCGCGC-TGCGC-----TGCGGCTGAGCACA---------GTCGTCGCCGTTTGCGC-GAGCGCTC--CGCGGAGCACCTGCAGTGCCGGCAGCCGCTTCTTAGAGGGACCAGCGACACTTTCGCAAGCCGCACGAGAAAGAGCAATAACAGGTCTGTGATGCCCTTAGATGTACGGGGCTGCACGCGTGCTACACTGACGGCGTCAGCGTGCGTTCAAGCCCGGCCTGGCAAGGCCAGGAAATCGGTTGAAACGTTCTCGTGACTGGGACAGGGAATTGCAATTATTTCCCTCGAA

>HVR-IV_Hap_I

TTAGTTGGTGGAGCGATTTGTCTGGCTAATTCCGATAACGAACGAGACTCTGGCCTACTAACTAGCGGCGGTATTCACGTCTCCCGCGCGACGCCGCGC-TGCGTCACGGCGCGGCTGAACACAGTCGTCGTCGTCGTCGCCGTTTGCGC-GAGCGCTC--CGCGGAGCACTTGCAGTGCCGGTAGCCGCTTCTTAGAGGGACCAGCGACACTTTCGCAAGCCGCACGAGAAAGAGCAATAACAGGTCTGTGATGCCCTTAGATGTACGGGGCTGCACGCGTGCTACACTGACGGCGTCAGCGTGCGTTCAAGCCCGGCCTGGCAAGGCCAGGAAATCGGTTGAAACGTTCTCGTGACTGGGACAGGGAATTGCAATTATTTCCCTCGAA

>HVR-IV_Hap_J

TTAGTTGGTGGAGCGATTTGTCTGGCTAATTCCGATAACGAACGAGACTCTGGCCTACTAACTAGCGGCGGTATTCACGTCTCCTGCGCGACGCCGCGC-TGCGTCACGGCGCGGCTGAACACAGTCGTCGTCGTCGTCGCCGTTTGCGC-GAGCGCTC--CGCGGAGCACTTGCAGTGCCGGTAGCCGCTTCTTAGAGGGACCAGCGACACTTTCGCAAGCCGCACGAGAAAGAGCAATAACAGGTCTGTGATGCCCTTAGATGTACGGGGCTGCACGCGTGCTACACTGACGGCGTCAGCGTGCGTTCAAGCCCGGCCTGGCAAGGCCAGGAAATCGGTTGAAACGTTCTCGTGACTGGGACAGGGAATTGCAATTATTTCCCTCGAA

>HVR-IV_Hap_K

TTAGTTGGTGGAGCGATTTGTCTGGCTAATTCCGATAACGAACGAGACTCTGGCCTACTAACTAGCGGCGGTATTCACGTCTGCCGCGCGACGCCGCGC-TGCGTCACGGCGCGGCTGAGCACAGTCGTCGTCGTCGTCGCCGTTTGCGC-GAGCGCTC--CGCGGAGCACTTGCAGTGCCGGCAGCCGCTTCTTAGAGGGACCAGCGACACTTTCGCAAGCCGCACGAGAAAGAGCAATAACAGGTCTGTGATGCCCTTAGATGTACGGGGCTGCACGCGTGCTACACTGACGGCGTCAGCGTGCGTTCAAGCCCGGCCTGGCAAGGCCAGGAAATCGGTTGAAACGTTCTCGTGACTGGGACAGGGAATTGCAATTATTTCCCTCGAA

>HVR-IV_Hap_L

TTAGTTGGTGGAGCGATTTGTCTGGCTAATTCCGATAACGAACGAGACTCTGGCCTACTAACTAGCGGCGGCATTCACGTCTCCCGCGCGACGCCGTGCTTACGC-----GTCGGCTGAGCACA------------GTCACCGTTCGCCC-GAGCGCTCCGCGCGGAGCACCTGCAGTGCCGGCGGCCGCTTCTTAGAGGGACCAGCGACACTTTTGCAAGCCGCACGAGAAAGAGCAATAACAGGTCTGTGATGCCCTTAGATGTACGGGGCTGCACGCGTGCTACACTGACGGCGTCAGCGTGCGTTCAAGCCCGGCCTGGCAAGGCCGGGAAATCGGCTGAAACGTTCTCGTGACTGGGACAGGGAATTGCAACTATTTCCCTCGAA

>HVR-IV_Hap_TMUE

TTAGTTGGTGGAGCGATTTGTCTGGCTAATTCCGATAACGAACGAGACTCTGGCCTACTAACTAGCGGCGGTGTTCATGTCTCCTGTATG--GCCGCA------------GACGGTTGCGCGC-----------TCGTCGTTACAACCGCTGCGCGCTC--TATGGAGCA---GCAGCGCCGGCAGCCGCTTCTTAGAGGGACCAGCGACACTTTCGCAAGCCGCACGAGAAAGAGCAATAACAGGTCTGTGATGCCCTTAGATGTACGGGGCTGCACGCGTGCTACACTGACGGCGTCAGCGTGCGTTCAAGCCCGGCCTGCCAAGGTCGGGAAATCGGTTGAAACGTTCTCGTGACTGGGACAGGGAATTGAAATTATTTCCCTCGAA

## *Trichuris* ITS1 and ITS2 sequences used to construct haplotype maps

#### ITS1 reference sequences

>Worm_10-5-10_ITS1

GGCGTTTGCTCCGTCCGATGCGAGCAACGTCCGCCGGCTTACAGTTCCAGGTCTGAAGAGCGCAGGCGGCGGCTCGTTCC

GCCCGGCGGGAAGCGAGTCGATCGTCGCCGTCATCGTCTCGATCGGGCAGCAGCAGCGGTTTTCACTTGCTGCTCGCCGC

CTGCGCGCAGTCACCGGTACCT

>Worm_3-4-1_ITS1

GGCGTTTGCTCGGTCCGATGCGAGCAACGTCCGCCGGCTTACAGTTCCAGGTCTGAAGAGCGCAGGCGGCGGCTCGTTCC

GCCCGGCGGGCGAGTCGTCGATCGTCGCCGTCACCGGCTCGATCAGGCAGCAGCAATTTTCACTTGCTGCTCGCCGCCTG

CGCGCAGTCACCGGTACCT

>Worm_3-4-2_ITS1

GGCGTTTGCTCGGTCCGATGCGAGCAACGTCCGCCGGCCTACAGTTCCAGGTCTGAAGAGCGCAGGCGGCGGCGGCTCGT

TCCGCCCGGCGGGCGAGTCGTCGATCGTCGCCGTCACCGGCTCGATCAGGCAGCAGCGGTTTTCACTTGCTGCTCGCCGC

CTGCGCGCAGTCACCGGTACCT

>Worm_3-4-3_ITS1_1

GGCGTTTGCTCCGTCCGATGCGAGCAACGTCCGCCGGCTTACAGTTCCAGGTCTGAAGAGCGCAGGCGGCGGCTCGTTCC

GCCCGGCGGGAAGCGAGTCGATCGTCGCCGTCACCGGCTCGATCGGGCAGCAGCAGCGGTTTTCACTTGCTGCTCGCCGC

CTGCGCGCAGTCACCGGTACCT

>Worm_3-4-3_ITS1_2

GGCGTTTGCTCCGTCCGATGCGAGCAACGTCCGCCGGCTTACAGTTCCAGGTCTGAAGAGCGCAGGCGGCGGCTCGTTCC

GCCCGGCGGGAAGCGAGTCGATCGTCGCCGTCACCGGCTCGATCGGGCAGCAGCAGCGGTTTTCACTTGCTGCTCGCCGC

CTGCGCGCAGTCACCGGTACCT

>Worm_3-4-4_ITS1

GGCGTTTGCTCCGTCCGATGCGAGCAACGTCCGCCGGCTTACAGTTCCAGGTCTGAAGAGCGCAGGCGGCGGCTCGTTCC

GCCCGGCGGGAAGCGAGTCGATCGTCGCCGTCACCGGCTCGATCGGGCAGCAGCAGCGGTTTTCACTTGCTGCTCGCCGC

CTGCGCGCAGTCACCGGTACCT

>Worm_3-4-5_ITS1

GGCGTTTGCTCGGTCCGATGCGAGCAACGTCCGCCGGCCTACAGTTCCAGGTCTGAAGAGCGCAGGCGGCGGCGGCTCGT

TCCGCCCGGCGGGCGAGTCGTCGATCGTCGCCGTCACCGGCTCGATCAGGCAGCAGCGGTTTTCACTTGCTGCTCGCCGC

CTGCGCGCAGTCACCGGTACCT

>Worm_3-4-7_ITS1_1

GGCGTTTGCTCCGTCCGATGCGAGCAACGTCCGCCGGCTTACAGTTCCAGGTCTGAAGAGCGCAGGCGGCGGCTCGTTCC

GCCCGGCGGGAAGCGAGTCGATCGTCGCCGTCACCGGCTCGATCGGGCAGCAGCAGCGGTTTTCACTTGCTGCTCGCCGC

CTGCGCGCAGTCACCGGTACCT

>Worm_3-4-7_ITS1_2

GGCGTTTGCTCGGTCCGATGCGAGCAACGTCCGCCGGCCTACAGTTCCAGGTCTGAAGAGCGCAGGCGGCGGCGGCTCGT

TCCGCCCGGCGGGCGAGTCGTCGATCGTCGCCGTCACCGGCTCGATCAGGCAGCAGCGGTTTTCACTTGCTGCTCGCCGC

CTGCGCGCAGTCACCGGTACCT

>Worm_3-4-9_ITS1_1

GGCGTTTGCTCGGTCCGATGCGAGCAACGTCCGCCGGCTTACAGTTCCAGGTCTGAAGAGCGCAGGCGGCGGCTCGTTCC

GCCCGGCGGGCGAGTCGTCGATCGTCGCCGTCACCGGCTCGATCAGGCAGCAGCAATTTTCACTTGCTGCTCGCCGCCTG

CGCGCAGTCACCGGTACCT

>Worm_3-4-9_ITS1_2

GGCGTTTGCTCGGTCCGATGCGAGCAACGTCCGCCGGCCTACAGTTCCAGGTCTGAAGAGCGCAGGCGGCGGCGGCTCGT

TCCGCCCGGCGGGCGAGTCGTCGATCGTCGCCGTCACCGGCTCGATCAGGCAGCAGCGGTTTTCACTTGCTGCTCGCCGC

CTGCGCGCAGTCACCGGTACCT

>Worm_3-4-10_ITS1_1

GGCGTTTGCTCGGTCCGATGCGAGCAACGTCCGCCGGCCTACAGTTCCAGGTCTGAAGAGCGCAGGCGGCGGCGGCTCGT

TCCGCCCGGCGGGCGAGTCGTCGATCGTCGCCGTCACCGGCTCGATCAGGCAGCAGCGGTTTTCACTTGCTGCTCGCCGC

CTGCGCGCAGTCACCGGTACCT

>Worm_3-4-10_ITS1_2

GGCGTTTGCTCGGTCCGATGCGAGCAACGTCCGCCGGCTTACAGTTCCAGGTCTGAAGAGCGCAGGCGGCGGCTCGTTCC

GCCCGGCGGGCGAGTCGTCGATCGTCGCCGTCACCGGCTCGATCAGGCAGCAGCAATTTTCACTTGCTGCTCGCCGCCTG

CGCGCAGTCACCGGTACCT

>Worm_3-4-11_ITS1_1

GGCGTTTGCTCGGTCCGATGCGAGCAACGTCCGCCGGCCTACAGTTCCAGGTCTGAAGAGCGCAGGCGGCGGCGGCTCGT

TCCGCCCGGCGGGCGAGTCGTCGATCGTCGCCGTCACCGGCTCGATCAGGCAGCAGCGGTTTTCACTTGCTGCTCGCCGC

CTGCGCGCAGTCACCGGTACCT

>Worm_3-4-11_ITS1_2

GGCGTTTGCTCGGTCCGATGCGAGCAACGTCCGCCGGCCTACAGTTCCAGGTCTGAAGAGCGCAGGCGGCGGCGGCTCGT

TCCGCCCGGCGGGCGAGTCGTCGATCGTCGCCGTCACCGGCTCGATCAGGCAGCAGCGGTTTTCACTTGCTGCTCGCCGC

CTGCGCGCAGTCACCGGTACCT

>Worm_3-4-12_ITS1

GGCGTTTGCTCGGTCCGATGCGAGCAACGTCCGCCGGCTTACAGTTCCAGGTCTGAAGAGCGCAGGCGGCGGCTCGTTCC

GCCCGGCGGGCGAGTCGTCGATCGTCGCCGTCACCGGCTCGATCAGGCAGCAGCAATTTTCACTTGCTGCTCGCCGCCTG

CGCGCAGTCACCGGTACCT

>Worm_3-4-13_ITS1_1

GGCGTTTGCTCCGTCCGATGCGAGCAACGTCCGCCGGCTTACAGTTCCAGGTCTGAAGAGCGCAGGCGGCGGCTCGTTCC

GCCCGGCGGGAAGCGAGTCGATCGTCGCCGTCATCGTCTCGATCGGGCAGCAGCAGCGGTTTTCACTTGCTGCTCGCCGC

CTGCGCGCAGTCACCGGTACCT

>Worm_3-4-13_ITS1_2

GGCGTTTGCTCGGTCCGATGCGAGCAACGTCCGCCGGCTTACAGTTCCAGGTCTGAAGAGCGCAGGCGGCGGCTCGTTCC

GCCCGGCGGGCGAGTCGTCGATCGTCGCCGTCACCGGCTCGATCAGGCAGCAGCAATTTTCACTTGCTGCTCGCCGCCTG

CGCGCAGTCACCGGTACCT

>Worm_3-4-13_ITS1_3

GGCGTTTGCTCGGTCCGATGCGAGCAACGTCCGCCGGCCTACAGTTCCAGGTCTGAAGAGCGCAGGCGGCGGCGGCTCGT

TCCGCCCGGCGGGCGAGTCGTCGATCGTCGCCGTCACCGGCTCGATCAGGCAGCAGCGGTTTTCACTTGCTGCTCGCCGC

CTGCGCGCAGTCACCGGTACCT

>Worm_6-1-2_ITS1_1

GGCGTTTGCTCCGTCCGATGCGAGCAACGTCCGCCGGCTTACAGTTCCAGGTCTGAAGAGCGCAGGCGGCGGCTCGTTCC

GCCCGGCGGGAAGCGAGTCGATCGTCGCCGTCACCGTCTCGATCGGGCAGCAGCAGCGGTTTTCACTTGCTGCTCGCCGC

CTGCGCGCAGTCACCGGTACCT

>Worm_6-1-2_ITS1_2

GGCGTTTGCTCCGTCCGATGCGAGCAACGTCCGCCGGCTTACAGTTCCAGGTCTGAAGAGCGCAGGCGGCGGCTCGTTCC

GCCCGGCGGGAAGCGAGTCGATCGTCGCCGTCACCGTCTCGATCGGGCAGCAGCAGCGGTTTTCACTTGCTGCTCGCCGC

CTGCGCGCAGTCACCGGTACCT

>Worm_6-1-4_ITS1

GGCGTTTGCTCGGTCCGATGCGAGCAACGTCCGCCGGCCTACAGTTCCAGGTCTGAAGAGCGCAGGCGGCGGCGGCTCGT

TCCGCCCGGCGGGCGAGTCGTCGATCGTCGCCGTCACCGGCTCGATCAGGCAGCAGCGGTTTTCACTTGCTGCTCGCCGC

CTGCGCGCAGTCACCGGTACCT

>Worm_6-1-6_ITS1_1

GGCGTTTGCTCGGTCCGATGCGAGCAACGTCCGCCGGCCTACAGTTCCAGGTCTGAAGAGCGCAGGCGGCGGCGGCTCGT

TCCGCCCGGCGGGCGAGTCGTCGATCGTCGCCGTCACCGGCTCGATCAGGCAGCAGCGGTTTTCACTTGCTGCTCGCCGC

CTGCGCGCAGTCACCGGTACCT

>Worm_6-1-6_ITS1_2

GGCGTTTGCTCGGTCCGATGCGAGCAACGTCCGCCGGCTTACAGTTCCAGGTCTGAAGAGCGCAGGCGGCGGCGGCTCGT

TCCGCCCGGCGGGCGAGTCGTCGATCGTCGCCGTCACCGGCTCGATCAGGCAGCAGCGGTTTTCACTTGCTGCTCGCCGC

CTGCGCGCAGTCACCGGTACCT

>Worm_6-1-8_ITS1_1

GGCGTTTGCTCGGTCCGATGCGAGCAACGTCCGCCGGCTTACAGTTCCAGGTCTGAAGAGCGCAGGCGGCGGCTCGTTCC

GCCCGGCGGGCGAGTCGTCGATCGTCGCCGTCACCGGCTCGATCAGGCAGCAGCAATTTTCACTTGCTGCTCGCCGCCTG

CGCGCAGTCACCGGTACCT

>Worm_6-1-8_ITS1_2

GGCGTTTGCTCCGTCCGATGCGAGCAACGTCCGCCGGCTTACAGTTCCAGGTCTGAAGAGCGCAGGCGGCGGCTCGTTCC

GCCCGGCGGGAAGCGAGTCGATCGTCGCCGTCACCGTCTCGATCGGGCAGCAGCAGCGGTTTTCACTTGCTGCTCGCCGC

CTGCGCGCAGTCACCGGTACCT

>Worm_6-1-10_ITS1

GGCGTTTGCTCCGTCCGATGCGAGCAACGTCCGCCGGCTTACAGTTCCAGGTCTGAAGAGCGCAGGCGGCGGCTCGTTCC

GCCCGGCGGGAAGCGAGTCGATCGTCGCCGTCACCGTCTCGATCGGGCAGCAGCAGCGGTTTTCACTTGCTGCTCGCCGC

CTGCGCGCAGTCACCGGTACCT

>Worm_10-5-1_ITS1

GGCGTTTGCTCGGTCCGATGCGAGCAACGTCCGCCGGCTTACAGTTCCAGGTCTGAAGAGCGCAGGCGGCGGCTCGTTCC

GCCCGGCGGGCGAGTCGTCGATCGTCGCCGTCACCGGCTCGATCAGGCAGCAGCAATTTTCACTTGCTGCTCGCCGCCTG

CGCGCAGTCACCGGTACCT

>Worm_10-5-2_ITS1

GGCGTTTGCTCGGTCCGATGCGAGCAACGTCCGCCGGCTTACAGTTCCAGGTCTGAAGAGCGCAGGCGGCGGCTCGTTCC

GCCCGGCGGGCGAGTCGTCGATCGTCGCCGTCACCGGCTCGATCAGGCAGCAGCAATTTTCACTTGCTGCTCGCCGCCTG

CGCGCAGTCACCGGTACCT

>Worm_10-5-3_ITS1_1

GGCGTTTGCTCGGTCCGATGCGAGCAACGTCCGCCGGCTTACAGTTCCAGGTCTGAAGAGCGCAGGCGGCGGCTCGTTCC

GCCCGGCGGGCGAGTCGTCGATCGTCGCCGTCACCGGCTCGATCAGGCAGCAGCAATTTTCACTTGCTGCTCGCCGCCTG

CGCGCAGTCACCGGTACCT

>Worm_10-5-3_ITS1_2

GGCGTTTGCTCCGTCCGATGCGAGCAACGTCCGCCGGCTTACAGTTCCAGGTCTGAAGAGCGCAGGCGGCGGCTCGTTCC

GCCCGGCGGGAAGCGAGTCGATCGTCGCCGTCATCGTCTCGATCGGGCAGCAGCAGCGGTTTTCACTTGCTGCTCGCCGC

CTGCGCGCAGTCACCGGTACCT

>Worm_10-5-4_ITS1

GGCGTTTGCTCCGTCCGATGCGAGCAACGTCCGCCGGCTTACAGTTCCAGGTCTGAAGAGCGCAGGCGGCGGCTCGTTCC

GCCCGGCGGGAAGCGAGTCGATCGTCGCCGTCATCGACTCGATCGGGCAGCAGCAGCGGTTTTCATTTGCTGCTCGCCGC

CTGCGCGCAGTCACCGGTACCT

>Worm_10-5-6_ITS1_1

GGCGTTTGCTCGGTCCGATGCGAGCAACGTCCGCCGGCTTACAGTTCCAGGTCTGAAGAGCGCAGGCGGCGGCTCGTTCC

GCCCGGCGGGCGAGTCGTCGATCGTCGCCGTCACCGGCTCGATCAGGCAGCAGCAATTTTCACTTGCTGCTCGCCGCCTG

CGCGCAGTCACCGGTACCT

>Worm_10-5-6_ITS1_2

GGCGTTTGCTCGGTCCGATGCGAGCAACGTCCGCCGGCCTACAGTTCCAGGTCTGAAGAGCGCAGGCGGCGGCGGCTCGT

TCCGCCCGGCGGGCGAGTCGTCGATCGTCGCCGTCACCGGCTCGATCAGGCAGCAGCGGTTTTCACTTGCTGCTCGCCGC

CTGCGCGCAGTCACCGGTACCT

>Worm_10-5-7_ITS1

GGCGTTTGCTCCGTCCGATGCGAGCAACGTCCGCCGGCTTACAGTTCCAGGTCTGAAGAGCGCAGGCGGCGGCTCGTTCC

GTCCGGCGGGAAGCGAGTCGATCGTCGCCGTCACCGTCTCGATCGGGCAGCAGCAGCGGTTTTCACTTGCTGCTCGCCGC

CTGCGCGCAGTCACCGGTACCT

>Worm_10-5-9_ITS1_1

GGCGTTTGCTCCGTCCGATGCGAGCAACGTCCGCCGGCTTACAGTTCCAGGTCTGAAGAGCGCAGGCGGCGGCTCGTTCC

GCCCGGCGGGAAGCGAGTCGATCGTCGCCGTCATCGACTCGATCGGGCAGCAGCAGCGGTTTTCATTTGCTGCTCGCCGC

CTGCGCGCAGTCACCGGTACCT

>Worm_10-5-9_ITS1_2

GGCGTTTGCTCGGTCCGATGCGAGCAACGTCCGCCGGCTTACAGTTCCAGGTCTGAAGAGCGCAGGCGGCGGCTCGTTCC

GCCCGGCGGGCGAGTCGTCGATCGTCGCCGTCACCGGCTCGATCAGGCAGCAGCAATTTTCACTTGCTGCTCGCCGCCTG

CGCGCAGTCACCGGTACCT

>Worm_10-5-9_ITS1_3

GAGCCAGGCTCCGTCCGATGCGAGCAACGTCCGCCGGCTTACAGTTCCAGGTCTGAAGAGCGCAGGCGGCGGCTCGTTCC

GCCCGGCGGGAAGCGAGTCGATCGTCGCCGTCATCGACTCGATCGGGCAGCAGCAGCGGTTTTCATTTGCTGCTCGCCGC

CTGCGCGCAGTCACCGGTACCT

>Worm_10-5-11_ITS1

GGCGTTTGCTCGGTCCGATGCGAGCAACGTCCGCCGGCTTACAGTTCCAGGTCTGAAGAGCGCAGGCGGCGGCTCGTTCC

GCCCGGCGGGCGAGTCGTCGATCGTCGCCGTCACCGGCTCGATCAGGCAGCAGCAATTTTCACTTGCTGCTCGCCGCCTG

CGCGCAGTCACCGGTACCT

>Worm_10-5-12_ITS1_1

GGCGTTTGCTCGGTCCGATGCGAGCAACGTCCGCCGGCCTACAGTTCCAGGTCTGAAGAGCGCAGGCGGCGGCGGCTCGT

TCCGCCCGGCGGGCGAGTCGTCGATCGTCGCCGTCACCGGCTCGATCAGGCAGCAGCGGTTTTCACTTGCTGCTCGCCGC

CTGCGCGCAGTCACCGGTACCT

>Worm_10-5-12_ITS1_2

GGCGTTTGCTCGGTCCGATGCGAGCAACGTCCGCCGGCTTACAGTTCCAGGTCTGAAGAGCGCAGGCGGCGGCTCGTTCC

GCCCGGCGGGCGAGTCGTCGATCGTCGCCGTCACCGGCTCGATCAGGCAGCAGCAATTTTCACTTGCTGCTCGCCGCCTG

CGCGCAGTCACCGGTACCT

>Worm_10-5-12_ITS1_3

GGCGTTTGCTCCGTCCGATGCGAGCAACGTCCGCCGGCTTACAGTTCCAGGTCTGAAGAGCGCAGGCGGCGGCTCGTTCC

GCCCGGCGGGAAGCGAGTCGATCGTCGCCGTCACCGTCTCGATCGGGCAGCAGCAGCGGTTTTCACTTGCTGCTCGCCGC

CTGCGCGCAGTCACCGGTACCT

>MH390370.1-Chlorocebus_aethiops-China Trichuris sp. Ca2 internal transcribed spacer 1, partial sequence; 5.8S ribosomal RNA gene, complete sequence; and internal transcribed spacer 2, partial sequence

GGCGTTTGCTCGGTCCGATGCGAGCAACGTCCGCCGGCTTACAGTTCCAGGTCTGAAGAGCGCAGGCGGCGGCTCGTTCC

GCCCGGCGGGCGAGTCGTCGATCGTCGCCGTCACCGGCTCGATCAGGCAGCAGCGGTTTTCACTTGCTGCTCGCCGCCTG

CGCGCAGTCACCGGTACCT

>KT344827.1-Cercopithecus_aethiops-China Trichuris sp. SC3 internal transcribed spacer 1, partial sequence; 5.8S ribosomal RNA gene, complete sequence; and internal transcribed spacer 2, partial sequence

GGCGTTTGCTCGGTCCGATGCGAGCAACGTCCGCCGGCTTACAGTTCCAGGTCTGAAGAGCGCAGGCGGCGGCTCGTTCC

GCCCGGCGGGCGAGTCGTCGATCGTCGCCGTCACCGGCTCGATCAGGCAGCAGCGGTTTTCACTTGCTGCTCGCCGCCTG

CGCGCAGTCACCGGTACCT

>KJ588071.1-Homo_sapiens-Uganda Trichuris sp. HU12 18S ribosomal RNA gene and internal transcribed spacer 1, partial sequence

GGCGTTTGCTCGGTCCGATGCGAGCAACGTCCGCCGGCTTACAGTTCCAGGTCTGAAGAGCGCAGGCGGCGGCTCGTTCC

GCCCGGCGGGCGAGTCGTCGATCGTCGCCGTCACCGGCTCGATCAGGCAGCAGCAATTTTCACTTGCTGCTCGCCGCCTG

CGCGCAGTCACCGGTACCT

>GQ301552.1-Papio_hamadryas_ursinus-South_Africa Trichuris sp. ex Papio ursinus NL-2009 isolate DGII internal transcribed spacer 1, partial sequence; 5.8S ribosomal RNA gene, complete sequence; and internal transcribed spacer 2, partial sequence

GGCGTTTGCTCGGTCCGATGCGAGCAACGTCCGCCGGCTTACAGTTCCAGGTCTGAAGAGCGCAGGCGGCGGCTCGTTCC

GCCCGGCGGGCGAGTCGTCCATCGTCGCCGTCACCGGCTCGATCAGGCAGCAGCAATTTTCACTTGCTGCTCGCCGCCTG

CGCGCAGTCACCGGTACCT

>AM992983.1-Unknown-China Trichuris trichiura ITS1 (partial), 5.8S rRNA gene and ITS2 (partial), isolate Ttr2c

GGCGTTTGCTCGGTCCGATGCGAGCAACGTCCGCCGGCTTACAGTTCCAGGTCTGAAGAGCGCAGGCGGCGGCTCGTTCC

GCCCGGCGGGCGAGTCGTCGATCGTCGCTGTCACCGCCTCGATCAGGCAGCAGCAATTTTCACTTGCTGCTCGCCGCCTG

CGCGCAGTCACCGGTACCT

>GQ301551.1-Papio_hamadryas_ursinus-South_Africa Trichuris sp. ex Papio ursinus NL-2009 isolate DGIII internal transcribed spacer 1, partial sequence; 5.8S ribosomal RNA gene, complete sequence; and internal transcribed spacer 2, partial sequence

GGCGTTTGCTCGGTCCGATGCGAGCAACGTCCGCCGGCTTACAGTTCCAGGTCTGAAGAGCGCAGGCGGCGGCTCGTTCC

GCCCGGCGGGCGAGTCGTCGATCGTCGCCGTCACCGGCTCGATCAGGCAGCAGCAATTTTCACTTGCTGCTCGCCGCCTG

CGCGCAGTCACCGGTACCT

>LR898010.1-Papio_papio-Spain Trichuris trichiura genomic DNA sequence contains ITS1

GGCGTTTGCTCGGTCCGATGCGAGCAACGTCCGCCGGCTTACAGTTCCAGGTCTGAAGAGCGCAGGCGGCGGCTCGTTCC

GCCCGGCGGGCGAGTCGTCGATCGTCGCCGTCACCGGCTCGATCAGGCAGCAGCAATTTTCACTTGCTGCTCGCCGCCTG

CGCGCAGTCACCGGTACCT

>AM992998.1-Unknown-China Trichuris trichiura ITS1 (partial), 5.8S rRNA gene and ITS2 (partial), isolate Ttr8c

GGCGTTTGCTCGGTCCGATGCGAGCAACGTCCGCCGGCTTACAGTTCCAGGTCTGAAGAGCGCAGGCGGCGGCTCGTTCC

GCCCGGCGGGCGAGTCGTCGATCGTCGCCGTCACCGGTTCGATCAGGCAGCAGCAATTTTCACTTGCTGCTCGCCGCCTG

CGCGCAGTCACCGGTACCT

>AM992995.1-Unknown-China Trichuris trichiura ITS1 (partial), 5.8S rRNA gene and ITS2 (partial), isolate Ttr7c

GGCGTTTGCTCGGTCCGATGCGAGCAACGTCCGCCGGCCTACAGTTCCAGGTCTGAAGAGCGCAGGCGGCGGCTCGTTCC

GCCCGGCGGGCGAGTCGTCGATCGTCGCCGTCACCGGCTCGATCAGGCAGCAGCGGTTTTCACTTGCTGCTCGCCGCCTG

CGCGCAGTCACCGGTACCT

>AM992993.1-Unknown-China Trichuris trichiura ITS1 (partial), 5.8S rRNA gene and ITS2 (partial), isolate Ttr7a

GGCGTTTGCTCGGTCCGATGCGAGCAACGTCCGCCGGCCTACAGTTCCAGGTCTGAAGAGCGCAGGCGGCGGCTCGTTCC

GCCCGGCGGGCGAGTCGTCGATCGTCGCCGTCACCGGCTCGATCAGGCAGCAGCGGTTTTCACTTGCTGCTCGCCGCCTG

CGCGCAGTCACCGGTACCT

>AM992997.1-Unknown-China Trichuris trichiura ITS1 (partial), 5.8S rRNA gene and ITS2 (partial), isolate Ttr8b

GGCGTTTGCTCGGTCCGATGCGAGCAACGTCCGCCGGCTTACAGTTCCAGGTCTGAAGAGCGCAGGCGGCGGCTCGTTCC

GCCCGGCGGGCGAGTCGTCGATCGTCGCCGTCACCGGTTCGATCAGGCAGCAGCAATTTTCACTTGCTGCTCGCCGCCTG

CGCGCAGTCACCGGTACCT

>AM992981.1-Unknown-China Trichuris trichiura ITS1 (partial), 5.8S rRNA gene and ITS2 (partial), isolate Ttr2a

GGCGTTTGCTCGGTCCGATGCGAGCAACGTCCGCCGGCTTACAGTTCCAGGTCTGAAGAGCGCAGGCGGCGGCTCGTTCC

GCCCGGCGGGCGAGTCGTCGATCGTCGCTGTCACCGCCTCGATCAGGCAGCAGCAATTTTCACTTGCTGCTCGCCGCCTG

CGCGCAGTCACCGGTACCT

>GQ352554.1-Homo_sapiens-Thailand Trichuris trichiura isolate TST214 18S ribosomal RNA gene, partial sequence; internal transcribed spacer 1, complete sequence; and 5.8S ribosomal RNA gene, partial sequence

GGCGTTTGCTCGGTCCGATGCGAGCAACGTCCGCCGGCTTACAGTTCCAGGTCTGAAGAGCGCAGGCGGCGGCTCGTTCC

GCCCGGCGGGCGAGTCGTCGATCGTCGCCGTCACCGGTTCGATCAGGCAGCAGCAATTTTCACTTGCTGCTCGCCGCCTG

CGCGCAGTCACCGGTACCT

>OR502845.1-Erythrocebus_patas-China Trichuris sp. isolate SCAU22193 small subunit ribosomal RNA gene and internal transcribed spacer 1, partial sequence

GGCGTTTGCTCGGTCCGATGCGAGCAACGTCCGCCGGCTTACAGTTCCAGGTCTGAAGAGCGCAGGCGGCGGCGGCTCGT

TCCGCCCGGCGGGCGAGTCGTCGATCGTCGCCGTCACCGGCTCGATCAGGCAGCAGCGGTTTTCACTTGCTGCTCGCCGC

CTGCGCGCAGTCACCGGTACCT

>GQ301553.1-Papio_hamadryas_ursinus-South_Africa Trichuris sp. ex Papio ursinus NL-2009 isolate DGI internal transcribed spacer 1, partial sequence; 5.8S ribosomal RNA gene, complete sequence; and internal transcribed spacer 2, partial sequence

GGCGTTTGCTCGGTCCGATGCGAGCAACGTCCGCCGGCCTACAGTTCCAGGTCTGAAGAGCGCAGGCGGCGGCGGCTCGT

TCCGCCCGGCGGGCGAGTCGTCGATCGTCGCCGTCACCGGCTCGATCAGGCAGCAGCGGTTTTCACTTGCTGCTCGCCGC

CTGCGCGCAGTCACCGGTACCT

>MN447328.1-Papio_anubis-China Trichuris sp. Pa1 GY-2019 small subunit ribosomal RNA gene, partial sequence; internal transcribed spacer 1, 5.8S ribosomal RNA gene, and internal transcribed spacer 2, complete sequence; and large subunit ribosomal RNA gene, partial sequence

GGCGTTTGCTCGGTCCGATGCGAGCAACGTCCGCCGGCCTACAGTTCCAGGTCTGAAGAGCGCAGGCGGCGGCGGCTCGT

TCCGCCCGGCGGGCGAGTCGTCGATCGTCGCCGTCACCGGCTCGATCAGGCAGCAGCGGTTTTCACTTGCTGCTCGCCGC

CTGCGCGCAGTCACCGGTACCT

>GQ352555.1-Homo_sapiens-Thailand Trichuris trichiura isolate TST332 18S ribosomal RNA gene, partial sequence; internal transcribed spacer 1, complete sequence; and 5.8S ribosomal RNA gene, partial sequence

GGCGTTTGCTCGGTTCGATGCGAGCAACGTCCGCCGGCCTACAGTTCCAGGTCTGAAGAGCGCAGGCGGCGGCGGCTCGT

TCCGCCCGGCGGGCGAGTCGTCGATCGTCGCCGTCACCGGCTCGATCAGGCAGCAGCGGTTTTCACTTGCTGCTCGCCGC

CTGCGCGCAGTCACCGGTACCT

>GQ352551.1-Homo_sapiens-Thailand Trichuris trichiura isolate TH2 clone E 18S ribosomal RNA gene, partial sequence; internal transcribed spacer 1, complete sequence; and 5.8S ribosomal RNA gene, partial sequence

GGCGTTTGCTCGGTCCGATGCGAGCAACGTCCGCCGGCCTACAGTTCCAGGTCTGAAGAGCGCAGGCGGCGGCGGCTCGT

TCCGCCCGGCGGGCGAGTCGTCGATCGTCGCCGTCACCGGCTCGATCAGGCAGCAGCGGTTTTCACTTGCTGCTCGCCGC

CTGCGCGCAGTCACCGGTACCT

>GQ352549.1-Homo_sapiens-Thailand Trichuris trichiura isolate TH2 clone C 18S ribosomal RNA gene, partial sequence; internal transcribed spacer 1, complete sequence; and 5.8S ribosomal RNA gene, partial sequence

GGCGTTTGCTCGGTCCGATGCGAGCAACGTCCGCCGGCCTACAGTTCCAGGTCTGAAGAGCGCAGGCGGCGGCGGCTCGT

TCCGCCCGGCGGGCGAGTCGTCGATCGTCGCCGTCACCGGCTCGATCAGGCAGCAGCGGTTTTCACTTGCTGCTCGCCGC

CTGCGCGCAGTCACCGGTACCT

>GQ352550.1-Homo_sapiens-Thailand Trichuris trichiura isolate TH2 clone D 18S ribosomal RNA gene, partial sequence; internal transcribed spacer 1, complete sequence; and 5.8S ribosomal RNA gene, partial sequence

GGCGTTTGCTCGGTCCGATGCGAGCAACGTCCGCCGGCCTACAGTTCCAGGTCTGAAGAGCGCAGGCGGCGGCGGCTCGT

TCCGCCCGGCGGGCGAGTCGTCGATCGTCGCCGTCACCGGCTCGATCAGGCAGCAGCGGTTTTCACTTGCTGCTCGCCGC

CTGCGCGCAGTCACCGGTACCT

>AM992994.1-Unknown-China Trichuris trichiura ITS1 (partial), 5.8S rRNA gene and ITS2 (partial), isolate Ttr7b

GGCGTTTGCTCGGTCCGATGCGAGCAACGTCCGCCGGCCTACAGTTCCAGGTCTGAAGAGCGCAGGCGGCGGCGGCTCGT

TCCGCCCGGCGGGCGAGTCGTCGATCGTCGCCGTCACCGGCTCGATCAGGCAGCAGCGGTTTTCACTTGCTGCTCGCCGC

CTGCGCGCAGTCACCGGTACCT

>GQ352548.1-Homo_sapiens-Thailand Trichuris trichiura isolate TH2 clone B 18S ribosomal RNA gene, partial sequence; internal transcribed spacer 1, complete sequence; and 5.8S ribosomal RNA gene, partial sequence

GGCGTTTGCTCGGTCCGATGCGAGCAACGTCCGCCGGCCTACAGTTCCAGGTCTGAAGAGCGCAGGCGGCGGCGGCTCGT

TCCGCCCGGCGGGCGAGTCGTCGATCGTCGCCGTCACCGGCTCGGTCAGGCAGCAGCGGTTTTCACTTGCTGCTCGCCGC

CTGCGCGCAGTCGCCGGTACCT

>AM992991.1-Unknown-China Trichuris trichiura ITS1 (partial), 5.8S rRNA gene and ITS2 (partial), isolate Ttr6b

GGCGTTTGCTCGGTCCGATGCGAGCAACGTCCGCCAGCTTACAGTTCCAGGTCTGAAGAGCGCAGGCGGCGGCGGCTCGT

TCCGCCCGGCGGGCGAGTCGTCGATCGTCGCCGTCACCGGCTCGATCAGGCAGCAGCAATTTTCACTTGCTGCTCGCCGC

CTGCGCGCAGTCACCGGTACCT

>AM992990.1-Unknown-China Trichuris trichiura ITS1 (partial), 5.8S rRNA gene and ITS2 (partial), isolate Ttr6a

GGCGTTTGCTCGGTCCGATGCGAGCAACGTCCGCCAGCTTACAGTTCCAGGTCTGAAGAGCGCAGGCGGCGGCGGCTCGT

TCCGCCCGGCGGGCGAGTCGTCGATCGTCGCCGTCACCGGCTCGATCAGGCAGCAGCAATTTTCACTTGCTGCTCGCCGC

CTGCGCGCAGTCACCGGTACCT

>AM992987.1-Unknown-China Trichuris trichiura ITS1 (partial), 5.8S rRNA gene and ITS2 (partial), isolate Ttr5a

GGCGTTTGCTCGGTCCGATGCGAGCAACGTCCGCCGGCCTACAGCTCCAGGTCTGAAGAGCGCAGGCGGCGGCGGCTCGT

TCCGCCCGGCGGGCGAGTCGTCGATCGTCGCCGTCACCGGCTCGATCAGGCAGCAGCGGTTTTCACTTGCTGCTCGCCGC

CTGCGCGCAGTCACCGGTACCT

>AM992985.1-Unknown-China Trichuris trichiura ITS1 (partial), 5.8S rRNA gene and ITS2 (partial), isolate Ttr3b

GGCGTTTGCTCGGTCCGATGCGAGCAACGTCCGCCGGCCTACAGTTCCAGGTCTGAAGAGCGCAGGCGGCGGCGGCTCGT

TCCGCCCGGCGGGCGAGTCGTCGATCGTCGCCGTCACCGGCTCGATCAGGCAGCAGCGGTTTTCACTTGCTGCTCGCCGC

CTGCGCGCAGTCACCGGTACCT

>OR502850.1-Macaca_mulatta-China Trichuris sp. isolate SCAU52151 small subunit ribosomal RNA gene, partial sequence; internal transcribed spacer 1, complete sequence; and 5.8S ribosomal RNA gene, partial sequence

GGCGTTTGCTCCGTCCGATGCGAGCAACGTCCGCCGGCTTACAGTTCCAGGTCTGAAGAGCGCAGGCGGCGGCGGCTCGT

TCCGCCCGGCGGGCGAGTCGTCGATCGTCGCCGTCACCGGCTCGATCGGGCAGCAGCAGCGGCTTTCACTTGCTGCTCGC

CGCCTGCGCGCAGTCACCGGTACCT

>OR502852.1-Macaca_mulatta-China Trichuris sp. isolate SCAU52158 small subunit ribosomal RNA gene, partial sequence; internal transcribed spacer 1, complete sequence; and 5.8S ribosomal RNA gene, partial sequence

GGCGTTTGCTCCGTCCGATGCGAGCAACGTCCGCCGGCTTACAGTTCCAGGTCTGAAGAGCGCAGGCGGCGGCTCGTTCC

GCCCGGCGGGAAGCGAGTCGATCGTCGCCGTCACCGGCTCGATCGGGCAGCACAAGCGGTTTTCACTTGCTGCTCGCCGC

CTGCGCGCAGTCACCGGTACCT

>OR502847.1-Macaca_nemestrina-China Trichuris sp. isolate SCAU22174 small subunit ribosomal RNA gene and internal transcribed spacer 1, partial sequence

GGCGTTTGCTCCGTCCGATGCGAGCAACGTCCGCCGGCTTACAGTTCCAGGTCTGAAGAGCGCAGGCGGCGGCTCGTTCC

GCCCGGCGGGAAGCGAGTCGATCGTCGCCGTCACCGGCTCGATCGGGCAGCAGCAGCGGTTTTCACTTGCTGCTCGCCGC

CTGCGCGCAGTCACCGGTACCT

>LR861802.1-Erythrocebus_patas-Spain Trichuris trichiura genomic DNA sequence contains ITS1, clone 50Patas

GGCGTTTGCTCCGTCCGATGCGAGCAACGTCCGCCGGCTTACAGTTCCAGGTCTGAAGAGCGCAGGCGGCGGCTCGTTCC

GCCCGGCGGGAAGCGAGTCGATCGTCGCCGTCACCGGCTCGATCGGGCAGCAGCAGCGGTTTTCACTTGCTGCTCGCCGC

CTGCGCGCAGTCACCGGTACCT

>LR898014.1-Erythrocebus_patas-Spain Trichuris trichiura genomic DNA sequence contains ITS1

GGCGTTTGCTCCGTCCGATGCGAGCAACGTCCGCCGGCTTACAGTTCCAGGTCTGAAGAGCGCAGGCGGCGGCTCGTTCC

GCCCGGCGGGAAGCGAGTCGATCGTCGCCGTCACCGGCTCGATCGGGCAGCAGCAGCGGTTTTCACTTGCTGCTCGCCGC

CTGCGCGCAGTCACCGGTACCT

>GQ352553.1-Homo_sapiens-Thailand Trichuris trichiura isolate TH1 18S ribosomal RNA gene, partial sequence; internal transcribed spacer 1, complete sequence; and 5.8S ribosomal RNA gene, partial sequence

GGCGTTTGCTCCGTCCGATGCGAGCAACGTCCGCCGGCTTACAGTTCCAGGTCTGAAGAGCGCAGGCGGCGGCTCGTTCC

GCCCGGCGGGAAGCGAGTCGATCGTCGCCGTCACCGGCTCGATCGGGCAGCAGCAGCGGTTTTCACTTGCTGCTCGCCGC

CTGCGCGCAGTCACCGGTACCT

>OR502846.1-Macaca_mulatta-China Trichuris sp. isolate SCAU52152 small subunit ribosomal RNA gene, partial sequence; internal transcribed spacer 1, complete sequence; and 5.8S ribosomal RNA gene, partial sequence

GGCGTTTGCTCCGTCCGATGCGAGCAACGTCCGCCGGCTTACAGTTCCAGGTCTGAAGAGCGCAGGCGGCGGCTCGTTCC

GCCCGGCGGGAAGCGAGTCGATCGTCGCCGTCATCGTCTCGATCGGGCAGCAGCAGCGGTTTTCACTTGCTGCTCGCCGC

CTGCGCGCAGTCACCGGTACCT

>MN447326.1-Macaca_mulatta-China Trichuris sp. Mm2 GY-2019 small subunit ribosomal RNA gene, partial sequence; internal transcribed spacer 1, 5.8S ribosomal RNA gene, and internal transcribed spacer 2, complete sequence; and large subunit ribosomal RNA gene, partial sequence

GGCGTTTGCTCCGTCCGATGCGAGCAACGTCCGCCGGCTTACAGTTCCAGGTCTGAAGAGCGCAGGCGGCGGCTCGTTCC

GCCCGGCGGGAAGCGAGTCGATCGTCGCCGTCACCGTCTCGATCGGGCAGCAGCAGCGGTTTTCACTTGCTGCTCGCCGC

CTGCGCGCAGTCACCGGTACCT

>OR502848.1-Macaca_mulatta-China Trichuris sp. isolate SCAU52156 small subunit ribosomal RNA gene, partial sequence; internal transcribed spacer 1, complete sequence; and 5.8S ribosomal RNA gene, partial sequence

GGCGTTTGCTCCGTCCGATGCGAGCAACGTCCGCCGGCTTACAGTTCCAGGTCTGAAGAGCGCAGGCGGCGGCTCGTTCC

GCCCGGCGGGAAGCGAGTCGATCGTCGCCGTCATCGTCTCGATCGGGCAGCAGCAGCGGTTTTCACTTGCTGCTCGCCGC

CTGCGCGCAGTCACCGGTACCT

>PV185672.1-Papio_hamadryas-China Trichuris sp. isolate Papio hamadryas small subunit ribosomal RNA gene and internal transcribed spacer 1, partial sequence

GGCGTTTGCTCCGTCCGATGCGAGCAACGTCCGCCGGCTTACAGTTCCAGGTCTGAAGAGCGCAGGCGGCGGCTCGTTCC

GCCCGGCGGGAAGCGAGTCGATCGTCGCCGTCACCGGCTCGATCGGGCAGCAGCAGCGGTTTTCACTTGCTGCTCGCCGC

CTGCGCGCAGTCACCGGTACCT

>MK192054.1-Macaca_fascicularis-Thailand Trichuris trichiura isolate ML39 small subunit ribosomal RNA gene and internal transcribed spacer 1, partial sequence

GGCGTTTGCTCCGTCCGATGCGAGCAACGTCCGCCGGCTTACAGTTCCAGGTCTGAAGAGCGCAGGCGGCGGCTCGTTCC

GCCCGGCGGGAAGCGAGTCGATCGTCGCCGTCACCGGCTCGATCGGGCAGCAGCAGCGGTTTTCACTTGCTGCTCGCCGC

CTGCGCGCAGTCACCGGTACCT

>OR502849.1-Macaca_mulatta-China Trichuris sp. isolate SCAU52153 small subunit ribosomal RNA gene, partial sequence; internal transcribed spacer 1, complete sequence; and 5.8S ribosomal RNA gene, partial sequence

GGCGTTTGCTCCGTCCGATGCGAGCAACGTCCGCCGGCTTACAGTTCCAGGTCTGAAGAGCGCAGGCGGCGGCTCGTTCC

GCCCGGCGGGAAGCGAGTCGATCGTCGCCGTCACCGGCTCGATCGGGCAGCAGCAGCGGTTTTCACTTGCTGCTCGCCGC

CTGCGCGCAGTCACCGGTACCT

>MK192056.1--Macaca_fascicularis-Thailand Trichuris trichiura isolate ML137 small subunit ribosomal RNA gene and internal transcribed spacer 1, partial sequence

GGCGTTTGCTCCGTCCGATGCGAGCAACGTCCGCCGGCTTACAGTTCCAGGTCTGAAGAGCGCAGGCGGCGGCTCGTTCC

GCCCGGCGGGAAGCGAGTCGATCGTCGCCGTCACCGGCTCGATCGGGCAGCAGCAGCGGTTTTCACTTGCTGCTCGCCGC

CTGCGCGCAGTCACCGGTACCT

>PV185671.1-Nomascus_leucogenys-China Trichuris sp. isolate Nomascus leucogenys small subunit ribosomal RNA gene and internal transcribed spacer 1, partial sequence

GGCGTTTGCTCCGGCCGATGCGAGCAACGTCCGCCGGCTTACAGTTCCAGGTCTGAAGAGCGCAGGCGGCGGCTCGTTCC

GCCCGGCGGGAAGCGAGTCGATCGTCGCCGTCACCGGCTCGATCGGGCAGCTGCAGCGGTTTTCACTTGCTGCTCGCCGC

CTGCGCGCAGTCACCGGTACCT

>MN447320.1-Papio_hamadryas-China Trichuris sp. Ph1 GY-2019 small subunit ribosomal RNA gene, partial sequence; internal transcribed spacer 1, complete sequence; and 5.8S ribosomal RNA gene, partial sequence

GGCGTTTGCTCCGTCCGATGCGAGCAACGTCCGCCGGCTTACAGTTCCAGGTCTGAAGAGCGCAGGCGGCGGCTCGTTCC

GCCCGGCGGGAAGCGAGTCGATCGTCGCCGTCACCGTCTCGATCGGGCAGCAGCAGCGGTTTTCACTTGCTGCTCGCCGC

CTGCGCGCAGTCACCGGTACCT

>MK192055.1-Macaca_fascicularis-Thailand Trichuris trichiura isolate ML133 small subunit ribosomal RNA gene and internal transcribed spacer 1, partial sequence

GGCGTTTGCTCCGTCCGATGCGAGCAACGTCCGCCGGCTTACAGTTCCAGGTCTGAAGAGCGCAGGCGGCGGCTCGTTCC

GCCCGGCGGGAAGCGAGTCGATCGTCGCCGTCACCGTCTCGATCGGGCAGCAGCAGCGGTTTTCACTTGCTGCTCGCCGC

CTGCGCGCAGTCACCGGTACCT

>KT344828.1-Macaca_leonina-China Trichuris sp. SC4 internal transcribed spacer 1, partial sequence; 5.8S ribosomal RNA gene, complete sequence; and internal transcribed spacer 2, partial sequence

GGCGTTTGCTCCGTCCGATGCGAGCAACGTCCGCCGGCTTACAGTTCCAGGTCTGAAGAGCGCAGGCGGCGGCTCGTTCC

GCCCGGCGGGAAGCGAGCGAGTCGATCGTCGCCGTCACCGGCTCGATCAGGCAGCAGCAGCGGTTTTCACTTGCTGCTCG

CCGCCTGCGCGCAGTCACCGGTACCT

>MH390364.1-Macaca_leonina-China Trichuris sp. Ml2 internal transcribed spacer 1, partial sequence; 5.8S ribosomal RNA gene, complete sequence; and internal transcribed spacer 2, partial sequence

GGCGTTTGCTCCGTCCGATGCGAGCAACGTCCGCCGGCTTACAGTTCCAGGTCTGAAGAGCGCAGGCGGCGGCTCGTTCC

GCCCGGCGGGAAGCGAGCGAGTCGATCGTCGCCGTCACCGGCTCGATCAGGCAGCAGCAGCGGTTTTCACTTGCTGCTCG

CCGCCTGCGCGCAGTCACCGGTACCT

>LR898006.1-Macaca_sylvanus-Spain Trichuris trichiura genomic DNA sequence contains ITS1

GGCGTTTGCTCCGTCCGATGCGAGCAACGTCCGCCGGCTTACAGTTCCAGGTCTGAAGAGCGCAGGCGGCGGCTCGTTCC

GCCCGGCGGGAAGCGAGCGAGTCGATCGTCGCCGTCACCGGCTCGATCAGGCAGCAGCAGCGGTTTTCACTTGCTGCTCG

CCGCCTGCGCGCAGTCACCGGTACCT

>MH390365.1-Macaca_leonina-China Trichuris sp. Ml3 internal transcribed spacer 1, partial sequence; 5.8S ribosomal RNA gene, complete sequence; and internal transcribed spacer 2, partial sequence

GGCGTTTGCTCCGTCCGATGCGAGCAACGTCCGCCGGCTTACAGTTCCAGGTCTGAAGAGCGCAGGCGGCGGCTCGTTCC

GCCCGGCGGGAAGCGAGCGAGTCGATCGTCGCCGTCACCGGCTCGATCAGGCAGCAGCAGCGGTTTTCACTTGCTGCTCG

CCGCCTGCGCGCAGTCACCGGTACCT

>LR861796.1-Macaca_sylvanus-Spain Trichuris trichiura genomic DNA sequence contains ITS1, clone TM1, TM8, TM20, TH27

GGCGTTTGCTCCGTCCGATGCGAGCAACGTCCGCCGGCTTACAGTTCCAGGTCTGAAGAGCGCAGGCGGCGGCTCGTTCC

GCCCGGCGGGAAGCGAGCGAGTCGATCGTCGCCGTCACCGGCTCGATCAGGCAGCAGCAGCGGTTTTCACTTGCTGCTCG

CCGCCTGCGCGCAGTCACCGGTACCT

>LR898005.1-Macaca_sylvanus-Spain Trichuris trichiura genomic DNA sequence contains ITS1

GGCGTTTGCTCCGTCCGATGCGAGCAACGTCCGCCGGCTTACAGTTCCAGGTCTGAAGAGCGCAGGCGGCGGCTCGTTCC

GCCCGGCGGGAAGCGAGCGAGTCGATCGTCGCCGTCACCGGCTCGATCAGGCAGCAGCAGCGGTTTTCACTTGCTGCTCG

CCGCCTGCGCGCAGTCACCGGTACCT

>LR861795.1-Macaca_sylvanus-Spain Trichuris trichiura genomic DNA sequence contains ITS1, clone TM18

GGCGTTTGCTCCGTCCGATGCGAGCAACGTCCGCCGGCTTACAGTTCCAGGTCTGAAGAGCGCAGGCGGCGGCTCGTTCC

GCCCGGCGGGAAGCGAGCGAGTCGATCGTCGCCGTCACCGGCTCGATCAGGCAGCAGCAGCGGTTTTCACTTGCTGCTCG

CCGCCTGCGCGCAGTCACCGGTACCT

>LR132058.1-Macaca_sylvanus-Spain Trichuris sp. 8610 genomic DNA sequence contains ITS1

GGCGTTTGCTCCGTCCGATGCGAGCAACGTCCGCCGGCTTACAGTTCCAGGTCTGAAGAGCGCAGGCGGCGGCTCGTTCC

GCCCGGCGGGAAGCGAGCGAGTCGATCGTCGCCGTCACCGGCTCGATCAGGCAGCAGCAGCGGTTTTCACTTGCTGCTCG

CCGCCTGCGCGCAGTCACCGGTACCT

>LR898004.1-Macaca_sylvanus-Spain Trichuris trichiura genomic DNA sequence contains ITS1

GGCGTTTGCTCCGTCCGATGCGAGCAACGTCCGCCGGCTTACAGTTCCAGGTCTGAAGAGCGCAGGCGGCGGCTCGTTCC

GCCCGGCGGGAAGCGAGCGAGTCGATCGTCGCCGTCACCGGCTCGATCAGGCAGCAGCAGCGGTTTTCACTTGCTGCTCG

CCGCCTGCGCGCAGTCACCGGTACCT

>LR861792.1-Macaca_sylvanus-Spain Trichuris trichiura genomic DNA sequence contains ITS1, clone TH30

GGCGTTTGCTCCGTCCGATGCGAGCAACGTCCGCCGGCTTACAGTTCCAGGTCTGAAGAGCGCAGGCGGCGGCTCGTTCC

GCCCGGCGGGAAGCGAGCGAGTCGATCGTCGCCGTCACCGGCTCGATCAGGCAGCAGCAGCGGTTTTCACTTGCTGCTCG

CCGCCTGCGCGCAGTCACCGGTACCT

>LR861797.1-Macaca_sylvanus-Spain Trichuris trichiura genomic DNA sequence contains ITS1, clone TH31, TH3, TM3, TM5, TM19, TM31

GGCGTTTGCTCCGTCCGATGCGAGCAACGTCCGCCGGCTTACAGTTCCAGGTCTGAAGAGCGCAGGCGGCGGCTCGTTCC

GCCCGGCGGGAAGCGAGCGAGTCGATCGTCGCCGTCACCGGCTCGATCAGGCAGCAGCAGCGGTTTTCACTTGCTGCTCG

CCGCCTGCGCGCAGTCACCGGTACCT

>LR898009.1-Macaca_sylvanus-Spain Trichuris trichiura genomic DNA sequence contains ITS1

GGCGTTTGCTCCGTCCGATGCGAGCAACGTCCGCCGGCTTACAGTTCCAGGTCTGAAGAGCGCAGGCGGCGGCTCGTTCC

GCCCGGCGGGAAGCGAGCGAGTCGATCGTCGCCGTCACCGGCTCGATCAGGCAGCAGCAGCGGTTTTCACTTGCTGCTCG

CCGCCTGCGCGCAGTCACCGGTACCT

>LR861798.1-Papio_papio-Spain Trichuris trichiura genomic DNA sequence contains ITS1, clone TPM1

GGCGTTTGCTCCGTCCGATGCGAGCAACGTCCGCCGGCTTACAGTTCCAGGTCTGAAGAGCGCAGGCGGCGGCTCGTTCC

GCCCGGCGGGAAGCGAGCGAGTCGATCGTCGCCGTCACCGGCTCGATCAGGCAGCAGCAGCGGTTTTCACTTGCTGCTCG

CCGCCTGCGCGCAGTCACCGGTACCT

>PV185673.1-Erythrocebus_patas-China Trichuris sp. isolate Erythrocebus patas small subunit ribosomal RNA gene and internal transcribed spacer 1, partial sequence

GGCGTTTGCTCCGTCCGATGCGAGCAACGTCCGCCGGCTTACAGTTCCAGGTCTGAAGAGCGCAGGCGGCGGATCGTTCC

GCCCGGCGGGAAGCGAGCGAGTCGATCGTCGCCGTCACCGGCTCGATCAGGCAGCAGCAGCGGTTTTCACTTGCTGCTCG

CCGCCTGCGCGCAGTCACCGGTACCT

>MH390362.1-Rhinopithecus_bieti-China Trichuris sp. Rb2 internal transcribed spacer 1, partial sequence; 5.8S ribosomal RNA gene, complete sequence; and internal transcribed spacer 2, partial sequence

GGCGTTTGCTCCGTCCGATGCGAGCAACGTCCACCGGCTTACAGTTCCAGGTCTGAAGAGCGCAGGCGGCGGCTCGTTCC

GCCCGGCGAGTGAGTCGTCGCCGTCACCGGCTCGATCAGGCAGCAGCAATTTTCACTTGCTGCTCGCCGCCTGCGCACAA

TCACCGGTACCT

>KT344832.1-Rhinopithecus_bieti-China Trichuris sp. KM1 internal transcribed spacer 1, partial sequence; 5.8S ribosomal RNA gene, complete sequence; and internal transcribed spacer 2, partial sequence

GGCGTTTGCTCCGTCCGATGCGAGCAACGTCCACCGGCTTACAGTTCCAGGTCCGAAGAGCGCAGGCGGCGGCTCGTTCC

GCCCGGCGAGTGAGTCGTCGCCGTCACCGGCTCGATCAGGCAGCAGCAATTTTCACTTGCTGCTCGCCGCCTGCGCACAA

TCACCGGTACCT

>OR502843.1-Hylobatidae-China Trichuris sp. isolate SCAU22180 small subunit ribosomal RNA gene and internal transcribed spacer 1, partial sequence

GGCGTTTGCTCCGTCCGATGTGAGCAACGTCCACCGGCTTACAGTTCCAGGTCTGCAGAGCGCAGGCGGCGGCTCGTTCC

GCCCGGCGAGTTGATCGTCGCCGTCACCGGCACGATCAGGCAGCAGCGGTTTTCACTTGCTGCTCGCTGCCTGCGCACAA

TCACCGGTACCT

>LR898008.1-Macaca_sylvanus-Spain Trichuris trichiura genomic DNA sequence contains ITS1

GGTGTTTGCTCTTGCGGAGTCTGATGCGGTCCACGCGCGCAGGCTTCCAGGTCTGAAGAGCGCAGGCGGCGGCTCGTTCC

GCCCGGCGGGAAGCGAGCGAGTCGATCGTCGCCGTCACCGGCTCGATCAGGCAGCAGCAGCGGTTTTCACTTGCTGCTCG

CCGCCTGCGCGCAGTCACCGGTACCT

>LR861793.1-Macaca_sylvanus-Spain Trichuris trichiura genomic DNA sequence contains ITS1, clone TH26

GGTGTTTGCTCTTGCGGAGTCTGATGCGGTCCACGCGCGCAGGCTTCCAGGTCTGAAGAGCGCAGGCGGCGGCTCGTTCC

GCCCGGCGGGAAGCGAGCGAGTCGATCGTCGCCGTCACCGGCTCGATCAGGCAGCAGCAGCGGTTTTCACTTGCTGCTCG

CCGCCTGCGCGCAGTCACCGGTACCT

>LR861800.1-Chlorocebus_aethiops-Spain Trichuris trichiura genomic DNA sequence contains ITS1, clone HT50.2

GGCGTTTGCTCCGTCCGAATGCGACCGACGTCCGCGAGGCTCTAGCGGTTCCGTTCAGTCGTTAAATTAGTCGCAAAGTC

AGGCGGGCTGCCGTTCCGCCCGGTGGGAACCGAGTCGATCGTCGCCGTCACCGGCTCCATCGGGCAGCAGCAGCGGTTTT

CACTTGCTGCTCTTCCTCTGCGCGCTCTCCCCGGTACCT

>LR898012.1-Chlorocebus_aethiops-Spain Trichuris trichiura genomic DNA sequence contains ITS1

GGCGTTTGCTCCGTCCGAATGCGACCGACGTCCGCGAGGCTCTAGCGGTTCCGTTCAGTCGTTAAATTAGTCGCAAAGTC

AGGCGGGCTGCCGTTCCGCCCGGTGGGAACCGAGTCGATCGTCGCCGTCACCGGCTCCATCGGGCAGCAGCAGCGGTTTT

CACTTGCTGCTCTTCCTCTGCGCGCTCTCCCCGGTACCT

>AJ783398.1-Trichuris_suis-Sus_scrofa-Spain Trichuris suis internal transcribed spacer 1 (ITS1), specific host Sus scrofa scrofa

GACGTGAGCGCGCGTTGCCACTGCCGTGAGCAGCGTCCACCGTTGCACCACCACCACTTCCAGATCCCAAGCGCAGGCCG

CGCTGGCTACGGTCGTCCTGCGCCGCTTGACTCGGTGATCACCACCAGTACGACCGGCTGCAGACGGACGACTGCGTGGC

TGAGTCAGTCGGCATCGTCGCGGGACGTCCGTGCTTGCTCGCCTGCCAACAACGACCGGTACCT

>AM993016.1-Trichuris_suis-China Trichuris suis ITS1 (partial), 5.8S rRNA gene and ITS2 (partial), isolate TsZJ3c

GACGTGAGCGCGCGTTGCCACTGCCGTGAGCAGCGTCCACCGTTGCACCACCACTTCCAGATCCGAAGCGCAGGCCGCGC

TGGCTACGGTCGTCCTGCGCCGCTTGACTCGGTGATCACCACCAGTACGACCGGCTGCAGACGGACGACTGCGTGGCTGA

GTCAGTCGGCATCGTCGCGGGACGTCCGTGCTTGCTCGCCTGCCAACAACGACCGGTACCT

>FM991955.1-Trichuris_colobae-Nomascus_gabriellae-Spain Trichuris colobae ITS1, 5.8S rRNA gene and ITS2, isolated from Nomascus gabriellae

GACGTGAGCGCGCGTTGCCACTGCCGAGAGCAGCGTCCACCGTTGCACCAGCAGTTCCAGATCCGAAGAGCAGGTCGCGC

TGACCACGGTCGACCTGCGCCGCTCGACACGGTGACCACCAGCAGTACGGCCGGCTGCAGACGGACGACTGCGCGGCTGA

GTCAGCCAGCATCGTCGAGCGGCCGGACGTCCGTGTTCCCGCGCCTGCCAACAACGATGACCGGTACCT

>FM991956.1-Trichuris_colobae-Colobus_guereza_kikuyuensis Trichuris colobae ITS1, 5.8S rRNA gene and ITS2, isolated from Colobus guereza kikuyuensis

GACGTGAGCGCGCGTTGCCACTGCCGAGAGCAGCGTCCACCGTTGCACCAGCAGTTCCAGATCCGAAGAGCAGGTCGCGC

TGACCACGGTCGACCTGCGCCGCTCGACACGGTGACCACCAGCAGTACGGCCGGCTGCAGACGGACGACTGCGCGGCTGA

GTCAGCCAGCATCGTCGAGCGGCCGGACGTCCGTGTTCCCGCGCCTGCCAACAACGATGACCGGTACCT

#### ITS1 haplotypes

>Hap_1

GGCGTTTGCTCG-GTC-CGA-TGC---GAGCAACGTCCGCC--GGCC-T---A-CAGTTCC----AG--GTCTGAAGAG-

CGCAG-GCG--GCGGCGGCTCGTTCCGCCCGGCGGG-------CGAGTCGTCGATCGTCGCC-GTC--ACCGGCT-----

----CGA--T-----------CAGGCAGCAGCGGTTTTC------ACTTGC-TGCTCGC-CGCCTGCGCGCA--G-TCAC

CGGTACCT

>Hap_2

GGCGTTTGCTCG-GTC-CGA-TGC---GAGCAACGTCCGCC--GGCT-T---A-CAGTTCC----AG--GTCTGAAGAG-

CGCAG-GCG--GCGG-CT--CGTTCCGCCCGGCGGG-------CGAGTCGTCGATCGTCGCC-GTC--ACCGGCT-----

----CGA--T-----------CAGGCAGCAGCAATTTTC------ACTTGC-TGCTCGC-CGCCTGCGCGCA--G-TCAC

CGGTACCT

>Hap_3

GGCGTTTGCTC-CGTC-CGA-TGC---GAGCAACGTCCGCC--GGCT-T---A-CAGTTCC----AG--GTCTGAAGAG-

CGCAG-GCG--GCGG-CT--CGTTCCGCCCGGCGGGAAGCGAGCGAGTCG---ATCGTCGCC-GTC--ACCGGCT-----

----CGA--T-------CAGGCAG-CAGCAGCGGTTTTC------ACTTGC-TGCTCGC-CGCCTGCGCGCA--G-TCAC

CGGTACCT

>Hap_4

GGCGTTTGCTC-CGTC-CGA-TGC---GAGCAACGTCCGCC--GGCT-T---A-CAGTTCC----AG--GTCTGAAGAG-

CGCAG-GCG--GCGG-CT--CGTTCCGCCCGGCGGGA----AGCGAGTCG---ATCGTCGCC-GTC--ACCGGCT-----

----CGA--T-------CGGGCAG-CAGCAGCGGTTTTC------ACTTGC-TGCTCGC-CGCCTGCGCGCA--G-TCAC

CGGTACCT

>Hap_5

GGCGTTTGCTC-CGTC-CGA-TGC---GAGCAACGTCCGCC--GGCT-T---A-CAGTTCC----AG--GTCTGAAGAG-

CGCAG-GCG--GCGG-CT--CGTTCCGCCCGGCGGGA----AGCGAGTCG---ATCGTCGCC-GTC--ACCGTCT-----

----CGA--T-------CGGGCAG-CAGCAGCGGTTTTC------ACTTGC-TGCTCGC-CGCCTGCGCGCA--G-TCAC

CGGTACCT

>Hap_6

GGCGTTTGCTC-CGTC-CGA-TGC---GAGCAACGTCCGCC--GGCT-T---A-CAGTTCC----AG--GTCTGAAGAG-

CGCAG-GCG--GCGG-CT--CGTTCCGCCCGGCGGGA----AGCGAGTCG---ATCGTCGCC-GTC--ATCGTCT-----

----CGA--T-------CGGGCAG-CAGCAGCGGTTTTC------ACTTGC-TGCTCGC-CGCCTGCGCGCA--G-TCAC

CGGTACCT

>Hap_7

GGCGTTTGCTCG-GTC-CGA-TGC---GAGCAACGTCCGCC--GGCT-T---A-CAGTTCC----AG--GTCTGAAGAG-

CGCAG-GCG--GCGG-CT--CGTTCCGCCCGGCGGG-------CGAGTCGTCGATCGTCGCC-GTC--ACCGGTT-----

----CGA--T-----------CAGGCAGCAGCAATTTTC------ACTTGC-TGCTCGC-CGCCTGCGCGCA--G-TCAC

CGGTACCT

>Hap_8

GGCGTTTGCTC-CGTC-CGA-TGC---GAGCAACGTCCGCC--GGCT-T---A-CAGTTCC----AG--GTCTGAAGAG-

CGCAG-GCG--GCGG-CT--CGTTCCGCCCGGCGGGA----AGCGAGTCG---ATCGTCGCC-GTC--ATCGACT-----

----CGA--T-------CGGGCAG-CAGCAGCGGTTTTC------ATTTGC-TGCTCGC-CGCCTGCGCGCA--G-TCAC

CGGTACCT

>Hap_9

GGCGTTTGCTCG-GTC-CGA-TGC---GAGCAACGTCCGCC--GGCC-T---A-CAGTTCC----AG--GTCTGAAGAG-

CGCAG-GCG--GCGG-CT--CGTTCCGCCCGGCGGG-------CGAGTCGTCGATCGTCGCC-GTC--ACCGGCT-----

----CGA--T-----------CAGGCAGCAGCGGTTTTC------ACTTGC-TGCTCGC-CGCCTGCGCGCA--G-TCAC

CGGTACCT

>Hap_10

GGCGTTTGCTCG-GTC-CGA-TGC---GAGCAACGTCCGCC--GGCT-T---A-CAGTTCC----AG--GTCTGAAGAG-

CGCAG-GCG--GCGG-CT--CGTTCCGCCCGGCGGG-------CGAGTCGTCGATCGTCGCT-GTC--ACCGCCT-----

----CGA--T-----------CAGGCAGCAGCAATTTTC------ACTTGC-TGCTCGC-CGCCTGCGCGCA--G-TCAC

CGGTACCT

>Hap_11

GGCGTTTGCTCG-GTC-CGA-TGC---GAGCAACGTCCGCC--GGCT-T---A-CAGTTCC----AG--GTCTGAAGAG-

CGCAG-GCG--GCGG-CT--CGTTCCGCCCGGCGGG-------CGAGTCGTCGATCGTCGCC-GTC--ACCGGCT-----

----CGA--T-----------CAGGCAGCAGCGGTTTTC------ACTTGC-TGCTCGC-CGCCTGCGCGCAG---TCAC

CGGTACCT

>Hap_12

GGCGTTTGCTCG-GTC-CGA-TGC---GAGCAACGTCCGCC--AGCT-T---A-CAGTTCC----AG--GTCTGAAGAG-

CGCAG-GCG--GCGGCGGCTCGTTCCGCCCGGCGGG-------CGAGTCGTCGATCGTCGCC-GTC--ACCGGCT-----

----CGA--T-----------CAGGCAGCAGCAATTTTC------ACTTGC-TGCTCGC-CGCCTGCGCGCAG---TCAC

CGGTACCT

>Hap_13

GGCGTTTGCTCG-GTC-CGA-TGC---GAGCAACGTCCGCC--GGCT-T---A-CAGTTCC----AG--GTCTGAAGAG-

CGCAG-GCG--GCGGCGGCTCGTTCCGCCCGGCGGG-------CGAGTCGTCGATCGTCGCC-GTC--ACCGGCT-----

----CGA--T-----------CAGGCAGCAGCGGTTTTC------ACTTGC-TGCTCGC-CGCCTGCGCGCAG---TCAC

CGGTACCT

>Hap_14

GGCGTTTGCTC-CGTC-CGA-TGC---GAGCAACGTCCGCC--GGCT-T---A-CAGTTCC----AG--GTCTGAAGAG-

CGCAG-GCG--GCGG-CT--CGTTCCGTCCGGCGGGA----AGCGAGTCG---ATCGTCGCC-GTC--ACCGTCT-----

----CGA--T-------CGGGCAG-CAGCAGCGGTTTTC------ACTTGC-TGCTCGC-CGCCTGCGCGCAG---TCAC

CGGTACCT

>Hap_15

GGCGTTTGCTCG-GTT-CGA-TGC---GAGCAACGTCCGCC--GGCC-T---A-CAGTTCC----AG--GTCTGAAGAG-

CGCAG-GCG--GCGGCGGCTCGTTCCGCCCGGCGGG-------CGAGTCGTCGATCGTCGCC-GTC--ACCGGCT-----

----CGA--T-----------CAGGCAGCAGCGGTTTTC------ACTTGC-TGCTCGC-CGCCTGCGCGCAG---TCAC

CGGTACCT

>Hap_16

GGCGTTTGCTCG-GTC-CGA-TGC---GAGCAACGTCCGCC--GGCC-T---A-CAGTTCC----AG--GTCTGAAGAG-

CGCAG-GCG--GCGGCGGCTCGTTCCGCCCGGCGGG-------CGAGTCGTCGATCGTCGCC-GTC--ACCGGCT-----

----CG---G----------TCAGGCAGCAGCGGTTTTC------ACTTGC-TGCTCGC-CGCCTGCGCGCAG---TCGC

CGGTACCT

>Hap_17

GAGCCAGGCTC-CGTC-CGA-TGC---GAGCAACGTCCGCC--GGCT-T---A-CAGTTCC----AG--GTCTGAAGAG-

CGCAG-GCG--GCGG-CT--CGTTCCGCCCGGCGGGA----AGCGAGTCG---ATCGTCGCC-GTC--ATCGACTC----

-----GA---T------CGGGCAG-CAGCAGCGGTTTTC------ATTTGC-TGCTCGC-CGCCTGCGCGCAG---TCAC

CGGTACCT

>Hap_18

GGCGTTTGCTCG-GTC-CGA-TGC---GAGCAACGTCCGCC--GGCT-T---A-CAGTTCC----AG--GTCTGAAGAG-

CGCAG-GCG--GCGG-CT--CGTTCCGCCCGGCGGG-------CGAGTCGTCCATCGTCGCC-GTC--ACCGGCT-----

----CGA--T-----------CAGGCAGCAGCAATTTTC------ACTTGC-TGCTCGC-CGCCTGCGCGCAG---TCAC

CGGTACCT

>Hap_19

GGCGTTTGCTCG-GTC-CGA-TGC---GAGCAACGTCCGCC--GGCC-T---A-CAGCTCC----AG--GTCTGAAGAG-

CGCAG-GCG--GCGGCGGCTCGTTCCGCCCGGCGGG-------CGAGTCGTCGATCGTCGCC-GTC--ACCGGCT-----

----CGA--T-----------CAGGCAGCAGCGGTTTTC------ACTTGC-TGCTCGC-CGCCTGCGCGCAG---TCAC

CGGTACCT

>Hap_20

GGCGTTTGCTC-CGTC-CGA-TGC---GAGCAACGTCCACC--GGCT-T---A-CAGTTCC----AG--GTCCGAAGAG-

CGCAG-GCG--GCGG-CT--CGTTCCGCCCGGCGAG-------TGAGT-------CGTCGCC-GTC--ACCGGCT-----

----CGA--T-----------CAGGCAGCAGCAATTTTC------ACTTGC-TGCTCGC-CGCCTGCGCACAA---TCAC

CGGTACCT

>Hap_21

GGCGTTTGCTC-CGGC-CGA-TGC---GAGCAACGTCCGCC--GGCT-T---A-CAGTTCC----AG--GTCTGAAGAG-

CGCAG-GCG--GCGG-CT--CGTTCCGCCCGGCGGGA----AGCGAGTCG---ATCGTCGCC-GTC--ACCGGCT-----

----CGA--T-------CGGGCAG-CTGCAGCGGTTTTC------ACTTGC-TGCTCGC-CGCCTGCGCGCAG---TCAC

CGGTACCT

>Hap_22

GGCGTTTGCTC-CGTC-CGA-TGC---GAGCAACGTCCGCC--GGCT-T---A-CAGTTCC----AG--GTCTGAAGAG-

CGCAG-GCG--GCGG---ATCGTTCCGCCCGGCGGGAAGCGAGCGAGTCG---ATCGTCGCC-GTC--ACCGGCT-----

----CGA--T-------CAGGCAG-CAGCAGCGGTTTTC------ACTTGC-TGCTCGC-CGCCTGCGCGCAG---TCAC

CGGTACCT

>Hap_23

GGCGTTTGCTC-CGTC-CGA-TGC---GAGCAACGTCCACC--GGCT-T---A-CAGTTCC----AG--GTCTGAAGAG-

CGCAG-GCG--GCGG-CT--CGTTCCGCCCGGCGAG-------TGAGT-------CGTCGCC-GTC--ACCGGCT-----

----CGA--T-----------CAGGCAGCAGCAATTTTC------ACTTGC-TGCTCGC-CGCCTGCGCACAA---TCAC

CGGTACCT

>Hap_24

GGCGTTTGCTC-CGTC-CGA-TGC---GAGCAACGTCCGCC--GGCT-T---A-CAGTTCC----AG--GTCTGAAGAG-

CGCAG-GCG--GCGGCGGCTCGTTCCGCCCGGCGGG-------CGAGTCGTCGATCGTCGCC-GTC--ACCGGCT-----

----CGA--T-------CGGGCAG-CAGCAGCGGCTTTC------ACTTGC-TGCTCGC-CGCCTGCGCGCAG---TCAC

CGGTACCT

>Hap_25

GGCGTTTGCTC-CGTC-CGA-TGC---GAGCAACGTCCGCC--GGCT-T---A-CAGTTCC----AG--GTCTGAAGAG-

CGCAG-GCG--GCGG-CT--CGTTCCGCCCGGCGGGA----AGCGAGTCG---ATCGTCGCC-GTC--ACCGGCT-----

----CGA--T-------CGGGCAG-CACAAGCGGTTTTC------ACTTGC-TGCTCGC-CGCCTGCGCGCAG---TCAC

CGGTACCT

>Hap_26

GGCGTTTGCTC-CGTC-CGA-TGT---GAGCAACGTCCACC--GGCT-T---A-CAGTTCC----AG--GTCTGCAGAG-

CGCAG-GCG--GCGG-CT--CGTTCCGCCCGGC-----------GAGTTG---ATCGTCGCC-GTC--ACCGGCA-----

----CGA--T-----------CAGGCAGCAGCGGTTTTC------ACTTGC-TGCTCGC-TGCCTGCGCACAA---TCAC

CGGTACCT

>Hap_27

GGTGTTTGCTCTTGCGGAGTCTGATGCGGTCCACGCGCGCA--GGC-----------TTCC----AG--GTCTGAAGAG-

CGCAG-GCG--GCGG-CT--CGTTCCGCCCGGCGGGAAGCGAGCGAGTCG---ATCGTCGCC-GTC--ACCGGCT-----

----CGA--T-------CAGGCAG-CAGCAGCGGTTTTC------ACTTGC-TGCTCGC-CGCCTGCGCGCAG---TCAC

CGGTACCT

>Hap_28

GACGTGAGCGCGCGTTGCCACTGCCGAGAGCAGCGTCCACCGTTGCACC---AGCAGTTCC----AG--ATCCGAAGA--

-GCAG-GTCGCGCTGACCA-CGGT-CGACCTGCGCC----GCTCGACACGGTGACCACCAGCAGTACGGCCGGCTGCAGA

CGGACGACTGCGCGGCTGAGTCAGCCAGCATCGTCGAGCGGCCGGACGTCCGTGTTCCCGCGCCTGCCAACAACGATGAC

CGGTACCT

>Hap_29

GACGTGAGCGCGCGTTGCCACTGCCGTGAGCAGCGTCCACCGTTGCACC---ACCACTTCC----AGA--TCCGAAG---

CGCAG-GCCGCGCTGGCTA-CGGT-CGTCCTGCGCC----GCTTGACTCGGTGATCACCACCAGTACGACCGGCTGCAGA

CGGACGACTGCGTGGCTGAGTCAGTCGGCATCG--TCGCGG---GACGTCCGTGCTTGCTCGCCTGCCAACAACG---AC

CGGTACCT

>Hap_30

GACGTGAGCGCGCGTTGCCACTGCCGTGAGCAGCGTCCACCGTTGCACCACCACCACTTCC----AG--ATCCCAAG---

CGCAGGCCGC-GCTGGCTA-CGGT-CGTCCTGCGCCG----CTTGACTCGGTGATCACCACCAGTACGACCGGCTGCAGA

CGGACGACTGCGTGGCTGAGTCAGTCGGCATCG--TCGCGGG---ACGTCCGTGCTTGCTCGCCTGCCAACAACG---AC

CGGTACCT

>Hap_31

GGCGTTTGCTC-CGTC-CGAATGC---GACCGACGTCCGCG-AGGCTCT---AGCGGTTCCGTTCAGTCGTTAAATTAGT

CGCAAAGTCAGGCGGGCTGCCGTTCCGCCCGGTGGGA----ACCGAGTCG---ATCGTCGCC-GTC--ACCGGCT-C---

----CAT----C------GGGCAG-CAGCAGCGGTTTTC------ACTTGC-TGCTCTT-CCTCTGCGCGCTC---TCCC

CGGTACCT

#### ITS2 reference sequences

>MH390370.1-Chlorocebus_aethiops-China Trichuris sp. Ca2 internal transcribed spacer 1, partial sequence; 5.8S ribosomal RNA gene, complete sequence; and internal transcribed spacer 2, partial sequence

TCGGTCGAGCGGTGCCCGCCGCCGCAGCAAGGCGAACGGTTTCGTCGTCGTCGTCGACTGCTGCTGCCGCTGCTGCTGCC

GTGTAACTACGACGAACACTTGCTGCTACGAGCGCGTGCGGTCACCGCTCCCTGCTCAACTGCTGTACGCCGACGGCGCG

GCCGGTAGCCAAGTCGGGTAGTCTTCGCCCTACGCTGACTTGGCCGCTCTCCGCTGCTCCGCCTACCAGTTGCCATCGCG

GCTCGCTGGCAACGGAGCTGCGGCGTCACGCTCGCTCGCGCGCGGATGCAGGTTTGTCCGGTGCTCGCCACCAACCTCAA

CCTGTAGCCTGAGCGGCGCATTCGCTATTATGCTTAACGACCCTCAGACAGGCGTAGCGTCGACATCTTTGATCGACGCT

GCAATGTGCGTTCAAAGTATTCAAGTTCGGTGTGTCTGCAATTCACGTCAATTCTCGAGTGTCACGTCGTTCTTCAACGA

TCTACGAGCCAAGTGATCCACCGTTCGGAATGTACTTTCCTGCGCGTACAGCTGCAGTTCGAATGCTACTAGCGCTCATT

GCGCCGACAGCAGTGTCTGCGTGTCGAAGGCACATCGGCGTGCGTAGTAGGACGACGCCTTGTGCACTGGTGAAGCTGCT

TTTTC

>KT344827.1-Chlorocebus_aethiops-China Trichuris sp. SC3 internal transcribed spacer 1, partial sequence; 5.8S ribosomal RNA gene, complete sequence; and internal transcribed spacer 2, partial sequence

TCGGTCGAGCGGTGCCCGCCGCCGCAGCAAGGCGAACGGTTTCGTCGTCGTCGTCGACTGCTGCTGCCGCTGCTGCTGCC

GTGTAACTACGACGAACACTTGCTGCTACGAGCGCGTGCGGTCACCGCTCCCTGCTCAACTGCTGTACGCCGACGGCGCG

GCCGGTAGCCAAGTCGGGTAGTCTTCGCCCTACGCTGACTTGGCCGCTCTCCGCTGCTCCGCCTACCAGTTGCCATCGCG

GCTCGCTGGCAACGGAGCTGCGGCGTCACGCTCGCTCGCGCGCGGATGCAGGTTTGTCCGGTGCTCGCCACCAACCTCAA

CCTGTAGCCTGAGCGGCGCATTCGCTATTATGCTTAACGACCCTCAGACAGGCGTAGCGTCGACATCTTTGATCGACGCT

GCAATGTGCGTTCAAAGTATTCAAGTTCGGTGTGTCTGCAATTCACGTCAATTCTCGAGTGTCACGTCGTTCTTCAACGA

TCTACGAGCCAAGTGATCCACCGTTCGGAATGTACTTTCCTGCGCGTACAGCTGCAGTTCGAATGCTACTAGCGCTCATT

GCGCCGACAGCAGTGTCTGCGTGTCGAAGGCACATCGGCGTGCGTAGTAGGACGACGCCTTGTGCACTGGTGAAGCTGCT

TTTTC

>AM992995.1-Unknown-China Trichuris trichiura ITS1 (partial), 5.8S rRNA gene and ITS2 (partial), isolate Ttr7c

TCGGTCGAGCGGTGCCCGCCGCCGCAGCAAGGCGAACGGTTTCGTCGTCGTCGTCGACTGCTGCTGCCGCTGCTGCTGCC

GTGCAACGACGACGAACACTTGCTGCTACGAGCGCGTGCGGTCACCGCTCCCTGCTCAACTGCTGTACGCCGACGGCGCG

GCCGGTAGCCAAGTCGGGTAGTCTTCGCCCTACGCTGACTTGGCCGCTCTCCGCTGCTCCGCCTACCAGTTGCCATCGCG

GCTCGCTGGCAACGGAGCTGCGGCGTCACGCTCGCTCGCGCGCGGATGCAGGTTTGTCCGGTGCTCGCCACCAACCTCAA

CCTGTAGCCTGAGCGGCGCATTCGCTATTATGCTTAACGACCCTCAGACAGGCGTAGCGTCGACATCTTTGATCGACGCT

GCAATGTGCGTTCAAAGTATTCAAGTTCGGTGTGTCTGCAATTCACGTCAATTCTCGAGTGTCACGTCGTTCTTCAACGA

TCTACGAGCCAAGTGATCCACCGTTCGGAATGTACTTTCCTGCGCGTACAGCAGCAGTTCGAATGCTACTAGCGCTCATT

GCGCCGACAGCAGTGTCTGCGTGTCGAAGGCACATCGGCGTGCGTAGTAGGACGACGCCTTGTGCACTGGTGAAGCTGCT

TTTTC

>AM992984.1-Unknown-China Trichuris trichiura ITS1 (partial), 5.8S rRNA gene and ITS2 (partial), isolate Ttr3a

TCGGTCGAGCGGTGCCCGCCGCCGCAGCAAGGCGAACGGTTTCGTCGTCGTCGTCGACTGCTGCTGCCGCTGCTGCTGCC

GTGCAACGACGACGAACACTTGCTGCTACGAGCGCGTGCGGTCACCGCTCCCTGCTCAACTGCTGTACGCCGACGGCGCG

GCCGGTAGCCAAGTCGGGTAGTCTTCGCCCTACGCTGACTTGGCCGCTCTCCGCTGCTCCGCCTACCAGTTGCCATCGCG

GCTCGCTGGCAACGGAGCTGCGGCGTCACGCTCGCTCGCGCGCGGATGCAGGTTTGTCCGGTGCTCGCCACCAACCTCAA

CCTGTAGCCTGAGCGGCGCATTCGCTATTATGCTTAACGACCCTCAGACAGGCGTAGCGTCGACATCTTTGATCGACGCT

GCAATGTGCGTTCAAAGTATTCAAGTTCGGTGTGTCTGCAATTCACGTCAATTCTCGAGTGTCACGTCGTTCTTCAACGA

TCTACGAGCCAAGTGATCCACCGTTCGGAATGTACTTTCCTGCGCGTACAGCAGCAGTTCGAATGCTACTAGCGCTCATT

GCGCCGACAGCAGTGTCTGCGTGTCGAAGGCACATCGGCGTGCGTAGTAGGACGACGCCTTGTGCACTGGTGAAGCTGCT

TTTTC

>AM992997.1-Unknown-China Trichuris trichiura ITS1 (partial), 5.8S rRNA gene and ITS2 (partial), isolate Ttr8b

TCGGTCGAGCGGTGCCCGCCGCCGCAGCAAGGCGAACGGTTTCGTCGTCGTCGTCGACTGCTGCTGCTGCTGCTGCTGCC

GTGCAACTACGACGAACACTTGCTGCTACGAGCGCGTGCGGTCACCGCTCCCTGCTCAACTGCTGTACGCCGACGGCGCG

GCCGGTAGCCAAGTCGGGTAGTCTTCGCCCTACGCTGACTTGGCCGCTCTCCGCTGCTCCGCCTACCAGTTGCCATCGCG

GCTCGCTGGCAACGGAGCTGCGGCGTCACGCTCGCTCGCGCGCGGATGCAGGTTTGTCCGGTGCTCGCCACCAACCTCAA

CCTGTAGCCTGAGCGGCGCATTCGCTATTATGCTTAACGACCCTCAGACAGGCGTAGCGTCGACATCTTTGATCGACGCT

GCAATGTGCGTTCAAAGTATTCAAGTTCGGTGTGTCTGCAATTCACGTCAATTCTCGAGTGTCACGTCGTTCTTCAACGA

TCTACGAGCCAAGTGATCCACCGTTCGGAATGTACTTTCCTGCGCGTACAGCAGCAGTTCGAATGCTACTAGCGCTCATT

GCGCCGACAGCAGTGTCTGCGTGTCGAAGGCACATCGGCGTGCGTAGTAGGACGACGCCTTGTGCACTGGTGAAGCTGCT

TTTTC

>KC877992.1-Papio_hamadryas-Turkey Trichuris trichiura voucher OMUPAR.53.12.01 internal transcribed spacer 1, partial sequence; 5.8S ribosomal RNA gene, complete sequence; and internal transcribed spacer 2, partial sequence

TCGGTCGAGCGGTGCCCGCCGCCGCAGCAAGGGGAACGGTTTCGTCGTCGTCGTCGACTGCTGCTGCCGCTGCTGCTGCC

GTGCAACTACGACGAACACTTGCTGCTACGAGCGCGTGCGGTCACCGCTCCCTGCTCAACTGCTGTACGCCGACGGCGCG

GCCGGTAGCCAAGTCGGGTAGTCTTCGCCCTACGCTGACTTGGCCGCTCTCCGCTGCTCCGCCTACCAGTTGCCATCGCG

GCTCGCTGGCAACGGAGCTGCGGCGTCACGCTCGCTCGCGCGCGGATGCAGGTTTGTCCGGTGCTCGCCACCAACCTCAA

CCTGTAGCCTGAGCGGCGCATTCGCTATTATGCTTAACGACCCTCAGACAGGCGTAGCGTCGACATCTTTGATCGACGCT

GCAATGTGCGTTCAAAGTATTCAAGTTCGGTGTGTCTGCAATTCACGTCAATTCTCGAGTGTCACGTCGTTCTTCAACGA

TCTACGAGCCAAGTGATCCACCGTTCGGAATGTACTTTCCTGCGCGTACAGCAGCAGTTCGAATGCTACTAGCGCTCATT

GCGCCGACAGCAGTGTCTGCGTGTCGAAGGCACATCGGCGTGCGTAGTAGGACGACGCCTTGTGCACTGGTGAAGCTGCT

TTTTC

>MH390361.1-Papio_anubis-China Trichuris sp. Pa4 internal transcribed spacer 1, partial sequence; 5.8S ribosomal RNA gene, complete sequence; and internal transcribed spacer 2, partial sequence

TCGGTCGAGCGGTGCCCGCCGCCGCAGCAAGGCGAACGGTTTCGTCGTCGTCGTCGACTGCTGCTGCCGCTGCTGCTGCC

GTGCAACTACGACGAACACTTGCTGCTACGAGCGCGTGCGGTCACCGCTCCCTGCTCAACTGCTGTACGCCGACGGCGCG

GCCGGTAGCCAAGTCGGGTAGTCTTCGCCCTACGCTGACTTGGCCGCTCTCCGCTGCTCCGCCTACCAGTTGCCATCGCG

GCTCGCTGGCAACGGAGCTGCGGCGTCACGCTCGCTCGCGCGCGGATGCAGGTTTGTCCGGTGCTCGCCACCAACCTCAA

CCTGTAGCCTGAGCGGCGCATTCGCTATTATGCTTAACGACCCTCAGACAGGCGTAGCGTCGACATCTTTGATCGACGCT

GCAATGTGCGTTCAAAGTATTCAAGTTCGGTGTGTCTGCAATTCACGTCAATTCTCGAGTGTCACGTCGTTCTTCAACGA

TCTACGAGCCAAGTGATCCACCGTTCGGAATGTACTTTCCTGCGCGTACAGCAGCAGTTCGAATGATACTAGCGCTCATT

GCGCCGACAGCAGTGTCTGCGTGTCGAAGGCACATCGGCGTGCGTAGTAGGACGACGCCTTGTGCACTGGTGAAGCCGCT

TTTTC

>MH390359.1-Papio_anubis-China Trichuris sp. Pa2 internal transcribed spacer 1, partial sequence; 5.8S ribosomal RNA gene, complete sequence; and internal transcribed spacer 2, partial sequence

TCGGTCGAGCGGTGCCCGCCGCCGCAGCAAGGCGAACGGTTTCGTCGTCGTCGTCGACTGCTGCTGCCGCTGCTGCTGCC

GTGCAACTACGACGAACACTTGCTGCTACGAGCGCGTGCGGTCACCGCTCCCTGCTCAACTGCTGTACGCCGACGGCGCG

GCCGGTAGCCAAGTCGGGTAGTCTTCGCCCTACGCTGACTTGGCCGCTCTCCGCTGCTCCGCCTACCAGTTGCCATCGCG

GCTCGCTGGCAACGGAGCTGCGGCGTCACGCTCGCTCGCGCGCGGATGCAGGTTTGTCCGGTGCTCGCCACCAACCTCAA

CCTGTAGCCTGAGCGGCGCATTCGCTATTATGCTTAACGACCCTCAGACAGGCGTAGCGTCGACATCTTTGATCGACGCT

GCAATGTGCGTTCAAAGTATTCAAGTTCGGTGTGTCTGCAATTCACGTCAATTCTCGAGTGTCACGTCGTTCTTCAACGA

TCTACGAGCCAAGTGATCCACCGTTCGGAATGTACTTTCCTGCGCGTACAGCAGCAGTTCGAATGATACTAGCGCTCATT

GCGCCGACAGCAGTGTCTGCGTGTCGAAGGCACATCGGCGTGCGTAGTAGGACGACGCCTTGTGCACTGGTGAAGCCGCT

TTTTC

>KT344826.1-Papio_anubis-China Trichuris sp. SC2 internal transcribed spacer 1, partial sequence; 5.8S ribosomal RNA gene, complete sequence; and internal transcribed spacer 2, partial sequence

TCGGTCGAGCGGTGCCCGCCGCCGCAGCAAGGCGAACGGTTTCGTCGTCGTCGTCGACTGCTGCTGCCGCTGCTGCTGCC

GTGCAACTACGACGAACACTTGCTGCTACGAGCGCGTGCGGTCACCGCTCCCTGCTCAACTGCTGTACGCCGACGGCGCG

GCCGGTAGCCAAGTCGGGTAGTCTTCGCCCTACGCTGACTTGGCCGCTCTCCGCTGCTCCGCCTACCAGTTGCCATCGCG

GCTCGCTGGCAACGGAGCTGCGGCGTCACGCTCGCTCGCGCGCGGATGCAGGTTTGTCCGGTGCTCGCCACCAACCTCAA

CCTGTAGCCTGAGCGGCGCATTCGCTATTATGCTTAACGACCCTCAGACAGGCGTAGCGTCGACATCTTTGATCGACGCT

GCAATGTGCGTTCAAAGTATTCAAGTTCGGTGTGTCTGCAATTCACGTCAATTCTCGAGTGTCACGTCGTTCTTCAACGA

TCTACGAGCCAAGTGATCCACCGTTCGGAATGTACTTTCCTGCGCGTACAGCAGCAGTTCGAATGATACTAGCGCTCATT

GCGCCGACAGCAGTGTCTGCGTGTCGAAGGCACATCGGCGTGCGTAGTAGGACGACGCCTTGTGCACTGGTGAAGCCGCT

TTTTC

>MH390360.1-Papio_anubis-China Trichuris sp. Pa3 internal transcribed spacer 1, partial sequence; 5.8S ribosomal RNA gene, complete sequence; and internal transcribed spacer 2, partial sequence

TCGGTCGAGCGGTGCCCGCCGCCGCAGCAAGGCGAACGGTTTCGTCGTCGTCGTCGACTGCTGCTGCCGCTGCTGCTGCC

GTGCAACTACGACGAACACTTGCTGCTACGAGCGCGTGCGGTCACCGCTCCCTGCTCAACTGCTGTACGCCGACGGCGCG

GCCGGTAGCCAAGTCGGGTAGTCTTCGCCCTACGCTGACTTGGCCGCTCTCCGCTGCTCCGCCTACCAGTTGCCATCGCG

GCTCGCTGGCAACGGAGCTGCGGCGTCACGCTCGCTCGCGCGCGGATGCAGGTTTGTCCGGTGCTCGCCACCAACCTCAA

CCTGTAGCCTGAGCGGCGCATTCGCTATTATGCTTAACGACCCTCAGACAGGCGTAGCGTCGACATCTTTGATCGACGCT

GCAATGTGCGTTCAAAGTATTCAAGTTCGGTGTGTCTGCAATTCACGTCAATTCTCGAGTGTCACGTCGTTCTTCAACGA

TCTACGAGCCAAGTGATCCACCGTTCGGAATGTACTTTCCTGCGCGTACAGCAGCAGTTCGAATGATACTAGCGCTCATT

GCGCCGACAGCAGTGTCTGCGTGTCGAAGGCACATCGGCGTGCGTAGTAGGACGACGCCTTGTGCACTGGTGAAGCCGCT

TTTTC

>KT344830.1-Nomascus_leucogenys-China Trichuris sp. SC6 internal transcribed spacer 1, partial sequence; 5.8S ribosomal RNA gene, complete sequence; and internal transcribed spacer 2, partial sequence

TCGGTCGAGCGGTGCCCGCCGCCGCAGCAAGGCGAACGGTTTCGTCGTCGTCGACTGCTGCTGCCGCTGCTGCTGCCGTG

CAACTACGACGAACACTTGCTGCTACGAGCGCGTGCGGTCACCGCTCCCTGCTCAACTGCTGTACGCCGACGGCGCGGCC

GGTAGCCAAGTCGGGTAGTCTTCGCCCTACGCTGACTTGGCCGCTCTCCGCTGCTCCGCCTACCAGTTGCCATCGCGGCT

CGCTGGCAACGGAGCTGCGGCGTCACGCTCGCTCGCGCGCGGATGCAGGTTTGTCCGGTGCTCGCCACCAACCTCAACCT

GTAGCCTGAGCGGCGCATTCGCTATTATGCTTAACGACCCTCAGACAGGCGTAGCGTCGACATCTTTGATCGACGCTGCA

ATGTGCGTTCAAAGTATTCAAGTTCGGTGTGTCTGCAATTCACGTCAATTCTCGAGTGTCACGTCGTTCTTCAACGATCT

ACGAGCCAAGTGATCCACCGTTCGGAATGTACTTTCCTGCGCGTACAGCAGCAGTTCGAATGCTACTAGCGCTCATTGCG

CCGACAGCAGTGTCTGCGTGTCGAAGGCACATCGGCGTGCGTAGTAGGACGACGCCTTGTGCACTGGTGAAGCTGCCTTT

TC

>MH390366.1-Nomascus_leucogenys-China Trichuris sp. Nl2 internal transcribed spacer 1, partial sequence; 5.8S ribosomal RNA gene, complete sequence; and internal transcribed spacer 2, partial sequence

TCGGTCGAGCGGTGCCCGCCGCCGCAGCAAGGCGAACGGTTTCGTCGTCGTCGACTGCTGCTGCCGCTGCTGCTGCCGTG

CAACTACGACGAACACTTGCTGCTACGAGCGCGTGCGGTCACCGCTCCCTGCTCAACTGCTGTACGCCGACGGCGCGGCC

GGTAGCCAAGTCGGGTAGTCTTCGCCCTACGCTGACTTGGCCGCTCTCCGCTGCTCCGCCTACCAGTTGCCATCGCGGCT

CGCTGGCAACGGAGCTGCGGCGTCACGCTCGCTCGCGCGCGGATGCAGGTTTGTCCGGTGCTCGCCACCAACCTCAACCT

GTAGCCTGAGCGGCGCATTCGCTATTATGCTTAACGACCCTCAGACAGGCGTAGCGTCGACATCTTTGATCGACGCTGCA

ATGTGCGTTCAAAGTATTCAAGTTCGGTGTGTCTGCAATTCACGTCAATTCTCGAGTGTCACGTCGTTCTTCAACGATCT

ACGAGCCAAGTGATCCACCGTTCGGAATGTACTTTCCTGCGCGTACAGCAGCAGTTCGAATGCTACTAGCGCTCATTGCG

CCGACAGCAGTGTCTGCGTGTCGAAGGCACATCGGCGTGCGTAGTAGGACGACGCCTTGTGCACTGGTGAAGCTGCCTTT

TC

>MH390363.1-Papio_hamadryas-China Trichuris sp. Ph2 internal transcribed spacer 1, partial sequence; 5.8S ribosomal RNA gene, complete sequence; and internal transcribed spacer 2, partial sequence

TCGGTCGAGCGGTGCCCGCCGCCGCAGCAAGGCGAACGGTTTCGTCGTCGTCGACTGCTGCTGCCGCTGCTGCTGCCGTG

CAACTACGACGAACACTTGCTGCTACGAGCGCGTGCGGTCACCGCTCCCTGCTCAACTGCTGTACGCCGACGGCGCGGCC

GGTAGCCAAGTCGGGTAGTCTTCGCCCTACGCTGACTTGGCCGCTCTCCGCTGCTCCGCCTACCAGTTGCCATCGCGGCT

CGCTGGCAACGGAGCTGCGGCGTCACGCTCGCTCGCGCGCGGATGCAGGTTTGTCCGGTGCTCGCCACCAACCTCAACCT

GTAGCCTGAGCGGCGCATTCGCTATTATGCTTAACGACCCTCAGACAGGCGTAGCGTCGACATCTTTGATCGACGCTGCA

ATGTGCGTTCAAAGTATTCAAGTTCGGTGTGTCTGCAATTCACGTCAATTCTCGAGTGTCACGTCGTTCTTCAACGATCT

ACGAGCCAAGTGATCCACCGTTCGGAATGTACTTTCCTGCGCGTACAGCAGCAGTTCGAATGCTACTAGCGCTCATTGCG

CCGACAGCAGTGTCTGCGTGTCGAAGGCACATCGGCGTGCGTAGTAGGACGACGCCTTGTGCACTGGTGAAGCTGCCTTT

TC

>AM992993.1-Unknown-China Trichuris trichiura ITS1 (partial), 5.8S rRNA gene and ITS2 (partial), isolate Ttr7a

TCGGTCGAGCGGTGCCCGCCGCCGCAGCAAGGCGAACGGTTTCGTCGTCGTCGACTGCTGCTGCCGCTGCTGCTGCCGTG

CAACGACGACGAACACTTGCTGCTACGAGCGCGTGCGGTCACCGCTCCCTGCTCAACTGCTGTACGCCGACGGCGCGGCC

GGTAGCCAAGTCGGGTAGTCTTCGCCCTACGCTGACTTGGCCGCTCTCCGCTGCTCCGCCTACCAGTTGCCATCGCGGCT

CGCTGGCAACGGAGCTGCGGCGTCACGCTCGCTCGCGCGCGGATGCAGGTTTGTCCGGTGCTCGCCACCAACCTCAACCT

GTAGCCTGAGCGGCGCATTCGCTATTATGCTTAACGACCCTCAGACAGGCGTAGCGTCGACATCTTTGATCGACGCTGCA

ATGTGCGTTCAAAGTATTCAAGTTCGGTGTGTCTGCAATTCACGTCAATTCTCGAGTGTCACGTCGTTCTTCAACGATCT

ACGAGCCAAGTGATCCACCGTTCGGAATGTACTTTCCTGCGCGTACAGCAGCAGTTCGAATGCTACTAGCGCTCATTGCG

CCGACAGCAGTGTCTGCGTGTCGAAGGCACATCGGCGTGCGTAGTAGGACGACGCCTTGTGCACTGGTGAAGCTGCTTTT

TC

>AM992985.1-Unknown-China Trichuris trichiura ITS1 (partial), 5.8S rRNA gene and ITS2 (partial), isolate Ttr3b

TCGGTCGAGCGGTGCCCGCCGCCGCAGCAAGGCGAACGGTTTCGTCGTCGTCGTCGACTGCTGCTGCCGCTGCTGCCGTG

CAACGACGACGAACACTTGCTGCTACGAGCGCGTGCGGTCACCGCTCCCTGCTCAACTGCTGTACGCCGACGGCGCGGCC

GGTAGCCAAGTCGGGTAGTCTTCGCCCTACGCTGACTTGGCCGCTCTCCGCTGCTCCGCCTACCAGTTGCCATCGCGGCT

CGCTGGCAACGGAGCTGCGGCGTCACGCTCGCTCGCGCGCGGATGCAGGTTTGTCCGGTGCTCGCCACCAACCTCAACCT

GTAGCCTGAGCGGCGCATTCGCTATTATGCTTAACGACCCTCAGACAGGCGTAGCGTCGACATCTTTGATCGACGCTGCA

ATGTGCGTTCAAAGTATTCAAGTTCGGTGTGTCTGCAATTCACGTCAATTCTCGAGTGTCACGTCGTTCTTCAACGATCT

ACGAGCCAAGTGATCCACCGTTCGGAATGTACTTTCCTGCGCGTACAGCAGCAGTTCGAATGCTACTAGCGCTCATTGCG

CCGACAGCAGTGTCTGCGTGTCGAAGGCACATCGGCGTGCGTAGTAGGACGACGCCTTGTGCACTGGTGAAGCTGCTTTT

TC

>AM992986.1-Unknown-China Trichuris trichiura ITS1 (partial), 5.8S rRNA gene and ITS2 (partial), isolate Ttr3c

TCGGTCGAGCGGTGCCCGCCGCCGCAGCAAGGCGAACGGTTTCGTCGTCGTCGTCGACTGCTGCTGCCGCTGCTGCCGTG

CAACGACGACGAACACTTGCTGCTACGAGCGCGTGCGGTCACCGCTCCCTGCTCAACTGCTGTACGCCGACGGCGCGGCC

GGTAGCCAAGTCGGGTAGTCTTCGCCCTACGCTGACTTGGCCGCTCTCCGCTGCTCCGCCTACCAGTTGCCATCGCGGCT

CGCTGGCAACGGAGCTGCGGCGTCACGCTCGCTCGCGCGCGGATGCAGGTTTGTCCGGTGCTCGCCACCAACCTCAACCT

GTAGCCTGAGCGGCGCATTCGCTATTATGCTTAACGACCCTCAGACAGGCGTAGCGTCGACATCTTTGATCGACGCTGCA

ATGTGCGTTCAAAGTATTCAAGTTCGGTGTGTCTGCAATTCACGTCAATTCTCGAGTGTCACGTCGTTCTTCAACGATCT

ACGAGCCAAGTGATCCACCGTTCGGAATGTACTTTCCTGCGCGTACAGCAGCAGTTCGAATGCTACTAGCGCTCATTGCG

CCGACAGCAGTGTCTGCGTGTCGAAGGCACATCGGCGTGCGTAGTAGGACGACGCCTTGTGCACTGGTGAAGCTGCTTTT

TC

>AM992991.1-Unknown-China Trichuris trichiura ITS1 (partial), 5.8S rRNA gene and ITS2 (partial), isolate Ttr6b

TCGGTCGAGCGGTGCCCGCCGCCGCAGCAAGGCGAACGGTTTCGTCGTCGTCGTCGACTGCTGCTGCCGCTGCTGCCGTG

CAACGACGACGAACACTTGCTGCTACGAGCGCGTGCGGTCACCGCTCCCTGCTCAGCTGCTGAACGCCGACGGCGCGGCC

GGTAGCCAAGTCGGGTAGTCTTCGCCCTACGCTGACTTGGCCGCTCTCCGCTGCTCCGCCTACCAGTTGCCATCGCGGCT

CGCTGGCAACGGAGCTGCGGCGTCACGCTCGCTCGCGCGCGGATGCAGGTTTGTCCGGTGCTCGCCACCAACCTCAACCT

GTAGCCTGAGCGGCGCATTCGCTATTATGCTTAACGACCCTCAGACAGGCGTAGCGTCGACATCTTTGATCGACGCTGCA

ATGTGCGTTCAAAGTATTCAAGTTCGGTGTGTCTGCAATTCACGTCAATTCTCGAGTGTCACGTCGTTCTTCAACGATCT

ACGAGCCAAGTGATCCACCGTTCGGAATGTACTTTCCTGCGCGTACAGCAGCAGTTCGAATGCTACTAGCGCTCATTGCG

CCGACAGCAGTGTCTGCGTGTCGAAGGCACATCGGCGTGCGTAGTAGGACGACGCCTTGTGCACTGGTGAAGCTGCCTTT

TC

>AM992994.1-Unknown-China Trichuris trichiura ITS1 (partial), 5.8S rRNA gene and ITS2 (partial), isolate Ttr7b

TCGGTCGAGCGGTGCCCGCCGCCGCAGCAAGGCGAACGGTTTCGTCGTCGTCGACTGCTGCTGCCGCTGCTGCCGTGCAA

CGACGACGAACACTTGCTGCTACGAGCGCGTGCGGTCACCGCTCCCTGCTCAACTGCTGTACGCCGACGGCGCGGCCGGT

AGCCAAGTCGGGTAGTCTTCGCCCTACGCTGACTTGGCCGCTCTCCGCTGCTCCGCCTACCAGTTGCCATCGCGGCTCGC

TGGCAACGGAGCTGCGGCGTCACGCTCGCTCGCGCGCGGATGCAGGTTTGTCCGGTGCTCGCCACCAACCTCAACCTGTA

GCCTGAGCGGCGCATTCGCTATTATGCTTAACGACCCTCAGACAGGCGTAGCGTCGACATCTTTGATCGACGCTGCAATG

TGCGTTCAAAGTATTCAAGTTCGGTGTGTCTGCAATTCACGTCAATTCTCGAGTGTCACGTCGTTCTTCAACGATCTACG

AGCCAAGTGATCCACCGTTCGGAATGTACTTTCCTGCGCGTACAGCAGCAGTTCGAATGCTACTAGCGCTCATTGCGCCG

ACAGCAGTGTCTGCGTGTCGAAGGCACATCGGCGTGCGTAGTAGGACGACGCCTTGTGCACTGGTGAAGCTGCTTTTTC

>MN447327.1-Macaca_mulatta-China Trichuris sp. Mm3 GY-2019 internal transcribed spacer 1, partial sequence; 5.8S ribosomal RNA gene and internal transcribed spacer 2, complete sequence; and large subunit ribosomal RNA gene, partial sequence

TCGGTCGAGCGGTGCCCGCCGCCGCAGCAAGGCGAACGGTTTCGTCGTCGTCGTCGTCGACTGCTGCTGCCGCTGCTGCC

GTGCAACGACGACGAACACTTGCTGCTACGAGCGCGTGCGATCACCGCTCCCTGCTCAACTGCTGTACGCCGACGGCGCG

GCCGGTAGCCAAGTCGGGTAGTCTTCGCCCTACGCTGACTTGGCCGCTCTCCGCTGCTCCGCCTACCAGTTGCCATCGCG

GCTCGCTGGCAACGGAGCTGCGGCGTCACGCTCGCTCGCGCGCGGATGCAGGTTTGTCCGGTGCTCGCCACCAACCTCAA

CCTGTAGCCTGAGCGGCGCATTCGCTATTATGCTTAACGACCCTCAGACAGGCGTAGCGTCGACATCTTTGATCGACGCT

GCAATGTGCGTTCAAAGTATTCAAGTTCGGTGTGTCTGCAATTCACGTCAATTCTCGAGTGTCACGTCGTTCTTCAACGA

TCTACGAGCCAAGTGATCCACCGTTCGGAATGTACTTTCCTGCGCGTACAGCTGCAGTTCGAATGCTACTAGCGCTCATT

GCGCCGACAGCAGTGTCTGCGTGTCGAAGGCACATCGGCGTGCGTAGTAGGACGACGCCTTGTGCACTGGTGAAGCTGCT

TTTTC

>AM992992.1-Unknown-China Trichuris trichiura ITS1 (partial), 5.8S rRNA gene and ITS2 (partial), isolate Ttr6c

TCGGTCGAGCGGTGCCCGCCGCCGCAGCAAGGCGAACGGTTTCGTCGTCGTCGTCGTCGACTGCTGCTGCCGCTGCTGCC

GTGCAACGACGACGAACACTTGCTGCTACGAGCGCGTGCGGTCACCGCTCCCTGCTCAGCTGCTGAACGCCGACGGCGCG

GCCGGTAGCCAAGTCGGGTAGTCTTCGCCCTACGCTGACTTGGCCGCTCTCCGCTGCTCCGCCTACCAGTTGCCATCGCG

GCTCGCTGGCAACGGAGCTGCGGCGTCACGCTCGCTCGCGCGCGGATGCAGGTTTGTCCGGTGCTCGCCACCAACCTCAA

CCTGTAGCCTGAGCGGCGCATTCGCTATTATGCTTAACGACCCTCAGACAGGCGTAGCGTCGACATCTTTGATCGACGCT

GCAATGTGCGTTCAAAGTATTCAAGTTCGGTGTGTCTGCAATTCACGTCAATTCTCGAGTGTCACGTCGTTCTTCAACGA

TCTACGAGCCAAGTGATCCACCGTTCGGAATGTACTTTCCTGCGCGTACAGCAGCAGTTCGAATGCTACTAGCGCTCATT

GCGCCGACAGCAGTGTCTGCGTGTCGAAGGCACATCGGCGTGCGTAGTAGGACGACGCCTTGTGCACTGGTGAAGCTGCC

TTTTC

>AM992990.1-Unknown-China Trichuris trichiura ITS1 (partial), 5.8S rRNA gene and ITS2 (partial), isolate Ttr6a

TCGGTCGAGCGGTGCCCGCCGCCGCAGCAAGGCGAACGGTTTCGTCGTCGTCGTCGTCGACTGCTGCTGCCGCTGCTGCC

GTGCAACGACGACGAACACTTGCTGCTACGAGCGCGTGCGGTCACCGCTCCCTGCTCAGCTGCTGAACGCCGACGGCGCG

GCCGGTAGCCAAGTCGGGTAGTCTTCGCCCTACGCTGACTTGGCCGCTCTCCGCTGCTCCGCCTACCAGTTGCCATCGCG

GCTCGCTGGCAACGGAGCTGCGGCGTCACGCTCGCTCGCGCGCGGATGCAGGTTTGTCCGGTGCTCGCCACCAACCTCAA

CCTGTAGCCTGAGCGGCGCATTCGCTATTATGCTTAACGACCCTCAGACAGGCGTAGCGTCGACATCTTTGATCGACGCT

GCAATGTGCGTTCAAAGTATTCAAGTTCGGTGTGTCTGCAATTCACGTCAATTCTCGAGTGTCACGTCGTTCTTCAACGA

TCTACGAGCCAAGTGATCCACCGTTCGGAATGTACTTTCCTGCGCGTACAGCAGCAGTTCGAATGCTACTAGCGCTCATT

GCGCCGACAGCAGTGTCTGCGTGTCGAAGGCACATCGGCGTGCGTAGTAGGACGACGCCTTGTGCACTGGTGAAGCTGCC

TTTTC

>GQ301551.1-Papio_hamadryas_ursinus-South_Africa Trichuris sp. ex Papio ursinus NL-2009 isolate DGIII internal transcribed spacer 1, partial sequence; 5.8S ribosomal RNA gene, complete sequence; and internal transcribed spacer 2, partial sequence

TCGGTCGAGCGGTGCCCGCCGCCGCAGCAAGGCGAACGGTTTCGTCGTCGTCGTCGACTGCTGCTGCCGCTGCTGCTGCT

GCCGTGCAACTACGACGAACACTTGCTGCTACGAGCGCGTGCGGTCACCGCTCCCTGCTCAACTGCTGTACGCCGACGGC

GCGGCCGGTAGCCAAGTCGGGTAGTCTTCGCCCTACGCTGACTTGGCCGCTCTCCGCTGCTCCGCCTACCAGTTGCCATC

GCGGCTCGCTGGCAACGGAGCTGCGGCGTCACGCTCGCTCGCGCGCGGATGCAGGTTTGTCCGGTGCTCGCCACCAACCT

CAACCTGTAGCCTGAGCGGCGCATTCGCTATTATGCTTAACGACCCTCAGACAGGCGTAGCGTCGACATCTTTGATCGAC

GCTGCAATGTGCGTTCAAAGTATTCAAGTTCGGTGTGTCTGCAATTCACGTCAATTCTCGAGTGTCACGTCGTTCTTCAA

CGATCTACGAGCCAAGTGATCCACCGTTCGGAATGTACTTTCCTGCGCGTACAGCAGCAGTTCGAATGCTACTAGCGCTC

ATTGCGCCGACAGCAGTGTCTGCGTGTCAAAGGCACATCGGCGTGCGTAGTAGGACGACGCCTTGTGCACTGGTGAAGCT

GCTTTTTC

>AM992981.1-Unknown-China Trichuris trichiura ITS1 (partial), 5.8S rRNA gene and ITS2 (partial), isolate Ttr2a

TCGGTCGAGCGGTGCCCGCCGCCGCAGCAAGGCGAACGGTTTCGTCGTCGTCGTCGTCGACTGCTGCTGCTGCTGCTGCT

GCCGTGCAACGACGACGAACACTTGCTGCTACGAGCGCGTGCGGTCACCGCTCCCTGCTCAACTGCTGTACGCCGACGGC

GCGGCCGGTAGCCAAGTCGGGTAGTCTTCGCCCTACGCTGACTTGGCCGCTCTCCGCTGCTCCGCCTACCAGTTGCCATC

GCGGCTCGCTGGCAACGGAGCTGCGGCGTCACGCTCGCTCGCGCGCGGATGCAGGTTTGTCCGGTGCTCGCCACCAACCT

CAACCTGTAGCCTGAGCGGCGCATTCGCTATTATGCTTAACGACCCTCAGACAGGCGTAGCGTCGACATCTTTGATCGAC

GCTGCAATGTGCGTTCAAAGTATTCAAGTTCGGTGTGTCTGCAATTCACGTCAATTCTCGAGTGTCACGTCGTTCTTCAA

CGATCTACGAGCCAAGTGATCCACCGTTCGGAATGTACTTTCCTGCGCGTACAGCAGCAGTTCGAATGCTACTAGCGCTC

ATTGCGCCGACAGCAGTGTCTGCGTGTCGAAGGCACATCGGCGTGCGTAGTAGGACGACGCCTTGTGCACTGGTGAAGCT

GCTTTTTC

>AM992983.1-Unknown-China Trichuris trichiura ITS1 (partial), 5.8S rRNA gene and ITS2 (partial), isolate Ttr2c

TCGGTCGAGCGGTGCCCGCCGCCGCAGCAAGGCGAACGGTTTCGTCGTCGTCGTCGTCGACTGCTGCTGCTGCTGCTGCT

GCCGTGCAACGACGACGAACACTTGCTGCTACGAGCGCGTGCGGTCACCGCTCCCTGCTCAACTGCTGTACGCCGACGGC

GCGGCCGGTAGCCAAGTCGGGTAGTCTTCGCCCTACGCTGACTTGGCCGCTCTCCGCTGCTCCGCCTACCAGTTGCCATC

GCGGCTCGCTGGCAACGGAGCTGCGGCGTCACGCTCGCTCGCGCGCGGATGCAGGTTTGTCCGGTGCTCGCCACCAACCT

CAACCTGTAGCCTGAGCGGCGCATTCGCTATTATGCTTAACGACCCTCAGACAGGCGTAGCGTCGACATCTTTGATCGAC

GCTGCAATGTGCGTTCAAAGTATTCAAGTTCGGTGTGTCTGCAATTCACGTCAATTCTCGAGTGTCACGTCGTTCTTCAA

CGATCTACGAGCCAAGTGATCCACCGTTCGGAATGTACTTTCCTGCGCGTACAGCAGCAGTTCGAATGCTACTAGCGCTC

ATTGCGCCGACAGCAGTGTCTGCGTGTCGAAGGCACATCGGCGTGCGTAGTAGGACGACGCCTTGTGCACTGGTGAAGCT

GCTTTTTC

>GQ301552.1-Papio_hamadryas_ursinus-South_Africa Trichuris sp. ex Papio ursinus NL-2009 isolate DGII internal transcribed spacer 1, partial sequence; 5.8S ribosomal RNA gene, complete sequence; and internal transcribed spacer 2, partial sequence

TCGGTCGAGCGGTGCCCGCCGCCGCAGCAAGGCGAACGGTTTCGTCGTCGTCGTCGTCGACTGCTGCTGCCGCTGCTGCT

GCTGCCGTGCAACTACGACGAACACTTGCTGCTACGAGCGCGTGCGGTCACCGCTCCCTGCTCAACTGCTGTACGCCGAC

GGCGCGGCCGGTAGCCAAGTCGGGTAGTCTTCGCCCTACGCTGACTTGGCCGCTCTCCGCTGCTCCGCCTACCAGTTGCC

ATCGCGGCTCGCTGGCAACGGAGCTGCGGCGTCACGCTCGCTCGCGCGCGGATGCAGGTTTGTCCGGTGCTCGCCACCAA

CCTCAACCTGTAGCCTGAGCGGCGCATTCGCTATTATGCTTAACGACCCTCAGACAGGCGTAGCGTCGACATCTTTGATC

GACGCTGCAATGTGCGTTCAAAGTATTCAAGTTCGGTGTGTCTGCAATTCACGTCAATTCTCGAGTGTCACGTCGTTCTT

CAACGATCTACGAGCCAAGTGATCCACCGTTCGGAATGTACTTTCCTGCGCGTACAGCAGCAGTTCGAATGCTACTAGCG

CTCATTGCGCCGACAGCAGTGTCTGCGTGTCGAAGGCACATCGGCGTGCGTAGTAGGACGACGCCTTGTGCACTGGTGAA

GCTGCTTTTTC

>GQ301553.1-Papio_hamadryas_ursinus-South_Africa Trichuris sp. ex Papio ursinus NL-2009 isolate DGI internal transcribed spacer 1, partial sequence; 5.8S ribosomal RNA gene, complete sequence; and internal transcribed spacer 2, partial sequence

TCGGTCGAGCGGTGCCCGCCGCCGCAGCAAGGCGAACGGTTTCGTCGTCGTCGTCGTCGACTGCTGCTGCCGCTGCTGCT

GCTGCCGTGCAACTACGACGAACACTTGCTGCTACGAGCGCGTGCGGTCACCGCTCCCTGCTCAACTGCTGTACGCCGAC

GGCGCGGCCGGTAGCCAAGTCGGGTAGTCTTCGCCCTACGCTGACTTGGCCGCTCTCCGCTGCTCCGCCTACCAGTTGCC

ATCGCGGCTCGCTGGCAACGGAGCTGCGGCGTCACGCTCGCTCGCGCGCGGATGCAGGTTTGTCCGGTGCTCGCCACCAA

CCTCAACCTGTAGCCTGAGCGGCGCATTCGCTATTATGCTTAACGACCCTCAGACAGGCGTAGCGTCGACATCTTTGATC

GACGCTGCAATGTGCGTTCAAAGTATTCAAGTTCGGTGTGTCTGCAATTCACGTCAATTCTCGAGTGTCACGTCGTTCTT

CAACGATCTACGAGCCAAGTGATCCACCGTTCGGAATGTACTTTCCTGCGCGTACAGCAGCAGTTCGAATGCTACTAGCG

CTCATTGCGCCGACAGCAGTGTCTGCGTGTCGAAGGCACATCGGCGTGCGTAGTAGGACGACGCCTTGTGCACTGGTGAA

GCTGCTTTTTC

>KT344825.1-Rhinopithecus_roxellana-China Trichuris sp. SC1 internal transcribed spacer 1, partial sequence; 5.8S ribosomal RNA gene, complete sequence; and internal transcribed spacer 2, partial sequence

TCGGTCGAGCGGTGCCCGCCGCCGCCGCAGCAAGGCGAACGGTTTCGTCGTCGTCGTCGACTGCTGCTGCCGCTGCTGCC

GTGCAACGACGACGAACACTTGCTGCTACGAGCGCGTGCGGTCACCGCTCCCTGCTCAACTGCTGTACGCCGACGGCGCG

GCCGGTAGCCAAGTCGGGTAGTCTTCGCCCTACGCTGACTTGGCCGCTCTCCGCTGCTCCGCCTACCAGTTGCCATCGCG

GCTCGCTGGCAACGGAGCTGCGGCGTCACGCTCGCTCGCGCGCGGATGCAGGTTTGTCCGGTGCTCGCCACCAACCTCAA

CCTGTAGCCTGAGCGGCGCATTCGCTATTATGCTTAACGACCCTCAGACAGGCGTAGCGTCGACATCTTTGATCGACGCT

GCAATGTGCGTTCAAAGTATTCAAGTTCGGTGTGTCTGCAATTCACGTCAATTCTCGAGTGTCACGTCGTTCTTCAACGA

TCTACGAGCCAAGTGATCCACCGTTCGGAATGTACTTTCCTGCGCGTACAGCAGCAGTTCGAATGCTACTAGCGCTCATT

GCGCCGACAGCAGTGTCTGCGTGTCGAAGGCACATCGGCGTGCGTAGTAGGACGACGCCTTGTGCACTGGTGAAGCTGCC

TTTTC

>MN447328.1-Papio_anubis-China Trichuris sp. Pa1 GY-2019 small subunit ribosomal RNA gene, partial sequence; internal transcribed spacer 1, 5.8S ribosomal RNA gene, and internal transcribed spacer 2, complete sequence; and large subunit ribosomal RNA gene, partial sequence

TCGGTCGAGCGGTGCCCGCCGCCGCCGCAGCAAGGCGAACGGTTTCGTCGTCGTCGTCGTCGACTGCTGCTGCCGCTGCT

GCCGTGCAACGACGACGAACACTTGCTGCTACGAGCGCGTGCGATCACCGCTCCCTGCTCAACTGCTGTACGCCGACGGC

GCGGCCGGTAGCCAAGTCGGGTAGTCTTCGCCCTACGCTGACTTGGCCGCTCTCCGCTGCTCCGCCTACCAGTTGCCATC

GCGGCTCGCTGGCAACGGAGCTGCGGCGTCACGCTCGCTCGCGCGCGGATGCAGGTTTGTCCGGTGCTCGCCACCAACCT

CAACCTGTAGCCTGAGCGGCGCATTCGCTATTATGCTTAACGACCCTCAGACAGGCGTAGCGTCGACATCTTTGATCGAC

GCTGCAATGTGCGTTCAAAGTATTCAAGTTCGGTGTGTCTGCAATTCACGTCAATTCTCGAGTGTCACGTCGTTCTTCAA

CGATCTACGAGCCAAGTGATCCACCGTTCGGAATGTACTTTCCTGCGCGTACAGCAGCAGTTCGAATGCTACTAGCGCTC

ATTGCGCCGACAGCAGTGTCTGCGTGTCGAAGGCACATCGGCGTGCGTAGTAGGACGACGCCTTGTGCACTGGTGAAGCT

GCTTTTTC

>AM992998.1-Unknown-China Trichuris trichiura ITS1 (partial), 5.8S rRNA gene and ITS2 (partial), isolate Ttr8c

TCGGTCGAGCGGTGCCCGCCGCCGCAGCAAGGCGAACGGTTTCGTCGTCGTCGTCGACTGCTGCTGCTGCCGTGCAACTA

CGACGAACACTTGCTGCTACGAGCGCGTGCGGTCACCGCTCCCTGCTCAACTGCTGTACGCCGACGGCGCGGCCGGTAGC

CAAGTCGGGTAGTCTTCGCCCTACGCTGACTTGGCCGCTCTCCGCTGCTCCGCCTACCAGTTGCCATCGCGGCTCGCTGG

CAACGGAGCTGCGGCGTCACGCTCGCTCGCGCGCGGATGCAGGTTTGTCCGGTGCTCGCCACCAACCTCAACCTGTAGCC

TGAGCGGCGCATTCGCTATTATGCTTAACGACCCTCAGACAGGCGTAGCGTCGACATCTTTGATCGACGCTGCAATGTGC

GTTCAAAGTATTCAAGTTCGGTGTGTCTGCAATTCACGTCAATTCTCGAGTGTCACGTCGTTCTTCAACGATCTACGAGC

CAAGTGATCCACCGTTCGGAATGTACTTTCCTGCGCGTACAGCAGCAGTTCGAATGCTACTAGCGCTCATTGCGCCGACA

GCAGTGTCTGCGTGTCGAAGGCACATCGGCGTGCGTAGTAGGACGACGCCTTGTGCACTGGTGAAGCTGCTTTTTC

>AM992987.1-Unknown-China Trichuris trichiura ITS1 (partial), 5.8S rRNA gene and ITS2 (partial), isolate Ttr5a

TCGGTCGAGCGGTGCCCGCCGCCGCCGCAGCAAGGCGAACGGTTTCGTCGTCGTCGACTGCTGCTGCCGTGCAACTACGA

CGAACACTTGCTGCTACGAGCGCGTGCGATCACCGCTCCCTGCTCAACTGCTGTACGCCGACGGCGCGGCCGGTAGCCAA

GTCGGGTAGTCTTCGCCCTACGCTGACTTGGCCGCTCTCCGCTGCTCCGCCTACCAGTTGCCATCGCGGCTCGCTGGCAA

CGGAGCTGCGGCGTCACGCTCGCTCGCGCGCGGATGCAGGTTTGTCCGGTGCTCGCCACCAACCTCAACCTGTAGCCTGA

GCGGCGCATTCGCTATTATGCTTAACGACCCTCAGACAGGCGTAGCGTCGACATCTTTGATCGACGCTGCAATGTGCGTT

CAAAGTATTCAAGTTCGGTGTGTCTGCAATTCACGTCAATTCTCGAGTGTCACGTCGTTCTTCAACGATCTACGAGCCAA

GTGATCCACCGTTCGGAATGTACTTTCCTGCGCGTACAGCAGCAGTTCGAATGCTACTAGCGCTCATTGCGCCGACAGCA

GTGTCTGCGTGTCGAAGGCACATCGGCGTGCGTAGTAGGACGACGCCTTGTGCACTGGTGAAGCTGCCTTTTC

>AM992996.1-Unknown-China Trichuris trichiura ITS1 (partial), 5.8S rRNA gene and ITS2 (partial), isolate Ttr8a

TCGGTCGAGCGGTGCCCGCCGCCGCAGCAAGGCGAACGGTTTCGTCGTCGTCGTCGACTGCTGCTGCTGCCGTGCAACTA

CGACGAACACTTGCTGCTACGAGCGCGTGCGGTCACCGCTCCCTGCTCAACTGCTGTACGCCGACGGCGCGGCCGGTAGC

CAAGTCGGGTAGTCTTCGCCCTACGCTGACTTGGCCGCTCTCCGCTGCTCCGCCTACCAGTTGCCATCGCGGCTCGCTGG

CAACGGAGCTGCGGCGTCACGCTCGCTCGCGCGCGGATGCAGGTTTGTCCGGTGCTCGCCACCAACCTCAACCTGTAGCC

TGAGCGGCGCATTCGCTATTATGCTTAACTTTGATCGACGCTGCAATGTGCGTTCAAAGTATTCAAGTTCGGTGTGTCTG

CAATTCACGTCAATTCTCGAGTGTCACGTCGTTCTTCAACGATCTACGAGCCAAGTGATCCACCGTTCGGAATGTACTTT

CCTGCGCGTACAGCAGCAGTTCGAATGCTACTAGCGCTCATTGCGCCGACAGCAGTGTCTGCGTGTCGAAGGCACATCGG

CGTGCGTAGTAGGACGACGCCTTGTGCACTGGTGAAGCTGCTTTTTC

>KT344832.1-Rhinopithecus_bieti-China Trichuris sp. KM1 internal transcribed spacer 1, partial sequence; 5.8S ribosomal RNA gene, complete sequence; and internal transcribed spacer 2, partial sequence

TCGGTCGAGCGGTGCCGGCCGCAGCAAGGCGAACGGTGTCGTCGTCGTCGGCTGCTGCTGCCGTCGAACGACGACGAACA

CTTGCTGCGAGCGCGTGCGGTCACCGCTCGCTGCTCAACTGTCCAGTCGCGCTGCTGTACGCCGACGGCGCGGCCGGTAG

CCAAGTCGGGTAGTCTTCGCACTACGCTGACTTGGCCGCTCTCCGCTGCTCCGCCTACCAGTTGCCATCGCCGCTCGCTG

GCAACGGAGCTGCGGCGTCACGCTCGCTCGCCGGATGCAGGTTTGTCCAGTGCTCGCCACCAACCTCAACCTGTAGCCTG

AGCGGCGCATTCGCTATTGCTTAACGACCCTCAGACAGGCGTAGCGTCGACATCTTTGATCGACGCTGCAATGTGCGTTC

AAAGTATTCAAGTTCGGTGTGTCTGCAATTCACGTCAATTCTCGAGTGTCACGTCGTTCTTCAACGATCTACGAGCCAAG

TGATCCACCGTTCGGAATGTACTTTCCTGCGCGTACAGCAGCAGTTCGAATGCTACTAGCGCTCATTGCGCCGACAGCAG

TGTCTGCGTGTCGAAGGCACATCGGCGTGCGTAGTAGGACGACGCCTTGTGCACTGGTGAAGCTGCCTTTTC

>MH390362.1-Rhinopithecus_bieti-China Trichuris sp. Rb2 internal transcribed spacer 1, partial sequence; 5.8S ribosomal RNA gene, complete sequence; and internal transcribed spacer 2, partial sequence

TCGGTCGAGCGGTGCCGGCCGCAGCAAGGCGAACGGTGTCGTCGTCGTCGGCTGCTGCTGCCGTCGAACGACGACGAACA

CTTGCTGCGAGCGCGTGCGGTCACCGCTCGCTGCTCAACTGTCCAGTCGCGCTGCTGTACGCCGACGGCGCGGCCGGTAG

CCAAGTCGGGTAGTCTTCGCACTACGCTGACTTGGCCGCTCTCCGCTGCTCCGCCTACCAGTTGCCATCGCCGCTCGCTG

GCAACGGAGCTGCGGCGTCACGCTCGCTCGCCGGATGCAGGTTTGTCCAGTGCTCGCCACCAACCTCAACCTGTAGCCTG

AGCGGCGCATTCGCTATTGCTTAACGACCCTCAGACAGGCGTAGCGTCGACATCTTTGATCGACGCTGCAATGTGCGTTC

AAAGTATTCAAGTTCGGTGTGTCTGCAATTCACGTCAATTCTCGAGTGTCACGTCGTTCTTCAACGATCTACGAGCCAAG

TGATCCACCGTTCGGAATGTACTTTCCTGCGCGTACAGCAGCAGTTCGAATGCTACTAGCGCTCATTGCGCCGACAGCAG

TGTCTGCGTGTCGAAGGCACATCGGCGTGCGTAGTAGGACGACGCCTTGTGCACTGGTGAAGCTGCCTTTTC

>KP336469.1-Macaca_fuscata-Italy Trichuris sp. MCTV isolate MCTV1 internal transcribed spacer 1, partial sequence; 5.8S ribosomal RNA gene and internal transcribed spacer 2, complete sequence; and 28S ribosomal RNA gene, partial sequence

TCGGTCGAGCGGTGCCCGCCGCAGCAAGGCGGGCGGTATCGTCGTCGTCGACTGCTGCTGCCGTGCAACGACGTCGAACA

CTTGCTGCGAGCGCGTGCGGTCACCGCTCGGTGCTCAACTGTCGATTCGCGCTGCTGTACGCCGACGGCGCGGCCAGTAG

CCGAGTCGGGTAGTCTTCGCACTACGCTGACTTGGCCGCTCTCCGCTGCTCCGGCTAGCAGTTGCCATCGCGGCTCGCTG

GCAACGGAGCTGCGGCGTCACGCTCGCTCGCGGGTGCAGGTTTGTCCAGTGCTCGCCACCAACCTCAACCTGTAGCCTGA

GCGGCGCATTCGCTATTGCTTAACGACCCTCAGACAGGCGTAGCGTCGACATCTTTGATCGACGCTGCAATGTGCGTTCA

AAGTATTCAAGTTCGGTGTGTCTGCAATTCACGTCAATTCTCGAGTGTCACGTCGTTCTTCAACGATCTACGAGCCAAGT

GATCCACCGTTCGGAATGTACTTTCCTGCGCGTACAGCAGTTCGAATGGTACTAGCGCTCATTGCGCCGACAGCAATGTC

TGCGTGTCGAAGGCACGTCGGCATGTGTAGTACAACGCCTTGTGCACTGGTGAACGGCTATTTTC

>KP336468.1-Macaca_fuscata-Italy Trichuris sp. MCTV isolate MCTV10 internal transcribed spacer 1, partial sequence; 5.8S ribosomal RNA gene and internal transcribed spacer 2, complete sequence; and 28S ribosomal RNA gene, partial sequence

TCGGTCGAGCGGTGCCCGCCGCAGCAAGGCGGGCGGTATCGTCGTCGTCGACTGCTGCTGCCGTGCAACGACGTCGAACA

CTTGCTGCGAGCGCGTGCGGTCACCGCTCGGTGCTCAACTGTCGATTCGCGCTGCTGTACGCCGACGGCGCGGCCAGTAG

CCGAGTCGGGTAGTCTTCGCACTACGCTGACTTGGCCGCTCTCCGCTGCTCCGGCTAGCAGTTGCCATCGCGGCTCGCTG

GCAACGGAGCTGCGGCGTCACGCTCGCTCGCGGGTGCAGGTTTGTCCAGTGCTCGCCACCAACCTCAACCTGTAGCCTGA

GCGGCGCATTCGCTATTGCTTAACGACCCTCAGACAGGCGTAGCGTCGACATCTTTGATCGACGCTGCAATGTGCGTTCA

AAGTATTCAAGTTCGGTGTGTCTGCAATTCACGTCAATTCTCGAGTGTCACGTCGTTCTTCAACGATCTACGAGCCAAGT

GATCCACCGTTCGGAATGTACTTTCCTGCGCGTACAGCAGTTCGAATGGTACTAGCGCTCATTGCGCCGACAGCAATGTC

TGCGTGTCGAAGGCACGTCGGCATGTGTAGTACAACGCCTTGTGCACTGGTGAACGGCTATTTTC

>KP336466.1-Macaca_fuscata-Italy Trichuris sp. MCTV isolate MCTV3 internal transcribed spacer 1, partial sequence; 5.8S ribosomal RNA gene and internal transcribed spacer 2, complete sequence; and 28S ribosomal RNA gene, partial sequence

TCGGTCGAGCGGTGCCCGCCGCAGCAAGGCGGGCGGTATCGTCGTCGTCGACTGCTGCTGCCGTGCAACGACGTCGAACA

CTTGCTGCGAGCGCGTGCGGTCACCGCTCGGTGCTCAACTGTCGATTCGCGCTGCTGTACGCCGACGGCGCGGCCAGTAG

CCGAGTCGGGTAGTCTTCGCACTACGCTGACTTGGCCGCTCTCCGCTGCTCCGGCTAGCAGTTGCCATCGCGGCTCGCTG

GCAACGGAGCTGCGGCGTCACGCTCGCTCGCGGGTGCAGGTTTGTCCAGTGCTCGCCACCAACCTCAACCTGTAGCCTGA

GCGGCGCATTCGCTATTGCTTAACGACCCTCAGACAGGCGTAGCGTCGACATCTTTGATCGACGCTGCAATGTGCGTTCA

AAGTATTCAAGTTCGGTGTGTCTGCAATTCACGTCAATTCTCGAGTGTCACGTCGTTCTTCAACGATCTACGAGCCAAGT

GATCCACCGTTCGGAATGTACTTTCCTGCGCGTACAGCAGTTCGAATGGTACTAGCGCTCATTGCGCCGACAGCAATGTC

TGCGTGTCGAAGGCACGTCGGCATGTGTAGTACAACGCCTTGTGCACTGGTGAACGGCTATTTTC

>KP336470.1-Macaca_fuscata-Italy Trichuris sp. MCTV isolate MCTV18 internal transcribed spacer 1, partial sequence; 5.8S ribosomal RNA gene and internal transcribed spacer 2, complete sequence; and 28S ribosomal RNA gene, partial sequence

TCGGTCGAGCGGTGCCCGCCGCAGCAAGGCGGGCGGTATCGTCGTCGTCGACTGCTGCTGCCGTGCAACGACGTCGAACA

CTTGCTGCGAGCGCGTGCGGTCACCGCTCGGTGCTCAACTGTCGATTCGCGCTGCTGTACGCCGACGGCGCGGCCAGTAG

CCGAGTCGGGTAGTCTTCGCACTACGCTGACTTGGCCGCTCTCCGCTGCTCCGGCTAGCAGTTGCCATCGCGGCTCGCTG

GCAACGGAGCTGCGGCGTCACGCTCGCTCGCGGGTGCAGGTTTGTCCAGTGCTCGCCACCAACCTCAACCTGTAGCCTGA

GCGGCGCATTCGCTATTGCTTAACGACCCTCAGACAGGCGTAGCGTCGACATCTTTGATCGACGCTGCAATGTGCGTTCA

AAGTATTCAAGTTCGGTGTGTCTGCAATTCACGTCAATTCTCGAGTGTCACGTCGTTCTTCAACGATCTACGAGCCAAGT

GATCCACCGTTCGGAATGTACTTTCCTGCGCGTACAGCAGTTCGAATGGTACTAGCGCTCATTGCGCCGACAGCAATGTC

TGCGTGTCGAAGGCACGTCGGCATGTGTAGTACAACGCCTTGTGCACTGGTGAACGGCTATTTTC

>KP336464.1-Macaca_fuscata-Italy Trichuris sp. MCTV isolate MCTV7 internal transcribed spacer 1, partial sequence; 5.8S ribosomal RNA gene and internal transcribed spacer 2, complete sequence; and 28S ribosomal RNA gene, partial sequence

TCGGTCGAGCGGTGCCCGCCGCAGCAAGGCGGGCGGTATCGTCGTCGTCGACTGCTGCTGCCGTGCAACGACGTCGAACA

CTTGCTGCGAGCGCGTGCGGTCACCGCTCGGTGCTCAACTGTCGATTCGCGCTGCTGTACGCCGACGGCGCGGCCAGTAG

CCGAGTCGGGTAGTCTTCGCACTACGCTGACTTGGCCGCTCTCCGCTGCTCCGGCTAGCAGTTGCCATCGCGGCTCGCTG

GCAACGGAGCTGCGGCGTCACGCTCGCTCGCGGATGCAGGTTTGTCCAGTGCTCGCCACCAACCTCAACCTGTAGCCTGA

GCGGCGCATTCGCTATTGCTTAACGACCCTCAGACAGGCGTAGCGTCGACATCTTTGATCGACGCTGCAATGTGCGTTCA

AAGTATTCAAGTTCGGTGTGTCTGCAATTCACGTCAATTCTCGAGTGTCACGTCGTTCTTCAACGATCTACGAGCCAAGT

GATCCACCGTTCGGAATGTACTTTCCTGCGCGTACAGCAGTTCGAATGGTACTAGCGCTCATTGCGCCGACAGCAATGTC

TGCGTGTCGAAGGCACGTCGGCATGTGTAGTACAACGCCTTGTGCACTGGTGAACGGCTATTTTC

>KP336471.1-Macaca_fuscata-Italy Trichuris sp. MCTV isolate MCTV12 internal transcribed spacer 1, partial sequence; 5.8S ribosomal RNA gene and internal transcribed spacer 2, complete sequence; and 28S ribosomal RNA gene, partial sequence

TCGGTCGAGCGGTGCCCGCCGCAGCAAGGCGGGCGGTATCGTCGTCGTCGACTGCTGCTGCCGTGCAACGACGTCGAACA

CTTGCTGCGAGCGCGTGCGGTCACCGCTCGGTGCTCAACTGTCGATTTGCGCTGCTGTACGCCGACGGCGCGGCCAGTAG

CCGAGTCGGGTAGTCTTCGCACTACGCTGACTTGGCCGCTCTCCGCTGCTCCGGCTAGCAGTTGCCATCGCGGCTCGCTG

GCAACGGAGCTGCGGCGTCACGCTCGCTCGCGGGTGCAGGTTTGTCCAGTGCTCGCCACCAACCTCAACCTGTAGCCTGA

GCGGCGCATTCGCTATTGCTTAACGACCCTCAGACAGGCGTAGCGTCGACATCTTTGATCGACGCTGCAATGTGCGTTCA

AAGTATTCAAGTTCGGTGTGTCTGCAATTCACGTCAATTCTCGAGTGTCACGTCGTTCTTCAACGATCTACGAGCCAAGT

GATCCACCGTTCGGAATGTACTTTCCTGCGCGTACAGCAGTTCGAATGGTACTAGCGCTCATTGCGCCGACAGCAATGTC

TGCGTGTCGAAGGCACGTCGGCATGTGTAGTACAACGCCTTGTGCACTGGTGAACGGCTATTTTC

>KP336459.1-Macaca_fuscata-Italy Trichuris sp. MCTV isolate MCTV6 internal transcribed spacer 1, partial sequence; 5.8S ribosomal RNA gene and internal transcribed spacer 2, complete sequence; and 28S ribosomal RNA gene, partial sequence

TCGGTCGAGCGGTGCCCGCCGCAGCAAGGCGGGCGGTATCGTCGTCGTCGACTGCTGCTGCCGTGCAACGACGTCGAACA

CTTGCTGCGAGCGCGTGCGGTCACCGCTCGGTGCTCAACTGTCGATTTGCGCTGCTGTACGCCGACGGCGCGGCCAGTAG

CCGAGTCGGGTAGTCTTCGCACTACGCTGACTTGGCCGCTCTCCGCTGCTCCGGCTAGCAGTTGCCATCGCGGCTCGCTG

GCAACGGAGCTGCGGCGTCACGCTCGCTCGCGGGTGCAGGTTTGTCCAGTGCTCGCCACCAACCTCAACCTGTAGCCTGA

GCGGCGCATTCGCTATTGCTTAACGACCCTCAGACAGGCGTAGCGTCGACATCTTTGATCGACGCTGCAATGTGCGTTCA

AAGTATTCAAGTTCGGTGTGTCTGCAATTCACGTCAATTCTCGAGTGTCACGTCGTTCTTCAACGATCTACGAGCCAAGT

GATCCACCGTTCGGAATGTACTTTCCTGCGCGTACAGCAGTTCGAATGGTACTAGCGCTCATTGCGCCGACAGCAATGTC

TGCGTGTCGAAGGCACGTCGGCATGTGTAGTACAACGCCTTGTGCACTGGTGAACGGCTATTTTC

>KP336463.1-Macaca_fuscata-Italy Trichuris sp. MCTV isolate MCTV16 internal transcribed spacer 1, partial sequence; 5.8S ribosomal RNA gene and internal transcribed spacer 2, complete sequence; and 28S ribosomal RNA gene, partial sequence

TCGGTCGAGCGGTGCCCGCCGCAGCAAGGCGGGCGGTATCGTCGTCGTCGACTGCTGCTGCCGTGCAACGACGTCGAACA

CTTGCTGCGAGCGCGTGCGGTCACCGCTCGGTGCTCAACTGTCGATTTGCGCTGCTGTACGCCGACGGCGCGGCCAGTAG

CCGAGTCGGGTAGTCTTCGCACTACGCTGACTTGGCCGCTCTCCGCTGCTCCGGCTAGCAGTTGCCATCGCGGCTCGCTG

GCAACGGAGCTGCGGCGTCACGCTCGCTCGCGGATGCAGGTTTGTCCAGTGCTCGCCACCAACCTCAACCTGTAGCCTGA

GCGGCGCATTCGCTATTGCTTAACGACCCTCAGACAGGCGTAGCGTCGACATCTTTGATCGACGCTGCAATGTGCGTTCA

AAGTATTCAAGTTCGGTGTGTCTGCAATTCACGTCAATTCTCGAGTGTCACGTCGTTCTTCAACGATCTACGAGCCAAGT

GATCCACCGTTCGGAATGTACTTTCCTGCGCGTACAGCAGTTCGAATGGTACTAGCGCTCATTGCGCCGACAGCAATGTC

TGCGTGTCGAAGGCACGTCGGCATGTGTAGTACAACGCCTTGTGCACTGGTGAACGGCTATTTTC

>KP336462.1-Macaca_fuscata-Italy Trichuris sp. MCTV isolate MCTV11 internal transcribed spacer 1, partial sequence; 5.8S ribosomal RNA gene and internal transcribed spacer 2, complete sequence; and 28S ribosomal RNA gene, partial sequence

TCGGTCGAGCGGTGCCCGCCGCAGCAAGGCGGGCGGTATCGTCGTCGTCGACTGCTGCTGCCGTGCAACGACGTCGAACA

CTTGCTGCGAGCGCGTGCGGTCACCGCTCGGTGCTCAACTGTCGATTTGCGCTGCTGTACGCCGACGGCGCGGCCAGTAG

CCGAGTCGGGTAGTCTTCGCACTACGCTGACTTGGCCGCTCTCCGCTGCTCCGGCTAGCAGTTGCCATCGCGGCTCGCTG

GCAACGGAGCTGCGGCGTCACGCTCGCTCGCGGATGCAGGTTTGTCCAGTGCTCGCCACCAACCTCAACCTGTAGCCTGA

GCGGCGCATTCGCTATTGCTTAACGACCCTCAGACAGGCGTAGCGTCGACATCTTTGATCGACGCTGCAATGTGCGTTCA

AAGTATTCAAGTTCGGTGTGTCTGCAATTCACGTCAATTCTCGAGTGTCACGTCGTTCTTCAACGATCTACGAGCCAAGT

GATCCACCGTTCGGAATGTACTTTCCTGCGCGTACAGCAGTTCGAATGGTACTAGCGCTCATTGCGCCGACAGCAATGTC

TGCGTGTCGAAGGCACGTCGGCATGTGTAGTACAACGCCTTGTGCACTGGTGAACGGCTATTTTC

>KP336473.1-Macaca_fuscata-Italy Trichuris sp. MCTV isolate MCTV9 internal transcribed spacer 1, partial sequence; 5.8S ribosomal RNA gene and internal transcribed spacer 2, complete sequence; and 28S ribosomal RNA gene, partial sequence

TCGGTCGAGCGGTGCCCGCCGCAGCAAGGCGGGCGGTATCGTCGTCGTCGACTGCTGCTGCCGTGCAACGACGTCGAACA

CTTGCTGCGAGCGCGTGCGGTCACCGCTCGGTGCTCAACTGTCGATTCGCGCTGCTGTACGCCGACGGCGCGGCCAGTAG

CCGAGTCGGGTAGTCTTCGCACTACGCTGACTTGGCCGCTCTCCGCTGCTCCGGCTAGCAGTTGCCATCGCGGCTCGCTG

GCAACGGAGCTGCGGCGTCACGCTCGCTCACGGGTGCAGGTTTGTCCAGTGCTCACCACCAACCTCAACCTGTAGCCTGA

GCGGCGCATTCGCTATTGCTTAACGACCCTCAGACAGGCGTAGCGTCGACATCTTTGATCGACGCTGCAATGTGCGTTCA

AAGTATTCAAGTTCGGTGTGTCTGCAATTCACGTCAATTCTCGAGTGTCACGTCGTTCTTCAACGATCTACGAGCCAAGT

GATCCACCGTTCGGAATGTACTTTCCTGCGCGTACAGCAGTTCGAATGGTACTAGCGCTCATTGCGCCGACAGCAATGTC

TGCGTGTCGAAGGCACGTCGGCATGTGTAGTACAACGCCTTGTGCACTGGTGAACGGCTATTTTC

>KP336476.1-Macaca_fuscata-Italy Trichuris sp. MCTV isolate MCTV5 internal transcribed spacer 1, partial sequence; 5.8S ribosomal RNA gene and internal transcribed spacer 2, complete sequence; and 28S ribosomal RNA gene, partial sequence

TCGGTCGAGCGGTGCCCGCCGCAGCAAGGCGGGCGGTATCGTCGTCGTCGACTGCTGCTGCCGTGCAACGACGTCGAACA

CTTGCTGCGAGCGCGTGCGGTCACCGCTCGGTGCTCAACTGTCGATTCGCGCTGCTGTACGCCGACGGCGCGGCCAGTAG

CCGAGTCGGGTAGTCTTCGCACTACGCTGACTTGGCCGCTCTCCGCTGCTCCGGCTAGCAGTTGCCATCGCGGCTCGCTG

GCAACGGAGCTGCGGCGTCACGCTCGCTCACGGGTGCAGGTTTGTCCAGTGCTCACCACCAACCTCAACCTGTAGCCTGA

GCGGCGCATTCGTTATTGCTTAACGACCCTCAGACAGGCGTAGCGTCGACATCTTTGATCGACGCTGCAATGTGCGTTCA

AAGTATTCAAGTTCGGTGTGTCTGCAATTCACGTCAATTCTCGAGTGTCACGTCGTTCTTCAACGATCTACGAGCCAAGT

GATCCACCGTTCGGAATGTACTTTCCTGCGCGTACAGCAGTTCGAATGGTACTAGCGCTCATTGCGCCGACAGCAATGTC

TGCGTGTCGAAGGCACGTCGGCATGTGTAGTACAACGCCTTGTGCACTGGTGAACGGCTATTTTC

>KP336475.1-Macaca_fuscata-Italy Trichuris sp. MCTV isolate MCTV17 internal transcribed spacer 1, partial sequence; 5.8S ribosomal RNA gene and internal transcribed spacer 2, complete sequence; and 28S ribosomal RNA gene, partial sequence

TCGGTCGAGCGGTGCCCGCCGCAGCAAGGCGGGCGGTATCGTCGTCGTCGACTGCTGCTGCCGTGCAACGACGTCGAACA

CTTGCTGCGAGCGCGTGCGGTCACCGCTCGGTGCTCAACTGTCGATTCGCGCTGCTGTACGCCGACGGCGCGGCCAGTAG

CCGAGTCGGGTAGTCTTCGCACTACGCTGACTTGGCCGCTCTCCGCTGCTCCGGCTAGCAGTTGCCATCGCGGCTCGCTG

GCAACGGAGCTGCGGCGTCACGCTCGCTCGCGGGTGCAGGTTTGTCCAGTGCTCACCACCAACCTCAACCTGTAGCCTGA

GCGGCGCATTCGCTATTGCTTAACGACCCTCAGACAGGCGTAGCGTCGACATCTTTGATCGACGCTGCAATGTGCGTTCA

AAGTATTCAAGTTCGGTGTGTCTGCAATTCACGTCAATTCTCGAGTGTCACGTCGTTCTTCAACGATCTACGAGCCAAGT

GATCCACCGTTCGGAATGTACTTTCCTGCGCGTACAGCAGTTTGAATGGTACTAGCGCTCATTGCGCCGACAGCAATGTC

TGCGTGTCGAAGGCACGTCGGCATGTGTAGTACAACGCCTTGTGCACTGGTGAACGGCTATTTTC

>KP336461.1-Macaca_fuscata-Italy Trichuris sp. MCTV isolate MCTV13 internal transcribed spacer 1, partial sequence; 5.8S ribosomal RNA gene and internal transcribed spacer 2, complete sequence; and 28S ribosomal RNA gene, partial sequence

TCGGTCGAGCGGTGCCCGCCGCAGCAAGGCGGGCGGTATCGTCGTCGTCGACTGCTGCTGCCGTGCAACGACGTCGAACA

CTTGCTGCGAGCGCGTGCGGTCACCGCTCGGTGCTCAACTGTCGATTTGCGCTGCTGTACGCCGACGGCGCGGCCAGTAG

CCGAGTCGGGTAGTCTTCGCACTACGCTGACTTGGCCGCTCTCCGCTGCTCCGGCTAGCAGTTGCCATCGCGGCTCGCTG

GCAACGGAGCTGCGGCGTCACGCTCGCTCGCGGATGCAGGTTTGTCCAGTGCTCGCCACCAACCTCAACCTGTAGCCTGA

GCGGCGCATTCGCTATTGCTTAACGACCCTCAGACAGGCGTAGCGTCGACATCTTTGATCGACGCTGCAATGTGCGTTCA

AAGTATTCAAGTTCGGTGTGTCTGCAATTCACGTCAATTCTCGAGTGTCACGTCGTTCTTCAACGATCTACGAGCCAAGT

GATCCACCGTTCGGAATGTACTTTCCTGCGCGTACAGCAGTTCGAATGGTACTAGCGCTCATTGCGCCGACAGCAATGTC

TGCGTGTCGAACGCACGTCGGCATGTGTAGTACAACGCCTTGTGCACTGATGAACGGCTATTTTC

>KP336460.1-Macaca_fuscata-Italy Trichuris sp. MCTV isolate MCTV2 internal transcribed spacer 1, partial sequence; 5.8S ribosomal RNA gene and internal transcribed spacer 2, complete sequence; and 28S ribosomal RNA gene, partial sequence

TCGGTCGAGCGGTGCCCGCCGCAGCAAGGCGGGCGGTATCGTCGTCGTCGACTGCTGCTGCCGTGCAACGACGTCGAACA

CTTGCTGCGAGCGCGTGCGGTCACCGCTCGGTGCTCAACTGTCGATTTGCGCTGCTGTACGCCGACGGCGCGGCCAGTAG

CCGAGTCGGGTAGTCTTCGCACTACGCTGACTTGGCCGCTCTCCGCTGCTCCGGCTAGCAGTTGCCATCGCGGCTCGCTG

GCAACGGAGCTGCGGCGTCACGCTCGCTCGCGGATGCAGGTTTGTCCAGTGCTCGCCACCAACCTCAACCTGTAGCCTGA

GCGGCGCATTCGCTATTGCTTAACGACCCTCAGACAGGCGTAGCGTCGACATCTTTGATCGACGCTGCAATGTGCGTTCA

AAGTATTCAAGTTCGGTGTGTCTGCAATTCACGTCAATTCTCGAGTGTCACGTCGTTCTTCAACGATCTACGAGCCAAGT

GATCCACCGTTCGGAATGTACTTTCCTGCGCGTACAGCAGTTCGAATGGTACTAGCGCTCATTGCGCCGACAGCAATGTC

TGCGTGTCGAAAGCACGTCGGCATGTGTAGTACAACGCCTTGTGCACTGATGAACGGCTATTTTC

>KP336465.1-Macaca_fuscata-Italy Trichuris sp. MCTV isolate MCTV8 internal transcribed spacer 1, partial sequence; 5.8S ribosomal RNA gene and internal transcribed spacer 2, complete sequence; and 28S ribosomal RNA gene, partial sequence

TCGGTCGAGCGGTGCCCGCCGCAGCAAGGCGGGCGGTATCGTCGTCGTCGACTGCTGCTGCCGTGCAACGACGTCGAACA

CTTGCTGCGAGCGCGTGCGGTCACCGCTCGGTGCTCAACTGTCGATTCGCGCTGCTGTACGCCGACGGCGCGGCCAGTAG

CCGAGTCGGGTAGTCTTCGCACTACGCTGACTTGGCCGCTCTCCGCTGCTCCGGCTAGCAGTTGCCATCGCGGCTCGCTG

GCAACGGAGCTGCGGCGTCACGCTCGCTCGCGGGTGCAGGTTTGTCCAGTGCTCGCCACCAACCTCAACCTGTAGCCTGA

GCGGCGCATTCGCTATTGCTTAACGACCCTCAGACAGGCGTAGCGTCGACATCTTTGATCGACGCTGCAATGTGCGTTCA

AAGTATTCAAGTTCGGTGTGTCTGCAATTCACGTCAATTCTCGAGTGTCACGTCGTTCTTCAACGATCTACGAGCCAAGT

GATCCACCTTTCGGAATGTACTTTCCTGCGCGTACAGCAGTTCGAATGGTACTAGCGCTCATTGCGCCTACAGCAATGTC

TGCGTGTCGAAGGCACGTCGGCATGTGTAGTACAACGCCTTGTGCACTGATGAGCGGCTATTTTC

>KP336477.1-Macaca_fuscata-Italy Trichuris sp. MCTV isolate MCTV14 internal transcribed spacer 1, partial sequence; 5.8S ribosomal RNA gene and internal transcribed spacer 2, complete sequence; and 28S ribosomal RNA gene, partial sequence

TCGGTCGAGCGGTGCCCGCCGCAGCAAGGCGGGCGGTATCGTCGTCGTCGACTGCTGCTGCCGTGCAACGACGTCGAACC

CTTGGTGCCAGCGCGTGCGGTCACCGCTCGGTGCTCAACTGTCGATTCGCGCTGCTTTACGCCGACGGCGGGGCCAGTAG

CCGAGTCGGGTAGTCTTCGCACTACGCTGACTTGGCCGCTCTCCGCTGCTCCGGCTAGCAGTTGCCATCGCGGCTCGCTG

GCAACGGAGCTGCGGCGTCACGCTCGCTCACGGGTGCAGGTTTGTCCAGTGCTCGCCACCAACCTCAACCTGTAGCCTGA

GCGGCGCATTCGCTATTGCTTAACGACCCTCAGACAGGCGTAGCGTCGACATCTTTGATCGACGCTGCAATGTGCGTTCA

AAGTATTCAAGTTCGGTGTGTCTGCAATTCACGTCAATTCTCGAGTGTCACGTCGTTCTTCAACGATCTACGAGCCAAGT

GATCCACCGTTCGGAATGTACTTTCCTGCGCGTACAGCAGTTCGAATGGTACTAGCGCTCATTGCGCCGACAGCAATGTC

TGCGTGTCGAAGGCACGTCGGCATGTGTAGTACAACGCCTTGTGCACTGGTGAACGGCTATTTTC

>KP336467.1-Macaca_fuscata-Italy Trichuris sp. MCTV isolate MCTV19 internal transcribed spacer 1, partial sequence; 5.8S ribosomal RNA gene and internal transcribed spacer 2, complete sequence; and 28S ribosomal RNA gene, partial sequence

TCGGTCGAGCGGTGCCCGCCGCAGCAAGGCGGGCGGTATCGTCGTCGTCGACTGCTGCTGCCGTGCAACGACGTCGAACA

CTTGCTGCGAGCGCGTGCGGTCACCGCTCGGTGCTCAACTGTCGATTCGCGCTGCTGTACGCCGACGGCGCGGCCAGTAG

CCGAGTCGGGTAGTCTTCGCACTACGCTGACTTGGCCGCTCTCCGCTGCTCCGGCTAGCAGTTGCCATCGCGGCTCGCTG

GCAACGGAGCTGCGGCGTCACGCTCGCTCGCGGGTGCAGGTTTGTCCAGTGCTCGCCACCAACCTCAACCTGTAGCCTGA

GCGGCGCATTCGCTATTGCTTAACGACCCTCAGACAGGCGTAGCGTCGACATCTTTGATCGACGCTGCAATGTGCGTTCA

AAGTATTCAAGTTCGGTGTGTCTGCCATTAAAGTCAATTCTGGAATGTCACATCGTTCTTCAACTATCTACGAGCCAAGT

GATCCACCGTTCGGAATGTACTTTCCTGCGCGTACAGCAGTTCGAATGGTACTAGCGCTCATTGCGCCGACAGCAATGTC

TGCGTGTCGAAGGCACGTCGGCATGTGTAGTACAACGCCTTGTGCACTGGTGAACGGCTATTTTC

>Worm_3-4-4_5.8S_ITS2

TCGGTCGAGCGGTGCCCGCCGCCGCAGCAAGGCGAACGGTTTCGTCGTCGTCGTCGACTGCTGCTGCCGCTGCTGCTGCC

GTGCAACTACGACGAACACTTGCTGCTACGAGCGCGTGCGGTCACCGCTCCCTGCTCAACTGCTGTACGCCGACGGCGCG

GCCGGTAGCCAAGTCGGGTAGTCTTCGCCCTACGCTGACTTGGCCGCTCTCCGCTGCTCCGCCTACCAGTTGCCATCGCG

GCTCGCTGGCAACGGAGCTGCGGCGTCACGCTCGCTCGCGCGCGGATGCAGGTTTGTCCGGTGCTCGCCACCAACCTCAA

CCTGTAGCCTGAGCGGCGCATTCGCTATTATGCTTAACGACCCTCAGACAGGCGTAGCGTCGACATCTTTGATCGACGCT

GCAATGTGCGTTCAAAGTATTCAAGTTCGGTGTGTCTGCAATTCACGTCAATTCTCGAGTGTCACGTCGTTCTTCAACGA

TCTACGAGCCAAGTGATCCACCGTTCGGAATGTACTTTCCTGCGCGTACAGCAGCAGTTCGAATGCTACTAGCGCTCATT

GCGCCGACAGCAGTGTCTGCGTGTCGAAGGCACATCGGCGTGCGTAGTAGGACGACGCCTTGTGCACTGGTGAAGCTGCT

TTTTC

>Worm_3-4-5_5.8S_ITS2

TCGGTCGAGCGGTGCCCGCCGCCGCAGCAAGGCGAACGGTTTCGTCGTCGTCGTCGACTGCTGCTGCCGCTGCTGCTGCC

GTGCAACTACGACGAACACTTGCTGCTACGAGCGCGTGCGGTCACCGCTCCCTGCTCAACTGCTGTACGCCGACGGCGCG

GCCGGTAGCCAAGTCGGGTAGTCTTCGCCCTACGCTGACTTGGCCGCTCTCCGCTGCTCCGCCTACCAGTTGCCATCGCG

GCTCGCTGGCAACGGAGCTGCGGCGTCACGCTCGCTCGCGCGCGGATGCAGGTTTGTCCGGTGCTCGCCACCAACCTCAA

CCTGTAGCCTGAGCGGCGCATTCGCTATTATGCTTAACGACCCTCAGACAGGCGTAGCGTCGACATCTTTGATCGACGCT

GCAATGTGCGTTCAAAGTATTCAAGTTCGGTGTGTCTGCAATTCACGTCAATTCTCGAGTGTCACGTCGTTCTTCAACGA

TCTACGAGCCAAGTGATCCACCGTTCGGAATGTACTTTCCTGCGCGTACAGCAGCAGTTCGAATGCTACTAGCGCTCATT

GCGCCGACAGCAGTGTCTGCGTGTCGAAGGCACATCGGCGTGCGTAGTAGGACGACGCCTTGTGCACTGGTGAAGCTGCT

TTTTC

>Worm_3-4-8_5.8S_ITS2

TCGGTCGAGCGGTGCCCGCCGCCGCAGCAAGGCGAACGGTTTCGTCGTCGTCGTCGACTGCTGCTGCCGCTGCTGCTGCC

GTGCAACTACGACGAACACTTGCTGCTACGAGCGCGTGCGGTCACCGCTCCCTGCTCAACTGCTGTACGCCGACGGCGCG

GCCGGTAGCCAAGTCGGGTAGTCTTCGCCCTACGCTGACTTGGCCGCTCTCCGCTGCTCCGCCTACCAGTTGCCATCGCG

GCTCGCTGGCAACGGAGCTGCGGCGTCACGCTCGCTCGCGCGCGGATGCAGGTTTGTCCGGTGCTCGCCACCAACCTCAA

CCTGTAGCCTGAGCGGCGCATTCGCTATTATGCTTAACGACCCTCAGACAGGCGTAGCGTCGACATCTTTGATCGACGCT

GCAATGTGCGTTCAAAGTATTCAAGTTCGGTGTGTCTGCAATTCACGTCAATTCTCGAGTGTCACGTCGTTCTTCAACGA

TCTACGAGCCAAGTGATCCACCGTTCGGAATGTACTTTCCTGCGCGTACAGCAGCAGTTCGAATGCTACTAGCGCTCATT

GCGCCGACAGCAGTGTCTGCGTGTCGAAGGCACATCGGCGTGCGTAGTAGGACGACGCCTTGTGCACTGGTGAAGCTGCT

TTTTC

>Worm_3-4-13_5.8S_ITS2_1

TCGGTCGAGCGGTGCCCGCCGCCGCAGCAAGGCGAACGGTTTCGTCGTCGTCGTCGTCGACTGCTGCTGCCGCTGCTGCC

GTGCAACGACGACGAACACTTGCTGCTACGAGCGCGTGCGATCACCGCTCCCTGCTCAACTGCTGTACGCCGACGGCGCG

GCCGGTAGCCAAGTCGGGTAGTCTTCGCCCTACGCTGACTTGGCCGCTCTCCGCTGCTCCGCCTACCAGTTGCCATCGCG

GCTCGCTGGCAACGGAGCTGCGGCGTCACGCTCGCTCGCGCGCGGATGCAGGTTTGTCCGGTGCTCGCCACCAACCTCAA

CCTGTAGCCTGAGCGGCGCATTCGCTATTATGCTTAACGACCCTCAGACAGGCGTAGCGTCGACATCTTTGATCGACGCT

GCAATGTGCGTTCAAAGTATTCAAGTTCGGTGTGTCTGCAATTCACGTCAATTCTCGAGTGTCACGTCGTTCTTCAACGA

TCTACGAGCCAAGTGATCCACCGTTCGGAATGTACTTTCCTGCGCGTACAGCAGCAGTTCGAATGCTACTAGCGCTCATT

GCGCCGACAGCAGTGTCTGCGTGTCGAAGGCACATCGGCGTGCGTAGTAGGACGACGCCTTGTGCACTGGTGAAGCTGCT

TTTTC

>Worm_10-5-6_5.8S_ITS2

TCGGTCGAGCGGTGCCCGCCGCCGCAGCAAGGCGAACGGTTTCGTCGTCGTCGTCGTCGACTGCTGCTGCCGCTGCTGCC

GTGCAACGACGACGAACACTTGCTGCTACGAGCGCGTGCGATCACCGCTCCCTGCTCAACTGCTGTACGCCGACGGCGCG

GCCGGTAGCCAAGTCGGGTAGTCTTCGCCCTACGCTGACTTGGCCGCTCTCCGCTGCTCCGCCTACCAGTTGCCATCGCG

GCTCGCTGGCAACGGAGCTGCGGCGTCACGCTCGCTCGCGCGCGGATGCAGGTTTGTCCGGTGCTCGCCACCAACCTCAA

CCTGTAGCCTGAGCGGCGCATTCGCTATTATGCTTAACGACCCTCAGACAGGCGTAGCGTCGACATCTTTGATCGACGCT

GCAATGTGCGTTCAAAGTATTCAAGTTCGGTGTGTCTGCAATTCACGTCAATTCTCGAGTGTCACGTCGTTCTTCAACGA

TCTACGAGCCAAGTGATCCACCGTTCGGAATGTACTTTCCTGCGCGTACAGCAGCAGTTCGAATGCTACTAGCGCTCATT

GCGCCGACAGCAGTGTCTGCGTGTCGAAGGCACATCGGCGTGCGTAGTAGGACGACGCCTTGTGCACTGGTGAAGCTGCT

TTTTC

>Worm_10-5-9_5.8S_ITS2

TCGGTCGAGCGGTGCCCGCCGCCGCAGCAAGGCGAACGGTTTCGTCGTCGTCGTCGACTGCTGCTGCCGCTGCTGCTGCC

GTGCAACTACGACGAACACTTGCTGCTACGAGCGCGTGCGGTCACCGCTCCCTGCTCAACTGCTGTACGCCGACGGCGCG

GCCGGTAGCCAAGTCGGGTAGTCTTCGCCCTACGCTGACTTGGCCGCTCTCCGCTGCTCCGCCTACCAGTTGCCATCGCG

GCTCGCTGGCAACGGAGCTGCGGCGTCACGCTCGCTCGCGCGCGGATGCAGGTTTGTCCGGTGCTCGCCACCAACCTCAA

CCTGTAGCCTGAGCGGCGCATTCGCTATTATGCTTAACGACCCTCAGACAGGCGTAGCGTCGACATCTTTGATCGACGCT

GCAATGTGCGTTCAAAGTATTCAAGTTCGGTGTGTCTGCAATTCACGTCAATTCTCGAGTGTCACGTCGTTCTTCAACGA

TCTACGAGCCAAGTGATCCACCGTTCGGAATGTACTTTCCTGCGCGTACAGCAGCAGTTCGAATGCTACTAGCGCTCATT

GCGCCGACAGCAGTGTCTGCGTGTCGAAGGCACATCGGCGTGCGTAGTAGGACGACGCCTTGTGCACTGGTGAAGCTGCT

TTTTC

#### ITS2 Haplotypes

>Hap_1

TCGGTCGAGCGGTGCC---CGCCGCCGCAGCAAGGCGAACGGTTTCG---TCGTCGTCGTCGACTGCTGCT------GCC

GCTGCTGCCGTGCAACGACGACGAACACTTGCTGCTACGAGCGCGTGCGGTCACCGCTCCCTGCTCA-------------

GCTGCTGAACGCCGACGGCGCGGCCGGTAGCCAAGTCGGGTAGTCTTCGCCCTACGCTGACTTGGCCGCTCTCCGCTGCT

CCGCCTACCAGTTGCCATCGCGGCTCGCTGGCAACGGAGCTGCGGCGTCACGCTCGCTCGCGCGCGGATGCAGGTTTGTC

CGGTGCTCGCCACCAACCTCAACCTGTAGCCTGAGCGGCGCATTCGCTATTATGCTTAACGACCCTCAGACAGGCGTAGC

GTCGACATCTTTGATCGACGCTGCAATGTGCGTTCAAAGTATTCAAGTTCGGTGTGTCTGCAATTCACGTCAATTCTCGA

GTGTCACGTCGTTCTTCAACGATCTACGAGCCAAGTGATCCACCGTTCGGAATGTACTTTCCTGCGCGTACAGCAGCAGT

TCGAATGCTACTAGCGCTCATTGCGCCGACAGCAGTGTCTGCGTGTCGAAGGCACATCGGCGTGCGTAGTAGGACGACGC

CTTGTGCACTGGTGAAGCTGCC-TTTTC

>Hap_2

TCGGTCGAGCGGTGCC---CGCCGCCGCAGCAAGGCGAACGGTT------TCGTCGTCGTCGACTGCTGCT------GCC

GCTGCTGCCGTGCAACGACGACGAACACTTGCTGCTACGAGCGCGTGCGGTCACCGCTCCCTGCTCAA------------

-CTGCTGTACGCCGACGGCGCGGCCGGTAGCCAAGTCGGGTAGTCTTCGCCCTACGCTGACTTGGCCGCTCTCCGCTGCT

CCGCCTACCAGTTGCCATCGCGGCTCGCTGGCAACGGAGCTGCGGCGTCACGCTCGCTCGCGCGCGGATGCAGGTTTGTC

CGGTGCTCGCCACCAACCTCAACCTGTAGCCTGAGCGGCGCATTCGCTATTATGCTTAACGACCCTCAGACAGGCGTAGC

GTCGACATCTTTGATCGACGCTGCAATGTGCGTTCAAAGTATTCAAGTTCGGTGTGTCTGCAATTCACGTCAATTCTCGA

GTGTCACGTCGTTCTTCAACGATCTACGAGCCAAGTGATCCACCGTTCGGAATGTACTTTCCTGCGCGTACAGCAGCAGT

TCGAATGCTACTAGCGCTCATTGCGCCGACAGCAGTGTCTGCGTGTCGAAGGCACATCGGCGTGCGTAGTAGGACGACGC

CTTGTGCACTGGTGAAGCTGCT-TTTTC

>Hap_3

TCGGTCGAGCGGTGCC---CGCCGCCGCAGCAAGGCGAACGGTTTCGTCGTCGTCGTCGTCGACTGCTGCT------GCC

GCTGCTGCCGTGCAACGACGACGAACACTTGCTGCTACGAGCGCGTGCGATCACCGCTCCCTGCTCAA------------

-CTGCTGTACGCCGACGGCGCGGCCGGTAGCCAAGTCGGGTAGTCTTCGCCCTACGCTGACTTGGCCGCTCTCCGCTGCT

CCGCCTACCAGTTGCCATCGCGGCTCGCTGGCAACGGAGCTGCGGCGTCACGCTCGCTCGCGCGCGGATGCAGGTTTGTC

CGGTGCTCGCCACCAACCTCAACCTGTAGCCTGAGCGGCGCATTCGCTATTATGCTTAACGACCCTCAGACAGGCGTAGC

GTCGACATCTTTGATCGACGCTGCAATGTGCGTTCAAAGTATTCAAGTTCGGTGTGTCTGCAATTCACGTCAATTCTCGA

GTGTCACGTCGTTCTTCAACGATCTACGAGCCAAGTGATCCACCGTTCGGAATGTACTTTCCTGCGCGTACAGCTGCAGT

TCGAATGCTACTAGCGCTCATTGCGCCGACAGCAGTGTCTGCGTGTCGAAGGCACATCGGCGTGCGTAGTAGGACGACGC

CTTGTGCACTGGTGAAGCTGCT-TTTTC

>Hap_4

TCGGTCGAGCGGTGCC---CGCCGCCGCAGCAAGGCGAACGGTTTCGTCGTCGTCGTCGTCGACTGCTGCT------GCC

GCTGCTGCCGTGCAACGACGACGAACACTTGCTGCTACGAGCGCGTGCGGTCACCGCTCCCTGCTCA-------------

GCTGCTGAACGCCGACGGCGCGGCCGGTAGCCAAGTCGGGTAGTCTTCGCCCTACGCTGACTTGGCCGCTCTCCGCTGCT

CCGCCTACCAGTTGCCATCGCGGCTCGCTGGCAACGGAGCTGCGGCGTCACGCTCGCTCGCGCGCGGATGCAGGTTTGTC

CGGTGCTCGCCACCAACCTCAACCTGTAGCCTGAGCGGCGCATTCGCTATTATGCTTAACGACCCTCAGACAGGCGTAGC

GTCGACATCTTTGATCGACGCTGCAATGTGCGTTCAAAGTATTCAAGTTCGGTGTGTCTGCAATTCACGTCAATTCTCGA

GTGTCACGTCGTTCTTCAACGATCTACGAGCCAAGTGATCCACCGTTCGGAATGTACTTTCCTGCGCGTACAGCAGCAGT

TCGAATGCTACTAGCGCTCATTGCGCCGACAGCAGTGTCTGCGTGTCGAAGGCACATCGGCGTGCGTAGTAGGACGACGC

CTTGTGCACTGGTGAAGCTGCC-TTTTC

>Hap_5

TCGGTCGAGCGGTGCC---CGCCGCCGCAGCAAGGCGAACGGTTTCG---TCGTCGTCGTCGACTGCTGCTGCCGCTGCT

GCTGCTGCCGTGCAACTACGACGAACACTTGCTGCTACGAGCGCGTGCGGTCACCGCTCCCTGCTCAA------------

-CTGCTGTACGCCGACGGCGCGGCCGGTAGCCAAGTCGGGTAGTCTTCGCCCTACGCTGACTTGGCCGCTCTCCGCTGCT

CCGCCTACCAGTTGCCATCGCGGCTCGCTGGCAACGGAGCTGCGGCGTCACGCTCGCTCGCGCGCGGATGCAGGTTTGTC

CGGTGCTCGCCACCAACCTCAACCTGTAGCCTGAGCGGCGCATTCGCTATTATGCTTAACGACCCTCAGACAGGCGTAGC

GTCGACATCTTTGATCGACGCTGCAATGTGCGTTCAAAGTATTCAAGTTCGGTGTGTCTGCAATTCACGTCAATTCTCGA

GTGTCACGTCGTTCTTCAACGATCTACGAGCCAAGTGATCCACCGTTCGGAATGTACTTTCCTGCGCGTACAGCAGCAGT

TCGAATGCTACTAGCGCTCATTGCGCCGACAGCAGTGTCTGCGTGTCAAAGGCACATCGGCGTGCGTAGTAGGACGACGC

CTTGTGCACTGGTGAAGCTGCT-TTTTC

>Hap_6

TCGGTCGAGCGGTGCC---CGCCGCCGCAGCAAGGCGAACGGTTTCGTCGTCGTCGTCGTCGACTGCTGCT---GCTGCT

GCTGCTGCCGTGCAACGACGACGAACACTTGCTGCTACGAGCGCGTGCGGTCACCGCTCCCTGCTCAA------------

-CTGCTGTACGCCGACGGCGCGGCCGGTAGCCAAGTCGGGTAGTCTTCGCCCTACGCTGACTTGGCCGCTCTCCGCTGCT

CCGCCTACCAGTTGCCATCGCGGCTCGCTGGCAACGGAGCTGCGGCGTCACGCTCGCTCGCGCGCGGATGCAGGTTTGTC

CGGTGCTCGCCACCAACCTCAACCTGTAGCCTGAGCGGCGCATTCGCTATTATGCTTAACGACCCTCAGACAGGCGTAGC

GTCGACATCTTTGATCGACGCTGCAATGTGCGTTCAAAGTATTCAAGTTCGGTGTGTCTGCAATTCACGTCAATTCTCGA

GTGTCACGTCGTTCTTCAACGATCTACGAGCCAAGTGATCCACCGTTCGGAATGTACTTTCCTGCGCGTACAGCAGCAGT

TCGAATGCTACTAGCGCTCATTGCGCCGACAGCAGTGTCTGCGTGTCGAAGGCACATCGGCGTGCGTAGTAGGACGACGC

CTTGTGCACTGGTGAAGCTGCT-TTTTC

>Hap_7

TCGGTCGAGCGGTGCC---CGCCGCCGCAGCAAGGCGAACGGTTTCGTCGTCGTCGTCGTCGACTGCTGCTGCCGCTGCT

GCTGCTGCCGTGCAACTACGACGAACACTTGCTGCTACGAGCGCGTGCGGTCACCGCTCCCTGCTCAA------------

-CTGCTGTACGCCGACGGCGCGGCCGGTAGCCAAGTCGGGTAGTCTTCGCCCTACGCTGACTTGGCCGCTCTCCGCTGCT

CCGCCTACCAGTTGCCATCGCGGCTCGCTGGCAACGGAGCTGCGGCGTCACGCTCGCTCGCGCGCGGATGCAGGTTTGTC

CGGTGCTCGCCACCAACCTCAACCTGTAGCCTGAGCGGCGCATTCGCTATTATGCTTAACGACCCTCAGACAGGCGTAGC

GTCGACATCTTTGATCGACGCTGCAATGTGCGTTCAAAGTATTCAAGTTCGGTGTGTCTGCAATTCACGTCAATTCTCGA

GTGTCACGTCGTTCTTCAACGATCTACGAGCCAAGTGATCCACCGTTCGGAATGTACTTTCCTGCGCGTACAGCAGCAGT

TCGAATGCTACTAGCGCTCATTGCGCCGACAGCAGTGTCTGCGTGTCGAAGGCACATCGGCGTGCGTAGTAGGACGACGC

CTTGTGCACTGGTGAAGCTGCT-TTTTC

>Hap_8

TCGGTCGAGCGGTGCCCGCCGCCGCCGCAGCAAGGCGAACGGTTTCG---TCGTCGTCGTCGACTGCTGCT------GCC

GCTGCTGCCGTGCAACGACGACGAACACTTGCTGCTACGAGCGCGTGCGGTCACCGCTCCCTGCTCAA------------

-CTGCTGTACGCCGACGGCGCGGCCGGTAGCCAAGTCGGGTAGTCTTCGCCCTACGCTGACTTGGCCGCTCTCCGCTGCT

CCGCCTACCAGTTGCCATCGCGGCTCGCTGGCAACGGAGCTGCGGCGTCACGCTCGCTCGCGCGCGGATGCAGGTTTGTC

CGGTGCTCGCCACCAACCTCAACCTGTAGCCTGAGCGGCGCATTCGCTATTATGCTTAACGACCCTCAGACAGGCGTAGC

GTCGACATCTTTGATCGACGCTGCAATGTGCGTTCAAAGTATTCAAGTTCGGTGTGTCTGCAATTCACGTCAATTCTCGA

GTGTCACGTCGTTCTTCAACGATCTACGAGCCAAGTGATCCACCGTTCGGAATGTACTTTCCTGCGCGTACAGCAGCAGT

TCGAATGCTACTAGCGCTCATTGCGCCGACAGCAGTGTCTGCGTGTCGAAGGCACATCGGCGTGCGTAGTAGGACGACGC

CTTGTGCACTGGTGAAGCTGCC-TTTTC

>Hap_9

TCGGTCGAGCGGTGCCCGCCGCCGCCGCAGCAAGGCGAACGGTTTCGTCGTCGTCGTCGTCGACTGCTGCT------GCC

GCTGCTGCCGTGCAACGACGACGAACACTTGCTGCTACGAGCGCGTGCGATCACCGCTCCCTGCTCAA------------

-CTGCTGTACGCCGACGGCGCGGCCGGTAGCCAAGTCGGGTAGTCTTCGCCCTACGCTGACTTGGCCGCTCTCCGCTGCT

CCGCCTACCAGTTGCCATCGCGGCTCGCTGGCAACGGAGCTGCGGCGTCACGCTCGCTCGCGCGCGGATGCAGGTTTGTC

CGGTGCTCGCCACCAACCTCAACCTGTAGCCTGAGCGGCGCATTCGCTATTATGCTTAACGACCCTCAGACAGGCGTAGC

GTCGACATCTTTGATCGACGCTGCAATGTGCGTTCAAAGTATTCAAGTTCGGTGTGTCTGCAATTCACGTCAATTCTCGA

GTGTCACGTCGTTCTTCAACGATCTACGAGCCAAGTGATCCACCGTTCGGAATGTACTTTCCTGCGCGTACAGCAGCAGT

TCGAATGCTACTAGCGCTCATTGCGCCGACAGCAGTGTCTGCGTGTCGAAGGCACATCGGCGTGCGTAGTAGGACGACGC

CTTGTGCACTGGTGAAGCTGCT-TTTTC

>Hap_10

TCGGTCGAGCGGTGCC---CGCCGCCGCAGCAAGGCGAACGGTTTCG---TCGTCGTCGTCGACTGCTG---C-------

--TGCTGCCGTGCAACTACGACGAACACTTGCTGCTACGAGCGCGTGCGGTCACCGCTCCCTGCTCAA------------

-CTGCTGTACGCCGACGGCGCGGCCGGTAGCCAAGTCGGGTAGTCTTCGCCCTACGCTGACTTGGCCGCTCTCCGCTGCT

CCGCCTACCAGTTGCCATCGCGGCTCGCTGGCAACGGAGCTGCGGCGTCACGCTCGCTCGCGCGCGGATGCAGGTTTGTC

CGGTGCTCGCCACCAACCTCAACCTGTAGCCTGAGCGGCGCATTCGCTATTATGCTTAACGACCCTCAGACAGGCGTAGC

GTCGACATCTTTGATCGACGCTGCAATGTGCGTTCAAAGTATTCAAGTTCGGTGTGTCTGCAATTCACGTCAATTCTCGA

GTGTCACGTCGTTCTTCAACGATCTACGAGCCAAGTGATCCACCGTTCGGAATGTACTTTCCTGCGCGTACAGCAGCAGT

TCGAATGCTACTAGCGCTCATTGCGCCGACAGCAGTGTCTGCGTGTCGAAGGCACATCGGCGTGCGTAGTAGGACGACGC

CTTGTGCACTGGTGAAGCTGCT-TTTTC

>Hap_11

TCGGTCGAGCGGTGCCCGCCGCCGCCGCAGCAAGGCGAACGGTT------TCGTCGTCGTCGACTGCT------------

---GCTGCCGTGCAACTACGACGAACACTTGCTGCTACGAGCGCGTGCGATCACCGCTCCCTGCTCAA------------

-CTGCTGTACGCCGACGGCGCGGCCGGTAGCCAAGTCGGGTAGTCTTCGCCCTACGCTGACTTGGCCGCTCTCCGCTGCT

CCGCCTACCAGTTGCCATCGCGGCTCGCTGGCAACGGAGCTGCGGCGTCACGCTCGCTCGCGCGCGGATGCAGGTTTGTC

CGGTGCTCGCCACCAACCTCAACCTGTAGCCTGAGCGGCGCATTCGCTATTATGCTTAACGACCCTCAGACAGGCGTAGC

GTCGACATCTTTGATCGACGCTGCAATGTGCGTTCAAAGTATTCAAGTTCGGTGTGTCTGCAATTCACGTCAATTCTCGA

GTGTCACGTCGTTCTTCAACGATCTACGAGCCAAGTGATCCACCGTTCGGAATGTACTTTCCTGCGCGTACAGCAGCAGT

TCGAATGCTACTAGCGCTCATTGCGCCGACAGCAGTGTCTGCGTGTCGAAGGCACATCGGCGTGCGTAGTAGGACGACGC

CTTGTGCACTGGTGAAGCTGCC-TTTTC

>Hap_12

TCGGTCGAGCGGTGCC---CGCCGCCGCAGCAAGGCGAACGGTTTCG---TCGTCGTCGTCGACTGCTG---C-------

--TGCTGCCGTGCAACTACGACGAACACTTGCTGCTACGAGCGCGTGCGGTCACCGCTCCCTGCTCAA------------

-CTGCTGTACGCCGACGGCGCGGCCGGTAGCCAAGTCGGGTAGTCTTCGCCCTACGCTGACTTGGCCGCTCTCCGCTGCT

CCGCCTACCAGTTGCCATCGCGGCTCGCTGGCAACGGAGCTGCGGCGTCACGCTCGCTCGCGCGCGGATGCAGGTTTGTC

CGGTGCTCGCCACCAACCTCAACCTGTAGCCTGAGCGGCGCATTCGCTATTATGCTTAA---------------------

--------CTTTGATCGACGCTGCAATGTGCGTTCAAAGTATTCAAGTTCGGTGTGTCTGCAATTCACGTCAATTCTCGA

GTGTCACGTCGTTCTTCAACGATCTACGAGCCAAGTGATCCACCGTTCGGAATGTACTTTCCTGCGCGTACAGCAGCAGT

TCGAATGCTACTAGCGCTCATTGCGCCGACAGCAGTGTCTGCGTGTCGAAGGCACATCGGCGTGCGTAGTAGGACGACGC

CTTGTGCACTGGTGAAGCTGCT-TTTTC

>Hap_13

TCGGTCGAGCGGTGC------CGGCCGCAGCAAGGCGAACGGTG------TCGTCGTCGTCGGCTGCT------------

---GCTGCCGTCGAACGACGACGAACACTTGCTG---CGAGCGCGTGCGGTCACCGCTCGCTGCTCAACTGTCCAGTCGC

GCTGCTGTACGCCGACGGCGCGGCCGGTAGCCAAGTCGGGTAGTCTTCGCACTACGCTGACTTGGCCGCTCTCCGCTGCT

CCGCCTACCAGTTGCCATCGCCGCTCGCTGGCAACGGAGCTGCGGCGTCACGCTCGCTCGC---CGGATGCAGGTTTGTC

CAGTGCTCGCCACCAACCTCAACCTGTAGCCTGAGCGGCGCATTCGCTATT--GCTTAACGACCCTCAGACAGGCGTAGC

GTCGACATCTTTGATCGACGCTGCAATGTGCGTTCAAAGTATTCAAGTTCGGTGTGTCTGCAATTCACGTCAATTCTCGA

GTGTCACGTCGTTCTTCAACGATCTACGAGCCAAGTGATCCACCGTTCGGAATGTACTTTCCTGCGCGTACAGCAGCAGT

TCGAATGCTACTAGCGCTCATTGCGCCGACAGCAGTGTCTGCGTGTCGAAGGCACATCGGCGTGCGTAGTAGGACGACGC

CTTGTGCACTGGTGAAGCTGCC-TTTTC

>Hap_14

TCGGTCGAGCGGTGCC---CGCCGCCGCAGCAAGGCGAACGGTTTCG---TCGTCGTCGTCGACTGCTGCT---GCCGCT

GCTGCTGCCGTGTAACTACGACGAACACTTGCTGCTACGAGCGCGTGCGGTCACCGCTCCCTGCTCAA------------

-CTGCTGTACGCCGACGGCGCGGCCGGTAGCCAAGTCGGGTAGTCTTCGCCCTACGCTGACTTGGCCGCTCTCCGCTGCT

CCGCCTACCAGTTGCCATCGCGGCTCGCTGGCAACGGAGCTGCGGCGTCACGCTCGCTCGCGCGCGGATGCAGGTTTGTC

CGGTGCTCGCCACCAACCTCAACCTGTAGCCTGAGCGGCGCATTCGCTATTATGCTTAACGACCCTCAGACAGGCGTAGC

GTCGACATCTTTGATCGACGCTGCAATGTGCGTTCAAAGTATTCAAGTTCGGTGTGTCTGCAATTCACGTCAATTCTCGA

GTGTCACGTCGTTCTTCAACGATCTACGAGCCAAGTGATCCACCGTTCGGAATGTACTTTCCTGCGCGTACAGCTGCAGT

TCGAATGCTACTAGCGCTCATTGCGCCGACAGCAGTGTCTGCGTGTCGAAGGCACATCGGCGTGCGTAGTAGGACGACGC

CTTGTGCACTGGTGAAGCTGCT-TTTTC

>Hap_15

TCGGTCGAGCGGTGCC---CGCCGCCGCAGCAAGGCGAACGGTTTCG---TCGTCGTCGTCGACTGCTGCT---GCCGCT

GCTGCTGCCGTGCAACGACGACGAACACTTGCTGCTACGAGCGCGTGCGGTCACCGCTCCCTGCTCAA------------

-CTGCTGTACGCCGACGGCGCGGCCGGTAGCCAAGTCGGGTAGTCTTCGCCCTACGCTGACTTGGCCGCTCTCCGCTGCT

CCGCCTACCAGTTGCCATCGCGGCTCGCTGGCAACGGAGCTGCGGCGTCACGCTCGCTCGCGCGCGGATGCAGGTTTGTC

CGGTGCTCGCCACCAACCTCAACCTGTAGCCTGAGCGGCGCATTCGCTATTATGCTTAACGACCCTCAGACAGGCGTAGC

GTCGACATCTTTGATCGACGCTGCAATGTGCGTTCAAAGTATTCAAGTTCGGTGTGTCTGCAATTCACGTCAATTCTCGA

GTGTCACGTCGTTCTTCAACGATCTACGAGCCAAGTGATCCACCGTTCGGAATGTACTTTCCTGCGCGTACAGCAGCAGT

TCGAATGCTACTAGCGCTCATTGCGCCGACAGCAGTGTCTGCGTGTCGAAGGCACATCGGCGTGCGTAGTAGGACGACGC

CTTGTGCACTGGTGAAGCTGCT-TTTTC

>Hap_16

TCGGTCGAGCGGTGCC---CGCCGCCGCAGCAAGGCGAACGGTTTCG---TCGTCGTCGTCGACTGCTGCT---GCTGCT

GCTGCTGCCGTGCAACTACGACGAACACTTGCTGCTACGAGCGCGTGCGGTCACCGCTCCCTGCTCAA------------

-CTGCTGTACGCCGACGGCGCGGCCGGTAGCCAAGTCGGGTAGTCTTCGCCCTACGCTGACTTGGCCGCTCTCCGCTGCT

CCGCCTACCAGTTGCCATCGCGGCTCGCTGGCAACGGAGCTGCGGCGTCACGCTCGCTCGCGCGCGGATGCAGGTTTGTC

CGGTGCTCGCCACCAACCTCAACCTGTAGCCTGAGCGGCGCATTCGCTATTATGCTTAACGACCCTCAGACAGGCGTAGC

GTCGACATCTTTGATCGACGCTGCAATGTGCGTTCAAAGTATTCAAGTTCGGTGTGTCTGCAATTCACGTCAATTCTCGA

GTGTCACGTCGTTCTTCAACGATCTACGAGCCAAGTGATCCACCGTTCGGAATGTACTTTCCTGCGCGTACAGCAGCAGT

TCGAATGCTACTAGCGCTCATTGCGCCGACAGCAGTGTCTGCGTGTCGAAGGCACATCGGCGTGCGTAGTAGGACGACGC

CTTGTGCACTGGTGAAGCTGCT-TTTTC

>Hap_17

TCGGTCGAGCGGTGCC---CGCCGCCGCAGCAAGGGGAACGGTTTCG---TCGTCGTCGTCGACTGCTGCT---GCCGCT

GCTGCTGCCGTGCAACTACGACGAACACTTGCTGCTACGAGCGCGTGCGGTCACCGCTCCCTGCTCAA------------

-CTGCTGTACGCCGACGGCGCGGCCGGTAGCCAAGTCGGGTAGTCTTCGCCCTACGCTGACTTGGCCGCTCTCCGCTGCT

CCGCCTACCAGTTGCCATCGCGGCTCGCTGGCAACGGAGCTGCGGCGTCACGCTCGCTCGCGCGCGGATGCAGGTTTGTC

CGGTGCTCGCCACCAACCTCAACCTGTAGCCTGAGCGGCGCATTCGCTATTATGCTTAACGACCCTCAGACAGGCGTAGC

GTCGACATCTTTGATCGACGCTGCAATGTGCGTTCAAAGTATTCAAGTTCGGTGTGTCTGCAATTCACGTCAATTCTCGA

GTGTCACGTCGTTCTTCAACGATCTACGAGCCAAGTGATCCACCGTTCGGAATGTACTTTCCTGCGCGTACAGCAGCAGT

TCGAATGCTACTAGCGCTCATTGCGCCGACAGCAGTGTCTGCGTGTCGAAGGCACATCGGCGTGCGTAGTAGGACGACGC

CTTGTGCACTGGTGAAGCTGCT-TTTTC

>Hap_18

TCGGTCGAGCGGTGCC---CGCCGCCGCAGCAAGGCGAACGGTTTCG---TCGTCGTCGTCGACTGCTGCT---GCCGCT

GCTGCTGCCGTGCAACTACGACGAACACTTGCTGCTACGAGCGCGTGCGGTCACCGCTCCCTGCTCAA------------

-CTGCTGTACGCCGACGGCGCGGCCGGTAGCCAAGTCGGGTAGTCTTCGCCCTACGCTGACTTGGCCGCTCTCCGCTGCT

CCGCCTACCAGTTGCCATCGCGGCTCGCTGGCAACGGAGCTGCGGCGTCACGCTCGCTCGCGCGCGGATGCAGGTTTGTC

CGGTGCTCGCCACCAACCTCAACCTGTAGCCTGAGCGGCGCATTCGCTATTATGCTTAACGACCCTCAGACAGGCGTAGC

GTCGACATCTTTGATCGACGCTGCAATGTGCGTTCAAAGTATTCAAGTTCGGTGTGTCTGCAATTCACGTCAATTCTCGA

GTGTCACGTCGTTCTTCAACGATCTACGAGCCAAGTGATCCACCGTTCGGAATGTACTTTCCTGCGCGTACAGCAGCAGT

TCGAATGATACTAGCGCTCATTGCGCCGACAGCAGTGTCTGCGTGTCGAAGGCACATCGGCGTGCGTAGTAGGACGACGC

CTTGTGCACTGGTGAAGCCGCT-TTTTC

>Hap_19

TCGGTCGAGCGGTGCC---CGCCGCCGCAGCAAGGCGAACGGTT------TCGTCGTCGTCGACTGCTGCT---GCCGCT

GCTGCTGCCGTGCAACTACGACGAACACTTGCTGCTACGAGCGCGTGCGGTCACCGCTCCCTGCTCAA------------

-CTGCTGTACGCCGACGGCGCGGCCGGTAGCCAAGTCGGGTAGTCTTCGCCCTACGCTGACTTGGCCGCTCTCCGCTGCT

CCGCCTACCAGTTGCCATCGCGGCTCGCTGGCAACGGAGCTGCGGCGTCACGCTCGCTCGCGCGCGGATGCAGGTTTGTC

CGGTGCTCGCCACCAACCTCAACCTGTAGCCTGAGCGGCGCATTCGCTATTATGCTTAACGACCCTCAGACAGGCGTAGC

GTCGACATCTTTGATCGACGCTGCAATGTGCGTTCAAAGTATTCAAGTTCGGTGTGTCTGCAATTCACGTCAATTCTCGA

GTGTCACGTCGTTCTTCAACGATCTACGAGCCAAGTGATCCACCGTTCGGAATGTACTTTCCTGCGCGTACAGCAGCAGT

TCGAATGCTACTAGCGCTCATTGCGCCGACAGCAGTGTCTGCGTGTCGAAGGCACATCGGCGTGCGTAGTAGGACGACGC

CTTGTGCACTGGTGAAGCTGCC-TTTTC

>Hap_20

TCGGTCGAGCGGTGCC---CGCCGCCGCAGCAAGGCGAACGGTT------TCGTCGTCGTCGACTGCTGCT---GCCGCT

GCTGCTGCCGTGCAACGACGACGAACACTTGCTGCTACGAGCGCGTGCGGTCACCGCTCCCTGCTCAA------------

-CTGCTGTACGCCGACGGCGCGGCCGGTAGCCAAGTCGGGTAGTCTTCGCCCTACGCTGACTTGGCCGCTCTCCGCTGCT

CCGCCTACCAGTTGCCATCGCGGCTCGCTGGCAACGGAGCTGCGGCGTCACGCTCGCTCGCGCGCGGATGCAGGTTTGTC

CGGTGCTCGCCACCAACCTCAACCTGTAGCCTGAGCGGCGCATTCGCTATTATGCTTAACGACCCTCAGACAGGCGTAGC

GTCGACATCTTTGATCGACGCTGCAATGTGCGTTCAAAGTATTCAAGTTCGGTGTGTCTGCAATTCACGTCAATTCTCGA

GTGTCACGTCGTTCTTCAACGATCTACGAGCCAAGTGATCCACCGTTCGGAATGTACTTTCCTGCGCGTACAGCAGCAGT

TCGAATGCTACTAGCGCTCATTGCGCCGACAGCAGTGTCTGCGTGTCGAAGGCACATCGGCGTGCGTAGTAGGACGACGC

CTTGTGCACTGGTGAAGCTGCT-TTTTC

>Hap_21

TCGGTCGAGCGGTGCC---CGCCGCCGCAGCAAGGCGAACGGTTTCG---TCGTCGTCGTCGACTGCTGCT------GCC

GCTGCTGCCGTGCAACGACGACGAACACTTGCTGCTACGAGCGCGTGCGGTCACCGCTCCCTGCTCAA------------

-CTGCTGTACGCCGACGGCGCGGCCGGTAGCCAAGTCGGGTAGTCTTCGCCCTACGCTGACTTGGCCGCTCTCCGCTGCT

CCGCCTACCAGTTGCCATCGCGGCTCGCTGGCAACGGAGCTGCGGCGTCACGCTCGCTCGCGCGCGGATGCAGGTTTGTC

CGGTGCTCGCCACCAACCTCAACCTGTAGCCTGAGCGGCGCATTCGCTATTATGCTTAACGACCCTCAGACAGGCGTAGC

GTCGACATCTTTGATCGACGCTGCAATGTGCGTTCAAAGTATTCAAGTTCGGTGTGTCTGCAATTCACGTCAATTCTCGA

GTGTCACGTCGTTCTTCAACGATCTACGAGCCAAGTGATCCACCGTTCGGAATGTACTTTCCTGCGCGTACAGCAGCAGT

TCGAATGCTACTAGCGCTCATTGCGCCGACAGCAGTGTCTGCGTGTCGAAGGCACATCGGCGTGCGTAGTAGGACGACGC

CTTGTGCACTGGTGAAGCTGCT-TTTTC

>Hap_22

TCGGTCGAGCGGTGC------CCGCCGCAGCAAGGCGGGCGGTA------TCGTCGTCGTCGACTGCT------------

---GCTGCCGTGCAACGACGTCGAACACTTGCTG---CGAGCGCGTGCGGTCACCGCTCGGTGCTCAACTGTCGATTCGC

GCTGCTGTACGCCGACGGCGCGGCCAGTAGCCGAGTCGGGTAGTCTTCGCACTACGCTGACTTGGCCGCTCTCCGCTGCT

CCGGCTAGCAGTTGCCATCGCGGCTCGCTGGCAACGGAGCTGCGGCGTCACGCTCGCTCG----CGGGTGCAGGTTTGTC

CAGTGCTCGCCACCAACCTCAACCTGTAGCCTGAGCGGCGCATTCGCTATT--GCTTAACGACCCTCAGACAGGCGTAGC

GTCGACATCTTTGATCGACGCTGCAATGTGCGTTCAAAGTATTCAAGTTCGGTGTGTCTGCAATTCACGTCAATTCTCGA

GTGTCACGTCGTTCTTCAACGATCTACGAGCCAAGTGATCCACCGTTCGGAATGTACTTTCCTGCGCGTA---CAGCAGT

TCGAATGGTACTAGCGCTCATTGCGCCGACAGCAATGTCTGCGTGTCGAAGGCACGTCGGCATGTGTAGT---ACAACGC

CTTGTGCACTGGTGAA-CGGCTATTTTC

>Hap_23

TCGGTCGAGCGGTGC------CCGCCGCAGCAAGGCGGGCGGTA------TCGTCGTCGTCGACTGCT------------

---GCTGCCGTGCAACGACGTCGAACACTTGCTG---CGAGCGCGTGCGGTCACCGCTCGGTGCTCAACTGTCGATTCGC

GCTGCTGTACGCCGACGGCGCGGCCAGTAGCCGAGTCGGGTAGTCTTCGCACTACGCTGACTTGGCCGCTCTCCGCTGCT

CCGGCTAGCAGTTGCCATCGCGGCTCGCTGGCAACGGAGCTGCGGCGTCACGCTCGCTCG----CGGATGCAGGTTTGTC

CAGTGCTCGCCACCAACCTCAACCTGTAGCCTGAGCGGCGCATTCGCTATT--GCTTAACGACCCTCAGACAGGCGTAGC

GTCGACATCTTTGATCGACGCTGCAATGTGCGTTCAAAGTATTCAAGTTCGGTGTGTCTGCAATTCACGTCAATTCTCGA

GTGTCACGTCGTTCTTCAACGATCTACGAGCCAAGTGATCCACCGTTCGGAATGTACTTTCCTGCGCGTA---CAGCAGT

TCGAATGGTACTAGCGCTCATTGCGCCGACAGCAATGTCTGCGTGTCGAAGGCACGTCGGCATGTGTAGT---ACAACGC

CTTGTGCACTGGTGAA-CGGCTATTTTC

>Hap_24

TCGGTCGAGCGGTGC------CCGCCGCAGCAAGGCGGGCGGTA------TCGTCGTCGTCGACTGCT------------

---GCTGCCGTGCAACGACGTCGAACACTTGCTG---CGAGCGCGTGCGGTCACCGCTCGGTGCTCAACTGTCGATTTGC

GCTGCTGTACGCCGACGGCGCGGCCAGTAGCCGAGTCGGGTAGTCTTCGCACTACGCTGACTTGGCCGCTCTCCGCTGCT

CCGGCTAGCAGTTGCCATCGCGGCTCGCTGGCAACGGAGCTGCGGCGTCACGCTCGCTCG----CGGGTGCAGGTTTGTC

CAGTGCTCGCCACCAACCTCAACCTGTAGCCTGAGCGGCGCATTCGCTATT--GCTTAACGACCCTCAGACAGGCGTAGC

GTCGACATCTTTGATCGACGCTGCAATGTGCGTTCAAAGTATTCAAGTTCGGTGTGTCTGCAATTCACGTCAATTCTCGA

GTGTCACGTCGTTCTTCAACGATCTACGAGCCAAGTGATCCACCGTTCGGAATGTACTTTCCTGCGCGTA---CAGCAGT

TCGAATGGTACTAGCGCTCATTGCGCCGACAGCAATGTCTGCGTGTCGAAGGCACGTCGGCATGTGTAGT---ACAACGC

CTTGTGCACTGGTGAA-CGGCTATTTTC

>Hap_25

TCGGTCGAGCGGTGC------CCGCCGCAGCAAGGCGGGCGGTA------TCGTCGTCGTCGACTGCT------------

---GCTGCCGTGCAACGACGTCGAACACTTGCTG---CGAGCGCGTGCGGTCACCGCTCGGTGCTCAACTGTCGATTTGC

GCTGCTGTACGCCGACGGCGCGGCCAGTAGCCGAGTCGGGTAGTCTTCGCACTACGCTGACTTGGCCGCTCTCCGCTGCT

CCGGCTAGCAGTTGCCATCGCGGCTCGCTGGCAACGGAGCTGCGGCGTCACGCTCGCTCG----CGGATGCAGGTTTGTC

CAGTGCTCGCCACCAACCTCAACCTGTAGCCTGAGCGGCGCATTCGCTATT--GCTTAACGACCCTCAGACAGGCGTAGC

GTCGACATCTTTGATCGACGCTGCAATGTGCGTTCAAAGTATTCAAGTTCGGTGTGTCTGCAATTCACGTCAATTCTCGA

GTGTCACGTCGTTCTTCAACGATCTACGAGCCAAGTGATCCACCGTTCGGAATGTACTTTCCTGCGCGTA---CAGCAGT

TCGAATGGTACTAGCGCTCATTGCGCCGACAGCAATGTCTGCGTGTCGAAGGCACGTCGGCATGTGTAGT---ACAACGC

CTTGTGCACTGGTGAA-CGGCTATTTTC

>Hap_26

TCGGTCGAGCGGTGC------CCGCCGCAGCAAGGCGGGCGGTA------TCGTCGTCGTCGACTGCT------------

---GCTGCCGTGCAACGACGTCGAACACTTGCTG---CGAGCGCGTGCGGTCACCGCTCGGTGCTCAACTGTCGATTCGC

GCTGCTGTACGCCGACGGCGCGGCCAGTAGCCGAGTCGGGTAGTCTTCGCACTACGCTGACTTGGCCGCTCTCCGCTGCT

CCGGCTAGCAGTTGCCATCGCGGCTCGCTGGCAACGGAGCTGCGGCGTCACGCTCGCTCA----CGGGTGCAGGTTTGTC

CAGTGCTCACCACCAACCTCAACCTGTAGCCTGAGCGGCGCATTCGCTATT--GCTTAACGACCCTCAGACAGGCGTAGC

GTCGACATCTTTGATCGACGCTGCAATGTGCGTTCAAAGTATTCAAGTTCGGTGTGTCTGCAATTCACGTCAATTCTCGA

GTGTCACGTCGTTCTTCAACGATCTACGAGCCAAGTGATCCACCGTTCGGAATGTACTTTCCTGCGCGTA---CAGCAGT

TCGAATGGTACTAGCGCTCATTGCGCCGACAGCAATGTCTGCGTGTCGAAGGCACGTCGGCATGTGTAGT---ACAACGC

CTTGTGCACTGGTGAA-CGGCTATTTTC

>Hap_27

TCGGTCGAGCGGTGC------CCGCCGCAGCAAGGCGGGCGGTA------TCGTCGTCGTCGACTGCT------------

---GCTGCCGTGCAACGACGTCGAACACTTGCTG---CGAGCGCGTGCGGTCACCGCTCGGTGCTCAACTGTCGATTCGC

GCTGCTGTACGCCGACGGCGCGGCCAGTAGCCGAGTCGGGTAGTCTTCGCACTACGCTGACTTGGCCGCTCTCCGCTGCT

CCGGCTAGCAGTTGCCATCGCGGCTCGCTGGCAACGGAGCTGCGGCGTCACGCTCGCTCA----CGGGTGCAGGTTTGTC

CAGTGCTCACCACCAACCTCAACCTGTAGCCTGAGCGGCGCATTCGTTATT--GCTTAACGACCCTCAGACAGGCGTAGC

GTCGACATCTTTGATCGACGCTGCAATGTGCGTTCAAAGTATTCAAGTTCGGTGTGTCTGCAATTCACGTCAATTCTCGA

GTGTCACGTCGTTCTTCAACGATCTACGAGCCAAGTGATCCACCGTTCGGAATGTACTTTCCTGCGCGTA---CAGCAGT

TCGAATGGTACTAGCGCTCATTGCGCCGACAGCAATGTCTGCGTGTCGAAGGCACGTCGGCATGTGTAGT---ACAACGC

CTTGTGCACTGGTGAA-CGGCTATTTTC

>Hap_28

TCGGTCGAGCGGTGC------CCGCCGCAGCAAGGCGGGCGGTA------TCGTCGTCGTCGACTGCT------------

---GCTGCCGTGCAACGACGTCGAACACTTGCTG---CGAGCGCGTGCGGTCACCGCTCGGTGCTCAACTGTCGATTCGC

GCTGCTGTACGCCGACGGCGCGGCCAGTAGCCGAGTCGGGTAGTCTTCGCACTACGCTGACTTGGCCGCTCTCCGCTGCT

CCGGCTAGCAGTTGCCATCGCGGCTCGCTGGCAACGGAGCTGCGGCGTCACGCTCGCTCG----CGGGTGCAGGTTTGTC

CAGTGCTCACCACCAACCTCAACCTGTAGCCTGAGCGGCGCATTCGCTATT--GCTTAACGACCCTCAGACAGGCGTAGC

GTCGACATCTTTGATCGACGCTGCAATGTGCGTTCAAAGTATTCAAGTTCGGTGTGTCTGCAATTCACGTCAATTCTCGA

GTGTCACGTCGTTCTTCAACGATCTACGAGCCAAGTGATCCACCGTTCGGAATGTACTTTCCTGCGCGTA---CAGCAGT

TTGAATGGTACTAGCGCTCATTGCGCCGACAGCAATGTCTGCGTGTCGAAGGCACGTCGGCATGTGTAGT---ACAACGC

CTTGTGCACTGGTGAA-CGGCTATTTTC

>Hap_29

TCGGTCGAGCGGTGC------CCGCCGCAGCAAGGCGGGCGGTA------TCGTCGTCGTCGACTGCT------------

---GCTGCCGTGCAACGACGTCGAACACTTGCTG---CGAGCGCGTGCGGTCACCGCTCGGTGCTCAACTGTCGATTTGC

GCTGCTGTACGCCGACGGCGCGGCCAGTAGCCGAGTCGGGTAGTCTTCGCACTACGCTGACTTGGCCGCTCTCCGCTGCT

CCGGCTAGCAGTTGCCATCGCGGCTCGCTGGCAACGGAGCTGCGGCGTCACGCTCGCTCG----CGGATGCAGGTTTGTC

CAGTGCTCGCCACCAACCTCAACCTGTAGCCTGAGCGGCGCATTCGCTATT--GCTTAACGACCCTCAGACAGGCGTAGC

GTCGACATCTTTGATCGACGCTGCAATGTGCGTTCAAAGTATTCAAGTTCGGTGTGTCTGCAATTCACGTCAATTCTCGA

GTGTCACGTCGTTCTTCAACGATCTACGAGCCAAGTGATCCACCGTTCGGAATGTACTTTCCTGCGCGTA---CAGCAGT

TCGAATGGTACTAGCGCTCATTGCGCCGACAGCAATGTCTGCGTGTCGAACGCACGTCGGCATGTGTAGT---ACAACGC

CTTGTGCACTGATGAA-CGGCTATTTTC

>Hap_30

TCGGTCGAGCGGTGC------CCGCCGCAGCAAGGCGGGCGGTA------TCGTCGTCGTCGACTGCT------------

---GCTGCCGTGCAACGACGTCGAACACTTGCTG---CGAGCGCGTGCGGTCACCGCTCGGTGCTCAACTGTCGATTCGC

GCTGCTGTACGCCGACGGCGCGGCCAGTAGCCGAGTCGGGTAGTCTTCGCACTACGCTGACTTGGCCGCTCTCCGCTGCT

CCGGCTAGCAGTTGCCATCGCGGCTCGCTGGCAACGGAGCTGCGGCGTCACGCTCGCTCG----CGGGTGCAGGTTTGTC

CAGTGCTCGCCACCAACCTCAACCTGTAGCCTGAGCGGCGCATTCGCTATT--GCTTAACGACCCTCAGACAGGCGTAGC

GTCGACATCTTTGATCGACGCTGCAATGTGCGTTCAAAGTATTCAAGTTCGGTGTGTCTGCAATTCACGTCAATTCTCGA

GTGTCACGTCGTTCTTCAACGATCTACGAGCCAAGTGATCCACCTTTCGGAATGTACTTTCCTGCGCGTA---CAGCAGT

TCGAATGGTACTAGCGCTCATTGCGCCTACAGCAATGTCTGCGTGTCGAAGGCACGTCGGCATGTGTAGT---ACAACGC

CTTGTGCACTGATGA-GCGGCTATTTTC

>Hap_31

TCGGTCGAGCGGTGC------CCGCCGCAGCAAGGCGGGCGGTA------TCGTCGTCGTCGACTGCT------------

---GCTGCCGTGCAACGACGTCGAACACTTGCTG---CGAGCGCGTGCGGTCACCGCTCGGTGCTCAACTGTCGATTTGC

GCTGCTGTACGCCGACGGCGCGGCCAGTAGCCGAGTCGGGTAGTCTTCGCACTACGCTGACTTGGCCGCTCTCCGCTGCT

CCGGCTAGCAGTTGCCATCGCGGCTCGCTGGCAACGGAGCTGCGGCGTCACGCTCGCTCG----CGGATGCAGGTTTGTC

CAGTGCTCGCCACCAACCTCAACCTGTAGCCTGAGCGGCGCATTCGCTATT--GCTTAACGACCCTCAGACAGGCGTAGC

GTCGACATCTTTGATCGACGCTGCAATGTGCGTTCAAAGTATTCAAGTTCGGTGTGTCTGCAATTCACGTCAATTCTCGA

GTGTCACGTCGTTCTTCAACGATCTACGAGCCAAGTGATCCACCGTTCGGAATGTACTTTCCTGCGCGTA---CAGCAGT

TCGAATGGTACTAGCGCTCATTGCGCCGACAGCAATGTCTGCGTGTCGAAAGCACGTCGGCATGTGTAGT---ACAACGC

CTTGTGCACTGATGAA-CGGCTATTTTC

>Hap_32

TCGGTCGAGCGGTGC------CCGCCGCAGCAAGGCGGGCGGTA------TCGTCGTCGTCGACTGCT------------

---GCTGCCGTGCAACGACGTCGAACCCTTGGTG---CCAGCGCGTGCGGTCACCGCTCGGTGCTCAACTGTCGATTCGC

GCTGCTTTACGCCGACGGCGGGGCCAGTAGCCGAGTCGGGTAGTCTTCGCACTACGCTGACTTGGCCGCTCTCCGCTGCT

CCGGCTAGCAGTTGCCATCGCGGCTCGCTGGCAACGGAGCTGCGGCGTCACGCTCGCTCA----CGGGTGCAGGTTTGTC

CAGTGCTCGCCACCAACCTCAACCTGTAGCCTGAGCGGCGCATTCGCTATT--GCTTAACGACCCTCAGACAGGCGTAGC

GTCGACATCTTTGATCGACGCTGCAATGTGCGTTCAAAGTATTCAAGTTCGGTGTGTCTGCAATTCACGTCAATTCTCGA

GTGTCACGTCGTTCTTCAACGATCTACGAGCCAAGTGATCCACCGTTCGGAATGTACTTTCCTGCGCGTA---CAGCAGT

TCGAATGGTACTAGCGCTCATTGCGCCGACAGCAATGTCTGCGTGTCGAAGGCACGTCGGCATGTGTAGT---ACAACGC

CTTGTGCACTGGTGAA-CGGCTATTTTC

>Hap_33

TCGGTCGAGCGGTGC------CCGCCGCAGCAAGGCGGGCGGTA------TCGTCGTCGTCGACTGCT------------

---GCTGCCGTGCAACGACGTCGAACACTTGCTG---CGAGCGCGTGCGGTCACCGCTCGGTGCTCAACTGTCGATTCGC

GCTGCTGTACGCCGACGGCGCGGCCAGTAGCCGAGTCGGGTAGTCTTCGCACTACGCTGACTTGGCCGCTCTCCGCTGCT

CCGGCTAGCAGTTGCCATCGCGGCTCGCTGGCAACGGAGCTGCGGCGTCACGCTCGCTCG----CGGGTGCAGGTTTGTC

CAGTGCTCGCCACCAACCTCAACCTGTAGCCTGAGCGGCGCATTCGCTATT--GCTTAACGACCCTCAGACAGGCGTAGC

GTCGACATCTTTGATCGACGCTGCAATGTGCGTTCAAAGTATTCAAGTTCGGTGTGTCTGCCATTAAAGTCAATTCTGGA

ATGTCACATCGTTCTTCAACTATCTACGAGCCAAGTGATCCACCGTTCGGAATGTACTTTCCTGCGCGTA---CAGCAGT

TCGAATGGTACTAGCGCTCATTGCGCCGACAGCAATGTCTGCGTGTCGAAGGCACGTCGGCATGTGTAGT---ACAACGC

CTTGTGCACTGGTGAA-CGGCTATTTTC

>Hap_34

TCGGTCGAGCGGTGCC---CGCCGCCGCAGCAAGGCGAACGGTTTCG---TCGTCGTCGTCGACTGCTGCT---GCCGCT

GCTGCTGCCGTGCAACTACGACGAACACTTGCTGCTACGAGCGCGTGCGGTCACCGCTCCCTGCTCAA------------

-CTGCTGTACGCCGACGGCGCGGCCGGTAGCCAAGTCGGGTAGTCTTCGCCCTACGCTGACTTGGCCGCTCTCCGCTGCT

CCGCCTACCAGTTGCCATCGCGGCTCGCTGGCAACGGAGCTGCGGCGTCACGCTCGCTCGCGCGCGGATGCAGGTTTGTC

CGGTGCTCGCCACCAACCTCAACCTGTAGCCTGAGCGGCGCATTCGCTATTATGCTTAACGACCCTCAGACAGGCGTAGC

GTCGACATCTTTGATCGACGCTGCAATGTGCGTTCAAAGTATTCAAGTTCGGTGTGTCTGCAATTCACGTCAATTCTCGA

GTGTCACGTCGTTCTTCAACGATCTACGAGCCAAGTGATCCACCGTTCGGAATGTACTTTCCTGCGCGTACAGCAGCAGT

TCGAATGCTACTAGCGCTCATTGCGCCGACAGCAGTGTCTGCGTGTCGAAGGCACATCGGCGTGCGTAGTAGGACGACGC

CTTGTGCACTGGTGAAGCTGCT-TTTTC

>Hap_35

TCGGTCGAGCGGTGCC---CGCCGCCGCAGCAAGGCGAACGGTTTCGTCGTCGTCGTCGTCGACTGCTGCT------GCC

GCTGCTGCCGTGCAACGACGACGAACACTTGCTGCTACGAGCGCGTGCGATCACCGCTCCCTGCTCAA------------

-CTGCTGTACGCCGACGGCGCGGCCGGTAGCCAAGTCGGGTAGTCTTCGCCCTACGCTGACTTGGCCGCTCTCCGCTGCT

CCGCCTACCAGTTGCCATCGCGGCTCGCTGGCAACGGAGCTGCGGCGTCACGCTCGCTCGCGCGCGGATGCAGGTTTGTC

CGGTGCTCGCCACCAACCTCAACCTGTAGCCTGAGCGGCGCATTCGCTATTATGCTTAACGACCCTCAGACAGGCGTAGC

GTCGACATCTTTGATCGACGCTGCAATGTGCGTTCAAAGTATTCAAGTTCGGTGTGTCTGCAATTCACGTCAATTCTCGA

GTGTCACGTCGTTCTTCAACGATCTACGAGCCAAGTGATCCACCGTTCGGAATGTACTTTCCTGCGCGTACAGCAGCAGT

TCGAATGCTACTAGCGCTCATTGCGCCGACAGCAGTGTCTGCGTGTCGAAGGCACATCGGCGTGCGTAGTAGGACGACGC

CTTGTGCACTGGTGAAGCTGCT-TTTTC
