# Supplementary material for: Molecular and morphologic characterization of Trichuris trichiura infecting free-roaming African vervets (Chlorocebus aethiops sabaeus) on the Caribbean Island of St. Kitts
Source: PLoS Negl Trop Dis. 2026 Jul 16;20(7):e0014539. doi: 10.1371/journal.pntd.0014539 (PMC13405091; doi:10.1371/journal.pntd.0014539)
Supplement: S3 File — A phylogenetic tree identical to the tree shown in Fig 1, though with specimen labels provided on branch tips. (PDF) [file pntd.0014539.s003.pdf]

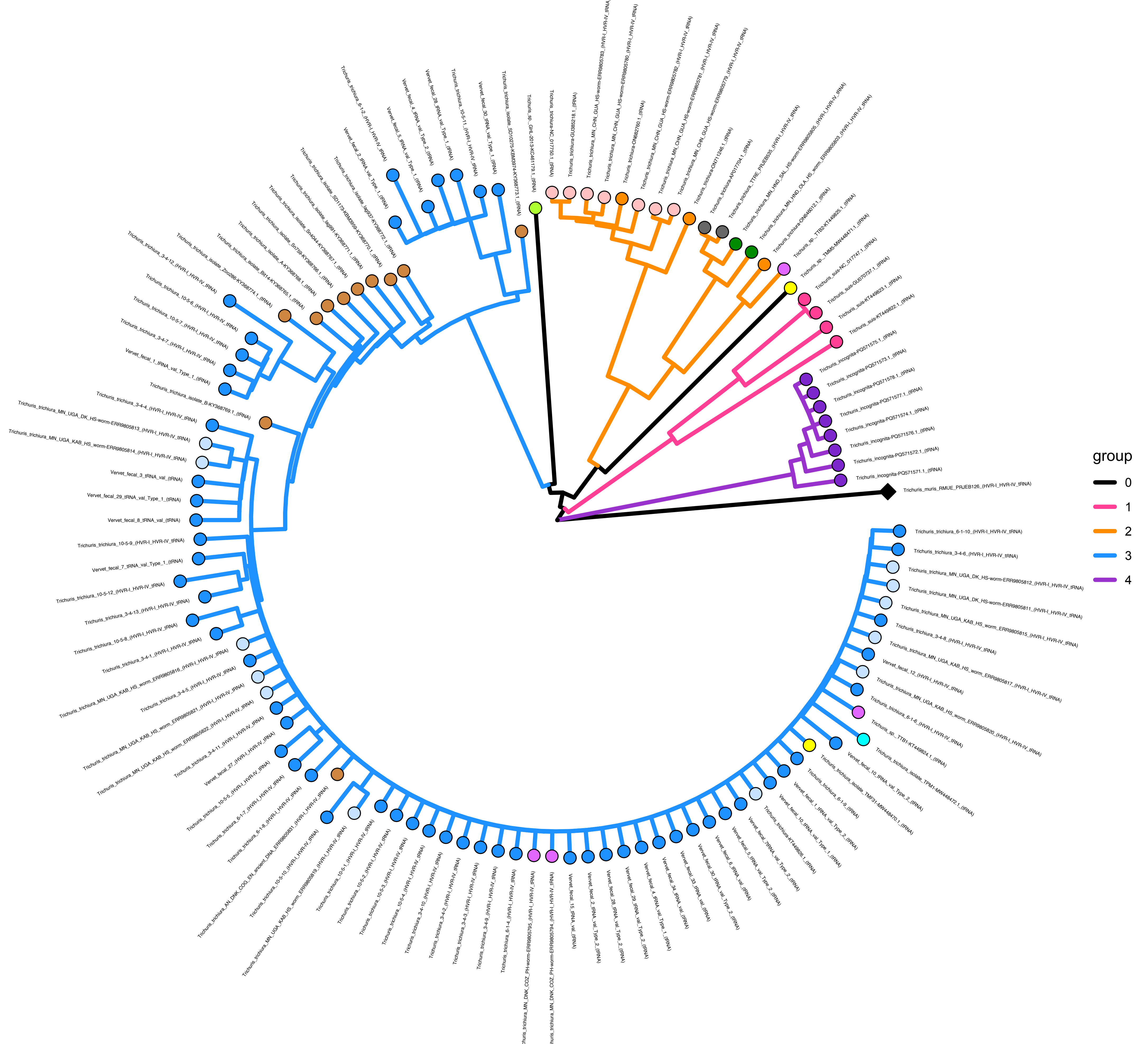
group
0
1
2
3
4
