# Supplementary material for: Molecular and morphologic characterization of Trichuris trichiura infecting free-roaming African vervets (Chlorocebus aethiops sabaeus) on the Caribbean Island of St. Kitts
Source: PLoS Negl Trop Dis. 2026 Jul 16;20(7):e0014539. doi: 10.1371/journal.pntd.0014539 (PMC13405091; doi:10.1371/journal.pntd.0014539)
Supplement: Attachment — Submitted filename: Reviewer_responses.docx [file pntd.0014539.s004.docx]

**Reviewers' Comments:**

**Reviewer #1:**

**Comment 1: Study design clearly outlined. Sample size seems appropriate. Methods clearly described. No concerns about ethical or regulatory requirements being met.**

**Our response: Thank you for your kind support of our work.
-------------------------------------------------------------------------------------**

**Comment 2: Figure 1 should be labelled in more detail - specifically, arrows should be added to indicate the features being described as it is not clear from the description for the non-expert reader.**

**Our response: We have added additional labels to the figure and updated the figure legend accordingly.
-------------------------------------------------------------------------------------
Comment 3: For a non-geneticist, the main part of the results section is hard to follow. Would suggest more word count afforded to make this accessible for general parasitologists - for example what is the significance of the different clades?**

**Comment 5: In general, this article is difficult to follow for non-geneticists. Would recommend some additional text in the results and discussion to describe/discuss the key findings in simple terms. For example, a gentle recap of the findings and their potential impact at the start of the discussion - perhaps move some of the text from the second paragraph into the first. Closing paragraph is strong and provides an excellent summary for non-experts.**

**Our response: Comments 3 and 5 raise a similar concern, and we have chosen to address both of these together. Response:**

**We apologize that this is confusing. The clades 2a through 2d were first described in a separate paper (see the following:** [**https://pmc.ncbi.nlm.nih.gov/articles/PMC7934208/**](https://pmc.ncbi.nlm.nih.gov/articles/PMC7934208/)**). This was reference 5 in our original submission.**

**As you know, our main objective was to ascertain if Trichuris trichiura from St Kitts vervets is of a type that infects humans (i.e., which can help assess the risk of zoonotic transmission) – which we achieved. This is because T. trichiura from vervets was assigned to the same clade as other T. trichiura samples of human origin.**

**However, we are also not certain of the significance of the various clades. Usually, the emergence of distinct clades is driven by a biological difference (e.g., adaptation to certain hosts), or vicariance (geographic separation over long periods). Figure 2 shows us that there is no clear relationship between host and clade (I.e., we cannot say that one clade prefers host X over host Y). We also see that the different clades are widely dispersed geographically, so this data does not tell us much about whether vicariance was a driver of their separation… It is possible that these various clades were distributed very widely due to human activities (i.e., ancient human movements), but we can never know for sure. Consequently, we know that these clades are supported genetically, but as to their significance, the present results do not provide sufficient insight to comment on this definitively.**

**To address this comment, we have added an additional paragraph to the discussion that speculates on the significance of the clades. We felt this belonged in the discussion rather than the results. This is because the significance of the clades is uncertain, so there is a great deal of conjecture woven in, which may not fit well in the Results. However, this paragraph does seek to summarize the take aways of the genetics to assist non-geneticists in extracting meaning from the paper.**

**-------------------------------------------------------------------------------------**

**Comment 4: Conclusions are supported by the data presented and limitations of study are described. Public health relevance clear - vervet monkeys could be a zoonotic source of trichuriasis infection on St Kitts.**

**Our response: Thank you for your kind support of our work.**

**-------------------------------------------------------------------------------------**

**Reviewer 2:**

**Minor 1: This study represents an interesting addition to the current body of literature investigating taxonomy and population structure within the Trichuris genus. The St. Kitts vervet population is particularly relevant given its close ecological overlap with humans and the resulting implications for zoonotic transmission. The integration of public genomic datasets is a strength of the manuscript and allows for meaningful phylogenetic contextualization.**

**Our response: Thank you for your kind appraisal of our work.**

**-------------------------------------------------------------------------------------
Major comments:
C1. Incomplete amplification and marker choice. A substantial proportion of fecal samples (17/34) did not yield sequence data, despite microscopy confirming the presence of Trichuris eggs. While incomplete amplification is expected in fecal-derived DNA, this raises concerns about potential underrepresentation of diversity within this population. I recommend that the authors consider testing additional primer pairs to reduce the risk of missing divergent Trichuris lineages. It has previously been demonstrated that primers designed for T. trichiura may fail to amplify other species, including T. incognita (**[**https://doi.org/10.3201/eid3101.240995**](https://nam11.safelinks.protection.outlook.com/?url=https%3A%2F%2Fdoi.org%2F10.3201%2Feid3101.240995&data=05%7C02%7Cjoel.barratt%40emory.edu%7Cd3a247569eba4dcafe3d08de803b7a13%7Ce004fb9cb0a4424fbcd0322606d5df38%7C0%7C0%7C639089191114193018%7CUnknown%7CTWFpbGZsb3d8eyJFbXB0eU1hcGkiOnRydWUsIlYiOiIwLjAuMDAwMCIsIlAiOiJXaW4zMiIsIkFOIjoiTWFpbCIsIldUIjoyfQ%3D%3D%7C0%7C%7C%7C&sdata=Ot35zRYlTfIxr0aFrbBiBqnqn2lrNFgXEU3PxojvxXc%3D&reserved=0)**). Expanding primer coverage would help ensure that non-trichiura taxa are not overlooked. Furthermore, sequencing of a full-length ITS2 fragment (ideally with a strategy enabling haplotype resolution) would help minimize reconstruction bias and provide an independent nuclear marker for phylogenetic comparison. A strengthened multi-locus approach would considerably reinforce the phylogenetic conclusions presented.**

**Our response: We agree with this statement. Unfortunately, budgetary constraints do not allow us to generate more sequence data. However, we have added a paragraph to the discussion mentioning that failure to amplify a PCR product in some cases could be related to the fact that the primer design may have excluded certain Trichuris populations.**

**-------------------------------------------------------------------------------------**

**C2. Exclusion of ITS1/ITS2 data. The justification for excluding ITS1 and ITS2 data is not fully convincing. While phasing multi-copy rDNA loci from short-read data can be challenging, this does not preclude analysis of ITS diversity. Amplicon-based ITS analyses in Trichuris, including cases with intra-individual heterogeneity, have been successfully conducted using short-read sequencing (e.g.,** [**https://doi.org/10.3201/eid3101.240995**](https://nam11.safelinks.protection.outlook.com/?url=https%3A%2F%2Fdoi.org%2F10.3201%2Feid3101.240995&data=05%7C02%7Cjoel.barratt%40emory.edu%7Cd3a247569eba4dcafe3d08de803b7a13%7Ce004fb9cb0a4424fbcd0322606d5df38%7C0%7C0%7C639089191114242415%7CUnknown%7CTWFpbGZsb3d8eyJFbXB0eU1hcGkiOnRydWUsIlYiOiIwLjAuMDAwMCIsIlAiOiJXaW4zMiIsIkFOIjoiTWFpbCIsIldUIjoyfQ%3D%3D%7C0%7C%7C%7C&sdata=P36HmBdPxYsNB6IoaimOAtrodUvXafY2cKQbQnkHfA4%3D&reserved=0)**). Even if full haplotype phasing at the individual-worm level is not feasible, the authors should at least present the detected ITS1/ITS2 sequence variants and their relative abundances per sample (e.g., as ASVs or clustered variants in supplementary material). Completely omitting these data despite successful sequencing leaves an important component of the dataset unexplored and reduces the overall completeness of the study.**

**Our response: We agree with the suggestion that we could do more with the ITS data. We were able to extract some well supported ASVs from this ITS1 and ITS2 data using DADA2. We used these sequences to generate haplotype maps. We thank the reviewer for this suggestion as it does add value to the final product. Note that we have uploaded the ITS1 and ITS2 sequences to GenBank but we are waiting on accession numbers. When accession numbers are assigned we will add these to the paper (i.e., during the proof stage if the manuscript is accepted).**

**-------------------------------------------------------------------------------------**

**Minor comment:
C3 – Taxonomic status of T. incognita. Line: “noting that the name ‘incognita’ was assigned without a formal taxonomic description and likely represents a nomen nudum [23]”
A formal taxonomic description has now been published (**[**https://doi.org/10.1016/j.lanmic.2025.101264**](https://nam11.safelinks.protection.outlook.com/?url=https%3A%2F%2Fdoi.org%2F10.1016%2Fj.lanmic.2025.101264&data=05%7C02%7Cjoel.barratt%40emory.edu%7Cd3a247569eba4dcafe3d08de803b7a13%7Ce004fb9cb0a4424fbcd0322606d5df38%7C0%7C0%7C639089191114284047%7CUnknown%7CTWFpbGZsb3d8eyJFbXB0eU1hcGkiOnRydWUsIlYiOiIwLjAuMDAwMCIsIlAiOiJXaW4zMiIsIkFOIjoiTWFpbCIsIldUIjoyfQ%3D%3D%7C0%7C%7C%7C&sdata=epxh9ozhiEVexStVwW8peZQtFPDpz%2BvUOrlDykAI9M4%3D&reserved=0)**), with further contextual characterization in** [**https://doi.org/10.3201/eid3101.240995**](https://nam11.safelinks.protection.outlook.com/?url=https%3A%2F%2Fdoi.org%2F10.3201%2Feid3101.240995&data=05%7C02%7Cjoel.barratt%40emory.edu%7Cd3a247569eba4dcafe3d08de803b7a13%7Ce004fb9cb0a4424fbcd0322606d5df38%7C0%7C0%7C639089191114323494%7CUnknown%7CTWFpbGZsb3d8eyJFbXB0eU1hcGkiOnRydWUsIlYiOiIwLjAuMDAwMCIsIlAiOiJXaW4zMiIsIkFOIjoiTWFpbCIsIldUIjoyfQ%3D%3D%7C0%7C%7C%7C&sdata=SEU8DddCojckVPrVyjcef0KM2WjJR0SnEocHfvldIL0%3D&reserved=0)**. The manuscript should be updated accordingly to reflect the current taxonomic status.**

**Our response: Apologies for this oversight. We have corrected Figure 2 (cognita is no longer in quotes) and we now cite the species description in the manuscript. Note that in the original submission of this article, the EID publication you mention here (**[**https://doi.org/10.3201/eid3101.240995**](https://nam11.safelinks.protection.outlook.com/?url=https%3A%2F%2Fdoi.org%2F10.3201%2Feid3101.240995&data=05%7C02%7Cjoel.barratt%40emory.edu%7Cd3a247569eba4dcafe3d08de803b7a13%7Ce004fb9cb0a4424fbcd0322606d5df38%7C0%7C0%7C639089191114323494%7CUnknown%7CTWFpbGZsb3d8eyJFbXB0eU1hcGkiOnRydWUsIlYiOiIwLjAuMDAwMCIsIlAiOiJXaW4zMiIsIkFOIjoiTWFpbCIsIldUIjoyfQ%3D%3D%7C0%7C%7C%7C&sdata=SEU8DddCojckVPrVyjcef0KM2WjJR0SnEocHfvldIL0%3D&reserved=0)**) was reference 23.**

**-------------------------------------------------------------------------------------**
