# Supplementary material for: Molecular and morphologic characterization of Trichuris trichiura infecting free-roaming African vervets (Chlorocebus aethiops sabaeus) on the Caribbean Island of St. Kitts
Source: PLoS Negl Trop Dis. 2026 Jul 16;20(7):e0014539. doi: 10.1371/journal.pntd.0014539 (PMC13405091; doi:10.1371/journal.pntd.0014539)
Supplement: Peer review history

# Peer review: Decision Letter 0

**DOI:** 10.1371/journal.pntd.0014539.r001

**Authors:** Maria Victoria Periago, Bruce A. Rosa

23 Feb 2026

-->PNTD-D-26-00096

Molecular and morphologic characterization of Trichuris trichiura infecting free-roaming African vervets (Chlorocebus aethiops sabaeus) on the Caribbean Island of St. Kitts

PLOS Neglected Tropical Diseases

Dear Dr. Richins,

Thank you for submitting your manuscript to PLOS Neglected Tropical Diseases. After careful consideration, we feel that it has merit but does not fully meet PLOS Neglected Tropical Diseases's publication criteria as it currently stands. Therefore, we invite you to submit a revised version of the manuscript that addresses the points raised during the review process.

Please submit your revised manuscript within by Apr 24 2026 11:59PM. If you will need more time than this to complete your revisions, please reply to this message or contact the journal office at plosntds@plos.org. When you're ready to submit your revision, log on to https://www.editorialmanager.com/pntd/ and select the 'Submissions Needing Revision' folder to locate your manuscript file.

Please include the following items when submitting your revised manuscript:

* A letter that responds to each point raised by the editor and reviewer(s). You should upload this letter as a separate file labeled 'Response to Reviewers'. This file does not need to include responses to any formatting updates and technical items listed in the 'Journal Requirements' section below.

* A marked-up copy of your manuscript that highlights changes made to the original version. You should upload this as a separate file labeled 'Revised Manuscript with Track Changes'.

* An unmarked version of your revised paper without tracked changes. You should upload this as a separate file labeled 'Manuscript'.

If you would like to make changes to your financial disclosure, competing interests statement, or data availability statement, please make these updates within the submission form at the time of resubmission. Guidelines for resubmitting your figure files are available below the reviewer comments at the end of this letter.

We look forward to receiving your revised manuscript.

Kind regards,

Bruce A. Rosa

Academic Editor

PLOS Neglected Tropical Diseases

María Periago

Section Editor

PLOS Neglected Tropical Diseases

Shaden Kamhawi

co-Editor-in-Chief

PLOS Neglected Tropical Diseases

orcid.org/0000-0003-4304-636XX

Paul Brindley

co-Editor-in-Chief

PLOS Neglected Tropical Diseases

orcid.org/0000-0003-1765-0002

**Additional Editor Comments:**

Please carefully consider the reviewer feedback and revise the manuscript accordingly.

**Journal Requirements:**

If the reviewer comments include a recommendation to cite specific previously published works, please review and evaluate these publications to determine whether they are relevant and should be cited. There is no requirement to cite these works unless the editor has indicated otherwise.

1) Please upload all main figures as separate Figure files in .tif or .eps format. For more information about how to convert and format your figure files please see our guidelines:

https://journals.plos.org/plosntds/s/figures

2) Some material included in your submission may be copyrighted. According to PLOSu2019s copyright policy, authors who use figures or other material (e.g., graphics, clipart, maps) from another author or copyright holder must demonstrate or obtain permission to publish this material under the Creative Commons Attribution 4.0 International (CC BY 4.0) License used by PLOS journals. Please closely review the details of PLOSu2019s copyright requirements here: PLOS Licenses and Copyright. If you need to request permissions from a copyright holder, you may use PLOS's Copyright Content Permission form.

Please respond directly to this email and provide any known details concerning your material's license terms and permissions required for reuse, even if you have not yet obtained copyright permissions or are unsure of your material's copyright compatibility. Once you have responded and addressed all other outstanding technical requirements, you may resubmit your manuscript within Editorial Manager.

Potential Copyright Issues:

i) Please confirm (a) that you are the photographer of 1, or (b) provide written permission from the photographer to publish the photo(s) under our CC BY 4.0 license.

ii) Figure 2. Please confirm whether you drew the images / clip-art within the figure panels by hand. If you did not draw the images, please provide (a) a link to the source of the images or icons and their license / terms of use; or (b) written permission from the copyright holder to publish the images or icons under our CC BY 4.0 license. Alternatively, you may replace the images with open source alternatives. See these open source resources you may use to replace images / clip-art:

- https://commons.wikimedia.org

- https://openclipart.org/.

iii) Figure 2. Please (a) provide a direct link to the base layer of the map (i.e., the country or region border shape) and ensure this is also included in the figure legend; and (b) provide a link to the terms of use / license information for the base layer image or shapefile. We cannot publish proprietary or copyrighted maps (e.g. Google Maps, Mapquest) and the terms of use for your map base layer must be compatible with our CC BY 4.0 license.

Note: if you created the map in a software program like R or ArcGIS, please locate and indicate the source of the basemap shapefile onto which data has been plotted.

If your map was obtained from a copyrighted source please amend the figure so that the base map used is from an openly available source. Alternatively, please provide explicit written permission from the copyright holder granting you the right to publish the material under our CC BY 4.0 license.

If you are unsure whether you can use a map or not, please do reach out and we will be able to help you. The following websites are good examples of where you can source open access or public domain maps:

* U.S. Geological Survey (USGS) - All maps are in the public domain. (http://www.usgs.gov)

* PlaniGlobe - All maps are published under a Creative Commons license so please cite u201cPlaniGlobe, http://www.planiglobe.com, CC BY 2.0u201d in the image credit after the caption. (http://www.planiglobe.com/?lang=enl)

* Natural Earth - All maps are public domain. (http://www.naturalearthdata.com/about/terms-of-use/).

3) In the online submission form, you indicated that your data will be submitted to a repository upon acceptance. We strongly recommend all authors deposit their data before acceptance, as the process can be lengthy and hold up publication timelines. Please note that, though access restrictions are acceptable now, your entire minimal dataset will need to be made freely accessible if your manuscript is accepted for publication. This policy applies to all data except where public deposition would breach compliance with the protocol approved by your research ethics board. If you are unable to adhere to our open data policy, please kindly revise your statement to explain your reasoning and we will seek the editor's input on an exemption.

**Reviewers' Comments:**

Reviewer's Responses to Questions

**Key Review Criteria Required for Acceptance?**

As you describe the new analyses required for acceptance, please consider the following:

**Methods**

-Are the objectives of the study clearly articulated with a clear testable hypothesis stated?

-Is the study design appropriate to address the stated objectives?

-Is the population clearly described and appropriate for the hypothesis being tested?

-Is the sample size sufficient to ensure adequate power to address the hypothesis being tested?

-Were correct statistical analysis used to support conclusions?

-Are there concerns about ethical or regulatory requirements being met?

Reviewer #1: Study design clearly outlined. Sample size seems appropriate. Methods clearly described. No concerns about ethical or regulatory requirements being met.

Reviewer #2: (No Response)

**********

**Results**

-Does the analysis presented match the analysis plan?

-Are the results clearly and completely presented?

-Are the figures (Tables, Images) of sufficient quality for clarity?

Reviewer #1: Figure 1 should be labelled in more detail - specifically, arrows should be added to indicate the features being described as it is not clear from the description for the non-expert reader.

For a non-geneticist, the main part of the results section is hard to follow. Would suggest more word count afforded to make this accessible for general parasitologists - for example what is the significance of the different clades?

Reviewer #2: (No Response)

**********

**Conclusions**

-Are the conclusions supported by the data presented?

-Are the limitations of analysis clearly described?

-Do the authors discuss how these data can be helpful to advance our understanding of the topic under study?

-Is public health relevance addressed?

Reviewer #1: Conclusions are supported by the data presented and limitations of study are described. Public health relevance clear - vervet monkeys could be a zoonotic source of trichuriasis infection on St Kitts.

Reviewer #2: (No Response)

**********

**Editorial and Data Presentation Modifications?**

Use this section for editorial suggestions as well as relatively minor modifications of existing data that would enhance clarity. If the only modifications needed are minor and/or editorial, you may wish to recommend “Minor Revision” or “Accept”.

Reviewer #1: See comment in results section regarding adding labels to figure 1 to enhance clarity.

Reviewer #2: (No Response)

**********

**Summary and General Comments**

Use this section to provide overall comments, discuss strengths/weaknesses of the study, novelty, significance, general execution and scholarship. You may also include additional comments for the author, including concerns about dual publication, research ethics, or publication ethics. If requesting major revision, please articulate the new experiments that are needed.

Reviewer #1: In general, this article is difficult to follow for non-geneticists. Would recommend some additional text in the results and discussion to describe/discuss the key findings in simple terms. For example, a gentle recap of the findings and their potential impact at the start of the discussion - perhaps move some of the text from the second paragraph into the first. Closing paragraph is strong and provides an excellent summary for non-experts.

Reviewer #2: This study represents an interesting addition to the current body of literature investigating taxonomy and population structure within the Trichuris genus. The St. Kitts vervet population is particularly relevant given its close ecological overlap with humans and the resulting implications for zoonotic transmission. The integration of public genomic datasets is a strength of the manuscript and allows for meaningful phylogenetic contextualization.

Major comments:

C1. Incomplete amplification and marker choice. A substantial proportion of fecal samples (17/34) did not yield sequence data, despite microscopy confirming the presence of Trichuris eggs. While incomplete amplification is expected in fecal-derived DNA, this raises concerns about potential underrepresentation of diversity within this population. I recommend that the authors consider testing additional primer pairs to reduce the risk of missing divergent Trichuris lineages. It has previously been demonstrated that primers designed for T. trichiura may fail to amplify other species, including T. incognita (https://doi.org/10.3201/eid3101.240995). Expanding primer coverage would help ensure that non-trichiura taxa are not overlooked. Furthermore, sequencing of a full-length ITS2 fragment (ideally with a strategy enabling haplotype resolution) would help minimize reconstruction bias and provide an independent nuclear marker for phylogenetic comparison. A strengthened multi-locus approach would considerably reinforce the phylogenetic conclusions presented.

C2. Exclusion of ITS1/ITS2 data. The justification for excluding ITS1 and ITS2 data is not fully convincing. While phasing multi-copy rDNA loci from short-read data can be challenging, this does not preclude analysis of ITS diversity. Amplicon-based ITS analyses in Trichuris, including cases with intra-individual heterogeneity, have been successfully conducted using short-read sequencing (e.g., https://doi.org/10.3201/eid3101.240995). Even if full haplotype phasing at the individual-worm level is not feasible, the authors should at least present the detected ITS1/ITS2 sequence variants and their relative abundances per sample (e.g., as ASVs or clustered variants in supplementary material). Completely omitting these data despite successful sequencing leaves an important component of the dataset unexplored and reduces the overall completeness of the study.

Minor comment:

C1 – Taxonomic status of T. incognita. Line: “noting that the name ‘incognita’ was assigned without a formal taxonomic description and likely represents a nomen nudum [23]”

A formal taxonomic description has now been published (https://doi.org/10.1016/j.lanmic.2025.101264), with further contextual characterization in https://doi.org/10.3201/eid3101.240995. The manuscript should be updated accordingly to reflect the current taxonomic status.

**********

PLOS authors have the option to publish the peer review history of their article (what does this mean?). If published, this will include your full peer review and any attached files.

If you choose “no”, your identity will remain anonymous but your review may still be made public.

**Do you want your identity to be public for this peer review?**  For information about this choice, including consent withdrawal, please see our Privacy Policy.

Reviewer #1: No

Reviewer #2: No

**Figure resubmission:**

While revising your submission, we strongly recommend that you use PLOS’s NAAS tool (https://ngplosjournals.pagemajik.ai/artanalysis) to test your figure files. NAAS can convert your figure files to the TIFF file type and meet basic requirements (such as print size, resolution), or provide you with a report on issues that do not meet our requirements and that NAAS cannot fix.

-->

After uploading your figures to PLOS’s NAAS tool - https://ngplosjournals.pagemajik.ai/artanalysis, NAAS will process the files provided and display the results in the "Uploaded Files" section of the page as the processing is complete. If the uploaded figures meet our requirements (or NAAS is able to fix the files to meet our requirements), the figure will be marked as "fixed" above. If NAAS is unable to fix the files, a red "failed" label will appear above. When NAAS has confirmed that the figure files meet our requirements, please download the file via the download option, and include these NAAS processed figure files when submitting your revised manuscript.-->

**Reproducibility:**

To enhance the reproducibility of your results, we recommend that authors of applicable studies deposit laboratory protocols in protocols.io, where a protocol can be assigned its own identifier (DOI) such that it can be cited independently in the future. Additionally, PLOS ONE offers an option to publish peer-reviewed clinical study protocols. Read more information on sharing protocols at https://plos.org/protocols?utm_medium=editorial-email&utm_source=authorletters&utm_campaign=protocols

-->

# Author comment: Author response to Decision Letter 1

**DOI:** 10.1371/journal.pntd.0014539.r002

4 Jun 2026

Attachment Submitted filename: Reviewer_responses.docx

# Peer review: Decision Letter 1

**DOI:** 10.1371/journal.pntd.0014539.r003

**Authors:** Bruce A. Rosa

2 Jul 2026

Dear Mr. Richins,

We are pleased to inform you that your manuscript 'Molecular and morphologic characterization of Trichuris trichiura infecting free-roaming African vervets (Chlorocebus aethiops sabaeus) on the Caribbean Island of St. Kitts' has been provisionally accepted for publication in PLOS Neglected Tropical Diseases.

Before your manuscript can be formally accepted you will need to complete some formatting changes, which you will receive in a follow up email. A member of our team will be in touch with a set of requests.

Please note that your manuscript will not be scheduled for publication until you have made the required changes, so a swift response is appreciated.

IMPORTANT: The editorial review process is now complete. PLOS will only permit corrections to spelling, formatting or significant scientific errors from this point onwards. Requests for major changes, or any which affect the scientific understanding of your work, will cause delays to the publication date of your manuscript.

Should you, your institution's press office or the journal office choose to press release your paper, you will automatically be opted out of early publication. We ask that you notify us now if you or your institution is planning to press release the article. All press must be co-ordinated with PLOS.

Thank you again for supporting Open Access publishing; we are looking forward to publishing your work in PLOS Neglected Tropical Diseases.

Best regards,

Bruce A. Rosa

Academic Editor

PLOS Neglected Tropical Diseases

Maria Victoria Periago

Section Editor

PLOS Neglected Tropical Diseases

Shaden Kamhawi

co-Editor-in-Chief

PLOS Neglected Tropical Diseases

orcid.org/0000-0003-4304-636XX

Paul Brindley

co-Editor-in-Chief

PLOS Neglected Tropical Diseases

orcid.org/0000-0003-1765-0002

***********************************************************

The reviewers appreciated the revisions to the manuscript and now feel that it is ready for acceptance

p.p1 {margin: 0.0px 0.0px 0.0px 0.0px; line-height: 16.0px; font: 14.0px Arial; color: #323333; -webkit-text-stroke: #323333}span.s1 {font-kerning: none

Reviewer's Responses to Questions

**Key Review Criteria Required for Acceptance?**

As you describe the new analyses required for acceptance, please consider the following:

**Methods**

-Are the objectives of the study clearly articulated with a clear testable hypothesis stated?

-Is the study design appropriate to address the stated objectives?

-Is the population clearly described and appropriate for the hypothesis being tested?

-Is the sample size sufficient to ensure adequate power to address the hypothesis being tested?

-Were correct statistical analysis used to support conclusions?

-Are there concerns about ethical or regulatory requirements being met?

Reviewer #1: As outlined in the original review of this manuscript, the study design is clearly outlined. Sample size seems appropriate.

Methods clearly described. No concerns about ethical or regulatory requirements

being met.

Reviewer #2: (No Response)

**********

**Results**

-Does the analysis presented match the analysis plan?

-Are the results clearly and completely presented?

-Are the figures (Tables, Images) of sufficient quality for clarity?

Reviewer #1: Yes - thank you to the authors for adding the labels requested for Figure 1.

Reviewer #2: (No Response)

**********

**Conclusions**

-Are the conclusions supported by the data presented?

-Are the limitations of analysis clearly described?

-Do the authors discuss how these data can be helpful to advance our understanding of the topic under study?

-Is public health relevance addressed?

Reviewer #1: Yes - useful addition of commentary on the relevance of the different clades and limitations of the current study in the discussion.

Reviewer #2: (No Response)

**********

**Editorial and Data Presentation Modifications?**

Use this section for editorial suggestions as well as relatively minor modifications of existing data that would enhance clarity. If the only modifications needed are minor and/or editorial, you may wish to recommend “Minor Revision” or “Accept”.

Reviewer #1: No

Reviewer #2: (No Response)

**********

**Summary and General Comments**

Use this section to provide overall comments, discuss strengths/weaknesses of the study, novelty, significance, general execution and scholarship. You may also include additional comments for the author, including concerns about dual publication, research ethics, or publication ethics. If requesting major revision, please articulate the new experiments that are needed.

Reviewer #1: The reviewer's comments have been adequately addressed and I recommend acceptance of the article for publication.

Reviewer #2: Many thanks to the authors for addressing the comments thoroughly. I recommend to accept the manuscript.

**********

PLOS authors have the option to publish the peer review history of their article (what does this mean?). If published, this will include your full peer review and any attached files.

If you choose “no”, your identity will remain anonymous but your review may still be made public.

**Do you want your identity to be public for this peer review?**  For information about this choice, including consent withdrawal, please see our Privacy Policy.

Reviewer #1: No

Reviewer #2: No

# Editor report: Acceptance letter

**DOI:** 10.1371/journal.pntd.0014539.r004

**Authors:** Bruce A. Rosa

Dear Mr. Richins,

We are delighted to inform you that your manuscript, "Molecular and morphologic characterization of Trichuris trichiura infecting free-roaming African vervets (Chlorocebus aethiops sabaeus) on the Caribbean Island of St. Kitts," has been formally accepted for publication in PLOS Neglected Tropical Diseases.

We have now passed your article onto the PLOS Production Department who will complete the rest of the publication process. All authors will receive a confirmation email upon publication.

The corresponding author will soon be receiving a typeset proof for review, to ensure errors have not been introduced during production. Please review the PDF proof of your manuscript carefully, as this is the last chance to correct any scientific or type-setting errors. Please note that major changes, or those which affect the scientific understanding of the work, will likely cause delays to the publication date of your manuscript. Note: Proofs for Front Matter articles (Editorial, Viewpoint, Symposium, Review, etc...) are generated on a different schedule and may not be made available as quickly.

Soon after your final files are uploaded, the early version of your manuscript will be published online unless you opted out of this process. The date of the early version will be your article's publication date. The final article will be published to the same URL, and all versions of the paper will be accessible to readers.

For Research Articles, you will receive an invoice from PLOS for your publication fee after your manuscript has reached the completed accept phase. If you receive an email requesting payment before acceptance or for any other service, this may be a phishing scheme. Learn how to identify phishing emails and protect your accounts at https://explore.plos.org/phishing.

Thank you again for supporting open-access publishing; we are looking forward to publishing your work in PLOS Neglected Tropical Diseases.

Best regards,

Shaden Kamhawi

co-Editor-in-Chief

PLOS Neglected Tropical Diseases

Paul Brindley

co-Editor-in-Chief

PLOS Neglected Tropical Diseases
